# Integrative taxonomy and analysis of species richness patterns of nocturnal Darwin wasps of the genus *Enicospilus* Stephens (Hymenoptera, Ichneumonidae, Ophioninae) in Japan

**DOI:** 10.3897/zookeys.990.55542

**Published:** 2020-11-10

**Authors:** So Shimizu, Gavin R. Broad, Kaoru Maeto

**Affiliations:** 1 Laboratory of Insect Biodiversity and Ecosystem Science, Graduate School of Agricultural Science, Kôbe University, Rokkôdaichô 1–1, Nada, Kôbe, Hyôgo 657–8501, Japan Kôbe University Kôbe Japan; 2 DC and Overseas Challenge Program for Young Researchers, Japan Society for the Promotion of Science, Tôkyô, Japan The Natural History Museum London United Kingdom; 3 Depertment of Life Sciences, the Natural History Museum, Cromwell Road, London SW7 5BD, UK Japan Society for the Promotion of Science Tokyo Japan

**Keywords:** Biogeography, DNA barcoding, East Asia, Japanese archipelago, latitudinal diversity gradient (LDG), new species, temperate region, tropical region

## Abstract

The predominantly tropical ophionine genus *Enicospilus* Stephens, 1835 is one of the largest genera of Darwin wasps (Hymenoptera, Ichneumonidae), with more than 700 extant species worldwide that are usually crepuscular or nocturnal and are parasitoids of Lepidoptera larvae. In the present study, the Japanese species of *Enicospilus* are revised using an integrative approach (combined morphology and DNA barcoding). On the basis of 3,110 specimens, 47 *Enicospilus* species are recognised in Japan, eight of which are new species (*E. acutus* Shimizu, **sp. nov.**, *E. kunigamiensis* Shimizu, **sp. nov.**, *E. limnophilus* Shimizu, **sp. nov.**, *E. matsumurai* Shimizu, **sp. nov.**, *E. pseudopuncticulatus* Shimizu, **sp. nov.**, *E. sharkeyi* Shimizu, **sp. nov.**, *E. takakuwai* Shimizu, **sp. nov.**, and *E. unctus* Shimizu, **sp. nov.**), seven are new records from Japan (*E. jilinensis* Tang, 1990, *E. laqueatus* (Enderlein, 1921), *E. multidens* Chiu, 1954, **stat. rev.**, *E. puncticulatus* Tang, 1990, *E. stenophleps* Cushman, 1937, *E. vestigator* (Smith, 1858), and *E. zeugos* Chiu, 1954, **stat. rev.**), 32 had already been recorded in Japan; three (*E. biharensis* Townes, Townes & Gupta, 1961, *E. flavicaput* (Morley, 1912), and *E. merdarius* (Gravenhorst, 1829)) have been erroneously recorded from Japan based on misidentifications, and four names that were previously on the Japanese list are deleted through synonymy. The following taxonomic changes are proposed: *E. vacuus* Gauld & Mitchell, 1981, **syn. nov.** (= *E. formosensis* (Uchida, 1928)); *E. multidens***stat. rev.**; *E. striatus* Cameron, 1899, **syn. nov.** = *E. lineolatus* (Roman, 1913), **syn. nov.** = *E. uniformis* Chiu, 1954, **syn. nov.** = *E. flatus* Chiu, 1954, **syn. nov.** = *E. gussakovskii* Viktorov, 1957, **syn. nov.** = *E. striolatus* Townes, Townes & Gupta, 1961, **syn. nov.** = *E. unicornis* Rao & Nikam, 1969, **syn. nov.** = *E. unicornis* Rao & Nikam, 1970, **syn. nov.** (= *E. pungens* (Smith, 1874)); *E. iracundus* Chiu, 1954, **syn. nov.** (= *E. sakaguchii* (Matsumura & Uchida, 1926)); *E. sigmatoides* Chiu, 1954, **syn. nov.** (= *E. shikokuensis* (Uchida, 1928)); *E. yamanakai* (Uchida, 1930), **syn. nov.** (= *E. shinkanus* (Uchida, 1928)); *E. ranunculus* Chiu, 1954, **syn. nov.** (= *E. yezoensis* (Uchida, 1928)); and *E. zeugos***stat. rev.** = *E. henrytownesi* Chao & Tang, 1991, **syn. nov.** In addition, the following new regional and country records are also provided: *E. flavocephalus* (Kirby, 1900), *E. puncticulatus*, and *E. vestigator* from the Eastern Palaearctic region, *E. laqueatus* from the Eastern Palaearctic and Oceanic regions, and *E. maruyamanus* (Uchida, 1928) from the Oriental region; *E. abdominalis* (Szépligeti, 1906) from Nepal, *E. flavocephalus* from Laos, *E. formosensis* from Laos and Malaysia, *E. insinuator* (Smith, 1860) from Taiwan, *E. maruyamanus* from India and Philippines, *E. nigronotatus* Cameron, 1903, *E. riukiuensis* (Matsumura & Uchida, 1926), and *E. sakaguchii* from Indonesia, *E. pungens* from 14 countries (Australia, Bhutan, Brunei, Indonesia, Laos, Malaysia, Nepal, New Caledonia, Papua New Guinea, Philippines, Solomon Islands, Sri Lanka, Tajikistan, and Taiwan), and *E. yezoensis* from South Korea. An identification key to all Japanese species of *Enicospilus* is proposed. Although 47 species are recognised in the present study, approximately 55 species could potentially be found in Japan based on ACE and Chao 1 estimators. The latitudinal diversity gradient of *Enicospilus* species richness is also tested in the Japanese archipelago based on the constructed robust taxonomic framework and extensive samples. *Enicospilus* species richness significantly increases towards the south, contrary to the ‘anomalous’ pattern of some other ichneumonid subfamilies.

## Introduction

### Darwin wasps and Ophioninae

Darwin wasps, the family Ichneumonidae, are one of the most species-rich branches of the tree of life (Klopfstein et al. 2019), consisting of 1,601 genera and more than 25,000 valid species worldwide (Yu et al. 2016), with an estimated 60,000–100,000 species (Townes 1969; Gauld et al. 2002), but our knowledge currently lags far behind their true enormous diversity (Klopfstein et al. 2019). Darwin wasps are parasitoids of other holometabolous insects or spiders (very occasionally other arthropods or phytophagous) so they play an important role in terrestrial ecosystems as regulators of host insect populations (e.g., Townes 1969; Gauld 1991; Wahl 1993; Quicke 2015; Broad et al. 2018).

Ophioninae Shuckard are a species-rich subfamily of Darwin wasps, comprising 32 extant genera and more than 1,100 valid species worldwide (Yu et al. 2016; Shimizu and Lima 2018; Shaw and Voogd 2019) and understood to be a monophyletic group within the ‘higher ophioniformes’, a clade of koinobiont endoparasitoids, mostly attacking Lepidoptera (e.g., Gauld 1985; Quicke et al. 2009; Bennett et al. 2019). Many members of the Ophioninae have morphological features associated with crepuscular and/or nocturnal behaviour (i.e., long antennae, large body, large ocelli, testaceous colour, etc.). This suite of characteristics is sometimes referred to as an “ophionoid facies” (Gauld and Huddleston 1976). Not only ophionines but also other nocturnal ichneumonoids (e.g., the genera *Netelia* Gray (Tryphoninae), *Cidaphus* Förster (Mesochorinae), and the subfamily Xiphozelinae van Achterberg (Braconidae)) often convergently share these features (e.g., Shimizu 2017), although a few species of some ophionine genera (e.g., the genera *Dictyonotus* Kriechbaumer, *Heinrichiella* Hedwig, *Hellwigiella* Szépligeti, and *Hellwigia* Gravenhorst) have morphology typical of diurnal species (i.e., smaller ocelli, shorter antennae, darker body colour, etc.), like other diurnal ichneumonids.

### The genus *Enicospilus*

The cosmopolitan genus *Enicospilus* Stephens is the largest genus within the Ophioninae, with more than 700 valid species (e.g., Broad and Shaw 2016; Yu et al. 2016; Shimizu 2017; Gadallah et al. 2017; Johansson 2018; Shimizu 2020) and an estimate of more than 1,000 species (Townes 1971), suggesting that this is the most species-rich genus in not only the subfamily but also Ichneumonidae as a whole. *Enicospilus* is a predominantly tropical genus, with more than 75% of the species occurring in the tropics (Gauld and Mitchell 1981).

As well as almost all other genera of Ophioninae, tropical species of *Enicospilus* are taxonomically relatively well known thanks to Ian Gauld’s comprehensive and ground-breaking regional revisions (e.g., Gauld and Mitchell 1978, 1981; Gauld 1988). However, fewer papers have been published for the temperate species, most of them focusing on the Western Palaearctic species (e.g., Viktorov 1957; Broad and Shaw 2016; Johansson 2018) and a few on the Eastern Palaearctic species (e.g., Uchida 1928; Chiu 1954; Tang 1990). Therefore, the true species diversity of the temperate fauna is largely unknown. Although a total of 39 species of *Enicospilus* have been recognised in Japan (Shimizu 2017; Table 1), the *Enicospilus* fauna of Japan and the far east of Asia has been particularly poorly known, with many taxonomic problems persisting. No identification keys to the Japanese species of *Enicospilus* have been published after Uchida (1928), while the keys provided by Chiu (1954) and Gauld and Mitchell (1981) include few Japanese species. Hence, a comprehensive study of the Japanese fauna is much needed to understand their true species diversity in the temperate region.

**Table 1.** Summary of taxonomic histories of the Japanese species of *Enicospilus*. Valid species names are in bold. Total species numbers were calculated as follow: (a) previous ‘total species number’, minus (b) numbers of ‘deleted species or names’, plus (c) number of added species (i.e., ‘new species or names’ plus ‘new records’). *Enicospilus combustus and *E. ramidulus* have been sometimes treated as a single species (e.g., Viktorov 1957; Townes et al. 1965; Gauld and Mitchell 1981) but we don’t agree with this and follow the recent papers (e.g., Broad and Shaw 2016).

New species or names	New records	Deleted species or names	Total species number
**Smith (1874)**			
**1**	**0**	**0**	**1** (= 0 - 0 + (1 + 0))
*** pungens ***			
**Matsumura and Uchida (1926)**			
**5**	**2**	**0**	**8** (= 1 - 0 + (5 + 2))
* analis *	*** flavicaput ***		
* okinawensis *	* striatus *		
*** riukiuensis ***			
*** sakaguchii ***			
* similis *			
**Uchida (1928)**			
**7**	**5**	**1**	**19** (= 8 - 1 + (7 + 5))
*combustus* var. ***shikokuensis***	combustus*	*similis* (synonym of ***flavocephalus***)	
* fuscomaculatus *	*** flavocephalus ***		
*** maruyamanus ***	*** merdarius ***		
* orientalis *	ramidulus*		
*** pudibundae ***	* reticulatus *		
*** yezoensis ***			
*** yonezawanus ***			
**Uchida (1930)**			
**1**	**0**	**0**	**20** (= 19 - 0 + (1 + 0))
*** yamanakai ***			
**Yasumatsu (1934)**			
**1**	**1**	**0**	**22** (= 20 - 0 + (1 + 1))
*fuscomaculatus yakushimensis*	* taiwanus *		
**Sonan (1940)**			
**1**	**1**	**0**	**24** (= 22 - 0 + (1 + 1))
* yanagiharai *	* mushauus *		
**Chiu (1954)**			
**8**	**4**	**0**	**36** (= 24 - 0 + (8 + 4))
* gephyrus *	* nigrivenalis *		
*** iracundus ***	* nigrostemmaticus *		
* multidens *	*** shinkanus ***		
* nasutus *	* sinuatus *		
* saepis *			
*** tripartitus ***			
* uniformis *			
* zyzzus *			
**Uchida (1955)**			
**1**	**0**	**2**	**35** (= 36 - 2 + (1 + 0))
*hirayamai* (new name for *orientalis*)		*orientalis* (homonym)	
		*multidens* (synonym of ***combustus***)	
**Uchida (1956)**			
**1**	**0**	**0**	**36** (= 35 - 0 + (1 + 0))
* microstriatellus *			
**Townes (1958)**			
**0**	**1**	**0**	**37** (= 36 - 0 + (0 + 1))
	* nocturnus *		
**Townes et al. (1961)**			
**0**	**1**	**1**	**37** (= 37 - 1 + (0 + 1))
	*** erythrocerus ***	*hirayamai* = *orientalis* (synonym of ***erythrocerus***)	
**Gauld and Mitchell (1981)**			
**1**	**12**	**18**	**32** (= 37 - 18 + (1 + 12))
*** vacuus ***	*** aciculatus ***	*analis* (synonym of ***sauteri***)	
	*** capensis ***	*fuscomaculatus* (synonym of ***nigropectus***)	
	*** dasychirae ***	*gephyrus* (synonym of ***javanus***)	
	*** formosensis ***	*microstriatellus* (synonym of ***yonezawanus***)	
	*** insinuator ***	*mushauus* (synonym of ***pseudoconspersae***)	
	*** javanus ***	*nasutus* (synonym of ***riukiuensis***)	
	*** lineolatus ***	*nigrivenalis* (synonym of ***melanocarpus***)	
	*** melanocarpus ***	*nigrostemmaticus* (synonym of ***nigropectus***)	
	*** nigropectus ***	*nocturnus* (synonym of ***melanocarpus***)	
	*** pseudoconspersae ***	*okinawensis* (synonym of ***aciculatus***)	
	*** sauteri ***	*reticulatus* (synonym of ***melanocarpus***)	
	*** signativentris ***	*saepis* (synonym of ***formosensis***)	
		*sinuatus* (misidentification of ***erythrocerus***)	
		*striatus* (synonym of ***lineolatus***)	
		*taiwanus* (synonym of ***signativentris***)	
		*uniformis* (synonym of ***lineolatus***)	
		*yanagiharai* (synonym of ***capensis***)	
		*zyzzus* (synonym of ***insinuator***)	
**Gupta (1987)**			
**0**	**1**	**0**	**33** (= 32 - 0 + (0 +1))
	*** biharensis ***		
**Konishi (1993)**			
**0**	**3**	**0**	**36** (= 33 - 0 + (0 +3))
	*** concentralis ***		
	*** nigribasalis ***		
	*** nigristigma ***		
**Watanabe and Yamauchi (2014)**			
**0**	**0**	**1**	**35** (= 36 - 1 + (0 + 0))
		*fuscomaculatus yakushimensis* (= ***nigropectus***)	
**Shimizu and Maeto (2016)**			
**0**	**3**	**0**	**38** (= 35 - 0 + (0 + 3))
	*** abdominalis ***		
	*** nigronotatus ***		
	*** xanthocephalus ***		
**Shimizu (2017)**			
**1**	**0**	**0**	**39** (= 38 - 0 + (1 + 0))
*** kikuchii ***			

### Integrative taxonomy

Species delimitation and taxonomic revisions of poorly known, hyperdiverse groups, such as Darwin wasps, based on traditional morphology-based taxonomy is challenging, but has recently been rapidly improved by advancing integrative approaches that combine multiple perspectives (population genetics, morphometrics, behaviour, host, chemical composition, etc.). A combined morphological and DNA barcoding (partial sequencing of a mitochondrial protein-coding gene, cytochrome c oxidase 1, CO1) approach is the most straightforward method and has been used by many authors for various taxa (e.g., Gibbs 2009; Fernandez-Triana 2010; Schwarzfeld and Sperling 2014; Pentinsaari et al. 2019). For the latter approach, the appropriate sequence divergence distance to delineate species is still open to debate and will differ among taxa and authors, but 2–5% (especially 2%) have been frequently used (e.g., Hebert et al. 2004; Lin et al. 2015; Janzen et al. 2017; Wang et al. 2018; Meierotto et al. 2019).

Species delineation of ophionines has been considered to be more difficult than many other lineages of insects, including other subfamilies of Ichneumonidae, because extreme similarities in morphology do not provide enough diagnostic characters (although the pattern and shapes of fore wing sclerites in *Enicospilus* offer some very useful characters, lacking for other large genera, e.g., *Ophion* Fabricius), and some lineages exhibit a wide range of intraspecific morphological variation (e.g., Gauld and Mitchell 1981; Schwarzfeld and Sperling 2015; Johansson and Cederberg 2019). In particular, body colour frequently changes within a species, as in some other Ichneumonoidea, with differences in temperature or habitat, which has caused taxonomic confusion (e.g., Shu-Sheng and Carver 1982; Abe et al. 2013; Ito et al. 2015; Shimizu et al. 2019). Hence, careful evaluation of morphological characters is needed for more accurate species recognition.

### Latitudinal diversity gradient in species richness

There has been much research into patterns of Darwin wasp species richness across latitudinal gradients. This has been summarised fairly recently by Santos and Quicke (2011), Jones et al. (2012) and Veijalainen et al. (2012, 2013). Observations that there were apparently small numbers of individuals and species of Ichneumonidae in the tropics led to the idea that this represented one of a few insect examples of an ‘anomalous’ species richness gradient (e.g., Owen and Owen 1974; Janzen 1981). Various potential mechanisms have been proposed to explain the relative lack of ichneumonid species in the tropics, e.g., the resource fragmentation hypothesis by Janzen and Pond (1975), the predation hypothesis by Rathcke and Price (1976), and the “nasty” host hypothesis by Gauld et al. (1992). However, the increase in data from more recent large scale collecting has shown that the pattern is more complicated, with some ichneumonid subfamilies being potentially more species-rich at lower latitudes, some less species-rich, but that robust data are still lacking to accurately describe patterns of species richness, let alone propose mechanisms to explain the patterns (Santos and Quicke 2011; Veijalainen et al. 2012). Nevertheless, there are robust findings, such as that parasitoids of insect groups that are more diverse at higher latitudes are similarly more species-rich at higher latitudes, such as sawfly parasitoids of the subfamilies Ctenopelmatinae and Tryphoninae, and Diplazontinae, parasitoids of aphidophagous Syrphidae (Diptera) (Quicke 2015).

Most ophionines are typical nocturnal koinobiont parasitoids with their centre of species diversity in the (sub-)tropics (e.g., Gauld 1985, 1988; Gauld and Mitchell 1981), with a few exceptions, e.g., the nocturnal genus *Ophion* is most abundant in cooler temperate regions (e.g., Schwarzfeld and Sperling 2014; Schwarzfeld et al. 2016), as is a Southern equivalent of *Ophion*, *Alophophion* Cushman (Alvarado 2014). Gauld (1987) suggested that their nocturnal habit is one factor that has adapted ophionines to tropical rainforest, where they would be exposed to high predation pressure in the daytime, based on the predation hypothesis. Moreover, Quicke (2015) pointed out that daytime temperatures are too hot for much active host searching in the lowland tropics so that nocturnal habits are more suitable than diurnal there. However, this research field is still under discussion (e.g., Gauld and Mitchell 1981; Gauld 1995; Jones et al. 2012) and needs additional data.

### Aims of the present study

The Japanese archipelago is located in a long line between ca. 20–45°N, approximately 3,000 km from south to north, ranging from the southern subtropical to northern and high elevational subarctic zones, containing a high diversity of ecological habitats. Biogeographically, it also includes the Oceanic, Oriental, and Palaearctic regions and is a melting pot of species originating from these regions, so in some ways one of the most interesting biodiversity hotspots (e.g., Mittermeier et al. 2004). However, taxonomic knowledge of the speciose genus *Enicospilus* in Japan has been complicated due to the lack of revisionary studies based on comprehensive sampling and the difficulties of the traditional morphology-based taxonomy. For these reasons, to reveal the species diversity of *Enicospilus* in Japan, we revise the Japanese fauna using integrative approaches (i.e., combined morphological and DNA barcoding approach) and estimate their species richness based on the large number of specimens examined. In addition, as a genus that seems to be more species-rich at lower latitudes on a global scale, it is of interest to know whether *Enicospilus* diversity follows this trend at a local scale too, within the Japanese archipelago. Hence, we test a latitudinal diversity pattern in this group of nocturnal koinobiont parasitoids in Japan based on the constructed robust taxonomic framework.

## Materials and methods

### Specimens examined

**Figure 1.**
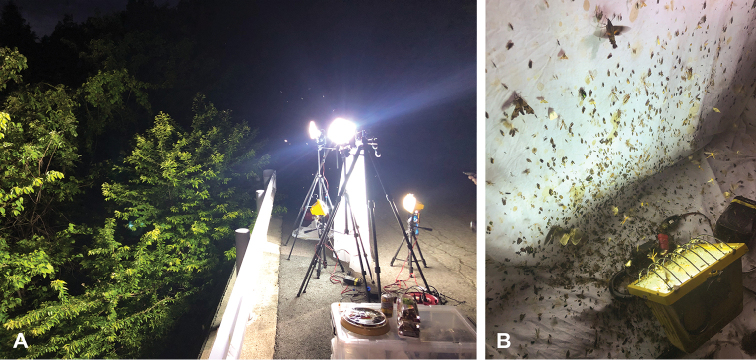
Field collection **A** High Intensity Discharge (HID) light trap equipment used by the first author **B** nocturnal insects attracted to the HID light on a white sheet.

The specimens examined were studied in or borrowed from insect collections, or newly collected for the present study, mainly using High Intensity Discharge (HID) light traps (Fig. 1) by the first author. A total of 3,110 specimens of *Enicospilus*, of which 1,863 are from Japan and 1,247 from other countries, were examined. Type specimens examined are listed in the main text, but the data for non-types are listed in Suppl. material 1: Table S1. The first author also examined, however, many more specimens (more than 20,000 specimens) from all over the world to develop an improved perspective on species criteria and range of variation within *Enicospilus*.

### Morphological observations and figures

Specimens were observed using a stereoscopic microscope (SMZ1500, Nikon, Tôkyô, Japan). Multi-focus photographs were taken using a single lens reflex camera (α7II, Sony, Tôkyô, Japan) with a micro-lens (LAOWA 25 mm F2.8 2.5–5× ULTRA MACRO, Anhui Changgeng Optics Technology Co., Ltd, Hefei, China and A FE 50mm F2.8 Macro SEL50M28, Sony, Tôkyô, Japan), captured in RAW format, developed using Adobe Lightroom CC v.2.2.1 (Adobe Systems Inc., San Jose, CA, USA), and stacked using Zerene Stacker v.1.04 (Zerene Systems LLC., Richland, WA, USA). The original maps were generated using SimpleMappr (Shorthouse 2010). All figures were edited in Adobe Illustrator CC v.23.0.2 and Photoshop CC v.20.0.4 (Adobe Systems Inc., San Jose, CA, USA).

### Terms, indices, and abbreviations

**Figure 2.**
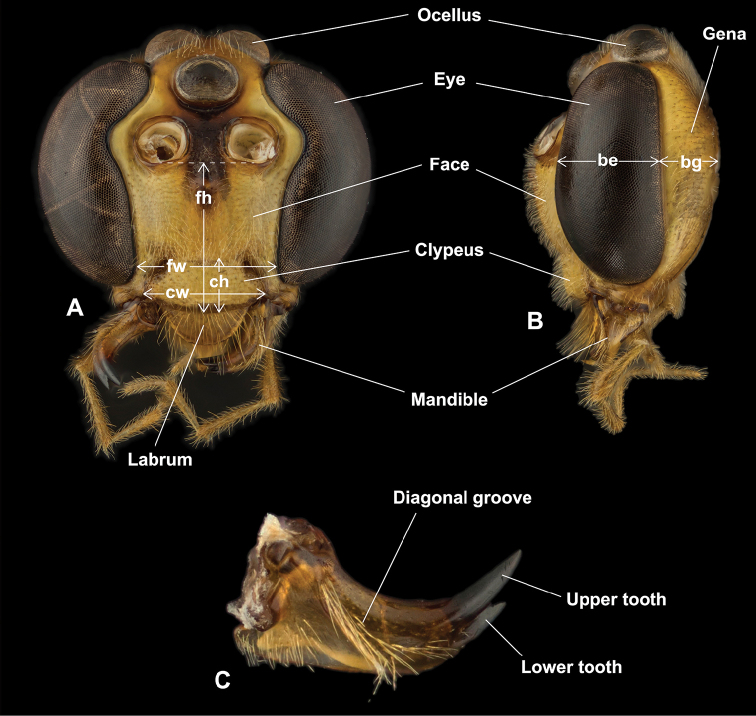
Morphological terms and measurement characters for head and mandible **A** head in frontal view (fh = face height, fw = face width, ch = clypeus height, cw = clypeus width) **B** head in lateral view (be = breadth of eye, bg = breadth of gena; GOI = be / bg) **C** mandible in outer view.

**Figure 3.**
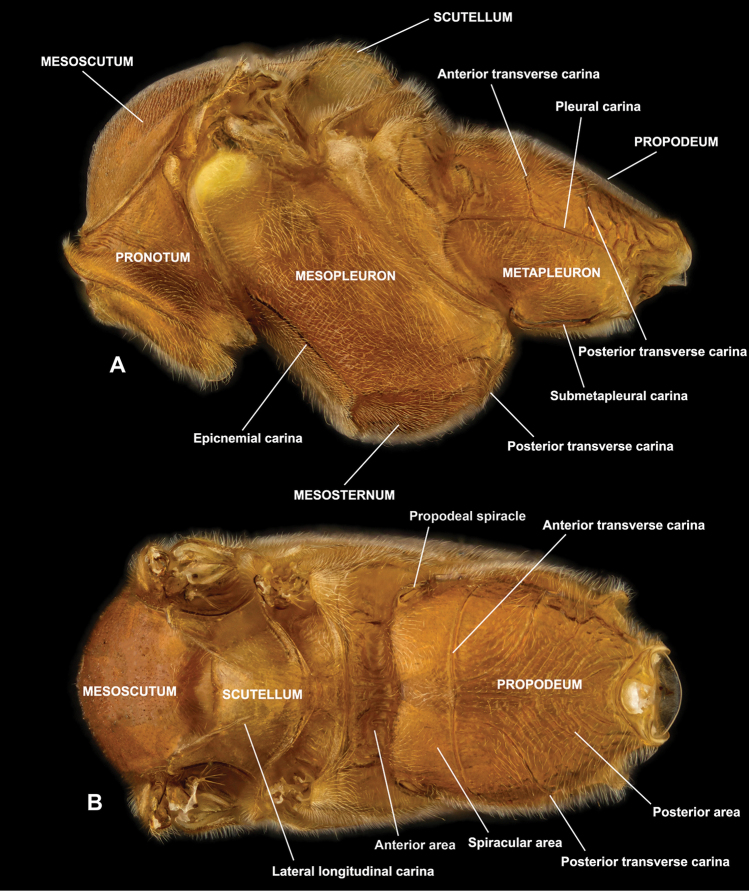
Morphological terms for mesosoma **A** lateral view **B** dorsal view.

**Figure 4.**
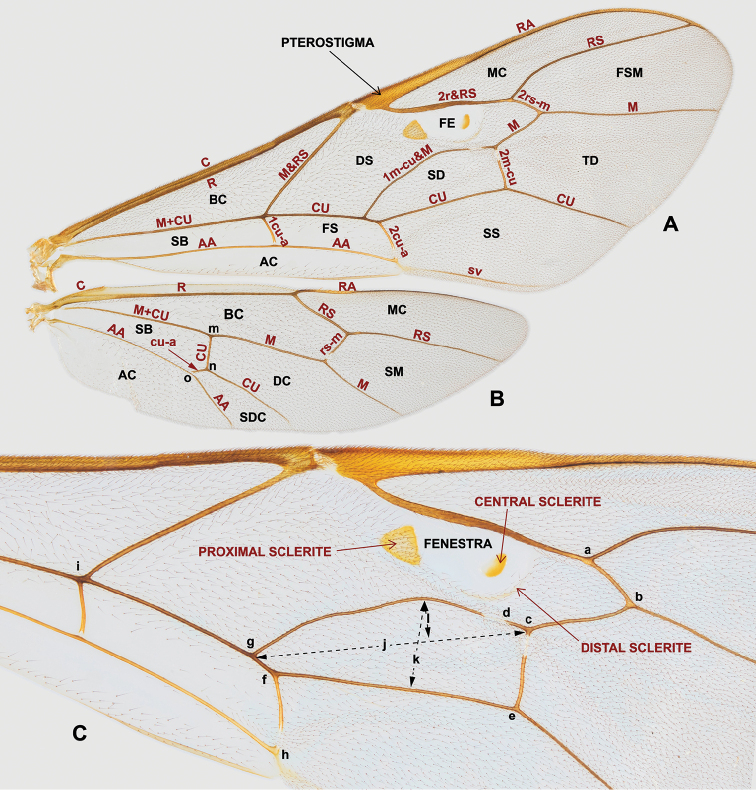
Morphological terms and measurement characters for wings **A** fore wing (AC = anal cell, BC = basal cell, DS = discosubmarginal cell, FE = fenestra, FS = first subdiscal cell, FSM = fourth submarginal cell, MC = marginal cell, SD = second discal cell, SB = subbasal cell, SS = second subdiscal cell, TD = third discal cell) **B** hind wing (NI = mn / no, AC = anal cell, BC = basal cell, DC = discal cell, MC = marginal cell, SB = subbasal cell, SDC = subdiscal cell, SM = submarginal cell) **C** central part of fore wing (AI = cd / ab, CI = gf / fh, DI = k / fe, ICI = ab / bc, SDI = ef / gi, SI = l / j, SRI = ce / ef). Brown letters indicate veins and sclerites.

**Figure 5.**
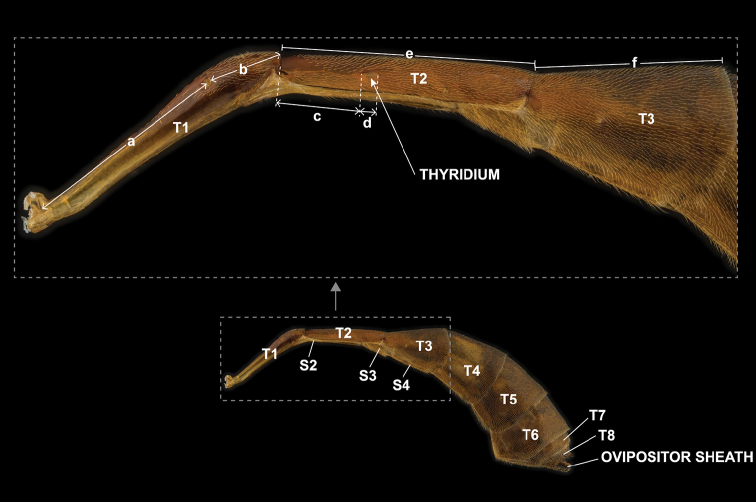
Morphological terms and measurement characters for metasoma (DMI = e / f, PI = a / b, THI = c / d).

The morphological terms mainly follow Broad et al. (2018). Terms for surface microsculpture follow Eady (1968) and Gauld and Mitchell (1981). The terms, abbreviations, and indices for head, wings and metasoma follow mainly Shimizu and Lima (2018), Shimizu (2020) and partly Broad et al. (2018). The indices, terms, and abbreviations used in this paper are illustrated in Figs 2–5, and the indices are listed in Table 2. The following non-morphological abbreviations are also used:

**AT** allotype

**HT** holotype

**LCT** lectotype

**LT** light traps

**MsT** Malaise traps

**PT** paratype

**SYT** syntype

The abbreviations for specimen repositories used in the present paper (some only in Suppl. material 1: Table S1) are listed in Table 3. In addition, the following abbreviations for collections are used:

**JMC** J. Minamikawa collection at NIAES

**KUSIG** K. Kusigemati collection at SEHU

**SAC** S. Asahina collection at NSMT

**SMCM** S. Momoi collection at MNHA

**TIC** T. Ishii collection at NIAES

**Table 2.** Indices used in the present paper.

Indices		Formula
**Head (Fig. 2B)**		
GOI	Geno-Orbital Index	maximum breadth of eye in profile [be] / maximum breadth of gena in same line [bg]
**Fore wing (Fig. 4A, C)**		
AI	Alar Index	length of 1m-cu&M between 2m-cu and bulla [cd] / length of 2rs-m [ab]
CI	Cubital Index	length of CU between 1m-cu&M and 2cu-a [gf] / length of 2cu-a [fh]
DI	Discoidal Index	maximum vertical distance between CU (between 2cu-a and 2m-cu) and 1m-cu&M [k] / length of CU between 2cu-a and 2m-cu [fe]
ICI	Intercubital Index	length of 2rs-m [ab] / length of M between 2m-cu and 2rs-m [bc]
SDI	Second Discoidal Index	length of CU between 2cu-a and 2m-cu [ef] / length of CU between M&RS and 1m-cu&M [gi]
SI	Sinuousness Index	maximum length between 1m-cu&M and a straight line connecting the intersection of M, 2m-cu, and 1m-cu&M and the intersection of 1m-cu&M and CU [l] / distance between the intersection of M, 2m-cu, and 1m-cu&M [c] and the intersection of 1m-cu&M and CU [g] [j]
SRI	Second Recurrent Index	length of 2m-cu [ce] / length of CU between 2cu-a and 2m-cu [ef]
**Hind wing (Fig. 4B)**		
NI	Nervellar Index	length of CU between M and cu-a [mn] / length of cu-a [no]
**Metasoma (Fig. 5)**		
DMI	Dorsal Metasomal Index	length of dorsum of T2 [e] / length of dorsum of T3 [f]
PI	Petiolar Index	distance between base of T1 and anterior margin of spiracle [a] / distance between posterior margin of spiracle and apex of T1 [b]
THI	Thyridium Index	distance between anterior margin of T2 and anterior margin of thyridium [c] / maximum diameter of thyridium [d]

**Table 3.** Abbreviations for repositories consulted (not all are referred to in the main text and some are only in Suppl. material 1: Table S1).

Abbreviations	Repositories
ANIC	Australian National Insect Collection, Canberra, Australia
CNC	Canadian National Collection of Insects, Ottawa, Canada
DEI	Senckenberg Deutsches Entomologisches Institut, Müncheberg, Germany
ELMU	Entomological Laboratory, Meijô University, Nagoya, Japan
EMUS	Utah State University Insect Collection (= American Entomological Institute: AEI), Department of Biology, Utah State University, Logan, Utah, USA
EUM	Ehime University Museum, Matsuyama, Japan
FAFU	The Parasitic Hymenoptera Collection of the Institute of Beneficial Insect, College of Plant Protection, Fujian Agriculture and Forestry University, Fuzhou, China
FZLU	Fachbereich Zoologie, Luther-Universitat, Halle, Germany
HMNH	Hiwa Museum for Natural History, Shôbara, Japan
IRSNB	Institut Royal des Sciences Naturelles de Bel-gique, Brussels, Belgium
IZPAN	Instytut Zoologiczny Polska Akademia Nauk, Warszawa, Poland
KPMNH	Kanagawa Prefectural Museum of Natural History, Odawara, Japan
KUEC	Entomological Laboratory, Kyûshû University, Fukuoka, Japan
MCZ	Museum of Comparative Zoology, Cambridge, USA
MNHA	Museum of Nature and Human Activities, Sanda, Japan
MNHN	Museum national d`Histoire naturelle, Paris, France
MUC	Marathwada University Collection, Aurangabad, India
NIAES	Institute for Agro-Environmental Sciences, NARO (= National Institute for Agro-Environmental Sciences), Tsukuba, Japan
NHMUK	Natural History Museum, London, United Kingdom (formerly BMNH)
NM	Naturhistorisches Museum, Vienna, Australia
NR	Naturhistoriska Riksmuseet, Stockholm, Sweden
NSMT	National Museum of Nature and Science, Tsukuba, Japan
OMNH	Ōsaka Museum of Natural History, Ōsaka, Japan
OUMNH	Oxford University Museum of Natural History (= the Hope Entomological Collection), Oxford, United Kingdom
SEHU	The Laboratory of Systematic Entomology (= Entomological Institute: EIHU), Hokkaidô University, Sapporo, Japan
TARI	Taiwan Agricultural Research Institute Council of Agriculture, Executive Yuan, Taichung, Taiwan
TM	Termeszettudomanyi Muzeum, Budapest, Hungary
TPM	Tochigi Prefectural Museum, Utsunomiya, Japan
ZIUU	Zoological Institute, University of Uppsala, Sweden
ZSI	Zoological Survey of India, Calcutta, India

### Literature records

There are many published distribution and host records that cannot be verified as we cannot access all voucher specimens or host remains underpinning these literature records; *Enicospilus* species have frequently been misidentified, as have their hosts, and there are various reasons why potential hosts and parasitoids are mis-associated (see Shaw 1994). There is no point repeating dubious distribution and host records, so we have just broadly summarised distributions and host ranges. However, Japanese and some reliable recent extralimital host records (e.g., Broad and Shaw 2016) are emphasised.

### Order of prefectures

We used the following order of Japanese Prefectures in the distribution of Japan: [Hokkaidô] Hokkaidô; [Tôhoku] Aomori, Akita, Iwate, Yamagata, Miyagi, Fukushima; [Hokuriku] Niigata, Toyama, Ishikawa, Fukui; [Kantô-Kôshin] Ibaraki, Tochigi, Gunma, Nagano, Yamanashi, Saitama, Tôkyô, Kanagawa, Chiba; [Tôkai] Gifu, Aichi, Shizuoka, Mie; [Kinki] Kyôto, Shiga, Ōsaka, Hyôgo, Nara, Wakayama; [Chûgoku] Tottori, Shimane, Okayama, Hiroshima, Yamaguchi; [Shikoku] Kagawa, Tokushima, Ehime, Kôchi; [Kyûshû] Fukuoka, Saga, Nagasaki, Ōita, Kumamoto, Miyazaki, Kagoshima; [Ryûkyûs] Kagoshima, Okinawa; [Ogasawara] Tôkyô. Honshû consists of Tôhoku, Hokuriku, Kantô-Kôshin, Tôkai, Kinki, and Chûgoku. Prefectures are ordered basically from North to South.

### DNA barcoding analysis

#### Taxon sampling

To test our assessment of taxonomy based on morphology, we employed a DNA barcoding approach. A total of 168 sequences of CO1 from 41 of 47 Japanese species of *Enicospilus* (including the species described in the present paper) and other *Enicospilus* species from the Eastern Palaearctic, Neotropical, and Oriental regions were sampled: 125 of those were newly sequenced and deposited in DNA Data Bank of Japan (DDBJ), and 43 were obtained from the Barcode of Life database (BOLD) and GenBank. We selected seven ophionine genera: *Afrophion* Gauld, *Dicamptus* Szépligeti, *Hellwigiella*, *Leptophion* Cameron, *Ophion*, *Rhynchophion* Enderlein, and *Thyreodon* Brullé as outgroups.

The species, identifiers for the specimens, collection localities, sample codes, and accession numbers for all terminal taxa used in the analyses are listed in Suppl. material 2: Table S2.

#### DNA extraction, amplification, and sequencing

Most specimens for DNA analysis were dried specimens borrowed from collections. Some specimens were newly collected for the present study; these were stored in 80.0–99.9% ethanol and, after DNA extraction, mounted as dried specimens, currently deposited in the respective insect collections. DNA was extracted from a single right mid leg or both right mid and hind legs using the DNeasy Blood and Tissue Kit (Qiagen, Düsseldorf, Germany).

Partial sequences of CO1 were amplified using primers designed by Folmer et al. (1994): LCO1490 (5’ – GGT CAA CAA ATC ATA AAG ATA TTG G – 3’) and HCO2198 (5’ – TAA ACT TCA GGG TGA CCA AAA AAT CA – 3’). Polymerase chain reactions (PCR) were conducted using the KOD FX NEO kit (Toyobo, Ōsaka, Japan). PCR conditions were 2 min at 94 °C as an initial denaturation, and 35 cycles of 10 s at 98 °C of denaturation, 30 s at 48 °C of annealing, and 30 s at 68 °C of extension, then a final extension at 72 °C for 10 min. PCR products were purified using Illustra GFX kit (GE Healthcare Life Sciences, Marlborough, USA). The purified PCR products were amplified with the same primers using the BigDyeTM Terminator v.3.1 Cycle Sequencing kit (Applied Biosystems, Waltham, USA). In order to save cost, cycle sequencing reactions were carried out in 10.0 μl total volume consisting of 0.5 μl Readdy Reaction Mix, 2.0 μl 5× Sequencing Buffer, 1.2 μl each primer (10.0 pmol), 5.0 μl PCR products (10.0 ng / 100 bp), and 1.3 μl Deionized water. Cycling conditions were 25 cycles of 10 s at 96 °C, 5 s at 50 °C, and 4 min at 60 °C. Products were purified using the 3.0 M sodium acetate, 95% ethanol, 70% ethanol, and Hi-Di formamide. Cycle sequencing products were run on an ABI Prism 3100 Genetic Analyzer (Applied Biosystems, Waltham, USA), and the forward and reverse sequences were assembled using the DNA Dynamo Sequence Analyse Software (Blue Tractor Software, North Wales, UK). Some sequences were incomplete; however, they were included in analyses with the gaps coded as missing data.

#### Multiple sequence alignments

We conducted multiple sequence alignments in MAFFT v.7.409 (Katoh and Standley 2013), using default parameters: the final dataset is 626 bp in length, without indels.

#### Analyses

Analyses were performed with Bayesian Inference (BI) and maximum likelihood (ML) approaches. Each codon position within the CO1 fragment was treated as a different data block, and the best-fit substitution model was determined using PartitionFinder v.2.1.1 (Lanfear et al. 2017) with the greedy search algorithm under the corrected Akaike information criterion (AICc): the selected model was the GTR+I+Γ model for all positions. The BI analyses were conducted using MrBayes v.3.2.2 (Ronquist et al. 2012). We ran two independent runs of a Bayesian Markov chain Monte Carlo (MCMC) analysis of eight chains each, heating 0.1, random starting trees, and trees sampled every 1,000th generation for 10,000,000 generations. We considered that the two MCMC runs had converged if the average standard deviation of split frequencies was below 0.01 (Ronquist and Huelsenbeck 2003). Moreover, we checked for chain stationarity in Tracer v.1.6 (Rambaut and Drummond 2007). Then, we discarded half of the generations as a conservative burn-in, obtained estimates for the harmonic means of the likelihood scores from the remaining half of the generations using the sump command, and conducted a final check of the convergence of the runs by the value of a potential scale reduction factor (PSRF): if the runs were convergent enough, PSRF was less than 5% divergent from 1.0. Finally, a majority-rule consensus tree with the Bayesian inference posterior probabilities was obtained using the sumt command in MrBayes. The ML analysis was conducted in RAxML v.8.2.10 (Stamatakis 2014) with 1,000 bootstrap replications. We mapped the bootstrap percentages to each node of the reconstructed best-fit tree by the pgsumtree command in Phylogears v.2.2.0 (Tanabe 2008). The trees were checked and edited in FigTree v.1.4.3 (Rambaut 2006–2016) and Adobe Illustrator. The p-distances were calculated using MEGA v.10.0.5 (Kumar et al. 2018).

### Species richness pattern analysis

The latitudinal diversity gradient (LDG) of species richness in the Japanese archipelago was analysed based on the constructed robust taxonomic framework and extensive samples. We divided the Japanese archipelago into six latitudinal zones of equal intervals (Table 4) and analysed the LDG based on the species richness in each zone to separate the Oriental and Palaearctic regions and reduce the effect of sampling biases along the Japanese archipelago. Species richness was usually counted in the prefectural capitals (Table 4), only using data from the specimens examined in the present study (Suppl. material 1: Table S1), because literature data are sometimes unreliable. Incomplete label data were not included in the analysis. To exclude regional biases in sample number, saturation species richness in each zone was estimated by extrapolation methods based on Chao1 richness estimator in EstimateS v.9.1.0 (Colwell 2013). We used Spearman’s rank correlation to test correlations between latitudinal zones and species richness in each zone using R v.3.6.3 (R Core Development Team 2020).

**Table 4.** Latitudinal zones used in the latitudinal diversity gradient analysis.

Zones	Latitudinal ranges (LR)	Provinces	Prefectures	Points	Latitudes
A	42 ≤ LR < 45	Hokkaidô	Hokkaidô	Sapporo	43°03'51"N
B	39 ≤ LR < 42	Tôhoku	Aomori	Aomori	40°49'28"N
			Akita	Akita	39°43'07"N
			Iwate	Morioka	39°42'13"N
C	36 ≤ LR < 39	Tôhoku	Yamagata	Yamagata	38°14'26"N
			Miyagi	Sendai	38°16'08"N
			Fukushima	Fukushima	37°45'00"N
		Hokuriku	Niigata	Niigata	37°54'08"N
			Toyama	Toyama	36°41'43"N
			Ishikawa	Kanazawa	36°35'40"N
			Fukui	Fukui	36°03'55"N
		Kantô-Kôshin	Ibaraki	Mito	36°20'29"N
			Tochigi	Utsunomiya	36°33'57"N
			Gunma	Maebashi	36°23'28"N
			Nagano	Nagano	36°39'05"N
D	33 ≤ LR < 36	Kantô-Kôshin	Yamanashi	Kôfu	35°39'50"N
			Saitama	Saitama	35°51'25"N
			Tôkyô	Shinjyuku	35°41'22"N
			Kanagawa	Yokohama	35°26'52"N
			Chiba	Chiba	35°36'17"N
		Tôkai	Gifu	Gifu	35°23'28"N
			Aichi	Nagoya	35°10'49"N
			Shizuoka	Shizuoka	34°58'37"N
			Mie	Tsu	34°43'49"N
		Kinki	Kyôto	Kyôto	35°01'17"N
			Shiga	Ōtsu	35°00'16"N
			Ōsaka	Ōsaka	34°41'11"N
			Hyôgo	Kôbe	34°41'29"N
			Nara	Nara	34°41'07"N
			Wakayama	Wakayama	34°13'34"N
		Chûgoku	Tottori	Tottori	35°30'13"N
			Shimane	Matsue	35°28'20"N
			Okayama	Okayama	34°39'42"N
			Hiroshima	Hiroshima	34°23'47"N
			Yamaguchi	Yamaguchi	34°11'09"N
		Shikoku	Kagawa	Takamatsu	34°20'25"N
			Tokushima	Tokushima	34°03'57"N
			Ehime	Matsuyama	33°50'30"N
			Kôchi	Kôchi	33°33'35"N
		Kyûshû	Fukuoka	Fukuoka	33°36'23"N
			Saga	Saga	33°14'58"N
			Ōita	Ōita	33°14'17"N
E	30 ≤ LR < 33	Kyûshû	Nagasaki	Nagasaki	32°44'41"N
			Kumamoto	Kumamoto	32°47'23"N
			Miyazaki	Miyazaki	31°54'40"N
			Kagoshima	Kagoshima	31°33'37"N
F	27 ≤ LR < 30	Ryûkyûs	Kagoshima	Amami	28°22'39"N
			Okinawa	Naha	26°12'45"N
		Ogasawara	Tôkyô	Ogasawara	27°05'39"N

To understand the regional pattern of sampling biases, numbers of four categories (specimens, collection events, collector, and species) were counted for each area. Each pattern is shown in the heat maps.

To infer the total species richness of the Japanese *Enicospilus*, the individual-based rarefaction curves were estimated based on ACE and Chao 1 richness estimators using EstimateS, with 100 runs of randomizations and the classic formula for Chao 1.

## Results

### Integrative taxonomy

A total of 47 morphospecies were recognised in Japan: 32 of which were previously known from the Japanese fauna, eight were new to science, seven were new to Japan, and seven were excluded from the Japanese fauna (Table 5). All Japanese species and nomenclatural changes are summarised in Suppl. material 3.

**Table 5.** Summary of taxonomic results for Japanese species of *Enicospilus*. Valid species names are in bold. Total species number was calculated as follow: (a) previous total species numbers (i.e., 39 species; cf. Table 1), minus (b) number of ‘deleted species or names’, plus (c) number of added species (i.e., ‘new species’ plus ‘new records’).

**New species**	**New records**	**Deleted species or names**	**Total species number**
**8**	**7**	**7**	**47** (= 39 - 7 + (8 + 7))
***acutus* sp. nov.**	*** jilinensis ***	***biharensis*** (misidentification)	
***kunigamiensis* sp. nov.**	*** laqueatus ***	***flavicaput*** (misidentification)	
***limnophilus* sp. nov.**	***multidens* stat. rev.**	*iracundus* syn. nov. (= ***sakaguchii***)	
***matsumurai* sp. nov.**	*** puncticulatus ***	***lineolatus***yn. nov. (= ***pungens***)	
***pseudopuncticulatus* sp. nov.**	*** stenophleps ***	***merdarius*** (misidentification)	
***sharkeyi* sp. nov.**	*** vestigator ***	***vacuus*** syn. nov. (= ***formosensis***)	
***takakuwai* sp. nov.**	***zeugos* stat. rev.**	*yamanakai* syn. nov. (= ***shinkanus***)	
***unctus* sp. nov.**			

**Figure 6.**
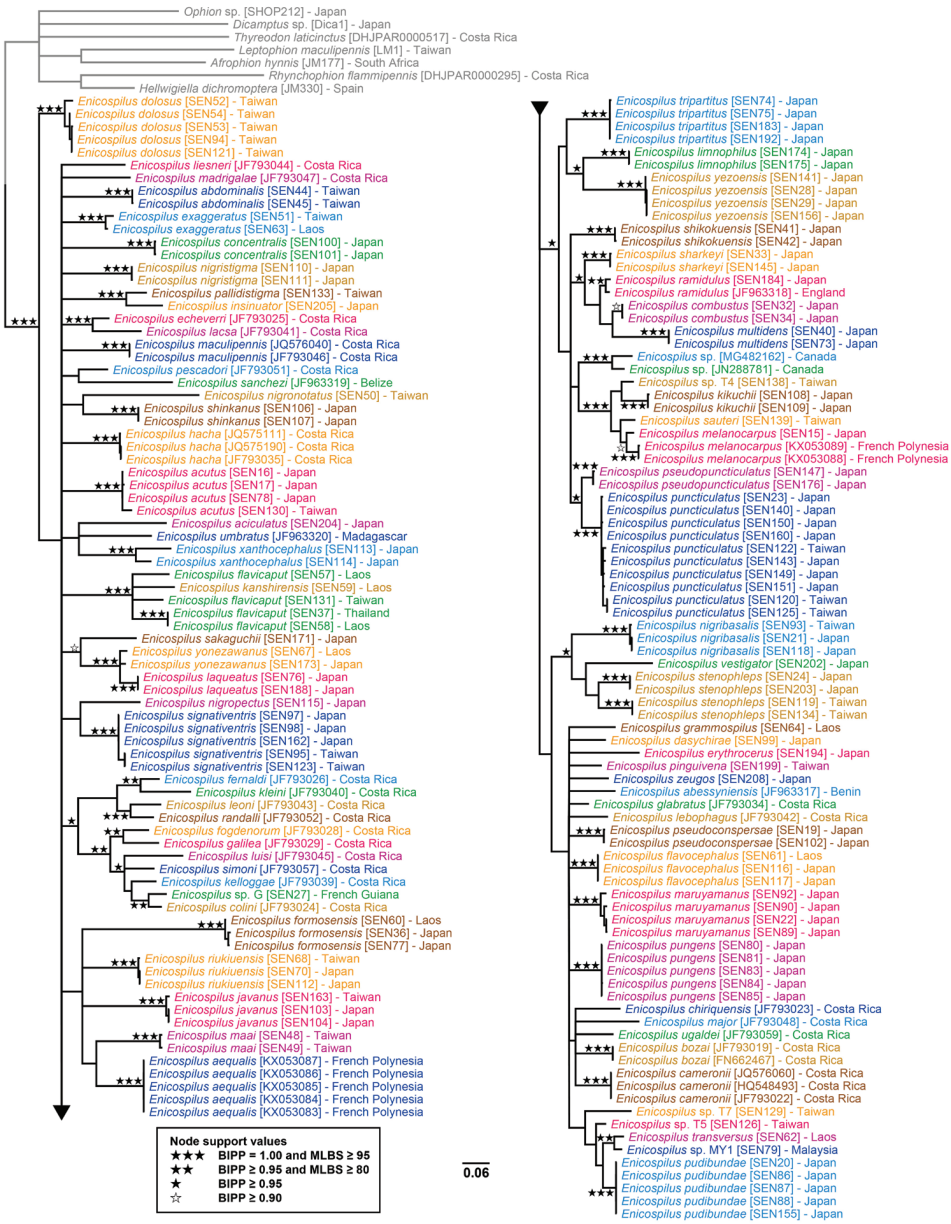
Bayesian majority-rule consensus tree based on a barcoding gene (BIPP = the Bayesian inference posterior probabilities; MLBS = the maximum likelihood bootstrap percentages).

For most Japanese *Enicospilus* species, the morphological and DNA barcoding results were complementary and consistent, and we could easily separate species (Fig. 6). However, the results from each approach were inconsistent for a few species: three Japanese species, *E. xanthocephalus* Cameron, 1905, *E. stenophleps* Cushman, 1937, and *E. puncticulatus* Tang, 1990, and the non-Japanese *E. flavicaput* (Morley, 1912). In our DNA barcoding analysis, maximum p-distances within species were less than 2% in virtually all species, but 6% in *E. stenophleps* and 7% in *E. flavicaput* and *E. xanthocephalus*. These species were morphologically stable, hence, we were following the traditional morphology-based taxonomy and not splitting them in the present paper. On the other hand, although *E. puncticulatus* exhibits a wide range of variation in morphology, especially of the shape of fore wing sclerites, maximum p-distances within this species were less than 1%. In the present paper, we treated them as a single species, because the fore wing variation is likely to be continuous (see the species account for *E. puncticulatus* for more details).

**Figure 7.**
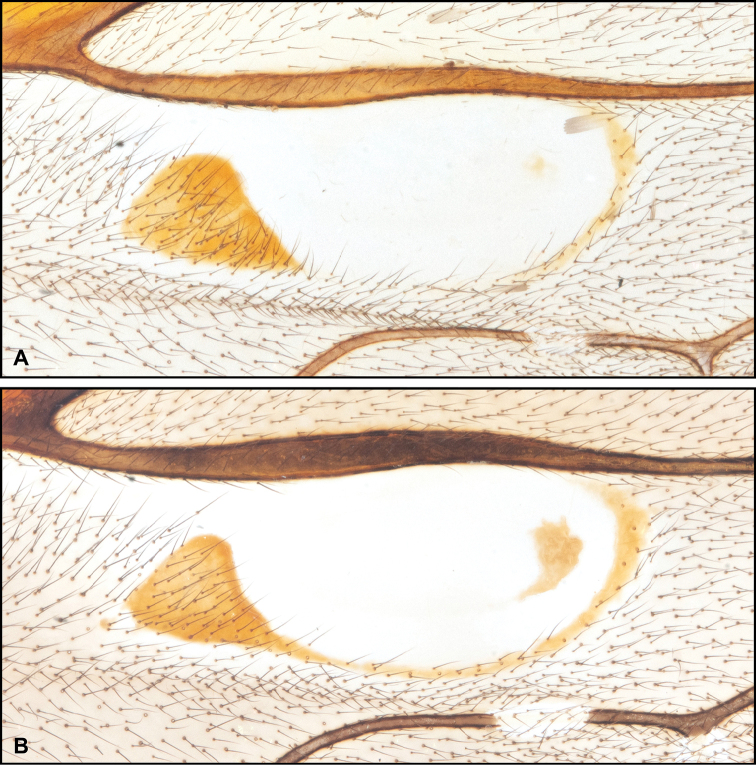
Intraspecific variation of the fore wing sclerite development in *E. shikokuensis* (Uchida, 1928) **A** the proximal and distal sclerites separated and the central sclerite weak (SEN42) **B** the proximal and distal sclerites confluent and the central sclerite strong (SEN41).

**Figure 8.**
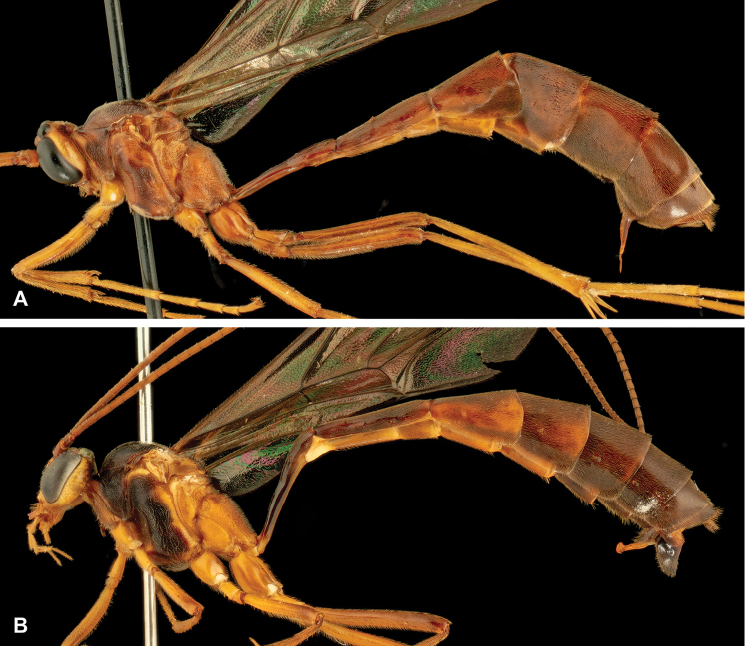
Intraspecific variation of the body colour in *E. shikokuensis* (Uchida, 1928) **A** paler (SEN42) **B** darker (SEN41) individuals.

DNA barcoding analyses sometimes show traditional diagnostic characters to be ineffectual. The confluence or separation of the proximal and distal sclerites of fore wing fenestra has been considered to be an important diagnostic character for *Enicospilus* species: for example, Gauld & Mitchell (1981) used it in rather early couplet of their identification key. However, in *E. shikokuensis* (Uchida, 1928), p-distance between the separated (SEN42, LC492901, from Niigata Prefecture, Fig. 7A) and confluent individuals (SEN41, LC492900, from Hyôgo Prefecture, Fig. 7B) was less than 1%. The body colour of the former is paler (Fig. 8A) and of the latter darker (Fig. 8B). It suggests that the shape of fore wing sclerites are strongly affected by the degree of melanisation. Our morphological results also suggest no other difference between them. Thus, in some specimens it is difficult to decide whether the proximal and distal sclerites are confluent or not. Hence, this character is sometimes not good for diagnosing species. *Enicospilus sigmatoides* Chiu, 1954, syn. nov. has been separated from *E. shikokuensis* only on the basis of the separated proximal and distal sclerites. It is a paler individual of *E. shikokuensis*. Therefore, *E. sigmatoides* syn. nov. is synonymised under *E. shikokuensis*.

### Taxonomic accounts

**Class Insecta Linnaeus, 1758**

**Order Hymenoptera Linnaeus, 1758**

**Superfamily Ichneumonoidea Latreille, 1802**

**Family Ichneumonidae Latreille, 1802**

**Subfamily Ophioninae Shuckard, 1840**

#### Genus Enicospilus

Taxon classificationAnimaliaHymenopteraIchneumonidae

Stephens, 1835

BA26F7DF-CCDE-5E55-A217-5606F27447D8

Enicospilus Stephens, 1835: 126; type species, Ophion merdarius Gravenhorst sensu Stephens (= Ichneumon ramidulus Linnaeus), by monotypy (Stephens 1845).
Henicospilus Agassiz 1846: 138; unjustified emendation.
Allocamptus Förster, 1869: 150; type species, Ophion undulatus Gravenhorst, 1829, by subsequent designation (Thomson 1888: 1189).
Dispilus Kriechbaumer, 1894: 309; type species, Ophion (Dispilus) natalensis Kriechbaumer, 1894, by monotypy.
Pleuroneurophion Ashmead, 1900: 86; type species, Pleuroneurophion hawaiiensis Ashmead, 1900, by original designation.
Banchogastra Ashmead, 1900: 87; type species, Banchogastra niger Ashmead, 1900, by original designation.
Pycnophion Ashmead, 1900: 87; type species, Pycnophion molokaiensis Ashmead, 1900, by original designation.
Cymatoneura Kriechbaumer, 1901a: 22; type species, Ophion undulatus Gravenhorst, 1829, by subsequent designation (Viereck 1914: 8).
Pterospilus Kriechbaumer, 1901b: 156; type species, Ophion (Enicospilus) dubius Tosquinet, 1896, by subsequent designation (Viereck 1914: 126); junior homonym of Pterospilus Rondani, 1856.
Trispilus Kriechbaumer, 1901b: 156; type species, Ophion (Enicospilus) trimaculatus Tosquinet, 1896, by monotypy.
Abanchogastra Perkins, 1902: 141; type species, Abanchogastra debilis Perkins, 1902, by monotypy.
Metophion Szépligeti, 1905: 28; type species, Metophion bicolor Szépligeti, 1905, by subsequent designation (Viereck 1914: 94).
Ceratospilus Szépligeti, 1905: 28; type species, Ceratospilus biroi Szépligeti, 1905, by monotypy.
Atoponeura Szépligeti, 1905: 34; type species, Atoponeura concolor Szépligeti, 1905 (= Enicospilus atoponeurus Cushman, 1947), by monotypy.
Ophiomorpha Szépligeti, 1905: 34; type species, Ophion curvinervis Cameron, 1886 (= Enicospilus cameronii Dalla Torre, 1901), by subsequent designation (Hooker 1912: 134); junior homonym of Ophiomorpha Nilsson, 1836.
Cryptocamptus Brèthes, 1909: 230; unnecessary replacement name for Allocamptus Förster, 1869.
Amesospilus Enderlein, 1914: 222; type species, Ophion unicallosus Vollenhoven, 1878, by original designation.
Eremotyloides Perkins, 1915: 530; type species, Eremotylus orbitalis Ashmead, 1901, by monotypy.
Schizospilus Seyrig, 1935: 79; type species, Schizospilus divisus Seyrig, 1935, by original designation.

##### Distribution.

Afrotropical, Australasian, Holarctic, Neotropical, Oceanic, and Oriental regions (Yu et al. 2016).

##### Bionomics.

According to the available evidence, species of *Enicospilus* are koinobiont endoparasitoids of usually late instar Lepidoptera larvae, but sometimes ovipositing in early instars (see summary in Broad et al. 2018). Many lepidopteran families, such as Noctuidae, Notodontidae and Saturniidae, are recorded as hosts (e.g., Gauld and Mitchell 1978, 1981; Gauld 1988; Broad and Shaw 2016). They are frequently collected at light and usually considered to be nocturnal or crepuscular (e.g., Gauld and Mitchell 1981; Gauld 1988; Shimizu and Maeto 2016; Shimizu 2017). Some *Enicospilus* are parasitoids of economically important Lepidoptera pests (e.g., Nagatomi et al. 1972; Kusigemati 1976). For instance, *Enicospilus signativentris* (Tosquinet, 1903) is a parasitoid of Poaceae pests, such as the noctuid moths *Anadevidia peponis* Fabricius, 1775, *Autographa nigrisigna* Walker, 1857 and *Trichoplusia intermixta* (Warren, 1913) (Kusigemati 1976, 1981; Kusigemati and Tanaka 1992); and *E. sakaguchii* (Matsumura & Uchida, 1926) is also known as a parasitoid of the rice pests *Sesamia turpis* (Butler, 1879) and *S. inferens* (Walker, 1856) (Nagatomi et al. 1972). Hence, Ophioninae could potentially be useful for agriculture as biocontrol agents (e.g., Gauld and Mitchell 1978, 1981; Gauld 1988).

##### Generic diagnosis.

*Enicospilus* species are moderately to very large insects, fore wing length usually 10.0–30.0 mm, with ophionoid facies. Easily distinguishable from other Ophioninae by the following characters: fore wing discosubmarginal cell with extensive glabrous area (fenestra), often with one or more sclerites (e.g., Fig. 4); mandibles narrow, slightly to strongly twisted (e.g., Fig. 2); inner surface of fore tibial spur lacking membranous flange. *Enicospilus* species can be confused with the genus *Dicamptus* but easily distinguished by the weakly to strongly tapered and twisted mandible (mandible very weakly tapered and never strongly twisted in *Dicamptus*). A key to the Japanese genera of Ophioninae has also been provided by Shimizu and Watanabe (2017).

##### Generic description.

Middle- to large-sized wasps (fore wing length usually 10.0–30.0 mm).

Head. Clypeus flat to strongly convex in profile, ventral margin acute, blunt, or impressed. Mandible weakly to strongly tapered and twisted, usually moderately long, outer surface with or without diagonal setose groove or line of punctures, and bidentate apically. Frons, vertex and gena shiny and smooth. Ocelli usually very large and posterior ocellus often close to or touching eye. Occipital carina usually complete, ventrally reaching oral carina or not. Antennae usually longer than fore wing, with usually more than 50 flagellomeres.

Mesosoma entirely weakly to moderately shiny with setae. Pronotum finely punctate or diagonally wrinkled and not specialised. Mesoscutum shiny and punctate to smooth with setae, evenly rounded in profile, and notauli usually absent. Scutellum moderately convex and usually with lateral longitudinal carinae. Epicnemium usually densely punctate with setae. Epicnemial carina present, straight to curved, inclined to curved to anterior margin of mesopleuron. Posterior transverse carina of mesosternum usually complete. Propodeum evenly rounded or declivous in profile; anterior transverse carina usually complete; anterior area longitudinally striate; spiracular area usually smooth; posterior area reticulate, wrinkled, striate, or rugose; and posterior transverse carina usually absent.

Wings. Fore wing pterostigma fairly slender; vein 1m-cu&M evenly curved, angulate or sinuate, usually without a ramulus; vein 2r&RS usually more or less widened and sinuate; discosubmarginal cell usually with bare fenestra, often with one or more sclerotised sclerites. Hind wing vein RS usually straight and rarely weakly curved; vein RA usually with 4–12 uniform hamuli.

Legs. Inner mesal surface of fore tibial spur without membranous flange. Outer distal margin of mid and hind trochantelli usually simple without decurved tooth. Hind tarsal claw moderately to strongly curved and usually simply pectinate.

Metasoma very slender. Spiracle of T1 far behind middle. Thyridium well developed. Ovipositor straight and almost always not longer than posterior depth of metasoma.

Colour. General body colour usually entirely testaceous, with posterior metasomal segments sometimes darker, but body sometimes entirely dark brown to black or pale. Wings usually entirely hyaline or weakly infuscate, but wings with strong infumate area in a few species; fenestra always hyaline; sclerites weakly to strongly pigmented amber.

##### Species criteria.

We summarise the especially important diagnostic characters to identify *Enicospilus* species below.

*Head* (Fig. 2). The head provides many good characters to define species, as many previous authors have indicated (e.g., Gauld and Mitchell 1978, 1981; Gauld 1988; Schwarzfeld and Sperling 2014; Johansson and Cederberg 2019). Among them, the width of lower face as well as of clypeus, colour of interocellar area (or stemmaticum), shape of clypeus, and mandibular characters are especially useful and easy to use.

Width of the lower face is usually stable within a species group and/or species, even if the species is widespread, and sometimes provides enough gaps between species, although a few species, such as *E. capensis*, exhibit considerable variation.

Although body colour can be very variable within species, the colour of the interocellar area is usually stable at the species level and a good diagnostic character.

The shape of the clypeus is also very useful. For instance, the nasute clypeus is one of the most critical diagnostic characters of *E. riukiuensis* and related species (Fig. 40D), and the flat and projecting clypeus of *E. sakaguchii* is distinctive (Fig. 41D). The shape of the ventral margin, i.e., acute, blunt, or impressed, is also a very useful diagnostic character.

Features of mandibles are some of the most important diagnostic characters of *Enicospilus* species. First, the outer mandibular surface sculpture, especially presence or absence of a diagonal setose groove or line of punctures between the dorsoproximal corner and base of the apical teeth, is important. For example, the outer mandible surface of *E. ramidulus* has a diagonal setose groove (Fig. 39B, D), but of *E. pungens* is smooth (Fig. 38B, D). Second, the torsion of the mandible is a useful character, although it is rather difficult to measure. For instance, the strongly twisted mandible of *E. acutus* sp. nov. is one of the most important diagnostic characters for this species (Fig. 11B, D). Finally, length and shape also provide good characters. For instance, the mandible of *E. shikokuensis* is very long, slender, strongly tapered proximally, and subparallel-sided distally (Fig. 44B, D), but of *E. sakaguchii* is very short, stout, and evenly tapered (Fig. 41B, D).

Some mandibular diagnostic characters at the species level, such as degree of torsion and length of teeth are possibly adaptive characters and have been considered to be related to modes of emergence from host insects; hence, these characters are usually easily modified and not phylogenetically restricted, so it is indeed useful for species level taxonomy.

*Mesosoma* (Fig. 3). Mesosomal characters are also very informative. Surface microsculuptures of meso- and metapleuron are rather stable within species and also show large gaps between species and/or species groups. For example, the mesopleuron is coarsely longitudinally striate and metapleuron coarsely rugose in *E. nigristigma* (Fig. 31E), but the meso- and metapleuron are evenly moderately punctate and strongly shiny in *E. unctus* sp. nov. (Fig. 50E). Propodeum characters are useful as well. The posterior transverse carina of the propodeum of *E. signativentris* is unique in the Japanese species (Fig. 46E), and globally few *Enicospilus* species possess this carina. In other cases, the posterior area can be entirely densely punctate to finely reticulate in *E. limnophilus* sp. nov. (Fig. 25E), but coarsely concentrically striate in *E. insinuator* (Fig. 19E), providing an easy means to separate them. In this way, the propodeum is useful for definition of the species or species group when combined with other characters. Characters of the scutellum are also sometimes good for species recognition. For example, the quadrate scutellum of *E. formosensis* contributes to its identification, with the scutellum more or less trapezoidal or triangular in most *Enicospilus* species. Additionally, the length of the lateral longitudinal carinae of the scutellum is almost always stable within species and useful, with very rare exceptions, such as in *E. limnophilus* sp. nov., that exhibits a very wide range of variation (length of the carinae varies from 0.1–1.0× scutellum length).

*Wings* (Fig. 4). Wing characters have probably been regarded as the most important diagnostic characters in nocturnal Ophioninae by many previous authors (e.g., Gauld 1977; Gauld and Mitchell 1981; Shimizu 2020). These are much easier to use than other characters and can easily be measured.

First, the number, shape, and position of sclerites of the fore wing fenestra is usually very useful in *Enicospilus*. The number of sclerites varies in *Enicospilus* species from zero to four, but is nearly always stable within a species. The shape and position of sclerites are also very diverse, and some previous research has suggested that some species exhibit a wide range of intraspecific variation (e.g., Gauld and Mitchell 1981). However, it is stable in many cases and, if a species shows a wide range of intraspecific variation, it is likely that cryptic species are involved.

Second, the shape and setosity of the fore wing fenestra is sometimes a very useful character. For example, among Japanese species, the fenestra of *E. nigribasalis* and *E. stenophleps* is long and its anterodistal corner interstitial to fore wing vein RS (Figs 30F, 47F), but in other Japanese species the fenestra is shorter and its anterodistal corner clearly antefurcal to RS (e.g., Figs 25F, 36F).

Finally, the length and shape of wing veins also offer very good diagnostic characters. For example, the shape of fore wing veins 1m-cu&M and 2r&RS, the position of fore wing vein 1cu-a, and values of indices (e.g., AI, CI, ICI) are useful to distinguish species.

*Legs*. Legs do not seem to provide many useful characters, but some characters, such as the density of spines on the outer surface of the fore tibia, and pectination of the hind tarsal claw, are useful in species definition. For example, *E. maruyamanus* and *E. pudibundae* are very difficult to distinguish from each other, but the hind tarsal claw of the former is entirely uniformly pectinate, and the latter is not pectinate proximally.

*Metasoma* (Fig. 5). The shape of the first metasomal segment (e.g., sinuous or straight in profile, slender or stout) is useful (Broad and Shaw 2016; Johansson and Cederberg 2019).

*Body size*. Measuring body length is rather difficult due to the wide range of contraction or expansion of metasomal segments after death, hence fore wing length is a more useful character. However, body size shows a very wide range of variation in many species, such as *E. pseudoconspersae*, although it is stable in a few species (e.g., *E. concentralis* and *E. nigronotatus*). Therefore, this character is occasionally useful for species definition.

*Colour*. As mentioned above, this character can easily change intraspecifically. For instance, *E. nigropectus* and *E. signativentris* show a considerable range of colour variation (body entirely blackish to entirely testaceous). However, this character is stable within some species (e.g., *E. acutus* sp. nov. and *E. nigribasalis*, as in Figs 11, 30). Hence, this is also a useful critical character, but we need to be careful when relying on colour pattern.

### Key to the Japanese species of *Enicospilus*

**Table:** 

1	Fore wing fenestra lacking both sclerites and quadra (Fig. 16F)	***E. erythrocerus* (Cameron, 1905)**
–	Fore wing fenestra with more or less distinct sclerites (e.g., Figs 9F, 15F, 20F, 26F, 33F) and sometimes with quadra (e.g., Fig. 31F)	**2**
2 (1)	Fore wing fenestra without proximal and central sclerites and only with rather strongly pigmented and thick distal sclerite (Fig. 38F)	***E. pungens* (Smith, 1874)**
–	Fore wing fenestra with weakly to strongly pigmented proximal sclerite and also sometimes with central and/or distal sclerite (if distal sclerite present, it is never thickened) (e.g., Figs 34F, 35F)	**3**
3 (2)	Mandible very strongly twisted by ca. 85°, therefore outer margin forming acute median longitudinal ridge between centroproximal part of mandible and base of apical teeth (this ridge is the ventral margin of the mandible) (Fig. 11B, D). Posterior part of mesoscutum infuscate. AI = 1.0–1.4 (Fig. 11F)	***E. acutus* Shimizu, sp. nov.**
–	Mandibular torsion various, not very strongly twisted and outer margin never forming acute median longitudinal ridge as above. Colour of mesoscutum various. AI various	**4**
4 (3)	Clypeus nasute, strongly convex, anterior margin acute and strongly projecting, and ventral margin strongly and abruptly impressed (Figs 40B, D)	***E. riukiuensis* (Matsumura & Uchida, 1926)**
–	Clypeus not nasute, flat to moderately convex, anterior margin obtuse and rounded if convex, and ventral margin impressed to acute (e.g., Figs 9D, 10D)	**5**
5 (4)	Proximal margin of proximal sclerite of fore wing fenestra distinctly separated from proximal margin of fenestra by more than width of proximal sclerite (e.g., Figs 14F, 15F, 17F, 20F, 26F)	**6**
–	Proximal margin of proximal sclerite of fore wing fenestra joining or close to proximal margin of fenestra, if separated then by less than half width of proximal sclerite (e.g., Figs 9F, 19F, 28F, 30F)	**15**
6 (5)	Interocellar area entirely black (Fig. 52C)	***E. xanthocephalus* Cameron, 1905**
–	Interocellar area entirely yellow- to red-brown (e.g., Fig. 55C)	**7**
7 (6)	Proximal sclerite of fore wing fenestra narrow and more or less linear (e.g., Fig. 26F)	**8**
–	Proximal sclerite of fore wing fenestra various but rather broad (triangular, circular, comma-shaped, etc.) (e.g., Figs 15F, 17F, 20F, 34F)	**10**
8 (7)	Fore wing fenestra with linear central sclerite (Fig. 55F)	***E. zeugos* Chiu, 1954, stat. rev.**
–	Fore wing fenestra without central sclerite (Figs 26F, 36F)	**9**
9 (8)	Fore wing vein 1m-cu&M evenly curved (Fig. 36F). Proximal pectin of hind tarsal claw absent. Mesopleuron and metapleuron closely punctate (Fig. 36E)	***E. pudibundae* (Uchida, 1928)**
–	Fore wing vein 1m-cu&M weakly to moderately sinuous (Fig. 26F). Hind tarsal claw uniformly pectinate. Mesopleuron and metapleuron closely punctostriate (Fig. 26E)	***E. maruyamanus* (Uchida, 1928)**
10 (7)	Fore wing with both CI and ICI more than 0.7 (Fig. 32F). Large insects (fore wing length more than 20 mm)	***E. nigronotatus* Cameron, 1903**
–	Fore wing with either or both CI and ICI less than 0.65 (Figs 14F, 15F, 17F, 20F, 34F). Small to moderate sized insects (fore wing length less than 18 mm)	**11**
11 (10)	Proximal part of marginal cell of fore wing widely glabrous (Fig. 14F). Fore wing infumate, particularly strongly infumate in marginal cell adjacent to glabrous area (Fig. 14F). Fore wing with ICI = 0.2–0.3 (Fig. 14F)	***E. concentralis* Cushman, 1937**
–	Marginal cell of fore wing uniformly setose (Figs 15F, 17F, 20F, 34F). Fore wing evenly weakly infumate or hyaline (Figs 15F, 17F, 20F, 34F). Fore wing with ICI = 0.4–0.7 (Figs 15F, 17F, 20F, 34F)	**12**
12 (11)	Fore wing fenestra with central sclerite (Fig. 17F). Fore wing vein 1m-cu&M strongly angulate and widened centrally (Fig. 17F)	***E. flavocephalus* (Kirby, 1900)**
–	Fore wing fenestra without central sclerite (Figs 15F, 20F, 34F). Fore wing vein 1m-cu&M evenly curved or sinuous and not widened (Figs 15F, 20F, 34F)	**13**
13 (12)	Proximal and distal sclerites of fore wing fenestra strongly confluent and distal sclerite strongly sclerotised (Fig. 20F). Confluent proximal and distal sclerites of fore wing fenestra shaped like a letter ‘P’, as in Fig. 20F	***E. javanus* (Szépligeti, 1910)**
–	Proximal sclerite of fore wing fenestra isolated and distal sclerite absent or vestigial (Figs 15F, 34F). Proximal sclerite of fore wing fenestra half-moon or drop-shaped, as in Figs 15F, 34F	**14**
14 (13)	Proximal sclerite of fore wing fenestra usually entirely weakly pigmented and half moon-shaped (Fig. 34F)	***E. pseudoconspersae* (Sonan, 1927)**
–	Proximal sclerite of fore wing fenestra partly to entirely strongly pigmented and drop-shaped (Fig. 15F).	***E. dasychirae* Cameron, 1905**
15 (5)	Outer mandibular surface usually with strong diagonal groove between dorsoproximal corner and medio- to ventrobasal part of apical teeth; groove usually bearing moderate to very dense setae (e.g., Figs 2C, 10B, D, 50B, D, 53B, D). In rare cases groove indistinct or absent, but always with diagonal visible line of dense punctures with setae or outer surface bearing very dense punctures with stout setae (e.g., Fig. 49B, D)	**16**
–	Outer mandibular surface smooth; sometimes with punctures or setae but never forming diagonal line and never dense (e.g., Figs 18B, D, 27B)	**37**
16 (15)	Clypeus flat, ventral margin acute and projecting (Fig. 41D). Mandible short and stout (Fig. 41B, D). Fore wing fenestra usually without central sclerite (rarely with vestigial small central sclerite) (Fig. 41F)	***E. sakaguchii* (Matsumura & Uchida, 1926)**
–	Clypeus flat to convex, and ventral margin acute to impressed but never projecting (e.g., Figs 44D, 50D, 54D). Mandible various, however moderately to very long and slender (e.g., Figs 28B, D, 44B, D, 53B, D). Fore wing fenestra with central sclerite (e.g., Figs 10F, 28F), except for *E. yonezawanus* (Fig. 54F)	**17**
17 (16)	Fore wing fenestra without central sclerite (Fig. 54F). Clypeus convex and ventral margin strongly impressed (Fig. 54D). Both meso- and metapleuron closely striate (Fig. 54E)	***E. yonezawanus* (Uchida, 1928)**
–	Fore wing fenestra with central sclerite (e.g., Figs 42F, 44F, 53F). Clypeus moderately convex to flat, ventral margin impressed to acute (e.g., Figs 10D, 28D). Meso- and metapleuron usually punctate or punctostriate (e.g., Figs 10E, 13E, 28E, 39E)	**18**
18 (17)	Proximal part of marginal cell of fore wing adjacent to vein 2r&RS with distinct and wide glabrous area (Fig. 42F). Usually terminal segments of metasoma infuscate (Fig. 42A)	***E. sauteri* (Enderlein, 1921)**
–	Marginal cell of fore wing uniformly setose, never with wide glabrous area (e.g., Figs 22F, 43F, 44F). Metasomal colour various (e.g., Figs 10A, 22A, 28A)	**19**
19 (18)	Face broad; lower face more than 0.82× as wide as high; clypeus more than 1.8× as wide as high (e.g., Figs 12B, 13B, 50B, 53B)	**20**
–	Face moderately broad; lower face less than 0.81× as wide as high; clypeus less than 1.8× as wide as high (e.g., Figs 10B, 24B, 28B)	**27**
20 (19)	Meso- and metapleuron entirely very densely punctate so that matt or submatt, punctures of metapleuron contiguous or separated by less than diameter of puncture, thus very weakly shiny or not (Fig. 12E). Central sclerite of fore wing fenestra well-delimited, oval (Fig. 12F)	***E. capensis* (Thunberg, 1824)**
–	Meso- and metapleuron finely to moderately punctate to punctostriate, strongly shiny, and never matt (e.g., Figs 13E, 39E, 44E). Central sclerite of fore wing fenestra various, sometimes poorly delimited with small, weakly pigmentated quadra (e.g., Figs 29F, 39F)	**21**
21 (20)	Posterior area of propodeum entirely punctate and strongly shiny. Meso- and metapleuron entirely punctate and strongly shiny (Fig. 50E)	***E. unctus* Shimizu, sp. nov.**
–	Posterior area of propodeum with fine to coarse sculpture (e.g., reticulation, rugae, striae), never entirely punctate, and usually weakly shiny. Meso- and metapleuron punctate to striate (e.g., Figs 39E, 44E)	**22**
22 (21)	Posterior ocellus distinctly separated from eye (Fig. 53B, C). Lower face always subquadrate (Fig. 53B). Central sclerite of fore wing fenestra comma-shaped, major axis parallel to distal margin of fenestra (Fig. 53F). Malar space 0.4–0.5× as long as basal mandibular width (Fig. 53B, D). Outer mandibular surface with significantly long dense setae (Fig. 53B, D). Posterior segments of metasoma always black (Fig. 53A)	***E. yezoensis* (Uchida, 1928)**
–	Posterior ocellus close to eye but not touching (e.g., Figs 13C, 29C, 39C, 44C). Lower face usually more or less elongate (Figs 13B, 29B, 39B), except very wide in *E. shikokuensis* (Fig. 44B). Central sclerite of fore wing fenestra D-shaped or oval, sometimes poorly delimited or with quadra (e.g., Figs 13F, 29F, 39F, 44F). Malar space less than 0.4× (almost always less than 0.3×) as long as basal mandibular width (e.g., Figs 13B, D, 29B, D, 39B, D, 44B, D). Outer mandibular surface with rather short and sparse setae (e.g., Figs 13B, D, 29B, D, 39B, D, 44B, D). Colour of metasoma various	**23**
23 (22)	Mandible very long and slender; apical 0.7 parallel-sided; proximal outer surface with very wide subtriangular concavity (Fig. 44B, D). Proximal and distal sclerites of fore wing fenestra usually confluent and rarely slightly separated (Fig. 44F). Fore wing vein 1m-cu&M weakly to strongly sinuous (Fig. 44F)	***E. shikokuensis* (Uchida, 1928)**
–	Mandible moderately long; apical 0.3–0.5 (sub-)parallel-sided; proximal outer surface usually with shallow and narrow crescent-shaped concavity (Figs 13B, D, 22B, D, 29B, D, 39B, D). Proximal and distal sclerites of fore wing fenestra always separated (Figs 13F, 29F, 39F) , except confluent in *E. kikuchii* (Fig. 22F). Fore wing vein 1m-cu&M moderately sinuous or evenly curved (Figs 13F, 22F, 29F, 39F)	**24**
24 (23)	Mesosoma and terminal segments of metasoma blackish (Figs 13A, 22A). Central sclerite of fore wing fenestra strongly sclerotised and well-delimited (Figs 13F, 22F)	**25**
–	Body entirely orange brown, except for terminal segments of metasoma sometimes infuscate or black (Figs 29A, 39A). Central sclerite of fore wing fenestra moderately sclerotised and sometimes rather ill-delimited (Figs 29F, 39A)	**26**
25 (24)	Proximal and distal sclerites of fore wing fenestra not confluent (Fig. 13F). Central sclerite of fore wing fenestra well-delimited D-shape (Fig. 13F)	***E. combustus* (Gravenhorst, 1829)**
–	Proximal and distal sclerites of fore wing fenestra confluent (Fig. 22F). Central sclerite of fore wing fenestra rounded (Fig. 22F)	***E. kikuchii* Shimizu, 2017** (in part)
26 (24)	T6–8 black (metasoma entirely testaceous in very rare cases) (Fig. 39A). Central sclerite of fore wing fenestra rather small and oval to suboval, and positioned in centrodistal part of fenestra (Fig. 39F)	***E. ramidulus* (Linnaeus, 1758)**
–	Metasoma always uniformly orange-brown, with posterior segments never black (Fig. 29A). Central sclerite of fore wing fenestra rather ill-defined D-shape, oval, or linear strongly sclerotised area, and positioned in anterodistal corner of fenestra (Fig. 29F)	***E. multidens* Chiu, 1954, stat. rev.**
27 (19)	Central sclerite of fore wing fenestra rather large, but ill-defined and never strongly sclerotised (Fig. 10F). Proximal sclerite of fore wing fenestra rather thin, proximal corner sharp and ca. 45° in Japanese individuals (Fig. 10F). Upper tooth of mandible more than 2.0× as long as lower (Fig. 10B, D)	***E. aciculatus* (Taschenberg, 1875)**
–	Central sclerite of fore wing fenestra variously sized, well defined, and strongly sclerotised (e.g., Figs 22F, 43F). Proximal sclerite of fore wing fenestra usually rather thick, proximal corner sharp to blunt and usually more than 55° (e.g., Figs 28F, 43F). Upper tooth of mandible usually less than 1.7× as long as lower (e.g., Figs 28B, 43B), except for 1.8–2.1 in *E. pseudopuncticulatus* Shimizu, sp. nov. (Fig. 35B)	**28**
28 (27)	Proximal and distal sclerites of fore wing fenestra confluent (e.g., Figs 22F, 28F, 43F)	**29**
–	Proximal and distal sclerites of fore wing fenestra not confluent (e.g., Figs 24F, 25F, 49F)	**32**
29 (28)	Body entirely yellow-brown (Fig. 37A), but posterior segments of metasoma sometimes infuscate (Fig. 28A)	**30**
–	Mesosoma and posterior segments of metasoma dark brown to black (Figs 22A, 43A)	**31**
30 (29)	Metasoma entirely yellow-brown with posterior segments always black (Fig. 28A). Restricted to Ryûkyûs and Ogasawara Islands in Japan	***E. melanocarpus* Cameron, 1905**
–	Metasoma uniformly yellow-brown and posterior segments never infuscate (Fig. 37A). Widely distributed in Japan	***E. puncticulatus* Tang, 1990** (in part)
31 (29)	Coxae, T1–2 and T5–8 dark-brown to black (Fig. 22A, E). Central sclerite of fore wing fenestra larger and more than 1.6× as wide as maximum thickness of 2r&RS (Fig. 22F). Metapleuron rather coarsely diagonally punctostrigose (Fig. 22E)	***E. kikuchii* Shimizu, 2017** (in part)
–	Coxae and T1–2 testaceous, never coloured (Fig. 43A, E). Central sclerite of fore wing fenestra smaller and less than 1.0× as wide as maximum thickness of 2r&RS (Fig. 43F). Metapleuron densely punctate (Fig. 43E)	***E. sharkeyi* Shimizu, sp. nov.**
32 (28)	Distal part of fore wing fenestra between central and distal sclerites covered with setae (Fig. 25F)	***E. limnophilus* Shimizu, sp. nov.**
–	Fore wing fenestra without any setae (e.g., Figs 24F, 48F)	**33**
33 (32)	Outer mandibular surface with deep basal concavity and dense punctures and stout setae, without diagonal groove but punctures and setae sometimes forming diagonal line (Fig. 49B, D)	***E. tripartitus* Chiu, 1954**
–	Outer mandibular surface with shallow basal concavity, with conspicuous diagonal deep setose groove (e.g., Figs 24B, D, 35B, D, 37B, D)	**34**
34 (33)	Central sclerite of fore wing fenestra well delimited, D-shaped (Figs 24F, 48F)	**35**
–	Central sclerite of fore wing fenestra poorly delimited, oval to linear (Figs 35F, 37F)	**36**
35 (34)	Central sclerite of fore wing fenestra positioned in anterodistal part of fenestra, smaller, moderately sclerotised (Fig. 48F)	***E. takakuwai* Shimizu, sp. nov.**
–	Central sclerite of fore wing fenestra positioned in centrodistal part of fenestra, larger, strongly sclerotised (Fig. 24F)	***E. laqueatus* (Enderlein, 1921)**
36 (34)	Fore wing vein 1cu-a more or less curved or angulate (Fig. 37F). Central sclerite of fore wing fenestra oval with quadra proximally (Fig. 37F)	***E. puncticulatus* Tang, 1990** (in part)
–	Fore wing vein 1cu-a straight (Fig. 35F). Central sclerite of fore wing fenestra linear and parallel with 2r&RS (Fig. 35F)	***E. pseudopuncticulatus* Shimizu, sp. nov.**
37 (15)	Interocellar area entirely black (Figs 31B, C, 33C)	**38**
–	Interocellar area entirely yellow- to red-brown (e.g., Figs 19C, 45B, C, 46B, C)	**39**
38 (37)	Fore wing fenestra without central sclerite or quadra (Fig. 33F). Proximal sclerite of fore wing fenestra bullet-shaped (Fig. 33F). Medium-sized insects (fore wing length usually less than 16.5 mm)	***E. nigropectus* Cameron, 1905** (most part)
–	Fore wing fenestra widely covered with very weakly pigmented quadra (Fig. 31F). Proximal sclerite of fore wing fenestra subquadrate (Fig. 31F). Large insects (fore wing length more than 20.0 mm)	***E. nigristigma* Cushman, 1937**
39 (37)	Fore wing fenestra usually without central sclerite (Figs 21F, 33F, 45F), exceptionally *E. insinuator* with large unpigmented quadra (Fig. 19F)	**40**
–	Fore wing fenestra with conspicuously pigmented central sclerite (e.g., Figs 9F, 27F, 46F)	**43**
40 (39)	Fore wing fenestra widely covered with unpigmented to very weakly pigmented quadra (Fig. 19F). Propodeum very coarsely concentrically striate (Fig. 19E)	***E. insinuator* (Smith, 1860)**
–	Fore wing fenestra without quadra (Figs 21F, 33F, 45F). Propodeum finely to coarsely reticulate (Figs 21F, 33E, 45E)	**41**
41 (40)	Fore wing fenestra with bullet-shaped proximal sclerite (Fig. 33F). At least ventral half of mesopleuron longitudinally strigose or striate (Fig. 33E). Metapleuron coarsely reticulate or diagonally strigose (Fig. 33E). Clypeus convex, and ventral margin acute to impressed, never flat and projecting above mandibles (Fig. 33B, D)	***E. nigropectus* Cameron, 1905** (in part)
–	Fore wing fenestra with triangular proximal sclerite (Figs 21F, 45F). Meso- and metapleuron evenly punctate or punctostriate (Figs 21E, 45E). Clypeus flat, sometimes weakly to strongly projecting above mandibles, ventral margin acute or weakly blunt (Figs 21D, 45D)	**42**
42 (41)	Fore wing fenestra wider and anterodistal corner antefurcal to RS by less than length of 2rs-m (Fig. 45F). Propodeum finely reticulate, sometimes with median longitudinal carina along more than anterior half of posterior area (Fig. 45E)	***E. shinkanus* (Uchida, 1928)**
–	Fore wing fenestra smaller and anterodistal corner antefurcal to RS by more than length of 2rs-m (Fig. 21F). Propodeum concentrically striate, without median longitudinal carina (Fig. 21E)	***E. jilinensis* Tang, 1990**
43 (39)	Fore wing fenestra long and anterodistal corner (sub)interstitial or antefurcal to RS by less than 0.3× length of 2rs-m (Figs 30F, 47F)	**44**
–	Fore wing fenestra moderately long and anterodistal corner antefurcal to RS by more than 0.4× length of 2rs-m (e.g., Figs 9F, 18F, 23F)	**45**
44 (43)	Mesoscutum, ventral part of T3–4, and entire of T5–8 infumate to black, otherwise pale brown (Fig. 30A, E). Central sclerite of fore wing fenestra moderately large and semi-linear (Fig. 30F). Wings entirely more or less infumate and proximal part of fore wing especially strongly infumate (Fig. 30A)	***E. nigribasalis* (Uchida, 1928)**
–	Mesoscutum and metasoma entirely orange-brown (Fig. 47A, E). Central sclerite of fore wing fenestra very small and circular (Fig. 47F). Wing uniformly hyaline (Fig. 47F)	***E. stenophleps* Cushman, 1937**
45 (43)	Central sclerite of fore wing fenestra linear and parallel with distal margin of fenestra (Fig. 18F). Discosubmarginal cell of fore wing with conspicuous long line of setae (Fig. 18F)	***E. formosensis* (Uchida, 1928)**
–	Central sclerite of fore wing fenestra various (Fig. 51F), if linear, always parallel with 2r&RS (Figs 23F, 27F). Discosubmarginal cell of fore wing without conspicuous line of setae (Figs 23F, 27F, 51F)	**46**
46 (45)	Propodeum with distinct posterior transverse carina arising from pleural carina (Fig. 46E)	***E. signativentris* (Tosquinet, 1903)**
–	Propodeum without posterior transverse carinae (e.g., Figs 9E)	**47**
47 (46)	Metasoma with striking colour pattern, i.e., anterior parts of T1–4 yellow-brown and posterior parts dark brown to black (Fig. 9A). Hind wing distally infuscate	.***E. abdominalis* (Szépligeti, 1906)**
–	Metasoma entirely orange-brown or brown (e.g., Figs 23A, 27A). Hind wing entirely hyaline	**48**
48 (47)	Fore wing fenestra with larger circular to D-shaped, very strongly sclerotised central sclerite (Fig. 51F)	***E. vestigator* (Smith, 1858)**
–	Fore wing fenestra with smaller linear to elongate suboval, weakly to strongly sclerotised central sclerite (Figs 23F, 27F)	**49**
49 (48)	Fore wing with central sclerite strongly sclerotised and pigmented; setae and veins darker brown (Fig. 23F). Moderately sized wasps (fore wing length ca. 16.0 mm)	***E. kunigamiensis* Shimizu, sp. nov.**
–	Fore wing with central sclerite weakly to moderately sclerotised and pigmented; setae and veins brighter brown (Fig. 27F). Large wasps (fore wing length 19.5–21.5 mm)	***E. matsumurai* Shimizu, sp. nov.**

#### Enicospilus abdominalis

Taxon classificationAnimaliaHymenopteraIchneumonidae

(Szépligeti, 1906)

D8DE9CD8-9289-5E4B-A5B9-90F14D8D9018

Figure 9

Henicospilus abdominalis Szépligeti, 1906: 138; HT ♀ from Sri Lanka, TM, not examined.
Ophion semiopacus Matsumura, 1912: 114; HT ♀ from Taiwan, SEHU, examined; synonymised by Gauld and Mitchell (1981: 429).

##### Specimens examined.

Total of 19 specimens (all ♀♀): Japan (1♀), Nepal (1♀), Sri Lanka (2♀♀), Taiwan (15♀♀).

Type series: HT ♀ of *Ophion semiopacus* Matsumura, 1912, Gyochi, TAIWAN, Matsumura leg. (SEHU).

##### Distribution.

Australasian, Eastern Palaearctic, and Oriental regions (Yu et al. 2016); this is a predominantly Oriental species.

Newly recorded from Nepal.

JAPAN: [Ryûkyûs] Okinawa (Shimizu and Maeto 2016; present study). This species is abundant in Taiwan and in other mountainous areas of the Oriental region, but only one Japanese specimen has been collected from Okinawa-hontô of the Ryûkyûs. This single Japanese individual could have been a wanderer from Taiwan or other southern areas.

##### Bionomics.

Unknown.

##### Differential diagnosis.

**Figure 9.**
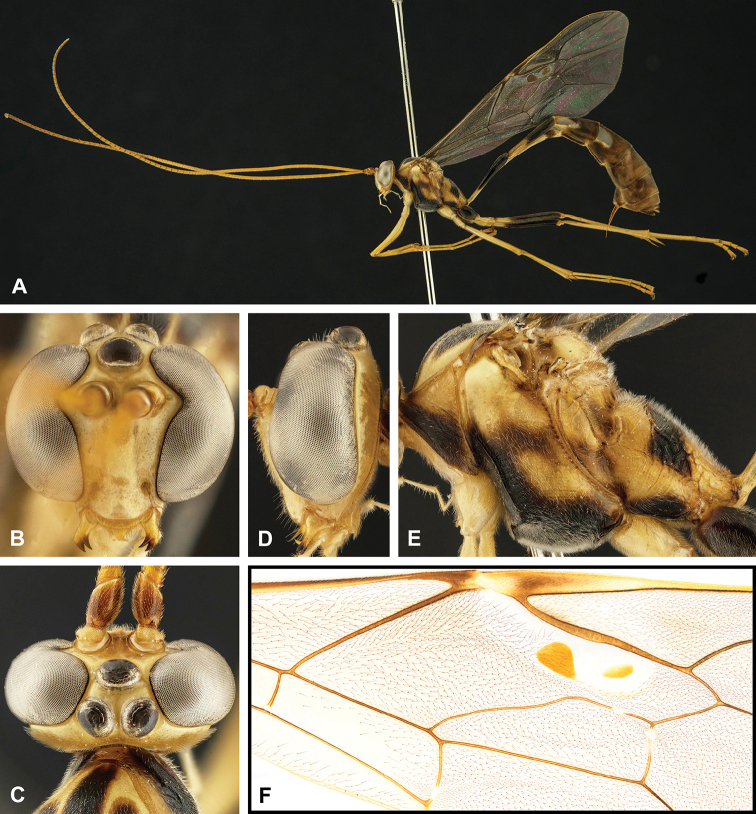
*Enicospilus abdominalis* (Szépligeti, 1906) ♀ from Japan **A** habitus **B** head, frontal view **C** head, dorsal view **D** head, lateral view **E** mesosoma, lateral view **F** central part of fore wing.

The characteristic striking colour pattern of this species (i.e., T1–4 each anteriorly yellow-brown and posteriorly dark brown, as in Fig. 9A) is the most useful diagnostic character among the Japanese species of *Enicospilus*. Some non-Japanese species, such as *E. zebrus* Gauld & Mitchell, 1981, have a similar colour pattern, but *E. abdominalis* is distinguishable by many characters, such as shape and size of fore wing fenestra and sclerites.

This species has been confused with *E. nigropectus* by many authors (cf. Gauld and Mitchell (1981)) but is easily distinguished from *E. nigropectus* by presence of central sclerite of fore wing fenestra, as in Fig. 9F (central sclerite absent in *E. nigropectus*, as in Fig. 33F), larger value of SDI (1.3–1.4 in this species, as in Fig. 9F, but 0.9–1.1 in *E. nigropectus*, as in Fig. 33F), yellowish interocellar area (Fig. 9B, C) (interocellar area usually blackish in *E. nigropectus*, as in Fig. 33B, C), etc. *Enicospilus abdominalis* is also morphologically similar to *E. signativentris* but can be distinguished by striking colour pattern and absence of distinct posterior transverse carina of propodeum.

#### Enicospilus aciculatus

Taxon classificationAnimaliaHymenopteraIchneumonidae

(Taschenberg, 1875)

B08ADC10-6355-51BD-90A3-FDAB909B387C

Figure 10

Ophion aciculatus Taschenberg, 1875: 434; LCT ♀ from Java, designated by Townes et al. (1961: 269), FZLU, not examined.
Enicospilus malaitensis Brues, 1918: 117; HT ♂ from Solomon Island, MCZ, not examined; synonymised by Gauld and Mitchell (1981: 394).
Henicospilus okinawensis Matsumura and Uchida, 1926: 71; LCT ♀ from Ryûkyûs, designated by Townes et al. (1961: 285), SEHU, examined; synonymised by Gauld and Mitchell (1981: 394).
Henicospilus neddenveli Cheesman, 1936: 185; HT ♂ from Vanuatu, NHMUK, examined; synonymised by Gauld and Mitchell (1981: 394).
Enicospilus crucis Chiu, 1954: 70; HT ♀ from Taiwan, TARI, examined; synonymised by Gauld and Mitchell (1981: 394).

##### Specimens examined.

Total of 54 specimens (29♀♀24♂♂ and 1 unsexed): India (17♀♀5♂♂), Japan (8♀♀17♂♂ and 1 unsexed), Solomon Islands (2♀♀), Sri Lanka (1♀1♂), Taiwan (1♀), Vanuatu (1♂).

Type series: LCT ♀ of *Henicospilus okinawensis* Matsumura & Uchida, 1926, Okinawa, Ryûkyûs, JAPAN, 1925, S. Sakaguchi leg. (SEHU); HT ♀ of *Enicospilus crucis* Chiu, 1954, Jûjiro, TAIWAN, 26.IV.1931, T. Shiraki leg. (TARI); HT ♂ of *Henicospilus neddenveli* Cheesman, 1936, Malekula, New Hebrides [VANUATU], III.1930, L.E. Cheesman leg. (NHMUK, Type 3b.1242).

##### Distribution.

Australasian, Eastern Palaearctic, Oceanic and Oriental regions (Yu et al. 2016).

JAPAN: [Kyûshû] Fukuoka* and Kagoshima (Momoi 1970; present study); [Ryûkyûs] Kagoshima (Uchida 1956; Momoi 1970; present study) and Okinawa (Matsumura and Uchida 1926; Uchida 1928; present study). *New record. *Enicospilus aciculatus* is a predominantly (sub-)tropical species and rare in the cooler zone.

##### Bionomics.

Although this is one of the most common species in the Oriental region, there are no host records.

##### Differential diagnosis.

**Figure 10.**
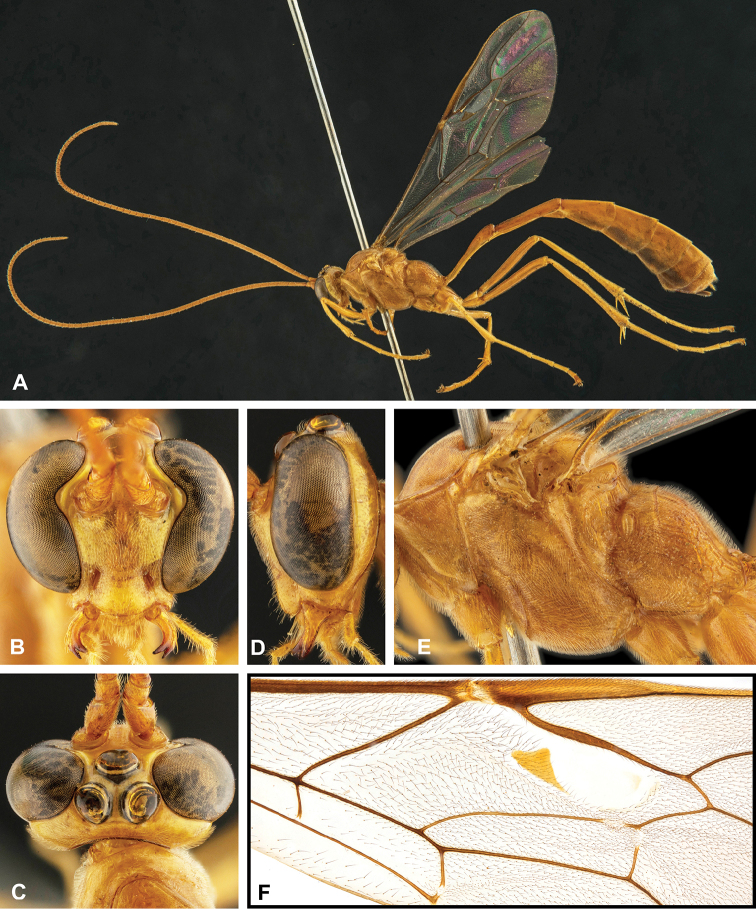
*Enicospilus aciculatus* (Taschenberg, 1875) ♀ from Japan **A** habitus **B** head, frontal view **C** head, dorsal view **D** head, lateral view **E** mesosoma, lateral view **F** central part of fore wing.

This species is morphologically relatively close to *E. laqueatus* and *E. yonezawanus*. However, *E. aciculatus* is rather easily distinguished from all other species of Japanese *Enicospilus* by the following combination of character states: mandible with diagonal setose groove, and upper mandibular tooth more than 2.0× as long as lower (Fig. 10B, D); central sclerite of fore wing fenestra moderately sized, ill-defined, very weakly pigmented, and positioned in anterodistal corner of fenestra (Fig. 10F); and proximal angle of proximal sclerite of fore wing fenestra ca. 45° (Fig. 10F). This species is morphologically stable but sometimes exhibits colour variation (i.e., usually the metasoma is entirely orange brown, but rarely the posterior segments are black).

#### Enicospilus acutus

Taxon classificationAnimaliaHymenopteraIchneumonidae

Shimizu sp. nov.

C4D36F34-A0DD-539A-ABB7-494D907419AE

http://zoobank.org/F6D9DDEC-2D45-48D3-8E4D-E00C67CAA1A7

Figure 11

##### Etymology.

The specific name is derived from the characteristic longitudinal acute ridge of the mandibular outer margin.

##### Type series.

Total of 87 specimens (66♀♀20♂♂ and 1 unsexed): Japan (65♀♀20♂♂ and 1 unsexed), Taiwan (1♀).

HT: ♀, Ōkuni-rindô (26°43'38.7"N, 128°12'34.4"E, 265 m alt.), Okuma, Kunigami Vil., Kunigami County, Okinawa-hontô, Okinawa Pref., Ryûkyûs, JAPAN, 30.VI–1.VII.2016, S. Shimizu et al. leg. (LT) (MNHA).

PT: 1♀, Kisokoma-kôgen, Kiso County, Nagano Pref., Kantô-Kôshin, JAPAN, 10.VII.1982, Y. Yoshida & S. Yoshimatsu leg. (LT) (NIAES); 4♀♀ and 1 unsexed, Kanaya, Shizuoka Pref., Tôkai, JAPAN, 14.VII.1947 (1♀), 5.VIII.1947 (1♀), 13.VIII.1947 (1♀), 25.IX.1955 (1♀), 30.IX.1955 (1 unsexed), J. Minamikawa leg. (NIAES-JMC); 1♀1♂, Mt. Iwamuro, E. Izu, Shizuoka Pref., Tôkai, JAPAN, 11.X.1966, T. Maenami leg. (NIAES); 4♀♀, Hachikita-kôgen (35°24'2"N, 134°32'5"E, 750 m alt.), Ōsasa, Muraoka, Kami City, Mikata County, Hyôgo Pref., Kinki, JAPAN, 6–7.VIII.2018, K. Sakagami leg. (LT) (2♀♀, NSMT, SEN17–DDBJ-LC484182; 2♀♀, NIAES, SEN16–DDBJ-LC484181); 4♀♀1♂, Kotochi (34.119 136.040, 850 m alt.), Kamikitayama Vil., Nara Pref., Kinki, JAPAN, 16–17.VII.2017, S. Fujie leg. (2♀♀, OMNH; 2♀♀1♂, MNHA); 1♀, Komatsu, Kitayama Vil., Wakayama Pref., Kinki, JAPAN, 12.VIII.2009, Ichikawa leg. (KPMNH); 3♀♀, Mt. Tsurugi-yama (33°51'50.8"N, 134°04'42.4"E, 1,250 m alt.), Higashiyasugeoi, Miyoshi City, Tokushima Pref., Shikoku, JAPAN, 22.VIII.2015, Y. Kitayama leg. (LT) (2♀♀, NIAES; 1♀, SEHU); 1♀, Rainbow Highland, Sugasawa, Matsuyama City, Ehime Pref., Shikoku, JAPAN, 27.VII.2014, R. Okano leg. (LT) (EUM, SEN78–DDBJ-LC484183); 1♀1♂, Tengu-kôgen (33°28'38.7"N, 133°0'19.3"E, 1,360 m alt.), Yoshiunotsu, Tsuno Town, Takaoka County, Kôchi, Shikoku, JAPAN, 10.IX.2016, D. Funamoto leg. (LT) (NIAES); 1♀, Tengu-kôgen, Yoshiunotsu, Tsuno Town, Takaoka County, Kôchi Pref., Shikoku, JAPAN, 13.VIII.2015, Y. Nakatani leg. (EUM); 1♀, Tengu-kôgen, Kôchi, Shikoku, JAPAN, 11.VIII.1998, I. Yamashita leg. (LT) (NIAES); 5♂♂, Mt. Eboshi, Haruno, Kôchi, Shikoku, JAPAN, 10.VII.1991 (1♂), 11.VII.1991 (4♂♂), I. Yamashita leg. (NIAES); 6♀♀, Teragawa, Hongawa Vil., Kôchi Pref., Shikoku, JAPAN, 22.VII.1989 (1♀), 11.IX.1989 (1♀), 25.VII.1991 (3♀♀), 18.VII.1993 (1♀), I. Yamashita leg. (NIAES); 1♀, Jigokudani, Kôchi City, Kôchi Pref., Shikoku, JAPAN, 11.VIII.1992, I. Yamashita leg. (LT) (NIAES); 1♀1♂, Mt. Hikosan, Fukuoka Pref, Kyûshû, JAPAN, 29.VIII.1983, K. Ōhara leg. (LT) (NIAES); 1♀1♂, Mt. Hiko-san, Fukuoka Pref., Kyûshû, JAPAN, 22.IX.1983 (1♀), 20–21.X.1983 (1♂), M.T. Chûjô leg. (LT) (NIAES); 1♀1♂, Mt. Hikosan, Fukuoka Pref., Kyûshû, JAPAN, 5.VII.1983 (1♂), 6.VII.1983 (1♀), R. Noda leg. (LT) (NIAES); 1♂, Hiko-san, Fukuoka Pref., Kyûshû, JAPAN, 1.VIII.1953, S. Kimoto leg. (MNHA-SMCM); 1♀, Hiko-san, Fukuoka Pref., Kyûshû, JAPAN, 5.VIII.1955 (MNHA-SMCM); 1♀, Mt. Hiko-san, Fukuoka Pref., Kyûshû, JAPAN, 18–25.VIII.1989, K. Takeno & M. Sharkey leg. (EMUS); 1♀, Nokonoshima, Fukuoka Pref., Kyûshû, JAPAN, 12.IX.1983, K. Konishi leg. (LT) (NIAES); 1♀, Fukuoka Pref., Kyûshû, JAPAN, 30.VIII.1983, K. Ōhara leg. (LT) (NIAES); 1♀, Mt. Shiratori, Izumi Vil., Kumamoto Pref., Kyûshû, JAPAN, 6.VII.1991, R. Noda leg. (NIAES); 1♀, Shiiya pass (1,400 m alt.), Kumamoto Pref., Kyûshû, JAPAN, 15.VI.1985, K. Konishi et al. (LT) (NIAES); 1♀, Mt. Kurodake, Mts. Kujû, Ōita Pref., Kyûshû, JAPAN, 3.X.1983, N. Kôda & R. Noda leg. (LT) (NIAES); 1♀, Mt. Kurodake, Mts. Kujû, Ōita Pref., Kyûshû, JAPAN, 15.IX.1985, K. Konishi leg. (LT) (NIAES); 1♂, Mt. Hoyoshi-dake, Kouyama Town, Kagoshima Pref., Kyûshû, JAPAN, 12–14.VII.1991, R. Noda leg. (LT) (NIAES); 3♀♀, Anbô-rindô (350 m alt.), Yakushima, Kagoshima Pref., Kyûshû, JAPAN, 22.VII.1982, K. Konishi & S. Yoshimatsu leg. (LT) (NIAES); 1♂, Shiratani-unsuikyô, Yakushima, Kagoshima Pref., Kyûshû, JAPAN, 25.VII.1982, K. Konishi & S. Yoshimatsu leg. (LT) (NIAES); 1♀, Chûô-rindô, Amami-ôshima, Kagoshima Pref., Ryûkyûs, JAPAN, 19.IX.1993, M. Yoshida leg. (LT) (NIAES); 1♀, Mt. Yui-dake, Amami-ôshima, Kagoshima Pref., Ryûkyûs, JAPAN, 14.X.2004, H. Makihara leg. (MsT) (KPMNH); 1♀, Kamiya, Amami-ôshima, Kagoshima Pref., Ryûkyûs, JAPAN, 30.VI.1992, R. Noda leg. (NIAES); 1♀, Higashinakama, Amami-ôshima, Kagoshima Pref., Ryûkyûs, JAPAN, 13.V.1983, S. Yoshimatsu & S. Nomura leg. (LT) (NIAES); 1♀, Mt. Yuidake, Amami-ôshima, Kagoshima Pref., Ryûkyûs, JAPAN, 1.IX.1983, R. Noda & K. Hirano leg. (LT) (NIAES); 1♂, Hiji agricultural road (26°43'16.8"N, 128°10'43.4"E, 85 m alt.), Hiji, Kunigami Vil., Kunigami County, Okinawa-hontô, Okinawa Pref., Ryûkyûs, JAPAN, 3–4.VII.2016, S. Shimizu et al. (LT) (NIAES); 2♀♀, Ōkuni-rindô (26°43'38.7"N, 128°12'34.4"E, 265 m alt.), Okuma, Kunigami Vil., Kunigami County, Okinawa-hontô, Okinawa Pref., Ryûkyûs, JAPAN, 30.VI–1.VII.2016, S. Shimizu et al. (LT) (SEHU); 1♀, Ōkuni bridge (26.704179 128.197087, 200 m alt.), Ōkuni-rindô, Hama, Kunigami Vil., Kunigami County, Okinawa-hontô, Okinawa Pref., Ryûkyûs, JAPAN, 6.IV.2019, S. Shimizu leg. (LT) (TARI); 1♀2♂♂, Oku-nigô-rindô (26.815735 128.297288, 200 m alt.), Sosu, Kunigami Vil., Kunigami County, Okinawa-hontô, Okinawa Pref., Ryûkyûs, JAPAN, 7.V.2019, S. Shimizu & T. Tokuhira leg. (LT) (1♀1♂, EMUS; 1♂, CNC); 9♀♀, Ōkuni bridge (26.704179 128.197087, 200 m alt.), Ōkuni-rindô, Hama, Kunigami Vil., Kunigami County, Okinawa-hontô, Okinawa Pref., Ryûkyûs, JAPAN, 29–30.III.2019, S. Shimizu leg. (LT) (2♀♀, NSMT; 5♀♀, KPMNH; 2♀♀, NIAES); 1♀, Okuyona-rindô (26.783608 128.282954, 240 m alt.), Ada, Kunigami Vil., Kunigami County, Okinawa-hontô, Okinawa Pref., Ryûkyûs, JAPAN, 7.IV.2019, S. Shimizu leg. (LT) (NIAES); 2♀♀1♂, Sate (26.774333 128.234803, 230 m alt.), Kunigami Vil., Kunigami County, Okinawa-hontô, Okinawa Pref., Ryûkyûs, JAPAN, 5–6.V.2019, S. Shimizu et al. (LT) (1♀1♂, MNHA; 1♀, NHMUK); 1♂, Janagusuku-rindô (26.688693 128.172478, 200 m alt.), Janagusuku, Ōgimi Vil., Kunigami County, Okinawa-hontô, Okinawa Pref., Ryûkyûs, JAPAN, 8.V.2019, S. Shimizu et al. (LT) (CNC); 1♂, Hama-ichigô-rindô (26.708926 128.181583, 200 m alt.), Hama, Kunigami Vil., Kunigami County, Okinawa-hontô, Okinawa Pref., Ryûkyûs, JAPAN, 7.V.2019, S. Shimizu & T. Tokuhira leg. (LT) (NHMUK); 1♀, Pingtung County (22°25'3"N, 120°43'16"E, 920 m alt.), Chunri Township, TAIWAN, 3.X.2015, S. Shimizu & M. Ito leg. (LT) (TARI, SEN130–DDBJ-LC484184).

##### Distribution.

Eastern Palaearctic and Oriental regions.

JAPAN: [Kantô-Kôshin] Nagano; [Tôkai] Shizuoka; [Kinki] Hyôgo, Nara, and Wakayama; [Shikoku] Tokushima, Ehime, and Kôchi; [Kyûshû] Fukuoka, Ōita, Kumamoto, and Kagoshima; [Ryûkyûs] Kagoshima and Okinawa. This is a rather common species in western and southern Japan.

##### Bionomics.

Unknown.

##### Differential diagnosis.

**Figure 11.**
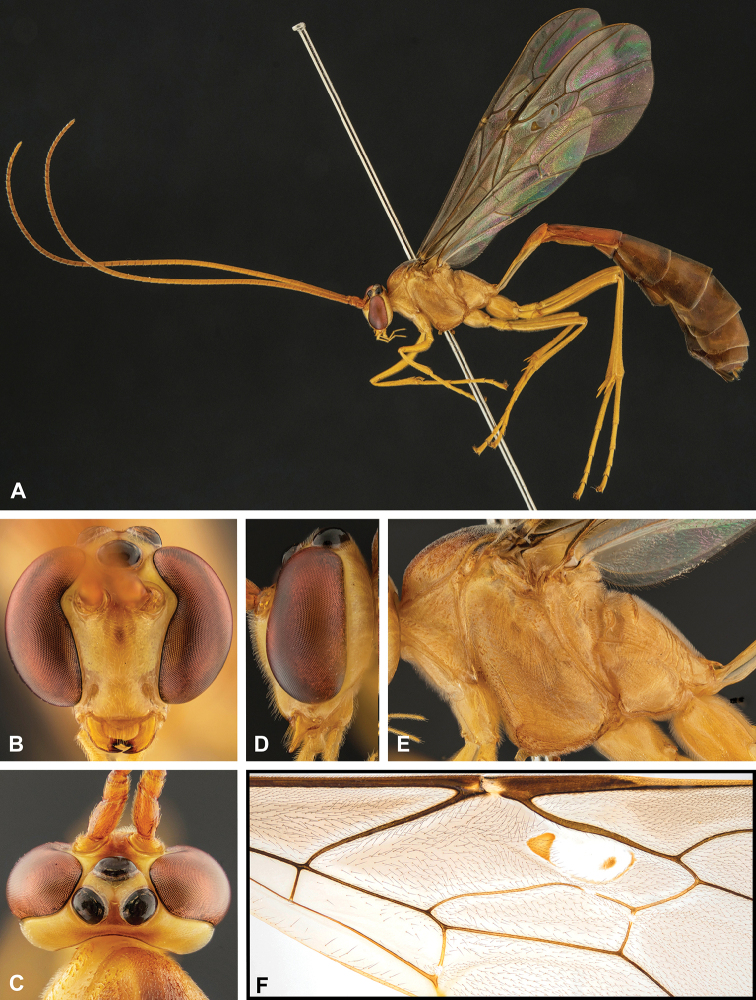
*Enicospilus acutus* Shimizu, sp. nov. ♀ from Japan (PT) **A** habitus **B** head, frontal view **C** head, dorsal view **D** head, lateral view **E** mesosoma, lateral view **F** central part of fore wing.

This species resembles *E. maai* in colour pattern but can be readily distinguished by the following characters: mandible strongly twisted ca. 85° so that outer margin forming acute median longitudinal ridge between near centro-proximal part of mandible and base of mandibular apical teeth (Fig. 11B, D) (mandible more or less weakly twisted less than 35° so that outer mandibular surface without any ridge or groove in *E. maai*); fore wing with AI more than 1.0 (Fig. 11F) (AI less than 0.7 in *E. maai*); fore wing fenestra with strongly pigmented central sclerite (Fig. 11F) (central sclerite absent in *E. maai*); and mesopleuron closely striate to punctostriate (Fig. 11E) (mesopleuron coarsely striate in *E. maai*). This species is uniquely distinctive and easily distinguishable within the Japanese *Enicospilus* on account of the mandibular morphology and colour pattern.

##### Description.

Female (n = 66). Body length 23.5–26.0 (HT: ca. 24.0) mm.

Head with GOI = 2.7–3.3 (HT: 2.8) (Fig. 11D). Lower face 0.7× as wide as high, strongly shiny, finely longitudinally striate centrally, and finely punctate with setae laterally (Fig. 11B). Clypeus 1.5–1.7× (HT: 1.5) as wide as high, sparsely finely punctate with setae, weakly convex in profile, and ventral margin acute (Fig. 11B, D). Malar space 0.4–0.5× (HT: 0.4) as long as basal mandibular width (Fig. 11B, D). Mandible strongly twisted by 80–85° (HT: ca. 85°), moderately long, evenly narrowed, outer surface without diagonal setose groove but with longitudinal acute ridge between near centroproximal part of mandible and base of mandibular teeth (Fig. 11B, D). Mandible teeth equal length, but upper tooth stouter than lower (Fig. 11B). Frons, vertex and gena strongly shiny with fine setae (Fig. 11B–D). Posterior ocellus close to eye (Fig. 11B–D). Ventral end of occipital carina not joining oral carina. Antenna with 54–60 (HT: 56) flagellomeres; first flagellomere 1.6–1.9× (HT: 1.6) as long as second; 20^th^ flagellomere 1.8–2.0× (HT: 1.8) as long as wide.

Mesosoma entirely weakly to moderately shiny with setae (Fig. 11E). Pronotum finely punctate or diagonally wrinkled (Fig. 11E). Mesoscutum 1.4–1.5× (HT: 1.4) as long as maximum width, rather strongly shiny and finely punctate to smooth with setae, and evenly rounded in profile (Fig. 11E). Notauli absent (Fig. 11E). Scutellum moderately convex, smooth with some irregular rugae, with lateral longitudinal carinae along entire length of scutellum. Epicnemium densely punctate with setae. Epicnemial carina strong, straight or very slightly curved, inclined to anterior, dorsal end not reaching anterior margin of mesopleuron (Fig. 11E). Mesopleuron entirely longitudinally striate to punctostriate (Fig. 11E). Submetapleural carina broadened anteriorly (Fig. 11E). Metapleuron coarsely rugose (Fig. 11E). Propodeum evenly rounded in profile; anterior transverse carina complete centrally and not joining pleural carina laterally; anterior area longitudinally striate; spiracular area smooth; posterior area rather coarsely rugose; propodeal spiracle elliptical and joining pleural carina by ridge (Fig. 11E).

Wings (Fig. 11F). Fore wing length 16.5–18.0 (HT: 16.5) mm with AI = 1.0–1.4 (HT: 1.4), CI = 0.4, DI = 0.3, ICI = 0.5, SDI = 1.3–1.4 (HT: 1.4), SI = 0.1–0.2 (HT: 0.1), SRI = 0.3; vein 1m-cu&M sinuate; vein 2r&RS slightly sinuate; vein RS rather evenly curved; fenestra and sclerites of discosubmarginal cell as in Fig. 11F; proximal sclerite triangular, not confluent with distal sclerite, strongly pigmented; central sclerite suboval, pigmented, positioned in centrodistal part of fenestra; distal sclerite pigmented; proximal corner of marginal cell evenly setose; posterodistal corner of second discal cell 90–100° (HT: ca. 100°) and of subbasal cell 80–85° (HT: ca. 80°); vein 1cu-a antefurcal to M&RS by 0.1–0.2× (HT: 0.2) length of 1cu-a. Hind wing with NI = 2.9–3.5 (HT: 2.9); vein RS straight; vein RA with 7–9 (HT: 8) uniform hamuli.

Legs. Hind legs with coxa in profile 1.7–1.8× (HT: 1.8) as long as deep; basitarsus 1.9–2.0× (HT: 2.0) as long as second tarsomere; fourth tarsomere 3.3–3.5× (HT: 3.5) as long as wide; tarsal claw simply pectinate.

Metasoma with DMI = 1.2–1.3 (HT: 1.3), PI = 3.3–3.5 (HT: 3.3), THI = 3.0–3.4 (HT: 3.3); thyridium oval; ovipositor sheath not longer than posterior depth of metasoma.

Colour (Fig. 11). Entirely light to rather dark brown except for apex of mandible, posterior part of mesoscutum, and central part of frons infuscate. Wings moderately infuscate; sclerites pigmented and amber; veins dark reddish brown.

Male (n = 20). Very similar to female.

#### Enicospilus capensis

Taxon classificationAnimaliaHymenopteraIchneumonidae

(Thunberg, 1824)

FB870F7C-19AF-59B0-8378-09F97BADC266

Figure 12

**Figure 12.**
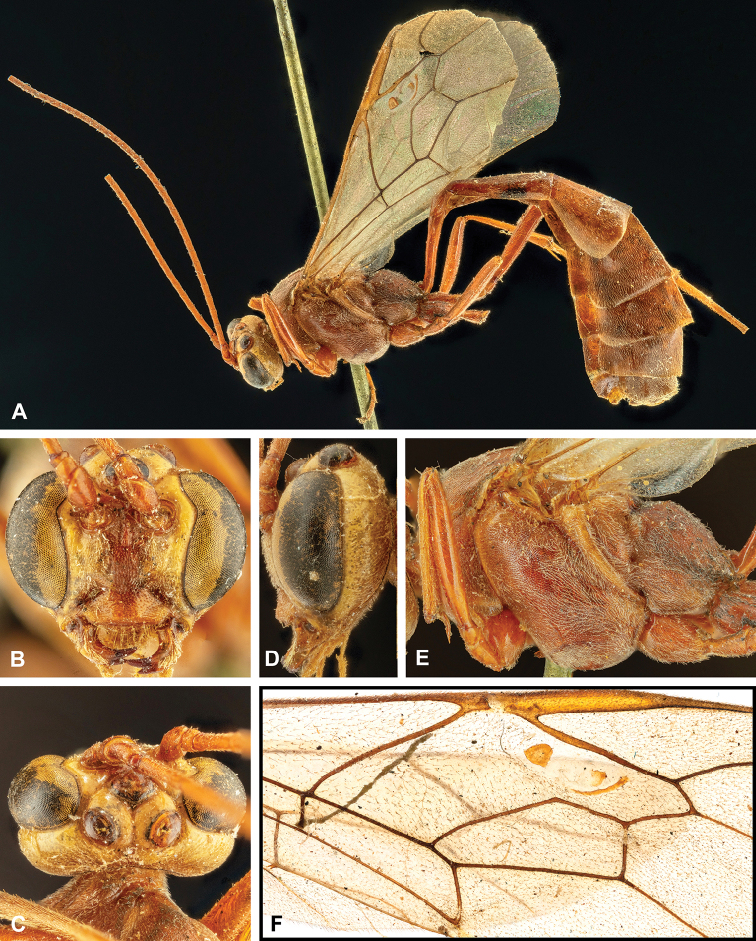
*Enicospilus capensis* (Thunberg, 1824) (HT ♀ of *E. yanagiharai* (Sonan, 1940)) **A** habitus **B** head, frontal view **C** head, dorsal view **D** head, lateral view **E** mesosoma, lateral view **F** central part of fore wing.

Ichneumon capensis Thunberg, 1824: 262; HT ♀ from South Africa, ZIUU, not examined; note that we follow Horstmann (2000) in regarding the authorship of Thunberg’s ichneumonid names as dating from 1824, not 1822; the latter is often incorrectly used (e.g., Yu et al. 2016).
Ophion lativertex Taschenberg, 1875: 435; HT ♀ from Java, FZLU, not examined; synonymised by Gauld and Mitchell (1981: 385).
Ophion antankarus Saussure, 1892: 15; type ♂ from Madagascar, MNHN, not examined; synonymised by Townes and Townes (1973: 174).
Henicospilus montinus Enderlein, 1921: 21; HT ♀ from Java, IZPAN, not examined; synonymised by Gauld and Mitchell (1981: 385).
Henicospilus praedator Enderlein, 1921: 28; HT ♀ from Madagascar, IZPAN, not examined; synonymised by Townes and Townes (1973: 175).
Henicospilus incarinatus Enderlein, 1921: 30; HT ♂ from Madagascar, IZPAN, not examined; synonymised by Townes and Townes (1973: 175).
Henicospilus euxoae Wilkinson, 1928: 261; HT ♀ from Zimbabwe, NHMUK, examined; synonymised by Gauld and Mitchell (1978: 143).
Enicospilus obnoxius Seyrig, 1935: 75; LCT ♀ from Kenya, designated by Townes and Townes (1973: 18), MNHN, not examined; synonymised by Gauld and Mitchell (1978: 143).
Henicospilus yanagiharai Sonan, 1940: 371; HT ♂ from Ryûkyû Island, TARI, examined (Fig. 12); synonymised by Gauld and Mitchell (1981: 385).
Enicospilus selvaraji Rao and Kurian, 1950: 174, 178, 180, 188; nomen nudum.
Enicospilus selvaraji Rao and Kurian, 1951: 68; HT ♀ from India, ZSI, not examined; synonymised by Gauld and Mitchell (1981: 385).
Enicospilus fossatus Chiu, 1954: 63; HT ♀ from Malaysia, TARI, examined; synonymised by Gauld and Mitchell (1981: 385).
Enicospilus indica Rao and Grover, 1960: 280; HT ♀ from India, MUC, destroyed (cf. Gauld and Mitchell (1981: 385)), not examined; synonymised by Gauld and Mitchell (1981: 385).

##### Specimens examined.

Total of 112 specimens (66♀♀42♂♂ and 4 unsexed): Japan (1♀), India (57♀♀41♂♂), Kenya (2♀♀1♂ and 1 unsexed), Madagascar (1♀ and 1 unsexed), Malaysia (1♀), Saudi Arabia (1 unsexed), South Africa (1♀), Uganda (2♀♀ and 1 unsexed), Zimbabwe (1♀).

Type series: HT ♂ of *Henicospilus yanagiharai* Sonan, 1940, Kitadaitô-jima, Okinawa Pref., Ryûkyûs, JAPAN, 18.III.1939, M. Yanagihara leg. (TARI) (Fig. 12); HT ♀ of *Enicospilus fossatus* Chiu, 1954, Jahore, MALAYSIA, 1.X.1916, J. Sonan leg. (TARI); HT ♀ of *Henicospilus euxoae* Wilkinson, 1928, Salisbury, ZIMBABWE, 31.XII.1927, J.I. Roberts leg. (from *Euxoa*) (NHMUK, Type 3b.1289).

##### Distribution.

Afrotropical, Australasian, Oceanic, and Oriental regions (Yu et al. 2016).

JAPAN: [Ryûkyûs] Okinawa (Sonan 1940; present study).

This species has a very wide distribution from South East Asia to South Africa. According to Gauld and Mitchell (1981), this distribution pattern is hardly surprising when considering many of their host moths are also widely distributed throughout the Old World tropics. *Enicospilus capensis* is frequently encountered as a parasitoid of economically important noctuid moths; however, only a single specimen has been collected in Japan.

##### Bionomics.

Recorded from various Lepidoptera hosts, but reliable records are mainly from Noctuidae (e.g., Gauld and Mitchell 1981; Nikam and Gaikwad 1989; Nikam 1990). No host records from Japan.

##### Differential diagnosis.

The Japanese specimen of *E. capensis* is very easily distinguishable from all other Japanese *Enicospilus* specimens on account of very wide face (i.e., lower face 1.2× as wide as high, as in Fig. 12B) and long mandible. This species is morphologically similar to *E. ramidulus*, but distinguishable by the following combination of morphological characters: metapleuron matt (Fig. 12E) (metapleuron evenly moderately punctate and never matt in *E. ramidulus*, as in Fig. 39E); metasoma usually entirely orange-brown (Fig. 12A) (posterior metasomal segments usually strongly infuscate in *E. ramidulus*, as in Fig. 39A). This species is usually morphologically rather uniform, but the Japanese specimen has a much broader lower face than others (Fig. 12B). However, there does not seem to be enough of a difference to justify a separate species, as Gauld and Mitchell (1981) also concluded.

#### Enicospilus combustus

Taxon classificationAnimaliaHymenopteraIchneumonidae

(Gravenhorst, 1829)

9225188C-7A6B-5AE5-A154-1AE9D52B2A16

Figure 13

Ophion combustus Gravenhorst, 1829: 701; type lost?

##### Specimens examined.

Total of 54 specimens (39♀♀14♂♂ and 1 unsexed): England (18♀♀2♂♂), Japan (19♀♀10♂♂ and 1 unsexed), Russia (1♂), unknown (2♀♀1♂).

##### Distribution.

Oriental and Palaearctic regions (Yu et al. 2016); this is a predominantly Palaearctic species that may be restricted to there, although Lee et al. (2011) reported this species from the Oriental region, probably based on a misidentification.

JAPAN: [Hokkaidô] (Uchida 1928, 1935; present study); [Honshû] (Uchida 1935; present study); [Tôhoku] Aomori (Uchida 1928; present study), Yamagata*, and Fukushima (Uchida 1928; present study); [Hokuriku] Niigata (Uchida 1928; present study); [Kantô-Kôshin] Tochigi (Uchida 1928; present study), Nagano*, and Tôkyô (Uchida 1928, 1930); [Tôkai] Gifu (Uchida 1928); [Kinki] Kyôto* and Hyôgo*; [Chûgoku] Hiroshima*; [Shikoku] (Uchida 1935); [Kyûshû] (Uchida 1935), Fukuoka*. *New records.

##### Bionomics.

Reared from one species of Noctuidae in Japan: *Trachea tokiensis* (Butler, 1884) (Uchida 1928, 1930). Notodontid and noctuid moths are recorded as hosts, but reliable records are only from Noctuidae of the subfamily Hadeninae (e.g., Broad and Shaw 2016).

##### Differential diagnosis.

**Figure 13.**
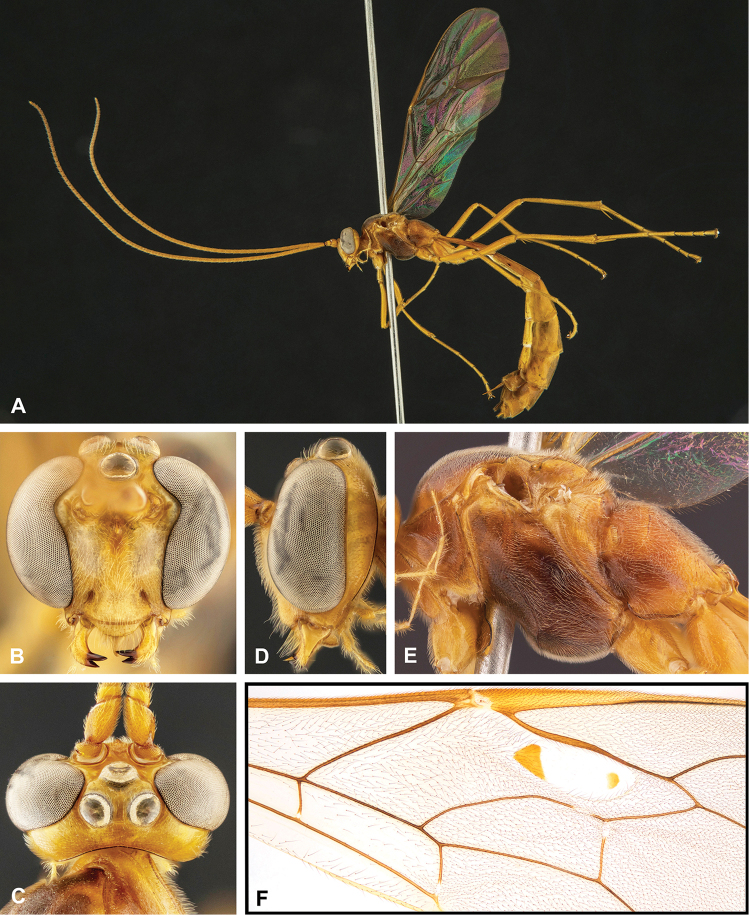
*Enicospilus combustus* (Gravenhorst, 1829) ♀ from Japan **A** habitus **B** head, frontal view **C** head, dorsal view **D** head, lateral view **E** mesosoma, lateral view **F** central part of fore wing.

This species is usually very easily distinguished from all other Palaearctic *Enicospilus* species by the black mesosoma, thyridium, and posterior segments of metasoma, as in Fig. 13A. *Enicospilus combustus* has sometimes been confused with *E. multidens* stat. rev., *E. ramidulus*, and *E. shikokuensis*; moreover, some authors have treated *E. combustus* and *E. ramidulus* as a single species (e.g., Viktorov 1957; Townes et al. 1965; Gauld and Mitchell 1981). However, *E. combustus* is easily separated from *E. shikokuensis* by the separated proximal and distal sclerites of fore wing fenestra, as in Fig. 13F (proximal and distal sclerites usually obviously confluent in *E. shikokuensis*, as in Fig. 44F), from *E. multidens* stat. rev. and *E. ramidulus* by the entirely more or less blackish mesosoma, as in Fig. 13A, E (mesosoma entirely orange-brown in *E. multidens* stat. rev. and *E. ramidulus*, as in Figs 29A, E and 39A, E respectively). Moreover, this species is similar to *E. sharkeyi* sp. nov. in colour pattern (Figs 13, 43), however, *E. combustus* can be readily distinguished from it by many characters, such as separated proximal and distal sclerites of fore wing fenestra, as in Fig. 13F (proximal and distal sclerites confluent in *E. sharkeyi* sp. nov., as in Fig. 43F), larger central sclerite of fore wing fenestra, as in Fig. 13F (central sclerite smaller in *E. sharkeyi* sp. nov., as in Fig. 43F), wider lower face, as in Fig. 13B (narrower in *E. sharkeyi* sp. nov., as in Fig. 43B), etc.

#### Enicospilus concentralis

Taxon classificationAnimaliaHymenopteraIchneumonidae

Cushman, 1937

853E06F8-C0AB-50BF-8D38-8CE761789B1A

Figure 14

Enicospilus concentralis Cushman, 1937: 305; HT ♀ from Taiwan, DEI, not examined.

##### Specimens examined.

Total of 59 specimens (51♀♀6♂♂ and 2 unsexed): Brunei (27♀♀2♂♂), Japan (22♀♀2♂♂ and 1 unsexed), Taiwan (2♀♀2♂♂ and 1 unsexed).

##### Distribution.

Australasian, Eastern Palaearctic, and Oriental regions (Yu et al. 2016).

JAPAN: [Kantô-Kôshin] Tôkyô* and Kanagawa*; [Kinki] Hyôgo*; [Chûgoku] Hiroshima (Konishi and Nakamura 2010; present study); [Shikoku] Tokushima*, Ehime (Konishi and Yamamoto 2000; present study), and Kôchi*; [Kyûshû] Fukuoka (Konishi 1993; present study), Nagasaki (Konishi 1993; present study), Ōita (Konishi 1993; present study), and Kumamoto (Konishi 1993; present study). *New records. *Enicospilus concentralis* is restricted to the warmer Pacific coast in Japan although has not been recorded from Ryûkyûs.

##### Bionomics.

Hosts unknown.

Although adult wasps are most active during summer, it is also relatively easily encountered in winter: hibernating adults are often found on the underside of leaves of evergreen plants (such as Aucubaceae shrubs).

##### Differential diagnosis.

**Figure 14.**
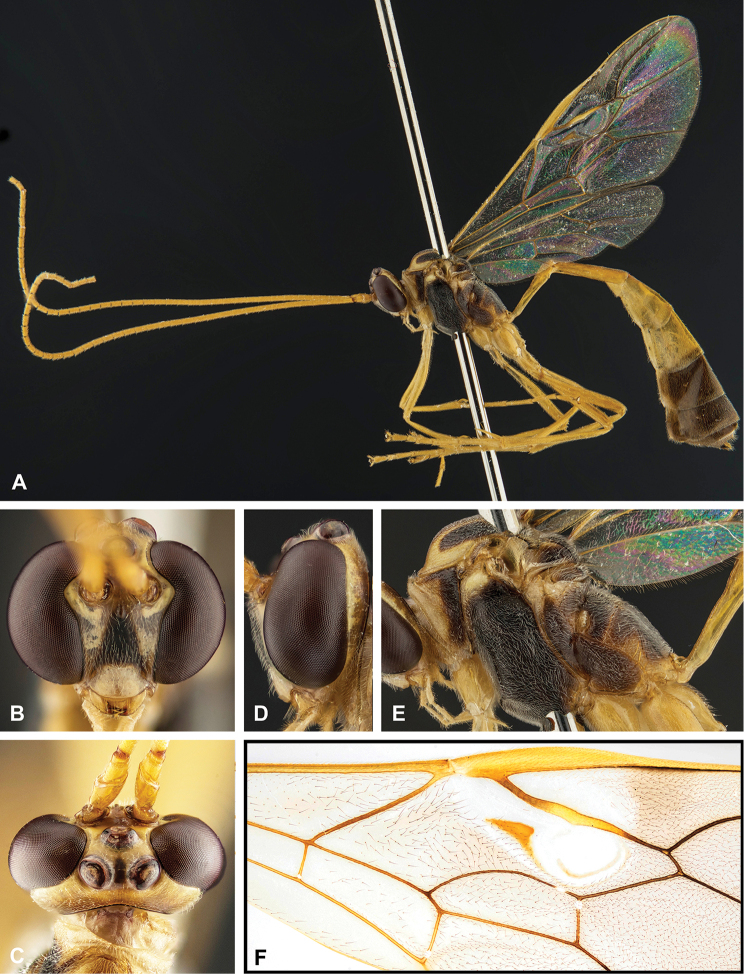
*Enicospilus concentralis* Cushman, 1937 ♀ from Japan **A** habitus **B** head, frontal view **C** head, dorsal view **D** head, lateral view **E** mesosoma, lateral view **F** central part of fore wing.

Wing characters of this species (e.g., fore wing with proximal part of marginal cell widely glabrous, CI = 0.1–0.3, ICI = 0.2–0.3, and central sclerite of fore wing fenestra linear and parallel to distal margin of fenestra, as in Fig. 14F) are unique within the genus; hence, this species can very easily be distinguished from all other species of *Enicospilus*.

#### Enicospilus dasychirae

Taxon classificationAnimaliaHymenopteraIchneumonidae

Cameron, 1905

EA2D7610-547C-5A81-9F30-5D685069AEBF

Figure 15

Eniscospilus (sic) dasychirae Cameron, 1905a: 123; HT ♀ from Sri Lanka, NHMUK, examined.
Eniscospilus (sic) horsfieldi Cameron, 1905a: 124; HT ♀ from Sri Lanka, NHMUK, examined; synonymised by Townes et al. (1961: 273).
Henicospilus borneensis Szépligeti, 1906: 138; HT ♀ from Borneo, TM, not examined; synonymised by Townes et al. (1961: 273).
Enicospilus nigrimarginalis Cushman, 1937: 311; HT ♂ from Taiwan, DEI, not examined; synonymised by Townes et al. (1961: 274).

##### Specimens examined.

Total of 49 specimens (44♀♀5♂♂): Japan (8♀♀3♂♂), Sri Lanka (2♀♀), Taiwan (34♀♀2♂♂).

Type series: HT ♀ of *Eniscospilus* (sic) *dasychirae* Cameron, 1905, Pundaluoya, SRI LANKA, I.1899, P. Cameron leg. (NHMUK, Type 3b.1267); HT ♀ of *Eniscospilus* (sic) *horsfieldi* Cameron, 1905, Peradeniya, SRI LANKA, IX.1902, P. Cameron leg. (NHMUK, Type 3b.1265).

##### Distribution.

Australasian, Eastern Palaearctic, and Oriental regions (Yu et al. 2016); this is a predominantly Oriental species.

JAPAN: [Ryûkyûs] Okinawa (Chiu 1954; Gauld and Mitchell 1981; present study). Though this species is widely distributed in Southeast Asia, it is restricted to Okinawa in Japan.

##### Bionomics.

Recorded as a parasitoid of several species of Erebidae (subfamily Lymantriinae) (Gauld and Mitchell 1981; Chiu et al. 1984; Chen et al. 2009) and Noctuidae (Tang 1990), although there are no host records from Japan.

##### Differential diagnosis.

**Figure 15.**
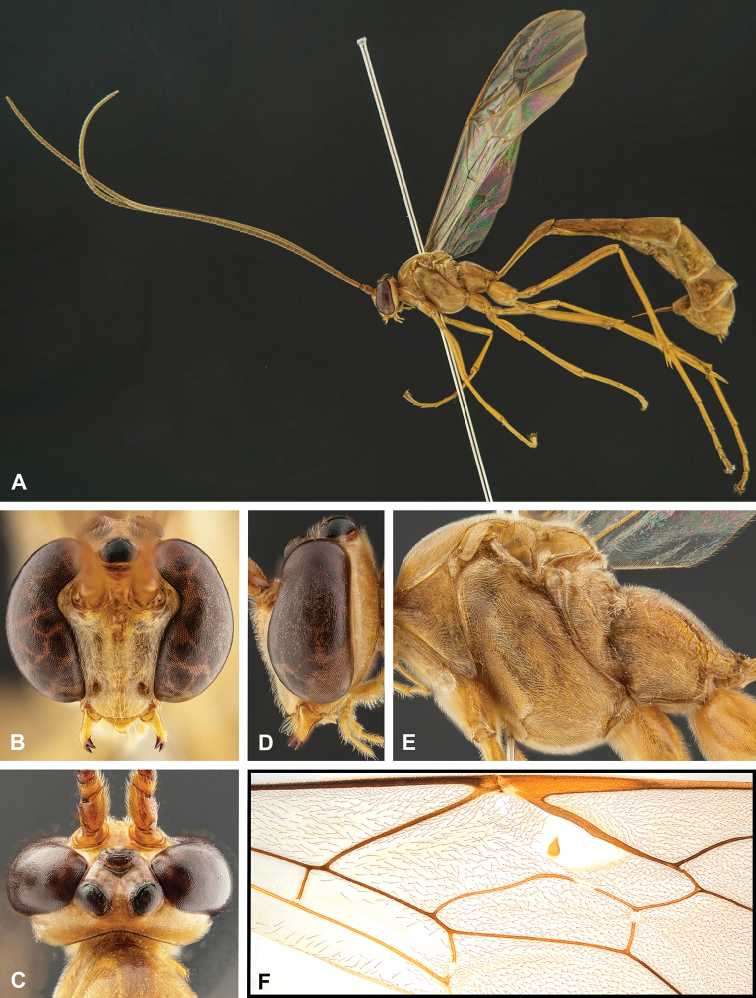
*Enicospilus dasychirae* Cameron, 1905 ♀ from Japan **A** habitus **B** head, frontal view **C** head, dorsal view **D** head, lateral view **E** mesosoma, lateral view **F** central part of fore wing.

This species is readily distinguishable from all other *Enicospilus* species by the unique small drop-shaped and isolated proximal sclerite of fore wing fenestra, as in Fig. 15F.

#### Enicospilus erythrocerus

Taxon classificationAnimaliaHymenopteraIchneumonidae

(Cameron, 1905)

E8428F60-4737-5FF5-87B2-C01281E94467

Figure 16

Pleuroneurophion erythrocerus Cameron, 1905a: 121; HT ♀ from Sri Lanka, NHMUK, examined.
Allocamptus orientalis Uchida, 1928: 230; LCT ♀ from Okinawa, designated by Gauld and Mitchell (1981: 175), SEHU, examined; synonymised by Townes et al. (1961: 275); junior secondary homonym of Enicospilus orientalis (Morley, 1913).
Enicospilus hirayamai Uchida, 1955: 120; replacement name for Enicospilus orientalis (Uchida, 1928).

##### Specimens examined.

Total of 14 specimens (12♀♀2♂♂): Japan (2♀♀1♂), Malaysia (6♀♀), Philippines (1♀), Sri Lanka (3♀♀), Taiwan (1♂).

Type series: HT ♀ of *Pleuroneurophion erythrocerus* Cameron, 1905, Peradeniya, SRI LANKA (NHMUK, Type 3b.1214); LCT ♀ of *Allocamptus orientalis* Uchida, 1928, Okinawa Pref., Ryûkyûs, JAPAN, VII.1926, Hirayama leg. (SEHU).

##### Distribution.

Australasian, Eastern Palaearctic, and Oriental regions (Yu et al. 2016); this is a predominantly Oriental species.

JAPAN: [Ryûkyûs] Okinawa (Uchida 1928; present study).

##### Bionomics.

Recorded from Erebidae by Sharma (1985).

##### Differential diagnosis.

**Figure 16.**
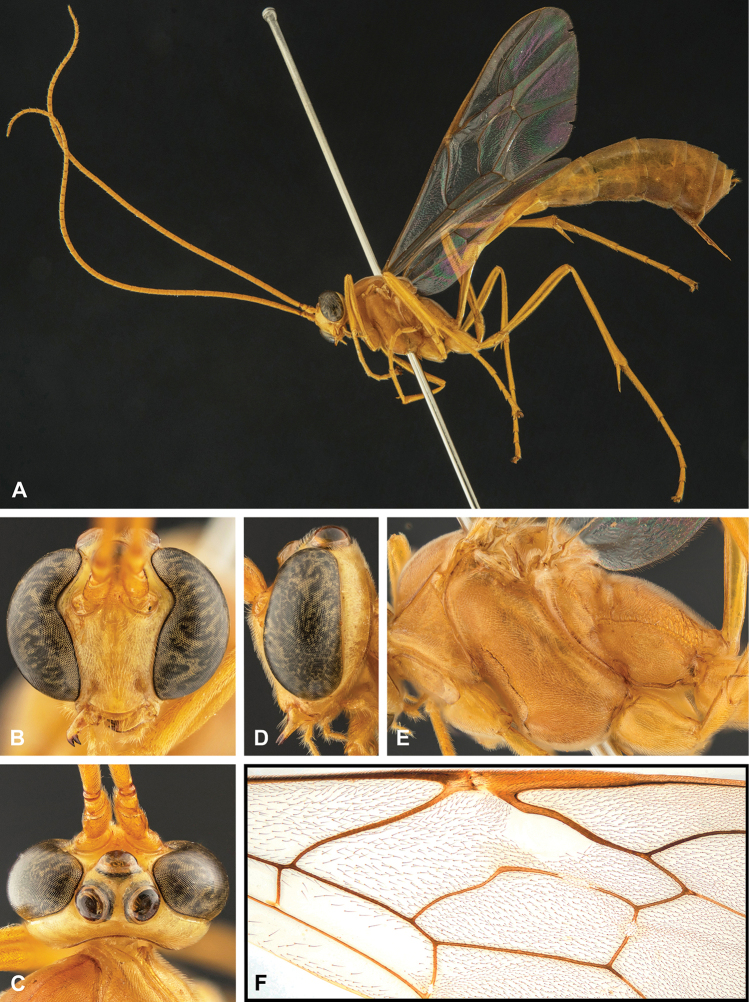
*Enicospilus erythrocerus* (Cameron, 1905) ♀ from Japan **A** habitus **B** head, frontal view **C** head, dorsal view **D** head, lateral view **E** mesosoma, lateral view **F** central part of fore wing.

This species especially resembles *E. grandis* (Cameron, 1905) and *E. plicatus* (Brullé, 1846), but is distinguishable by the smaller size, shorter antennae, and more matt and uniformly punctate meso- and metapleuron (Fig. 16A, E). Other than this species, all Japanese *Enicospilus* species have at least one fore wing sclerite; hence, it is fortunately very easily identifiable.

##### Remarks.

*Allocamptus orientalis* was described based on two females and one male from Okinawa and Taiwan (Uchida 1928). Although the Taiwanese specimen was designated as the lectotype by Gauld and Mitchell (1981), the lectotype label was attached to a Japanese specimen.

#### Enicospilus flavocephalus

Taxon classificationAnimaliaHymenopteraIchneumonidae

(Kirby, 1900)

DE1BEE97-30D3-53BE-B3FD-9C09EFB91384

Figure 17

Ophion flavocephalus Kirby, 1900: 82; LCT ♂ from Christmas Island, designated by Gauld (1977: 79), NHMUK, examined.
Henicospilus lunulatus Szépligeti, 1906: 143; HT ♂ from Bismarck Island, TM, not examined; synonymised by Gauld and Mitchell (1981: 416).
Henicospilus albicaput Morley, 1912: 50; HT ♂ from Australia, NHMUK, examined; synonymised by Townes et al. (1961: 275).
Henicospilus similis Matsumura and Uchida, 1926: 221; HT ♂ from Ryûkyûs, SEHU, examined; synonymised by Uchida (1928: 221).

##### Specimens examined.

Total of 57 specimens (34♀♀22♂♂ and 1 unsexed): Australia (5♀♀2♂♂ and 1 unsexed), Brunei (2♀♀1♂), Japan (21♀♀13♂♂), Laos (1♀), Singapore (1♀), Taiwan (4♀♀6♂♂).

Type series: LCT ♂ of *Ophion flavocephalus* Kirby, 1900, Flying Fish Cove, Christmas Island, AUSTRALIA, C.W. Andrews leg. (NHMUK, Type 3b.1273); HT ♂ of *Henicospilus albicaput* Morley, 1912, Mackay, Queensland, AUSTRALIA (NHMUK, Type 3b.1254); HT ♂ of *Henicospilus similis* Matsumura & Uchida, 1926, Okinawa, Ryûkyûs, JAPAN, S. Sakaguchi leg. (SEHU).

##### Distribution.

Australasian, Oceanic, and Oriental regions (Yu et al. 2016); new to the Eastern Palaearctic region.

Newly recorded from Laos.

JAPAN: [Kyûshû] Kagoshima*; [Ryûkyûs] Kagoshima (Uchida 1956; present study) and Okinawa (Matsumura and Uchida 1926; Uchida 1928; Sonan 1940; present study). *New record.

##### Bionomics.

Recorded as a parasitoid of three species of *Euproctis* (Erebidae: Lymantriinae) (Corbett and Miller 1928; Sonan 1944; Chen et al. 2009). Other host records seem less likely, and there are no host records from Japan.

##### Differential diagnosis.

**Figure 17.**
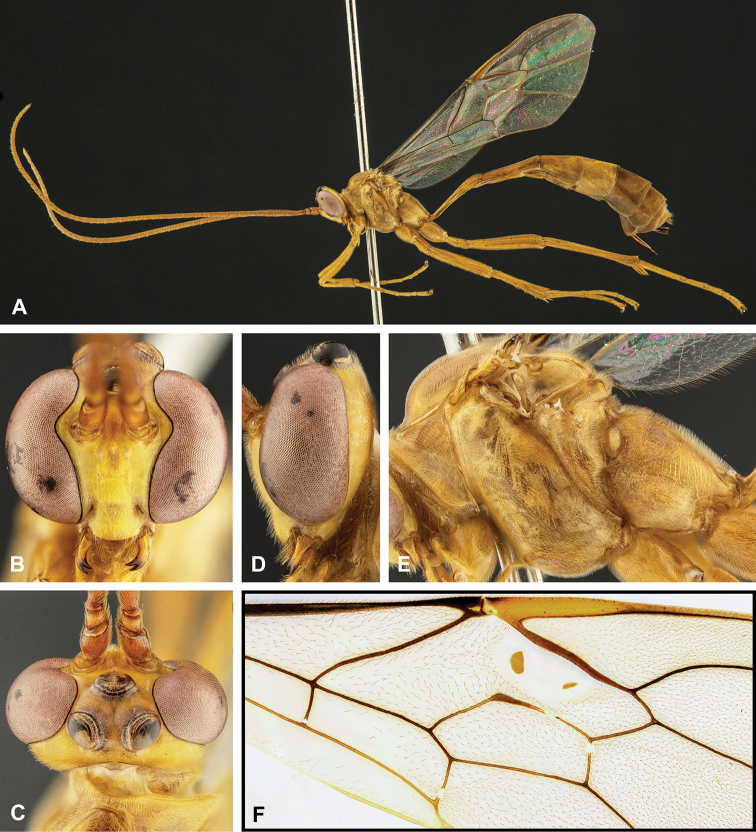
*Enicospilus flavocephalus* (Kirby, 1900) ♀ from Japan **A** habitus **B** head, frontal view **C** head, dorsal view **D** head, lateral view **E** mesosoma, lateral view **F** central part of fore wing.

This species is easily distinguishable from all other *Enicospilus* species by the angulate fore wing vein 1m-cu&M, large value of CI (i.e., 0.6–0.8), and characteristic sclerites of fore wing fenestra (Fig. 17F). *Enicospilus flavocephalus* is very similar to *E. xanthocephalus* in colour pattern, body size, and body profile, as in Figs 17 and 52, but these species are easily distinguishable from each other by the colour of interocellar area (entirely yellow-brown in *E. flavocephalus*, as in Fig. 17C, but entirely black in *E. xanthocephalus*, as in Fig. 52C), shape of fore wing vein 1m-cu&M (angulate in *E. flavocephalus*, as in Fig. 17F, but evenly curved in *E. xanthocephalus*, as in Fig. 52F), etc.

#### Enicospilus formosensis

Taxon classificationAnimaliaHymenopteraIchneumonidae

(Uchida, 1928)

0CFCE565-FC5B-5878-9D85-9A07B75F9D97

Figure 18

Henicospilus formosensis Uchida, 1928: 223; HT ♀ from Taiwan, SEHU, examined.
Enicospilus saepis Chiu, 1954: 77; HT ♀ from Japan, TARI, examined; synonymised by Gauld and Mitchell (1981: 424).
Enicospilus vacuus Gauld and Mitchell, 1981: 453; HT ♀ from Okinawa, EMUS, examined; **syn. nov.**

##### Specimens examined.

Total of 55 specimens (19♀♀35♂♂ and 1 unsexed): Brunei (1♂), India (1 unsexed), Japan (18♀♀32♂♂), Laos (1♂), Taiwan (1♀), unknown (1♂).

Type series: HT ♀ of *Henicospilus formosensis* Uchida, 1928, Baibara [= Meiyuan], TAIWAN, 15.VI.1926, Y. Saito & Kikuchi leg. (SEHU); HT ♀ of *Enicospilus vacuus* Gauld & Mitchell, 1981, Chizuka, Okinawa, Ryûkyûs, JAPAN, VII–IX, G.E. Bohart & C.L. Harnage leg. (EMUS); PT ♀ of *E. vacuus*, same data and repository as HT; HT ♀ of *Enicospilus saepis* Chiu, 1954, Nara, Kinki, JAPAN, 17.VIII.1918, J. Sonan leg. (TARI).

##### Distribution.

Eastern Palaearctic and Oriental regions (Yu et al. 2016).

Newly recorded from Laos and Malaysia.

JAPAN: [Kantô-Kôshin] Tôkyô*; [Tôkai] Mie*; [Kinki] Ōsaka (Chiu 1954; present study), Nara (Chiu 1954; Shimizu 2020; present study), and Wakayama*; [Chûgoku] Hiroshima (Konishi and Nakamura 2005, 2010; present study) and Yamaguchi*; [Shikoku] Ehime (Konishi and Yamamoto 2000; present study) and Kôchi*; [Kyûshû] Fukuoka*, Saga (Chiu 1954; present study), Nagasaki*, Kumamoto (Chiu 1954; present study), and Kagoshima*; [Ryûkyûs] Kagoshima* and Okinawa (Gauld and Mitchell 1981; present study). *New records.

##### Bionomics.

Unknown.

##### Differential diagnosis.

**Figure 18.**
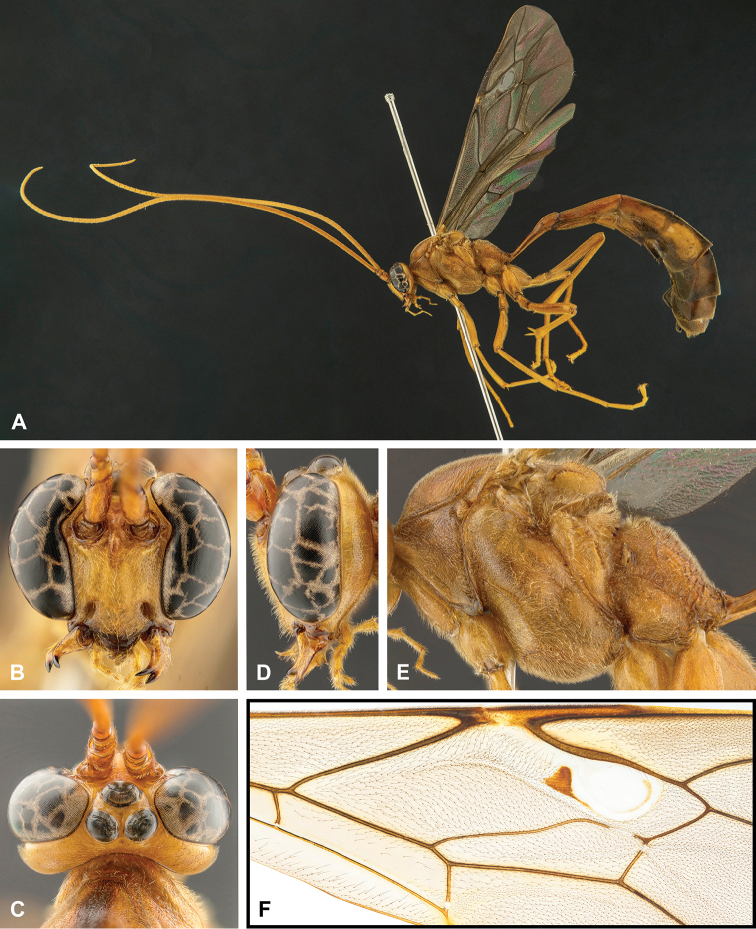
*Enicospilus formosensis* (Uchida, 1928) ♀ from Japan **A** habitus **B** head, frontal view **C** head, dorsal view **D** head, lateral view **E** mesosoma, lateral view **F** central part of fore wing.

This species is easily distinguishable by the wide face (Fig. 18B), unique conspicuous long line of setae of fore wing discosubmarginal cell (Fig. 18F), shape of central sclerite of fore wing fenestra (Fig. 18F), quadrate scutellum, and large size.

##### Remarks.

Gauld and Mitchell (1981) had separated *E. formosensis* and *E. vacuus* based on differences of value of CI. However, the CI of these ‘species’ are continuous and no other morphological differences could be recognised. Tang (1990) also suggested that these names represented the same species. Hence, *E. vacuus* is newly synonymised under *E. formosensis* in the present paper.

#### Enicospilus insinuator

Taxon classificationAnimaliaHymenopteraIchneumonidae

(Smith, 1860)

D8E9B449-DA03-5950-9455-CC17B12CDE0C

Figure 19

Ophion insinuator Smith, 1860: 141; HT ♀ from Moluccas, OUMNH, not examined.
Enicospilus zyzzus Chiu, 1954: 23; HT ♀ from China, TARI, examined; synonymised by Gauld and Mitchell (1981: 353).

##### Specimens examined.

Total of 36 specimens (35♀♀1♂): Brunei (1♀), China (1♀), Japan (28♀♀1♂), Taiwan (5♀♀).

Type series: HT ♀ of *Enicospilus zyzzus* Chiu, 1954, Foochow, Fukien, CHINA, 13.II.1948, H.F. Chao leg. (TARI); PT ♀ of *Enicospilus zyzzus*, Oshima, Kyûshû, JAPAN, V.1930, Takahashi leg. (TARI).

##### Distribution.

Australasian, Eastern Palaearctic, and Oriental regions (Yu et al. 2016).

Newly recorded from Taiwan.

JAPAN: [Kyûshû] Kagoshima*; [Ryûkyûs] Kagoshima* and Okinawa*. * New records. Chiu (1954) has recorded this species from Japan, but lacking collection locality data. Most Japanese specimens were collected in Yakushima by Malaise traps.

##### Bionomics.

Unknown.

##### Differential diagnosis.

**Figure 19.**
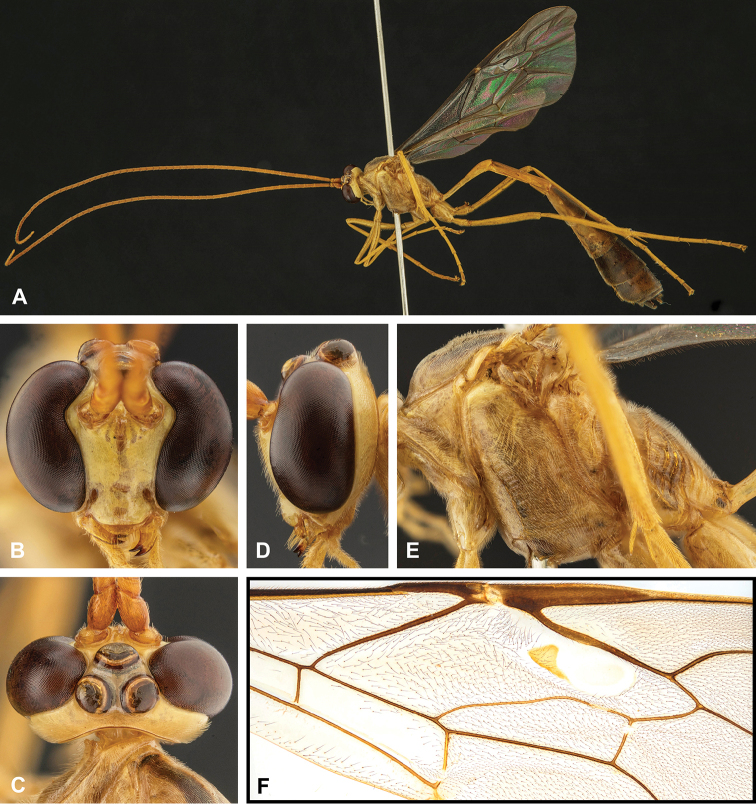
*Enicospilus insinuator* (Smith, 1860) ♀ from Japan **A** habitus **B** head, frontal view **C** head, dorsal view **D** head, lateral view **E** mesosoma, lateral view **F** central part of fore wing.

This species is closest to *E. pallidistigma* Cushman, 1937, but distinguished from it by the smaller fore wing fenestra and weaker central sclerite (= quadra) (Fig. 19F). According to Gauld and Mitchell (1981), this species also resembles *E. maai* and *E. rogus*, but is easily distinguishable from them by the mesoscutum colour (i.e., mesoscutum with longitudinal vittae or rarely entirely infuscate in *E. insinuator*, but only posterior part of mesoscutum strongly infuscate in *E. maai* and *E. rogus*). Furthermore, it differs from *E. maai* by the shape of fore wing fenestra (i.e., fenestra moderate length and anterodistal corner of fenestra antefurcal to RS by more than 0.7× length of 2rs-m in *E. insinuator*, as in Fig. 19F; but fenestra very long and anterodistal corner of fenestra interstitial to RS in *E. maai*) and from *E. rogus* by the torsion of mandible (i.e., mandible twisted by 15–35° in *E. insinuator*, as in Fig. 19B, D; but 75–80° in *E. rogus*).

#### Enicospilus javanus

Taxon classificationAnimaliaHymenopteraIchneumonidae

(Szépligeti, 1910)

6E0DC2D0-3B83-56DB-BB8E-B194BCF07BF4

Figure 20

Henicospilus javanus Szépligeti, 1910: 93; HT ♀ from Java, TM, not examined.
Enicospilus fulacorensis Brues, 1918: 117; HT ♀ from Solomon Island, MCZ, not examined; synonymised by Gauld and Mitchell (1981: 260).
Enicospilus gephyrus Chiu, 1954: 32; HT ♀ from Japan, TARI, examined; synonymised by Gauld and Mitchell (1981: 260).
Enicospilus (Bicorniata) diurnus Nikam, 1975: 193, 194; HT ♀ from India, MUC, not examined; synonymised by Gauld and Mitchell (1981: 260).

##### Specimens examined.

Total of 122 specimens (103♀♀19♂♂): Brunei (30♀♀2♂♂), India (2♀♀), Japan (64♀♀16♂♂), Papua New Guinea (4♀♀), Singapore (1♀), Sri Lanka (2♀♀), Taiwan (1♂).

Type series: HT ♀ of *Enicospilus gephyrus* Chiu, 1954, Tokusa, Yamaguchi Pref., Chûgoku, JAPAN, 1.VIII.1922, T. Shiraki leg. (TARI).

##### Distribution.

Australasian, Eastern Palaearctic, and Oriental regions (Yu et al. 2016).

JAPAN: [Kantô-Kôshin] Kanagawa (Watanabe et al. 2016); [Tôkai] Shizuoka* and Mie*; [Kinki] Kyôto*, Ōsaka*, and Hyôgo*; [Chûgoku] Hiroshima* and Yamaguchi (Chiu 1954; present study); [Shikoku] Tokushima*, Ehime (Konishi and Yamamoto 2000; present study), and Kôchi*; [Kyûshû] Fukuoka* and Kumamoto*; [Ryûkyûs] Kagoshima*. *New records. It is restricted to the warmer Pacific coast in Japan.

##### Bionomics.

Unknown.

##### Differential diagnosis.

**Figure 20.**
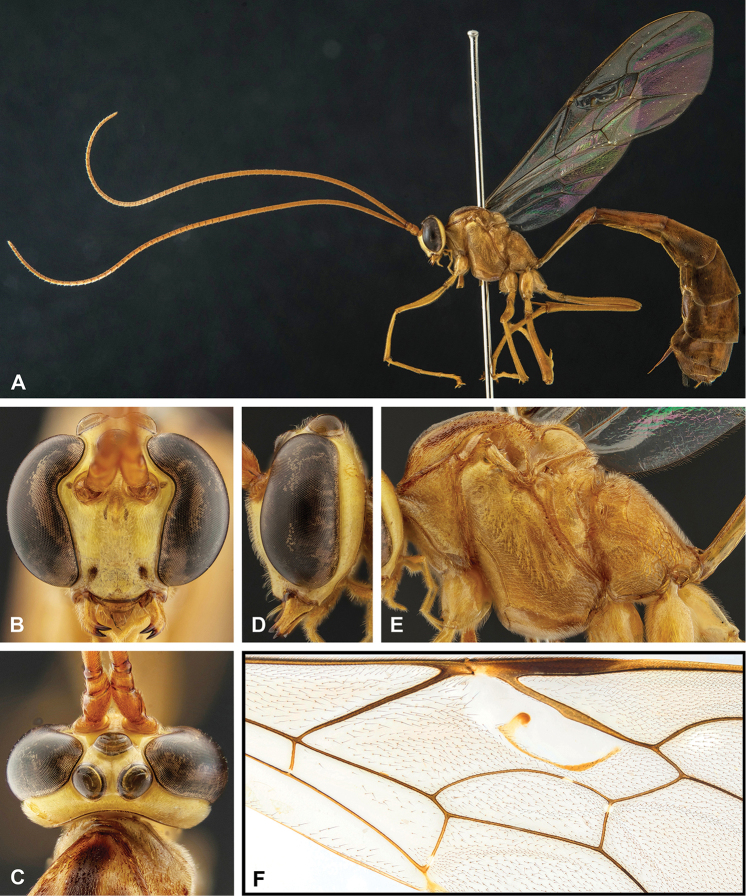
*Enicospilus javanus* (Szépligeti, 1910) ♀ from Japan **A** habitus **B** head, frontal view **C** head, dorsal view **D** head, lateral view **E** mesosoma, lateral view **F** central part of fore wing.

This species is very easily distinguishable from any other Japanese *Enicospilus* by its characteristic fenestra and sclerites of fore wing (i.e., fenestra widened proximally, and proximal and distal sclerites confluent and shaped like a letter ‘P’, as in Fig. 20F).

##### Remarks.

*Enicospilus javanus* exhibits a very wide range of variation in the shape of fore wing sclerites, body size, and colour, as mentioned by Gauld and Mitchell (1981: 262). We examined 24 female specimens from the same locality and date collected by I. D. Gauld in Brunei, and these specimens indeed show an extremely wide range of morphological variation. However, the variation is more or less continuous. This variation suggests that this species includes some cryptic species and integrative approaches are needed to reveal species boundaries. Regarding the Japanese population, it is very stable, suggesting that there are no cryptic species included.

#### Enicospilus jilinensis

Taxon classificationAnimaliaHymenopteraIchneumonidae

Tang, 1990

FDB5C5B4-B643-5A5D-9482-7D1C9F4EFA2A

Figure 21

Enicospilus jilinensis Tang, 1990: 72; HT ♀ from Jilin, China, FAFU, not examined.

##### Specimens examined.

Total of 3 specimens (2♀♀1♂): Japan (2♀♀1♂).

##### Distribution.

Eastern Palaearctic region (Tang 1990; Yu et al. 2016).

Newly recorded from Japan.

JAPAN: [Kantô-Kôshin] Tôkyô and Chiba. This species is collected only from a big city in Japan.

##### Bionomics.

Unknown.

##### Differential diagnosis.

**Figure 21.**
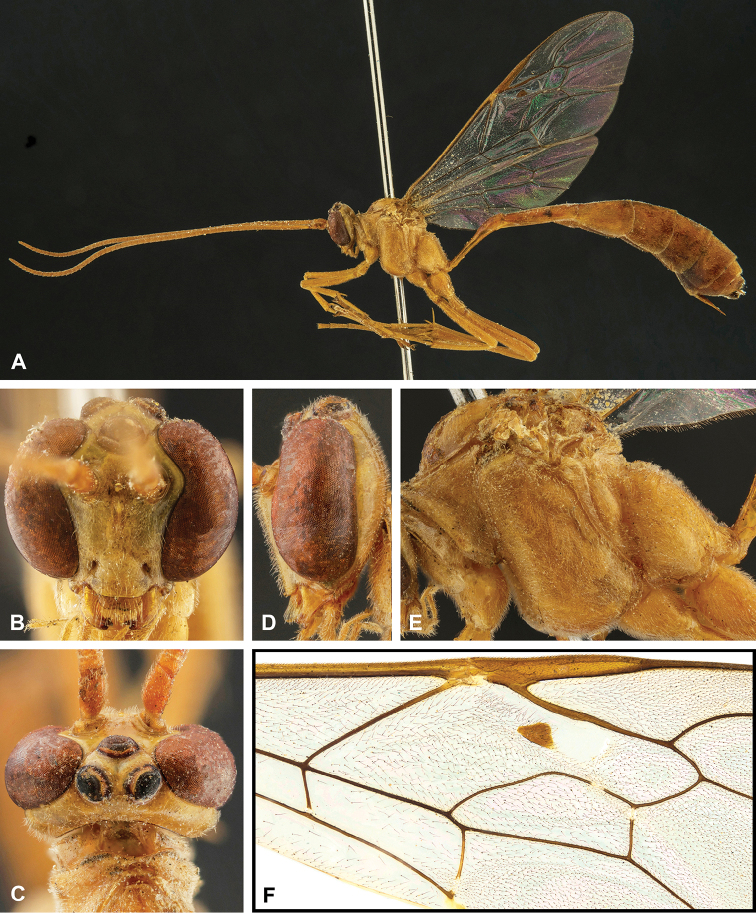
*Enicospilus jilinensis* Tang, 1990 ♀ from Japan **A** habitus **B** head, frontal view **C** head, dorsal view **D** head, lateral view **E** mesosoma, lateral view **F** central part of fore wing.

All Japanese specimens had been misidentified as *E. yonezawanus*, because they lack the central sclerite of fore wing fenestra and have a strongly pigmented triangular proximal sclerite, as in Figs 21F, 54F. However, *E. jilinensis* can be distinguished from *E. yonezawanus* by many characters, such as the smooth outer mandibular surface, smaller fore wing fenestra, entirely punctate mesopleuron, and larger value of ICI.

##### Remarks.

Some morphological features (e.g., flattened clypeus, moderately long and slender mandible, and absence of central sclerite of fore wing fenestra) suggest a relation to *E. shinkanus*, which should be tested when fresh specimens are available.

#### Enicospilus kikuchii

Taxon classificationAnimaliaHymenopteraIchneumonidae

Shimizu, 2017

7EDFC1C7-8A1C-548A-A122-68655B918FE6

Figure 22

Enicospilus kikuchii Shimizu, 2017: 187; HT ♀ from Japan, NIAES, examined.

##### Specimens examined.

Total of 6 specimens (5♀♀1♂): Japan (5♀♀1♂).

Type series: HT ♀ of *Enicospilus kikuchii* Shimizu, 2017, Kawamata, Chichibu, Saitama Pref., Kantô-Kôshin, JAPAN, 28–30.VIII.2012, N. Kikuchi leg. (NIAES); PT ♂ of *Enicospilus kikuchii*, Eboshi-dake, Kagoshima Pref., Kyûshû, JAPAN, 4.V.1969, K. Kusigemati leg. (SEHU-KUSIG).

##### Distribution.

Eastern Palaearctic region (Shimizu 2017; present study).

JAPAN: [Hokuriku] Fukui*; [Kantô-Kôshin] Tochigi* and Saitama (Shimizu 2017; present study); [Shikoku] Ehime* and Kôchi*; [Kyûshû] Kagoshima (Shimizu 2017; present study). *New records.

##### Bionomics.

Unknown.

##### Differential diagnosis.

**Figure 22.**
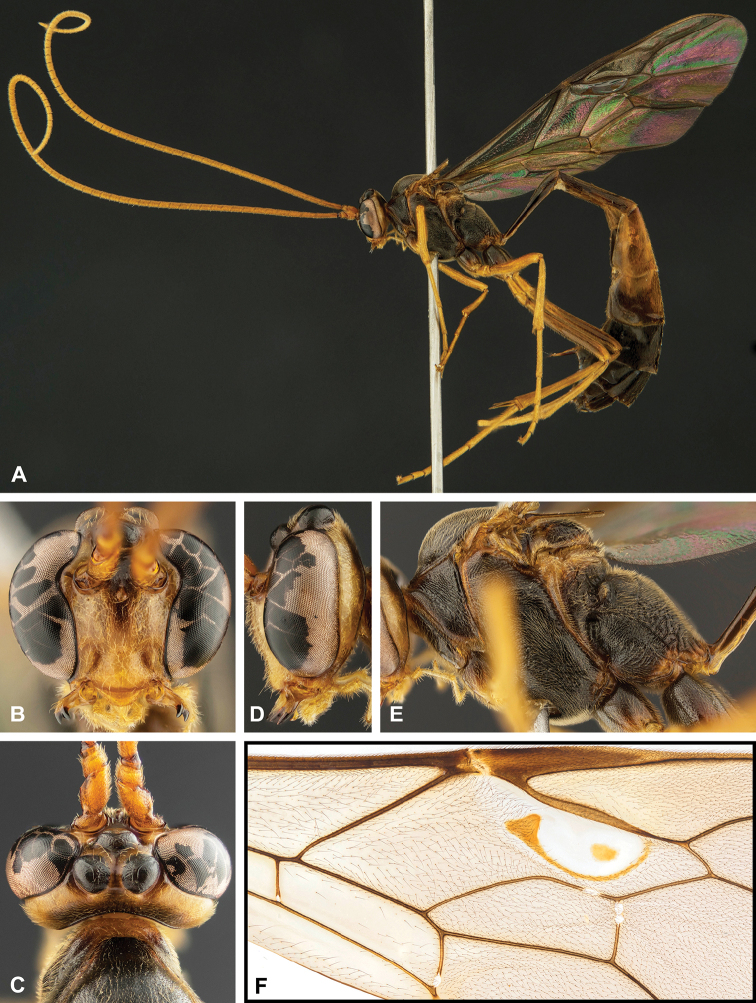
*Enicospilus kikuchii* Shimizu, 2017 ♀ from Japan **A** habitus **B** head, frontal view **C** head, dorsal view **D** head, lateral view **E** mesosoma, lateral view **F** central part of fore wing.

As in Fig. 22, the characteristic colour pattern easily distinguishes this species from other *Enicospilus* species. Morphologically, *E. kikuchii* resembles *E. melanocarpus*, but is distinguishable by the following combination of character states: mesosoma, T1, T2, and T5–8 black (Fig. 22A, E) (usually most of body yellow-brown with black T5–8 in *E. melanocarpus*, as in Fig. 28A, E); and metapleuron roughly diagonally punctostrigose (Fig. 22E) (uniformly punctate or finely diagonally punctostriate in *E. melanocarpus*, as in Fig. 28E).

#### Enicospilus kunigamiensis

Taxon classificationAnimaliaHymenopteraIchneumonidae

Shimizu sp. nov.

65CCBAF6-A844-551B-AFF5-5716677F07CF

http://zoobank.org/071D94B5-5B8D-4F25-B581-61A968A0911D

Figure 23

##### Etymology.

The specific name is derived from the type locality.

##### Type series.

A holotype female only.

HT: ♀, Benoki, Kunigami Vil., Kunigami County, Okinawa-hontô, Okinawa Pref., Ryûkyûs, JAPAN, 9.IV.1996, M. Aoyagi leg. (LT) (NIAES).

##### Distribution.

Oriental region.

JAPAN: [Ryûkyûs] Okinawa.

##### Bionomics.

Unknown.

##### Differential diagnosis.

This species is similar to *E. matsumurai* sp. nov. but easily distinguishable by the number of antennal flagellomeres (i.e., antenna with 54 flagellomeres in *E. kunigamiensis* sp. nov. but 59–60 in *E. matsumurai* sp. nov.), value of ICI (i.e., ICI = 0.4 in *E. kunigamiensis* sp. nov. but 0.7–0.8 in *E. matsumurai* sp. nov.), length of lateral longitudinal carinae of scutellum (i.e., lateral longitudinal carinae along anterior 0.2 of scutellum in *E. kunigamiensis* sp. nov. but more than 0.8 in *E. matsumurai* sp. nov.), body size (body length ca. 23.0 mm in *E. kunigamiensis* sp. nov. but 28.0–30.5 mm in *E. matsumurai* sp. nov.), etc. However, the detailed affinities of *E. kunigamiensis* sp. nov. are unknown.

##### Description.

Female (HT). Body length ca. 23.0 mm.

**Figure 23.**
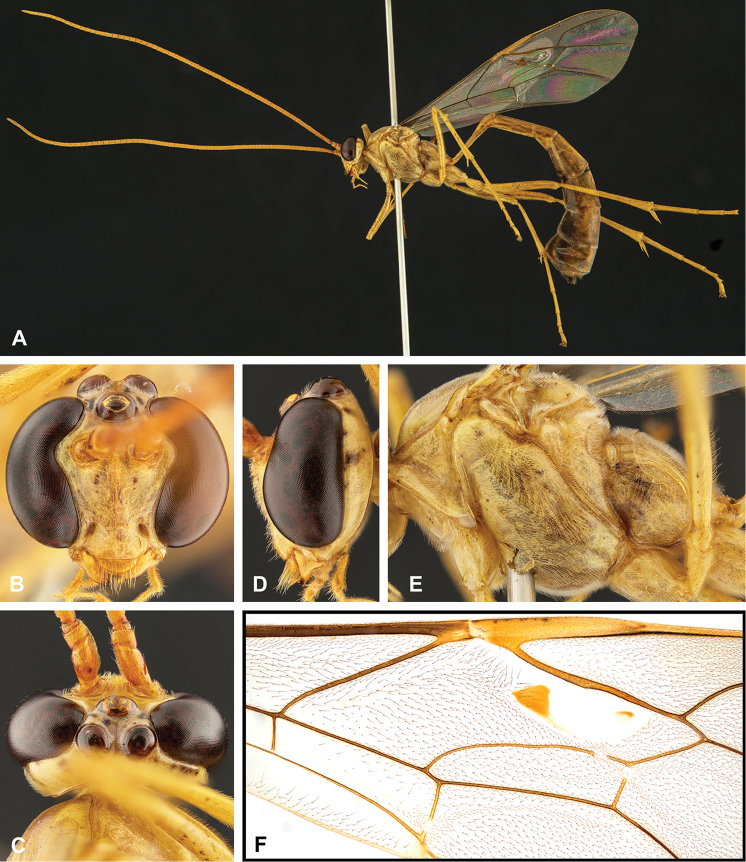
*Enicospilus kunigamiensis* Shimizu, sp. nov. ♀ (HT) from Japan **A** habitus **B** head, frontal view **C** head, dorsal view **D** head lateral view **E** mesosoma, lateral view **F** central part of fore wing.

Head with GOI = 2.5 (Fig. 23D). Lower face 0.7× as wide as high, strongly shiny, and smooth with fine setae (Fig. 23B). Clypeus 1.4× as wide as high, smooth, moderately convex in profile, and ventral margin acute (Fig. 23B, D). Malar space 0.2× as long as basal mandibular width (Fig. 23B, D). Mandible twisted by ca. 30°, moderately long, evenly rather strongly narrowed, outer surface smooth (Fig. 23B, D). Upper tooth of mandible 1.4× as long as lower. Frons, vertex and gena strongly shiny with fine setae (Fig. 23B–D). Posterior ocellus almost touching eye (Fig. 23B–D). Ventral end of occipital carina not joining oral carina. Antennae with 54 flagellomeres; first flagellomere 1.9× as long as second; 20^th^ flagellomere 1.7× as long as wide.

Mesosoma entirely strongly shiny with setae (Fig. 23E). Pronotum finely diagonally striate (Fig. 23E). Mesoscutum 1.4× as long as maximum width, strongly shiny and finely punctate with setae, and evenly rounded in profile (Fig. 23E). Notauli absent (Fig. 23E). Scutellum moderately convex, rather weakly shiny, punctate, with lateral longitudinal carinae along anterior 0.2 of scutellum. Epicnemium densely punctate with setae. Epicnemial carina present, almost straight and inclined to anterior, dorsal end not reaching anterior margin of mesopleuron (Fig. 23E). Dorsal part of mesopleuron smooth to finely punctate and ventral part longitudinally finely punctostriate (Fig. 23E). Submetapleural carina broadened anteriorly (Fig. 23E). Metapleuron moderately diagonally striate (Fig. 23E). Propodeum declivous in profile; anterior transverse carina complete; anterior area longitudinally striate; spiracular area smooth; posterior area moderately rugose; propodeal spiracle elliptical and not joining pleural carina by ridge (Fig. 23E).

Wings (Fig. 23F). Fore wing length ca. 16.0 mm with AI = 0.7, CI = 0.3, DI = 0.3, ICI = 0.4, SDI = 1.1, SI = 0.1, SRI = 0.3; vein 1m-cu&M almost evenly curved; vein 2r&RS almost straight; vein RS rather evenly curved; fenestra and sclerites of discosubmarginal cell as in Fig. 23F; proximal sclerite triangular, faintly confluent with distal sclerite, strongly pigmented; central sclerite not well-delimited and pigmented part suboval, positioned in anterodistal part of fenestra; distal sclerite vestigial; proximal corner of marginal cell evenly setose; posterodistal corner of second discal cell ca. 100° and of subbasal cell ca. 70°; vein 1cu-a antefurcal to M&RS by 0.1× length of 1cu-a. Hind wing with NI = 2.3; vein RS straight; vein RA with 7 uniform hamuli.

Legs. Hind leg with coxa in profile 1.8× as long as deep; basitarsus 1.9× as long as second tarsomere; fourth tarsomere 3.1× as long as wide; tarsal claw simply pectinate.

Metasoma with DMI = 1.4, PI = 3.2, THI = 3.5; thyridium oval; ovipositor sheath not longer than posterior depth of metasoma (Fig. 23A).

Colour (Fig. 23). Longitudinal vittae of mesoscutum and metasoma red-brown; apex of mandible black, otherwise whitish yellow. Wings weakly infuscate; sclerites pigmented and amber; vein dark reddish brown.

Male. Unknown.

#### Enicospilus laqueatus

Taxon classificationAnimaliaHymenopteraIchneumonidae

(Enderlein, 1921)

B243E3E5-F8A7-5101-8B99-F1687E317EE7

Figure 24

Henicospilus laqueatus Enderlein, 1921: 26; HT ♂ from Taiwan, IZPAN, not examined.
Enicospilus leetoni Chiu, 1954: 38; HT ♀ from Taiwan, TARI, examined; synonymised by Gauld and Mitchell (1981: 396).

##### Specimens examined.

Total of 76 specimens (48♀♀26♂♂ and 2 unsexed): India (2♀♀1♂), Japan (22♀♀23♂♂), Taiwan (23♀♀2♂♂ and 2 unsexed), Zambia (1♀).

Type series: HT ♀ of *Enicospilus leetoni* Chiu, 1954, Taihoku, TAIWAN, 1.IX.1925, J. Sonan leg. (TARI).

##### Distribution.

Afrotropical and Oriental regions (Yu et al. 2016); new to the Eastern Palaearctic (Hachijô-jima, Tôkyô, Kantô-Kôshin, Japan) and Oceanic (Nishi-jima, Tôkyô, Ogasawara, Japan) regions (cf. Suppl. material 1: Table S1); this is a predominantly (sub-)tropical species. This species is widely distributed from the Afrotropical to Oriental regions and Gauld (1982) suggested that it has possibly been introduced from Asia to Africa, although there is no reliable evidence to support or refute his hypotheses.

Newly recorded from Japan.

JAPAN: [Kantô-Kôshin] Tôkyô; [Ryûkyûs] Kagoshima and Okinawa; [Ogasawara] Tôkyô. This species is restricted to southern regions of Japan.

##### Bionomics.

Unknown.

##### Differential diagnosis.

**Figure 24.**
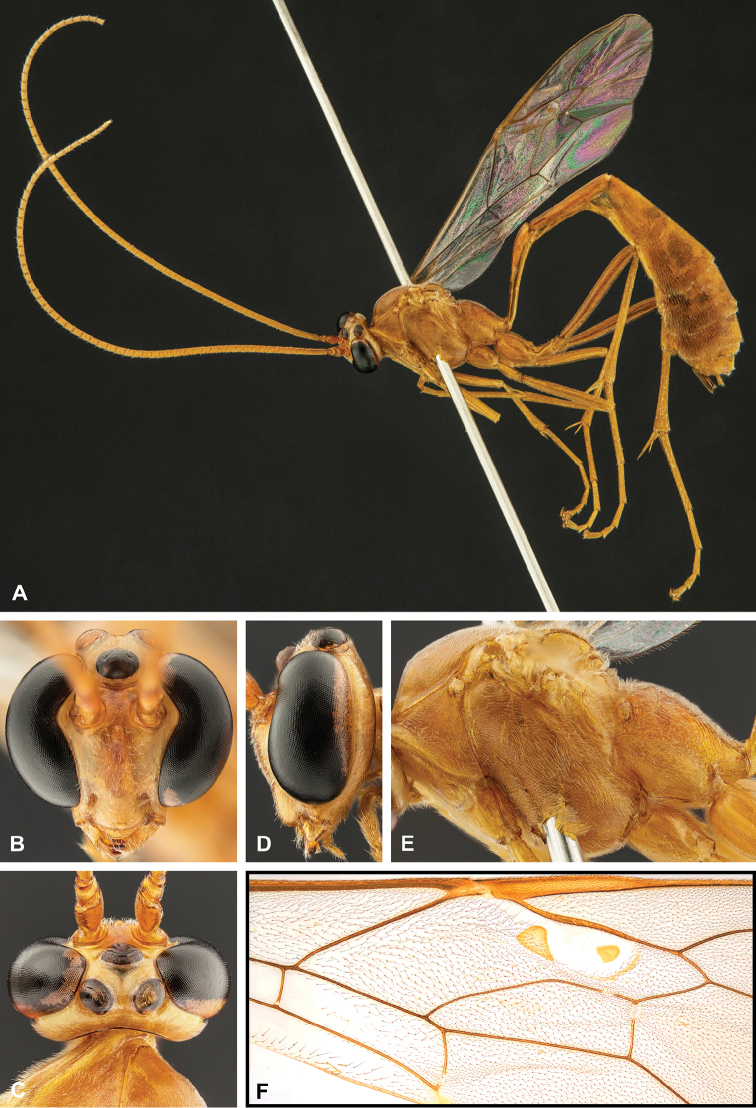
*Enicospilus laqueatus* (Enderlein, 1921) ♀ from Japan **A** habitus **B** head, frontal view **C** head, dorsal view **D** head, lateral view **E** mesosoma, lateral view **F** central part of fore wing.

This species resembles *E. pseudantennatus*, *E. vestigator*, and *E. tripartitus* on the shape of fore wing fenestra, sclerites, and venation. However, *E. laqueatus* is easily distinguishable from them by the outer mandibular surface morphology (i.e., outer mandibular surface with diagonal setose groove between dorsoproximal corner and base of apical teeth in *E. laqueatus*, but smooth or just densely punctate with stout setae in the other three species, as summarised in Table 6). In addition, this species morphologically resembles *E. aciculatus* and *E. yonezawanus* but is distinguished from them by its strongly pigmented central sclerite of fore wing fenestra (Fig. 24F) (central sclerite very weakly pigmented or vestigial in *E. aciculatus*, as in Fig. 10F, and completely lacking in *E. yonezawanus*, as in Fig. 54F).

**Table 6.** Comparison of diagnostic characters of *Enicospilus* species in Japan that share the triangular fore wing proximal sclerite, larger and strongly pigmented central sclerite, entirely testaceous body, and similar shape of fore wing veins: *E. laqueatus*, *E. tripartitus* and *E. vestigator*.

Characters	Species		
	* laqueatus *	* tripartitus *	* vestigator *
Lower margin of clypeus	acute	blunt and impressed	blunt
Torsion of mandible	10–15°	10–12°	60–80°
Outer surface of mandible	with a diagonal groove	covered with dence and long setae	smooth
Basal concavity of mandibular outer surface	shallow	deep	shallow
Discosubmarginal cell	rather short	rather short	long
ICI	0.4–0.5	0.5–0.7	0.6–0.9

#### Enicospilus limnophilus

Taxon classificationAnimaliaHymenopteraIchneumonidae

Shimizu sp. nov.

452AE17D-31B0-519B-865A-2AA900DAAF76

http://zoobank.org/2383AAC6-A428-4933-A7DC-50400B672084

Figure 25

##### Etymology.

This species probably prefers lakes. Hence, the specific name is derived from the Greek *limne* + *philos* meaning lake and lover respectively.

##### Type series.

Total of 37 specimens (18♀♀19♂♂): Japan (18♀♀19♂♂).

HT: ♀, marsh of Nakaikemi, Tsuruga City, Fukui Pref., Hokuriku, JAPAN, 19.IX–16.X.2016, A. Noishiki leg. (MsT) (MNHA).

PT: 1♀, Toro-ko, Kushiro City, Hokkaidô, JAPAN, 26.VII.1986, S. Nomura leg. (NIAES); 1♀, Kushiro-shitsugen, Kushiro City, Hokkaidô, JAPAN, 29.VII.1991, Y. Karasawa leg. (LT) (NIAES); 1♀, Sapporo, Hokkaidô, JAPAN, 9.VIII.1960, S. Ueda leg. (MNHA); 9♀♀5♂♂, same data as HT (3♀♀1♂, MNHA; 2♀♀, OMNH; 1♀1♂, KPMNH; 1♀, NHMUK; 1♀2♂♂, EUM; 1♀1♂, SEHU); 1♀, same data as HT except for 31.VII–14.VIII.2016 (NIAES); 1♀11♂♂, same data as HT except for 14–30.VIII.2016 (3♂♂, NIAES; 3♂♂, NSMT; 1♂, EMUS; 1♀2♂♂, MNHA; 2♂♂, SEHU); 2♀♀1♂, same data as HT except for 10–31.VII.2016 (1♀1♂, OMNH; 1♀, CNC); 1♀, same data as HT except for 24.V–17.VI.2016 (EMUS); 2♂♂, same data as HT except for 17.VI–10.VII.2016 (1♂, CNC; 1♂, NHMUK).

##### Distribution.

Eastern Palaearctic region.

JAPAN: [Hokkaidô]; [Hokuriku] Fukui.

##### Bionomics.

All specimens have been collected from marshes or lakes of rather cooler regions, suggesting that it is restricted to hosts that inhabit open, aquatic conditions. However, some factors, such as a progression of plant succession, isolation of habitats, and increasingly dry conditions, have led many wetland insects to become endangered in Japan (e.g., Yoshida et al. 2019).

##### Differential diagnosis.

**Figure 25.**
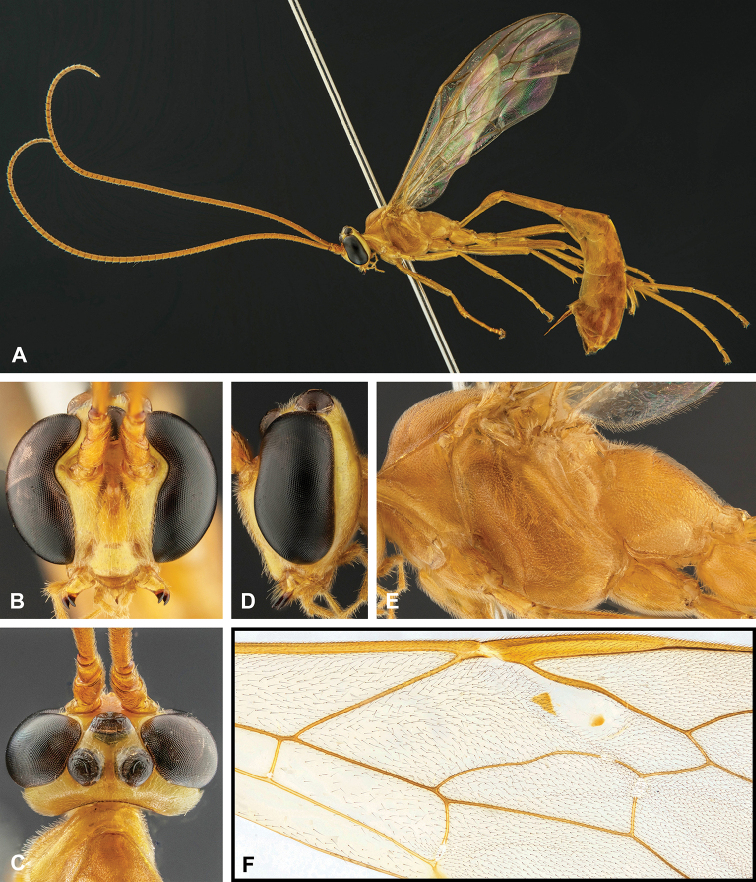
*Enicospilus limnophilus* Shimizu, sp. nov. ♀ (**A–E**HT**F**PT) from Japan **A** habitus **B** head, frontal view **C** head, dorsal view **D** head, lateral view **E** mesosoma, lateral view **F** central part of fore wing.

The distally setose fore wing fenestra is unique to this species within the Asian *Enicospilus* fauna (Fig. 25F), hence *E. limnophilus* sp. nov. is morphologically very easily recognisable.

##### Description.

Female (n = 18). Body length 15.0–18.0 (HT: ca. 17.5) mm.

Head with GOI = 1.9–2.9 (HT: 2.8) (Fig. 25D). Lower face 0.7–0.8× (HT: 0.8) as wide as high, moderately shiny, and entirely punctate with setae (Fig. 25B). Clypeus 1.5–1.7× (HT: 1.7) as wide as high, smooth with setae, weakly convex, and ventral margin impressed (Fig. 25B, D). Malar space 0.3× as long as basal mandibular width (Fig. 25B, D). Mandible twisted by 35–40° (HT: 40°), rather short, evenly narrowed, outer surface with diagonal setose groove (Fig. 25B, D). Upper tooth of mandible 1.4–1.6× (HT: 1.4) as long as lower (Fig. 25B). Frons, vertex and gena shiny with setae (Fig. 25B–D). Posterior ocellus almost touching eye (Fig. 25B–D). Ventral end of occipital carina joining oral carina. Antennae with 58–62 (HT: 61) flagellomeres; first flagellomere 1.7–1.8× (HT: 1.8) as long as second; 20^th^ flagellomere 1.7–1.8× (HT: 1.7) as long as wide.

Mesosoma entirely weakly to moderately shiny with setae (Fig. 25E). Pronotum finely punctate to diagonally striate (Fig. 25E). Mesoscutum 1.5× as long as maximum width, moderately shiny and punctate with setae, and evenly rounded in profile (Fig. 25E). Notauli absent (Fig. 25E). Scutellum moderately convex, smooth and strongly shiny, with lateral longitudinal carinae along anterior 0.1–1.0 of scutellum. Epicnemium densely punctate with setae. Epicnemial carina present, evenly slightly curved, inclined to anterior, dorsal end distinctly separated from anterior margin of mesopleuron (Fig. 25E). Mesopleuron densely punctate to punctostriate (Fig. 25E). Submetapleural carina broadened anteriorly (Fig. 25E). Metapleuron entirely very densely punctate, (sub-)matt (Fig. 25E). Propodeum evenly rounded in profile; anterior transverse carina complete centrally and absent laterally; anterior area longitudinally striate; spiracular and posterior areas entirely densely punctate to finely reticulate; propodeal spiracle elliptical and joining pleural carina by strong ridge (Fig. 25E).

Wings (Fig. 25F). Fore wing length 10.5–11.0 (HT: 11.0) mm with AI = 0.9–1.0 (HT: 0.9), CI = 0.2, DI = 0.4, ICI = 0.6, SDI = 1.2, SI = 0.1, SRI = 0.3; vein 1m-cu&M sinuous; vein 2r&RS slightly sinuous; vein RS evenly curved; fenestra and sclerites of discosubmarginal cell as in Fig. 25F; proximal sclerite triangular and strongly sclerotised; central sclerite moderately large, roundish, strongly sclerotised gradually from proximal to distal, and positioned in around centrodistal part of fenestra; distal sclerite vestigial and slightly pigmented; distal part of fenestra between central and distal sclerites setose; proximal corner of marginal cell evenly setose; posterodistal corner of second discal cell ca. 85° and of subbasal cell nearly at right angle; vein 1cu-a antefurcal to M&RS by 0.3–0.4× (HT: 0.4) length of 1cu-a. Hind wing with NI = 1.5–1.6 (HT: 1.5); vein RS straight; vein RA with 5–6 (HT: 5) uniform hamuli.

Legs. Hind leg with coxa in profile 1.7–1.9× (HT: 1.7) as long as deep; basitarsus 2.0× as long as second tarsomere; fourth tarsomere 3.1–4.2× (HT: 4.2) as long as wide; tarsal claw simply pectinate.

Metasoma with DMI = 1.4, PI = 2.9–3.1 (HT: 3.1), THI = 3.5; thyridium elongate oval; ovipositor sheath not longer than posterior depth of metasoma (Fig. 25A).

Colour (Fig. 25). Entirely reddish yellow but head lighter than meso- and metasoma, apex of mandible infuscate. Wings hyaline; proximal sclerite pigmented and amber; veins brown.

Male (n = 19). Very similar to female.

##### Remarks.

This is a fairly morphologically uniform species, although GOI (= 1.9–2.9) and length of lateral longitudinal carinae of scutellum (along anterior 0.1–0.9 of scutellum) exhibit a very wide range of variation within the same population.

#### Enicospilus maruyamanus

Taxon classificationAnimaliaHymenopteraIchneumonidae

(Uchida, 1928)

CD3844D3-D4B3-5422-9625-DD2382FFE6DE

Figure 26

Henicospilus maruyamanus Uchida, 1928: 220; LCT ♀ from Japan, designated by Townes et al. (1965: 329), SEHU, examined.

##### Specimens examined.

Total of 51 specimens (42♀♀9♂♂): India (2♀♀), Japan (40♀♀8♂♂), Philippines (1♂).

Type series: LCT ♀ of *Henicospilus maruyamanus* Uchida, 1928, Maruyama, Sapporo, Hokkaidô, JAPAN, 11.VIII.1926, Uchida leg. (SEHU).

##### Distribution.

Eastern Palaearctic region (Yu et al. 2016); new to the Oriental region, this is a predominantly Eastern Palaearctic species.

Newly recorded from India and Philippines.

JAPAN: [Hokkaidô] (Uchida 1928; present study); [Tôhoku] Aomori (Ichita 1994; Kudo et al. 1999); [Hokuriku] Niigata*; [Kantô-Kôshin] Tochigi*, Nagano*, and Kanagawa (Watanabe et al. 2016); [Chûgoku] Hiroshima*; [Shikoku] Ehime* and Kôchi*; [Kyûshû] Fukuoka* and Saga*; [Ryûkyûs] Okinawa*. *New records.

##### Bionomics.

Unknown.

##### Differential diagnosis.

**Figure 26.**
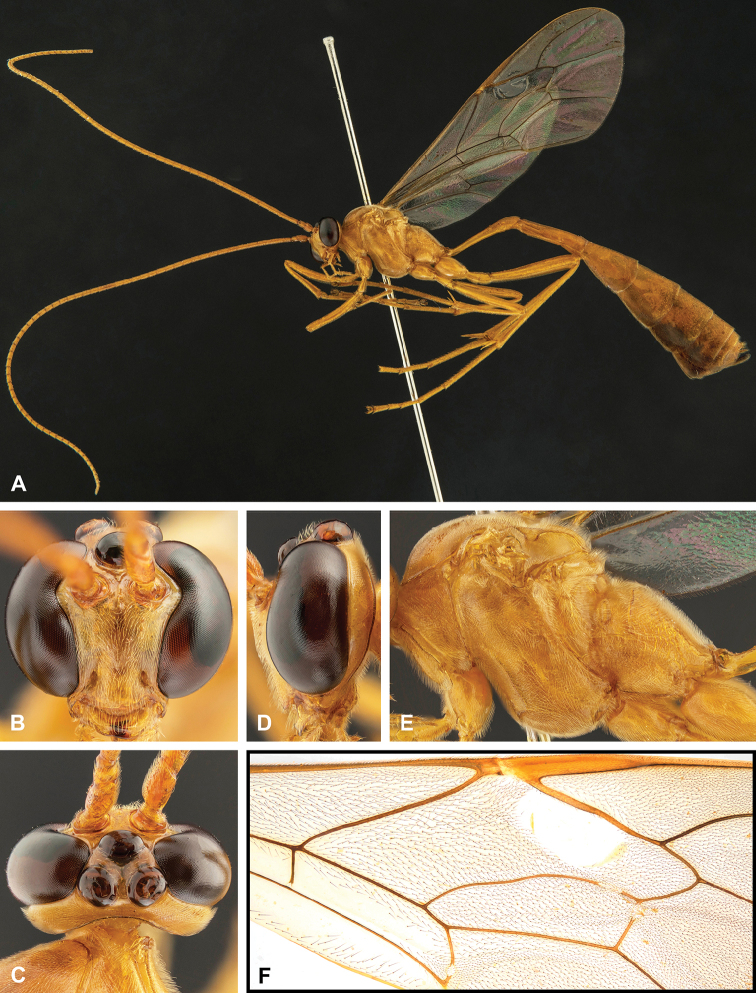
*Enicospilus maruyamanus* (Uchida, 1928) ♀ from Japan **A** habitus **B** head, frontal view **C** head, dorsal view **D** head, lateral view **E** mesosoma, lateral view **F** central part of fore wing.

This species resembles *E. biharensis*, *E. pudibundae*, and *E. transversus*, but can be distinguished from them by the following combination of character states: uniformly pectinate hind tarsal claw; weakly to moderately sinuate fore wing vein 1m-cu&M (Fig. 26F); and punctostriate meso- and metapleuron (Fig. 26E) (cf. Table 7).

**Table 7.** Comparison of diagnostic characters of *Enicospilus* species in Japan and its adjacent areas that share the narrow and linear fore wing proximal sclerite without central sclerite: E. biharensis, *E. maruyamanus*, *E. pudibundae*, and *E. transversus*.

Characters	Species			
	* biharensis *	* maruyamanus *	* pudibundae *	* transversus *
Sculpture of meso- and metasoma	roughly punctate to punctostriate	punctostriate	punctate	striate
Fore wing vein 1m-cu&M	evenly curved	weakly sinuous	evenly curved	weakly sinuous
CI	<0.5	≥ 0.5	> 0.5	≥0.5
Pectinae of hind tarsal claw	complete	complete	lacking proximally	complete

#### Enicospilus matsumurai

Taxon classificationAnimaliaHymenopteraIchneumonidae

Shimizu sp. nov.

B13DF4CF-E4EB-5828-8624-7C38DB2A3468

http://zoobank.org/DF52C899-7459-4CC9-8C1C-A2A2FBD46170

Figure 27

##### Etymology.

The specific name is derived from one of the greatest Japanese entomologists, Shônen Matsumura.

##### Type series.

Total of 12 specimens (8♀♀4♂♂): Japan (8♀♀4♂♂).

HT: ♀, Mt. Hikosan, Fukuoka Pref., Kyûshû, JAPAN, 22.IX.1983, M.T. Chûjô leg. (LT) (NIAES).

PT: 1♀, Tarumi, Matsuyama, Ehime Pref., Shikoku, JAPAN, 18.IX.1948, M. Miyatake leg. (EUM); 3♀♀, Matsuyama, Ehime Pref., Shikoku, JAPAN, 18.X.1954 (1♀), 19.X.1954 (1♀), 26.XI.1954 (1♀), S. Ueda leg. (EUM); 1♂, Matsuyama, Ehime Pref., Shikoku, JAPAN, 13.I.1952, T. Ishikawa leg. (EUM); 1♀, Teragawa, Hongawa, Kôchi Pref., Shikoku, JAPAN, 22.VII.1989, I. Yamashita leg. (LT) (NIAES); 1♀, Jigokudani, Kôchi City, Kôchi Pref., Shikoku, JAPAN, 7.VIII.1992, I. Yamashita leg. (LT) (NIAES);1♀, Kôchi Pref., Shikoku, JAPAN (MNHA); 1♂, Mt. Hikosan, Fukuoka Pref., Kyûshû, JAPAN, 11.X.1980, K. Konishi leg. (LT) (NIAES); 1♂, Nagasaki Pref., Kyûshû, JAPAN, V.1935 (NIAES-TIC); 1♂, Mt. Kurodake, Mts. Kujû, Ōita Pref., Kyûshû, JAPAN, 3.X.1983, N. Koda & R. Noda leg. (LT) (NIAES).

##### Distribution.

Eastern Palaearctic region.

JAPAN: [Shikoku] Ehime and Kôchi; [Kyûshû] Fukuoka, Nagasaki and Ōita.

##### Bionomics.

Unknown.

##### Differential diagnosis.

**Figure 27.**
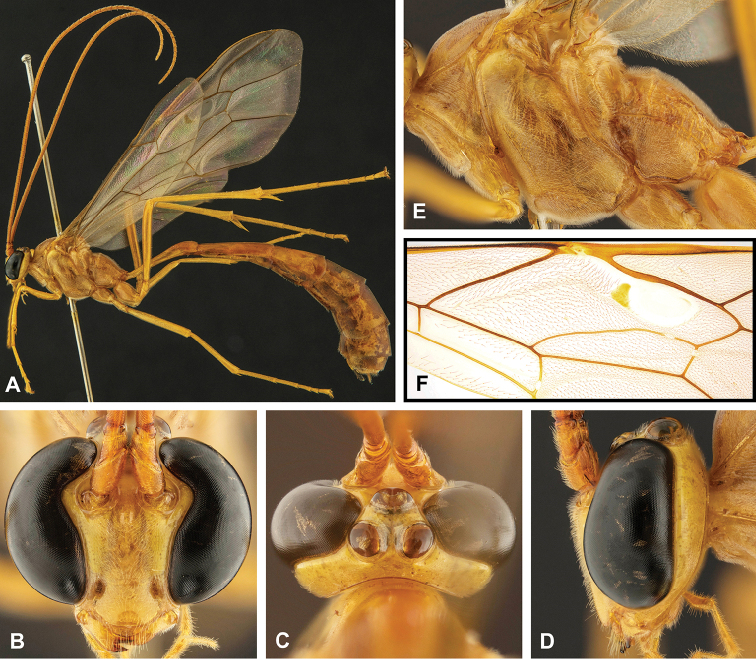
*Enicospilus matsumurai* Shimizu, sp. nov. ♀ (HT) from Japan **A** habitus **B** head, frontal view **C** head, dorsal view **D** head, lateral view **E** mesosoma, lateral view **F** central part of fore wing.

This species is similar to *E. flavicaput* and some of the type series were misidentified as that species, but *E. matsumurai* sp. nov. is distinguishable from *E. flavicaput* by the shape and position of the central sclerite of the fore wing fenestra (Fig. 27F), sculpture of the mesosoma (Fig. 27E), and wing venation (Fig. 27F). This species also resembles *E. kunigamiensis* sp. nov. but is distinguishable by several characters (cf. ‘Differential diagnosis’ under *E. kunigamiensis* sp. nov.). In addition, the large size of this species is also a useful diagnostic character.

##### Description.

Female (n = 8). Body length 28.0–30.5 (HT: ca. 30.5) mm.

Head with GOI = 2.5–2.9 (HT: 2.8) (Fig. 27D). Lower face 0.7× as wide as high, strongly shiny, finely punctate with setae (Fig. 27B). Clypeus 1.3–1.5× (HT: 1.5) as wide as high, sparsely finely punctate with setae, very weakly convex but almost flat in profile, and ventral margin subacute (Fig. 27B, D). Malar space 0.2× as long as basal mandibular width (Fig. 27B, D). Mandible moderately twisted by 50–55° (HT: ca. 50°), moderately long, evenly narrowed, outer surface smooth (Fig. 27B, D). Upper tooth of mandible 1.3–1.4× (HT: 1.4) as long as lower (Fig. 27B). Frons, vertex and gena strongly shiny with fine setae. Posterior ocellus touching eye (Fig. 27B–D). Ventral end of occipital carina joining oral carina. Antennae with 59–60 (HT: 60) flagellomeres; first flagellomere 2.0× as long as second; 20^th^ flagellomere 1.7–1.8× (HT: 1.7) as long as wide.

Mesosoma entirely weakly to moderately shiny with setae (Fig. 27E). Pronotum diagonally striate (Fig. 27E). Mesoscutum 1.4× as long as maximum width, moderately shiny and finely punctate to smooth with setae, and evenly rounded in profile (Fig. 27E). Notauli absent (Fig. 27E). Scutellum moderately convex, smooth, with lateral longitudinal carinae along anterior 0.8–1.0 of scutellum. Epicnemium densely punctate with setae. Epicnemial carina strong, straight, strongly inclined to anterior, dorsal end not reaching anterior margin of mesopleuron (Fig. 27E). Mesopleuron entirely longitudinally striate (Fig. 27E). Submetapleural carina broadened anteriorly (Fig. 27E). Metapleuron densely punctate with rather coarse diagonal striae (Fig. 27E). Propodeum evenly rounded in profile; anterior transverse carina complete; anterior area longitudinally striate; spiracular area smooth; posterior area coarsely rugose; propodeal spiracle elliptical and not joining pleural carina by ridge (Fig. 27E).

Wings (Fig. 27F). Fore wing length 19.5–21.5 (HT: ca. 21.0) mm with AI = 0.7–0.8 (HT: 0.8), CI = 0.2–0.3 (HT: 0.2), DI = 0.3, ICI = 0.7–0.8 (HT: 0.8), SDI = 1.2–1.3 (HT: 1.3), SI = 0.1, SRI = 0.2; vein 1m-cu&M slightly sinuous; vein 2r&RS slightly sinuous and angulate; vein RS evenly curved; fenestra and sclerites of discosubmarginal cell as in Fig. 27F; proximal sclerite subquadrate, not confluent with distal sclerite, strongly pigmented; central sclerite linear and rather weakly pigmented, subparallel to 2r&RS, positioned in anterodistal part of fenestra; distal sclerite vestigial or absent; proximal corner of marginal cell evenly setose; posterodistal corner of second discal cell 100–110° (HT: ca. 110°) and of subbasal cell 80–85° (HT: ca. 85°); vein 1cu-a antefurcal to M&RS by 0.2× length of 1cu-a. Hind wing with NI = 2.6–2.9 (HT: 2.6); vein RS basally slightly bowed and slightly evenly curved; vein RA with 8–9 (HT: 9) uniform hamuli.

Legs. Hind leg with coxa in profile 1.7–1.8× (HT: 1.7) as long as deep; basitarsus 1.8–1.9× (HT: 1.9) as long as second tarsomere; fourth tarsomere 2.8–3.3× (HT: 2.8) as long as wide; tarsal claw simply pectinate.

Metasoma with DMI = 1.3–1.4 (HT: 1.4), PI = 3.1–3.5 (HT: 3.1), THI = 4.3–4.7 (HT: 4.7); thyridium oval, rather small, not strongly impressed; ovipositor sheath not longer than posterior depth of metasoma (Fig. 27A).

Colour (Fig. 27). Entirely red-brown except for apex of mandible black. Wings weakly infuscate; sclerites pigmented and amber; veins red-brown.

Male (n = 4). Very similar to female except body length 22.0–30.5 mm; antennae with 55–63 flagellomeres.

#### Enicospilus melanocarpus

Taxon classificationAnimaliaHymenopteraIchneumonidae

Cameron, 1905

F813812E-6CA1-536F-A4A6-667D2FB32F7E

Figure 28

Enicospilus reticulatus Cameron, 1902: 52; HT ♂ from Maldive Islands, NHMUK, examined; synonymised by Gauld and Mitchell (1981: 377); junior primary homonym of Enicospilus reticulatus Cameron, 1899.
Eniscospilus (sic) melanocarpus Cameron, 1905a: 122; HT ♀ from Sri Lanka, NHMUK, examined.
Henicospilus nigrinervis Szépligeti, 1906: 142; HT ♀ from New Guinea, TM, not examined; synonymised by Gauld and Mitchell (1981: 377); junior secondary homonym of Enicospilus nigrinervis Cameron, 1901.
Ophion (Henicospilus) nocturnus Kohl, 1908: 315; HT ♀ from Samoa, NM, not examined; synonymised by Gauld and Mitchell (1981: 378).
Henicospilus batavianus Szépligeti, 1910: 92; HT ♀ from Java, TM, not examined; synonymised by Gauld and Mitchell (1981: 378).
Henicospilus turneri Morley, 1912: 51; LCT ♀ from Australia, designated by Townes et al. (1961: 291), NHMUK, examined; synonymised by Gauld and Mitchell (1981: 378).
Henicospilus atricornis var. zeylanicus Morley, 1913: 392; HT ♀ from Sri Lanka, NHMUK, examined; synonymised by Gauld and Mitchell (1981: 378).
Henicospilus uncivena Enderlein, 1921: 23; HT ♀ from India, IZPAN, not examined; synonymised by Gauld and Mitchell (1981: 378).
Henicospilus crassivena Enderlein, 1921: 24; HT ♀ from Sumatra, IZPAN, not examined; synonymised by Townes et al. (1961: 281).
Enicospilus nigrivenalis Cushman, 1937: 307; HT ♀ from Taiwan, DEI, not examined; synonymised by Gauld and Mitchell (1981: 378).
Enicospilus quintuplex Chiu, 1954: 61; HT ♀ from China, TARI, examined; synonymised by Gauld and Mitchell (1981: 378).
Enicospilus (Polycorniata) brunnis Rao and Nikam, 1971: 105; HT ♀ from India, MUC, not examined; synonymised by Gauld and Mitchell (1981: 378).

##### Specimens examined.

Total of 217 specimens (166♀♀41♂♂ and 10 unsexed): Australia (1♀), China (1♀), India (26♀♀), Indonesia (4♀♀2♂♂ and 1 unsexed), Japan (26♀♀12♂♂ and 1 unsexed), Malaysia (1♀), Maldives (1♂), Papua New Guinea (7♀♀1♂), Philippines (7♀♀), Singapore (1 unsexed), Sri Lanka (8♀♀), Taiwan (85♀♀25♂♂ and 7 unsexed).

Type series: HT ♂ of *Enicospilus reticulatus* Cameron, 1902, Hulule, MALDIVE, 20.VI.1900 (NHMUK, Type 3b.1268); HT ♀ of *Eniscospilus* (sic) *melanocarpus* Cameron, 1905, SRI LANKA (NHMUK, Type 3b.1234); LCT ♀ of *Henicospilus turneri* Morley, 1912, Mackay, Queensland, AUSTRALIA, 1899, Turner leg. (NHMUK, Type 3b.1261); HT ♀ of Henicospilus atricornis var zeylanicus Morley, 1913, Kandy, SRI LANKA, 11.VII.1910, Green leg. (NHMUK, Type 3b.2098); HT ♀ of *Enicospilus quintuplex* Chiu, 1954, Shaowu, Fukien, CHINA, 8.X.1945, S.H. Chao leg. (TARI).

##### Distribution.

Australasian, Eastern Palaearctic, Oceanic, and Oriental regions (Yu et al. 2016); this is a predominantly Oriental species.

JAPAN: [Ryûkyûs] Kagoshima* and Okinawa (Uchida 1928; Townes 1958; Shimizu 2020; present study); [Ogasawara] Tôkyô (Townes 1958; Takahashi and Shimizu 2006; present study). *New record. This is one of the most frequently encountered *Enicospilus* species in the Oriental region. Watanabe et al. (2012) and Shimizu (2014) recorded this species from Fukui and Niigata Prefectures, Hokuriku, Japan respectively, but these records were based on misidentifications of *E. ramidulus*.

##### Bionomics.

No host records from Japan. Gauld and Mitchell (1981) and Nikam (1990) report rearings from a disparate range of hosts in the families Erebidae, Lasiocampidae and Noctuidae, which clearly warrants investigation.

##### Differential diagnosis.

**Figure 28.**
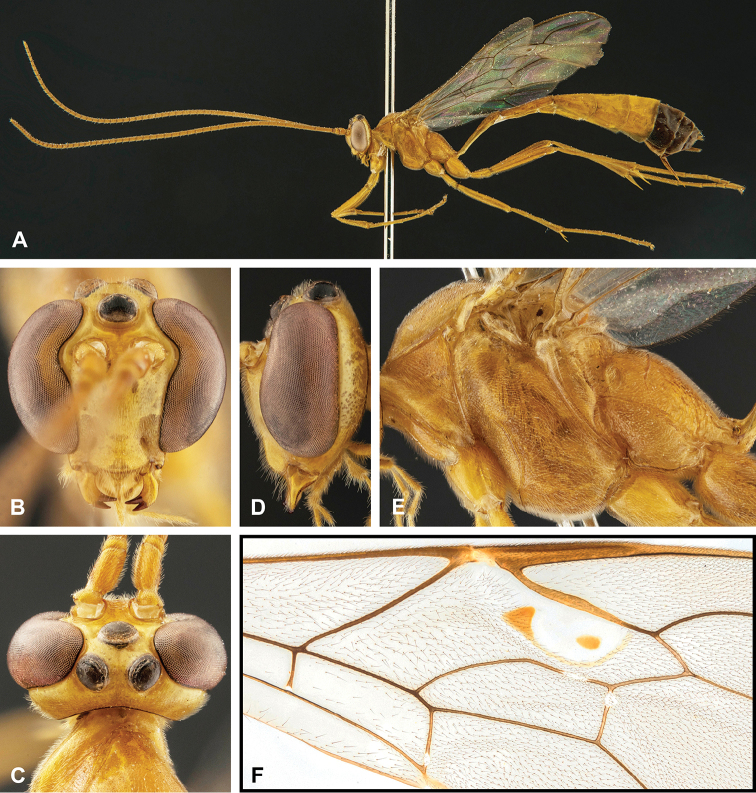
*Enicospilus melanocarpus* Cameron, 1905 ♀ from Japan **A** habitus **B** head, frontal view **C** head, dorsal view **D** head, lateral view **E** mesosoma, lateral view **F** central part of fore wing.

This species is morphologically most similar to *E. sauteri*, but is distinguished from it by the uniformly setose marginal cell of fore wing (Fig. 28F) (marginal cell proximally widely glabrous in *E. sauteri*, as in Fig. 42F), and oval central sclerite of fore wing fenestra (Fig. 28F) (central sclerite linear in *E. sauteri*, as in Fig. 42F). *Enicospilus melanocarpus* is also sometimes confused with *E. ramidulus* but is distinguished from it by the sculpture of the mesosoma (i.e., meso- and metapleuron punctate to punctostriate in *E. melanocarpus*, as in Fig. 28E, but entirely punctate in *E. ramidulus*, as in Fig. 39E), shape of the sclerites (i.e., proximal and distal sclerites confluent in *E. melanocarpus*, as in Fig. 28F, but not confluent in *E. ramidulus*, as in Fig. 39F), etc.

In Japanese collections, they are sometimes confused with *E. ramidulus* and *E. yezoensis*, as both species have a similar colour pattern (i.e., entirely testaceous body with posterior metasomal segments strongly infuscate, as in Figs 28A, 39A, and 53A). However, in Japan *E. melanocarpus* is restricted to Ryûkyûs and Ogasawara (i.e., the Oceanic and Oriental regions of Japan), with *E. ramidulus* and *E. yezoensis* in the Palaearctic area of Japan. We summarise the diagnostic characters in Table 8 for *E. melanocarpus*, *E. ramidulus*, *E. sauteri* and *E. yezoensis*, all of which have testaceous bodies with the metasoma black posteriorly.

**Table 8.** Comparison of diagnostic characters of *Enicospilus* species in Japan that share the entirely light-orange body and black terminal segments of the metasoma: *E. melanocarpus*, *E. ramidulus*, *E. sauteri*, and *E. yezoensis*.

Characters	Species			
	* melanocarpus *	* ramidulus *	* sauteri *	* yezoensis *
Lower face (width / height)	0.7–0.8	0.8–0.9	0.7–0.8	1.0
Length of tooth (upper teeth / lower teeth)	1.2–1.5	1.9–2.2	1.2–1.3	1.7–2.0
Posterior ocelli and orbit	very close	touching or very close	very close	distinctly separated
Sculpture of meso- and metapleuron	punctate to punctostriate	entirely punctate	punctate to punctostriate	punctate to punctostriate
Marginal cell of fore wing	entirely setose	entirely setose	with a glabrous	entirely setose
Shape of central sclerite	oval	oval	linear	comma-shaped
Proximal and distal sclerites	confluent	separated	confluent	separated

#### Enicospilus multidens

Taxon classificationAnimaliaHymenopteraIchneumonidae

Chiu, 1954 stat. rev.

E8056006-30A0-5ABC-B8EE-49E72475A4D8

Figure 29

Enicospilus multidens Chiu, 1954: 75; HT ♀ from Japan, TARI, examined; **stat. rev.**

##### Specimens examined.

Total of 30 specimens (14♀♀16♂♂): Japan (14♀♀16♂♂).

Type series: HT ♀ of *Enicospilus multidens* Chiu, 1954, Minoh, Ōsaka, Kinki, JAPAN, 20.VII.1918, N. Tosawa leg. (TARI).

##### Distribution.

Eastern Palaearctic region (Chiu 1954; present study); new to the Oriental region; this is a predominantly Eastern Palaearctic species.

JAPAN: [Hokkaidô]*; [Tôhoku] Aomori*; [Hokuriku] Niigata*; [Kantô-Kôshin] Gunma*, Nagano*, Yamanashi*, and Saitama*; [Tôkai] Shizuoka* and Mie*; [Kinki] Ōsaka (Chiu 1954; present study) and Hyôgo*; [Chûgoku] Tottori (Chiu 1954), Shimane* and Hiroshima*; [Shikoku] Ehime* and Kôchi*; [Kyûshû] Kumamoto*; [Ryûkyûs] Kagoshima*. *New records.

##### Bionomics.

Unknown.

##### Differential diagnosis.

**Figure 29.**
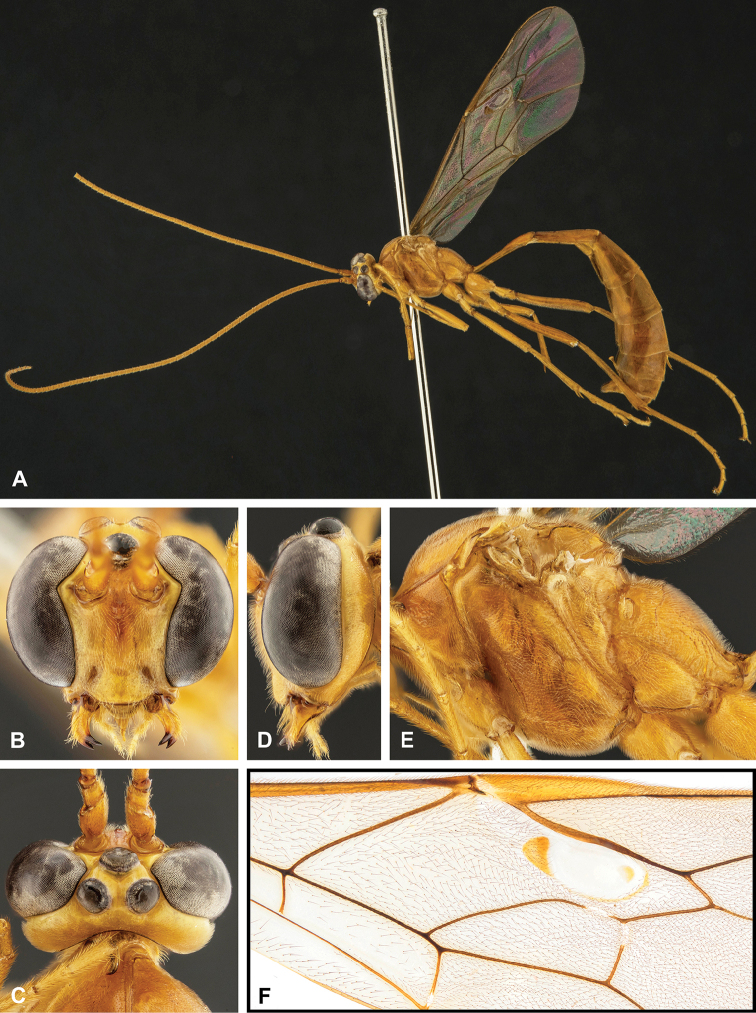
*Enicospilus multidens* Chiu, 1954, stat. rev. ♂ from Japan **A** habitus **B** head, frontal view **C** head, dorsal view **D** head, lateral view **E** mesosoma, lateral view **F** central part of fore wing.

In some ichneumonid collections, this species has been confused with *E. shikokuensis*. However, *E. multidens* stat. rev. can be distinguished from *E. shikokuensis* by shallow concavity of proximal outer mandibular surface (Fig. 29B, D) (proximal outer mandibular surface with very wide subtriangular concavity in *E. shikokuensis*, as in Fig. 44B, D); separated proximal and distal sclerites (Fig. 29F) (proximal and distal sclerites usually confluent, as in Figs 7B, 44F, or rarely separated, as in Fig. 7A, in *E. shikokuensis*); stouter, shorter, and evenly narrowed mandible (Fig. 29B, D) (mandible much longer, slender, proximally strongly narrowed, and apical 0.7 parallel-sided in *E. shikokuensis*, as in Fig. 44B, D); narrower lower face (Fig. 29B) (lower face wider in *E. shikokuensis*, as in Fig. 44B), etc.

This species is also similar to *E. combustus*, and Uchida (1955) synonymised *E. multidens* stat. rev. under *E. combustus*. However, they are morphologically easily distinguished by body colour pattern (i.e., body entirely testaceous in *E. multidens* stat. rev., as in Fig. 29A, but body with black marks in *E. combustus*, as in Fig. 13A), and DNA barcodes also separate them. Hence, we revise the status of *E. multidens* stat. rev. as a valid species.

#### Enicospilus nigribasalis

Taxon classificationAnimaliaHymenopteraIchneumonidae

(Uchida, 1928)

0111B971-B5D2-5A44-B3A1-66E674B24044

Figure 30

Henicospilus nigribasalis Uchida, 1928: 222; HT ♀ from Taiwan, SEHU, examined.

##### Specimens examined.

Total of 35 specimens (26♀♀9♂♂): India (5♀♀), Japan (14♀♀8♂♂), Sri Lanka (1♀), Taiwan (6♀♀1♂).

Type series: HT ♀ of *Henicospilus nigribasalis* Uchida, 1928, Baibara [= Meiyuan], TAIWAN, 25.VIII.1925, Kikuchi leg. (SEHU).

##### Distribution.

Eastern Palaearctic and Oriental regions (Yu et al. 2016).

JAPAN: [Kantô-Kôshin] Kanagawa (Watanabe et al. 2016; present study); [Tôkai] Shizuoka (Konishi 1993; present study); [Kinki] Hyôgo*; [Chûgoku] Shimane* and Hiroshima (Konishi 1993; Konishi and Nakamura 2000; present study); [Shikoku] Ehime (Konishi and Yamamoto 2000; Hisasue et al. 2015; present study) and Kôchi (Konishi 1993; present study); [Kyûshû] Fukuoka* and Kumamoto (Konishi 1993; present study); [Ryûkyûs] Okinawa (Konishi 1993; present study). *New records. In Japan, it is restricted to the warmer Pacific coast and Ryûkyûs.

##### Bionomics.

No host records from Japan. Recorded as a parasitoid of *Ericeia inangulata* (Guenée) (Erebidae) by Chiu et al. (1984) and Chen et al. (2009).

##### Differential diagnosis.

**Figure 30.**
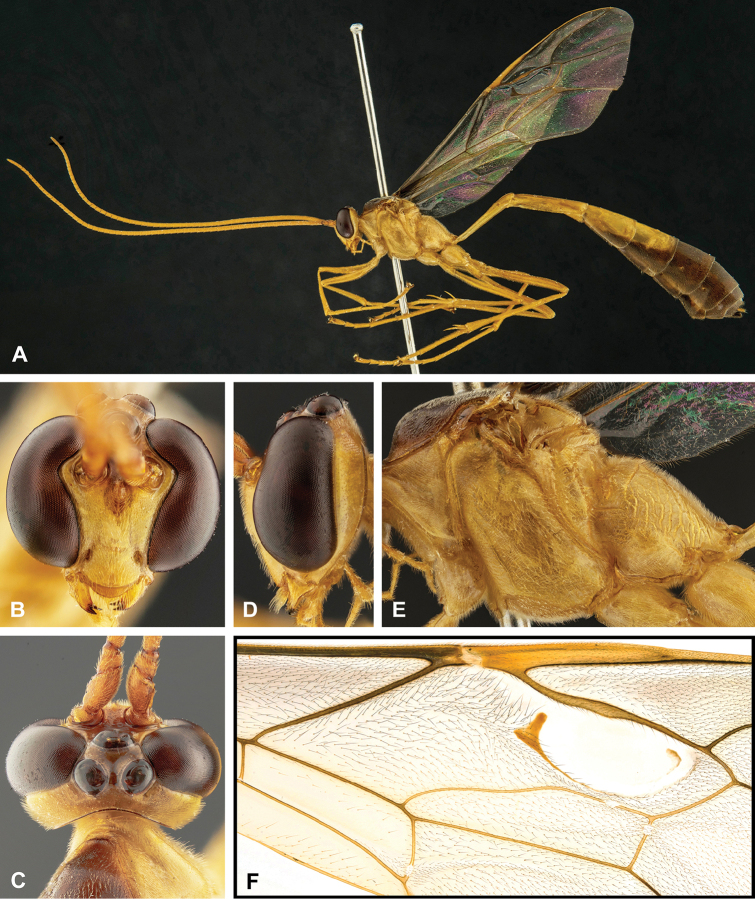
*Enicospilus nigribasalis* (Uchida, 1928) ♀ from Japan **A** habitus **B** head, frontal view **C** head, dorsal view **D** head, lateral view **E** mesosoma, lateral view **F** central part of fore wing.

This species is very easily distinguishable from any other *Enicospilus* species on account of the characteristic colour pattern, especially of the metasoma and wings, as in Fig. 30A. Gauld and Mitchell (1981) suggested that *E. nigribasalis* is closely related to *E. ashbyi* and *E. pallidus*; however, this species can easily be distinguished from them by colour pattern, shape of wing veins, and shape of the fore wing fenestra and sclerites.

#### Enicospilus nigristigma

Taxon classificationAnimaliaHymenopteraIchneumonidae

Cushman, 1937

3CC1458C-EE71-5632-9B62-AE7F9FAC44ED

Figure 31

Enicospilus nigristigma Cushman, 1937: 309; HT ♀ from Taiwan, DEI, not examined.

##### Specimens examined.

Total of 26 specimens (19♀♀7♂♂): Japan (18♀♀7♂♂), Taiwan (1♀).

##### Distribution.

Oriental region (Yu et al. 2016).

JAPAN: [Ryûkyûs] Kagoshima (Konishi 1993; present study) and Okinawa (Konishi 1993; present study).

##### Bionomics.

Unknown.

##### Differential diagnosis.

**Figure 31.**
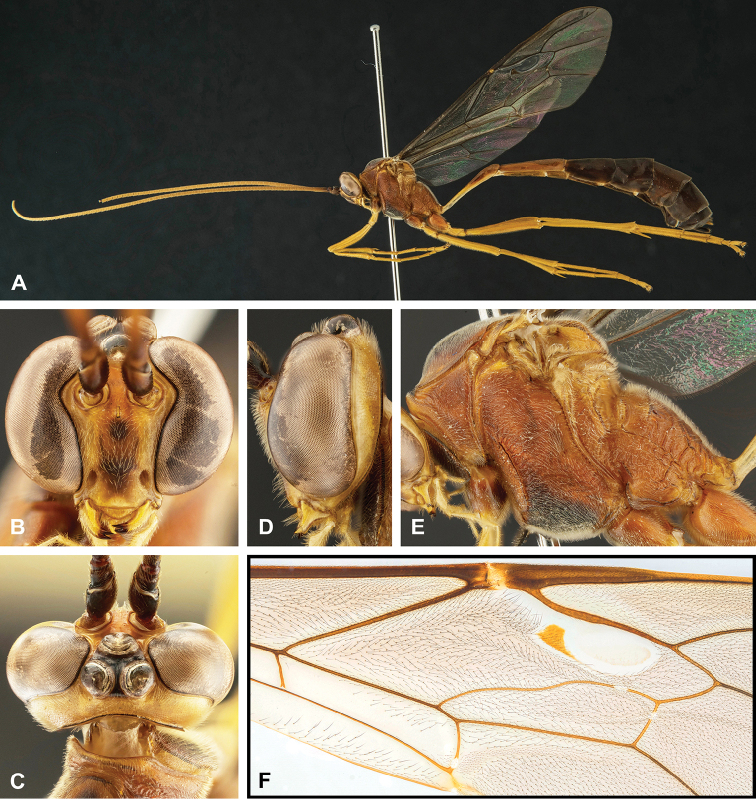
*Enicospilus nigristigma* Cushman, 1937 ♀ from Japan **A** habitus **B** head, frontal view **C** head, dorsal view **D** head, lateral view **E** mesosoma, lateral view **F** central part of fore wing.

This species is one of the largest Japanese ophionines, along with *E. nigronotatus* and *Dicamptus nigropictus* (Matsumura, 1912). The habitus and sculpture of this species are very similar to *E. stimulator* (Smith, 1865), but *E. nigristigma* can readily be distinguished from *E. stimulator* by the larger fore wing fenestra and quadra. Moreover, this species is easily distinguishable from all other Japanese *Enicospilus* by the following combination of character states: large size (i.e., fore wing length always greater than 20.0 mm); interocellar area black (Fig. 31C); mesopleuron coarsely longitudinally striate (Fig. 31E); metapleuron and scutellum coarsely rugose (Fig. 31E); and fore wing fenestra covered with a large unpigmented quadra (Fig. 31F).

#### Enicospilus nigronotatus

Taxon classificationAnimaliaHymenopteraIchneumonidae

Cameron, 1903

E9479D78-C3B4-57F2-B3E9-B95A2D5213BA

Figure 32

Enicospilus nigronotatus Cameron, 1903: 133; LCT ♀ from Borneo, designated by Townes et al. (1961: 284), NHMUK, examined.
Henicospilus triguttatus Uchida, 1928: 221; HT ♀ from Taiwan, SEHU, examined; synonymised by Gauld and Mitchell (1981: 310).

##### Specimens examined.

Total of 18 specimens (11♀♀4♂♂ and 3 unsexed): Brunei (2♀♀2♂♂ and 1 unsexed), Indonesia (2♀♀), Japan (1♀2♂♂), Singapore (1♀), Sri Lanka (2♀♀ and 2 unsexed), Taiwan (2♀♀), unknown (1♀).

Type series: LCT ♀ of *Enicospilus nigronotatus* Cameron, 1903, Borneo, MALAYSIA, Cameron leg. (NHMUK, Type 3b.1271); HT ♀ of *Henicospilus triguttatus* Uchida, 1928, Horisha, TAIWAN, Matsumura leg. (SEHU).

##### Distribution.

Oriental region (Yu et al. 2016).

Newly recorded from Indonesia.

JAPAN: [Ryûkyûs] Okinawa (Shimizu and Maeto 2016; present study).

##### Bionomics.

Unknown.

##### Differential diagnosis.

**Figure 32.**
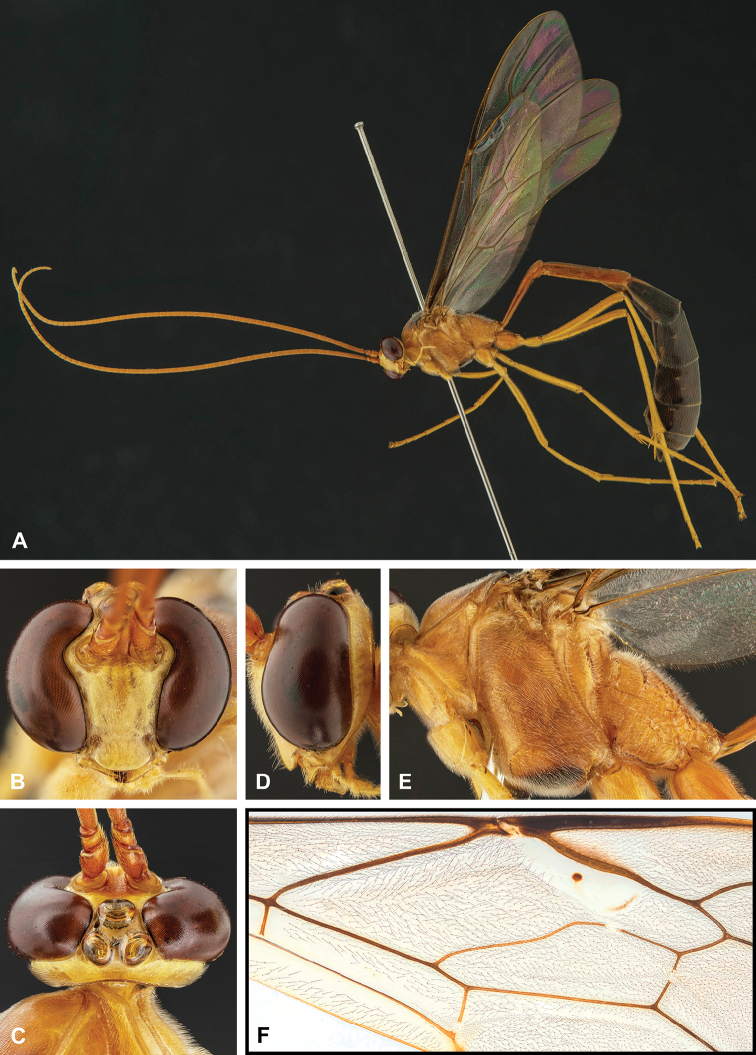
*Enicospilus nigronotatus* Cameron, 1903 ♀ from Japan **A** habitus **B** head, frontal view **C** head, dorsal view **D** head, lateral view **E** mesosoma, lateral view **F** central part of fore wing.

This species is an extremely large insect, as is *E. nigristigma*. Gauld and Mitchell (1981) suggest that this species is related to *E. trilobus* and we agree with this. Although *E. nigronotatus* is probably related to *E. trilobus*, *E. nigronotatus* can be distinguished from it by characters such as the shape of the fore wing fenestra, i.e., fenestra very long, proximally extensively glabrous and proximal end of fenestra widely touching the anterior margin of the discosubmarginal cell, as in Fig. 32F, but fenestra short and the proximal end of the fenestra widely separated from the anterior margin in *E. trilobus*.

This species is easily distinguishable from all other Japanese *Enicospilus* species by the following combination of character states: large size; interocellar area red-brown (Fig. 32C); mesopleuron never entirely coarsely longitudinally striate (Fig. 32E); metapleuron and propodeum coarsely rugose (Fig. 32E); fore wing with both CI and ICI more than 0.7 (Fig. 32F).

#### Enicospilus nigropectus

Taxon classificationAnimaliaHymenopteraIchneumonidae

Cameron, 1905

1DF2B9F9-D92E-5FFF-9988-6A733ECD50B7

Figure 33

Enicospilus nigropectus Cameron, 1905b: 123; HT ♀ from Sarawak, NHMUK, examined.
Henicospilus hariolus Morley, 1912: 44; LCT ♀ from Sri Lanka, designated by Gauld and Mitchell (1981: 220), NHMUK, examined; synonymised by Gauld and Mitchell (1981: 220).
Amesospilus nigrostemmaticus Enderlein, 1921: 19; LCT ♀ from Sumatra, designated by Townes et al. (1961: 288), IZPAN, not examined; synonymised by Gauld and Mitchell (1981: 220).
Henicospilus fuscomaculatus Uchida, 1928: 216; LCT ♀ from Korea, designated by Townes et al. (1965: 328), SEHU, examined; synonymised by Gauld and Mitchell (1981: 220).
Henicospilus fuscomaculatus yakushimensis Yasumatsu, 1934: 67; HT ♀ from Japan, KUEC, examined; synonymised by Watanabe and Yamauchi (2014: 86).

##### Specimens examined.

Total of 225 specimens (123♀♀100♂♂ and 2 unsexed): Brunei (3♀♀2♂♂), China (1♀1♂), Indonesia (1♀), Japan (99♀♀81♂♂ and 2 unsexed), Korea (1♀), Malaysia (1♀2♂♂), Sri Lanka (1♀), Taiwan (15♀♀13♂♂), Thailand (1♀), unknown (1♂).

Type series: HT ♀ of *Enicospilus nigropectus* Cameron, 1905, Kuching, Sarawak, MALAYSIA, V.1903, P. Cameron leg. (NHMUK, Type 3b.1238); LCT ♀ of *Henicospilus hariolus* Morley, 1912, Kandy, SRI LANKA, V.1910, Green leg. (NHMUK, Type 3b.1264); LCT ♀ of *Henicospilus fuscomaculatus* Uchida, 1928, Shakôji, KOREA, VII.1927, Takano leg. (SEHU); HT ♀ of *Henicospilus fuscomaculatus yakushimensis* Yasumatsu, 1934, Yakushima, Kagoshima Pref., Ryûkyûs, JAPAN, 21.VII.1931 (KUEC).

##### Distribution.

Australasian, Eastern Palaearctic, and Oriental regions (Yu et al. 2016).

JAPAN: [Tôhoku] Aomori (Uchida 1928; Ichita 1994; present study) and Yamagata*; [Hokuriku] Niigata*, Ishikawa*, and Fukui*; [Kantô-Kôshin] Tôkyô (Konishi et al. 2014; present study) and Kanagawa (Watanabe et al. 2016; present study); [Tôkai] Shizuoka (Watanabe and Makanai 2011; present study) and Mie (Uchida 1928; present study); [Kinki] Hyôgô (Iwata 1958; present study), Nara*, and Wakayama*; [Chûgoku] Okayama* and Hiroshima (Maeto and Shimizu 2019; present study); [Shikoku] Ehime* and Kôchi (Uchida 1928; present study); [Kyûshû] Fukuoka (Uchida 1928; present study), Kumamoto*, Miyazaki* and Kagoshima (Yasumatsu 1934; Watanabe and Yamauchi 2014; present study); [Ryûkyûs] Kagoshima (Uchida 1956; Watanabe 2018; present study) and Okinawa*. *New records. Ichita (1994) recorded this species under the incorrect spelling, '*nigripectus*'.

This is one of the most frequently encountered wasps in Japan as well as in southeast Asia, especially Sundaland.

##### Bionomics.

Unknown.

##### Differential diagnosis.

**Figure 33.**
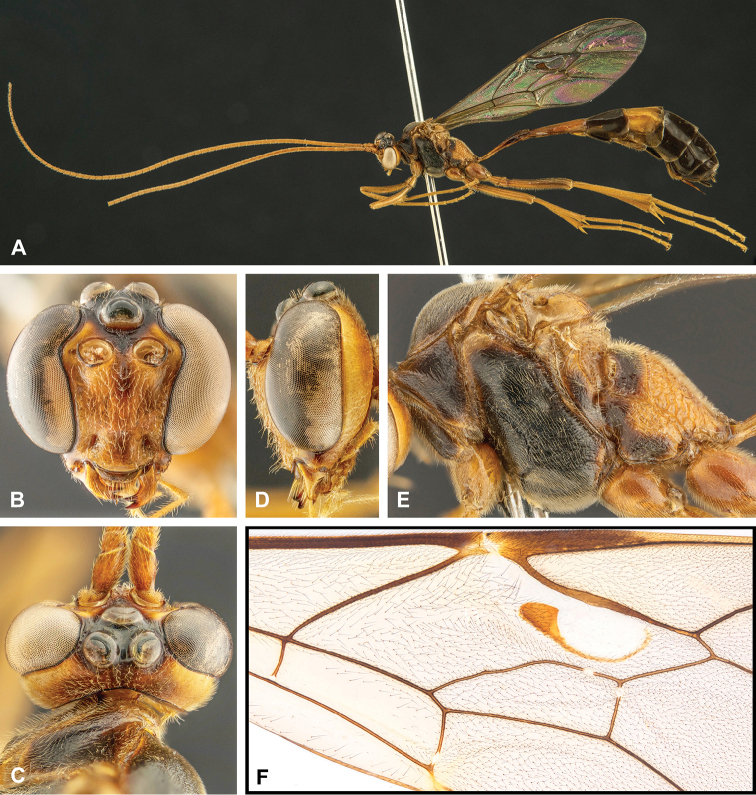
*Enicospilus nigropectus* Cameron, 1905 ♀ from Japan **A** habitus **B** head, frontal view **C** head, dorsal view **D** head, lateral view **E** mesosoma, lateral view **F** central part of fore wing.

This species is very easily distinguished from all other Japanese *Enicospilus* by the black interocellar area (Fig. 33B, C), characteristic bullet-shaped proximal sclerite (Fig. 33F), and the absence of the central sclerite (Fig. 33F). Morphological characters indicate that *E. nigropectus* is very closely related to *E. montaguei* (Turner, 1919), but can be distinguished by the longer fore wing fenestra and irregularly rugose scutellum of *E. nigropectus*. Moreover, this species has been confused with *E. abdominalis* by many authors but is very easily separated from it (cf. Differential diagnosis of *E. abdominalis*).

##### Remarks.

This species exhibits a wide range of colour variation, but the Japanese specimens are morphologically stable. Japanese specimens have a slightly wider mandible than specimens from other regions, as mentioned by Gauld and Mitchell (1981); however, we have not found any other characters to separate the Japanese population.

#### Enicospilus pseudoconspersae

Taxon classificationAnimaliaHymenopteraIchneumonidae

(Sonan, 1927)

902E57B7-F61C-5AEF-BF5D-C983AB198564

Figure 34

Henicospilus pseudoconspersae Sonan, 1927: 48; HT ♂ from Taiwan, TARI, examined.
Henicospilus mushanus Uchida, 1928: 216; HT ♀ from Taiwan, SEHU, examined; synonymised by Gauld and Mitchell (1981: 344).
Enicospilus tenuinubeculus Chiu, 1954: 34; HT ♀ from China, TARI, examined; synonymised by Gauld and Mitchell (1981: 345).

##### Specimens examined.

Total of 21 specimens (12♀♀9♂♂): China (1♀1♂), Japan (10♀♀6♂♂), Nepal (1♂), Taiwan (1♀1♂).

Type series: HT ♂ of *Henicospilus pseudoconspersae* Sonan, 1927, Taihoku, TAIWAN, 25.IV.1927, J. Sonan leg. (TARI); HT ♀ of *Henicospilus mushanus* Uchida, 1928, Musha, TAIWAN, 24.VII.1925, Matsumura (SEHU); HT ♀ of *Enicospilus tenuinubeculus* Chiu, 1954, Fukien, Shaown, CHINA, 23–29.V.1944, H.F. Chao leg. (TARI).

##### Distribution.

Eastern Palaearctic and Oriental regions (Yu et al. 2016).

JAPAN: [Kantô-Kôshin] Saitama*; [Tôkai] Shizuoka*; [Kinki] Hyôgo*; [Chûgoku] Shimane* and Hiroshima (Maeto and Shimizu 2019; present study); [Shikoku] Tokushima* and Kôchi*; [Kyûshû] Kumamoto* and Kagoshima*; [Ryûkyûs] Kagoshima* and Okinawa (Sonan 1940; Shimizu 2020; present study). *New records.

##### Bionomics.

No host records from Japan. Described as a parasitoid of *Arna pseudoconspersa* Strand (Erebidae) (Sonan 1944; Gauld and Mitchell 1981; Chen et al. 2009) and recorded as a parasitoid of Lymantriinae (Erebidae) in China by He et al. (2004).

##### Differential diagnosis.

**Figure 34.**
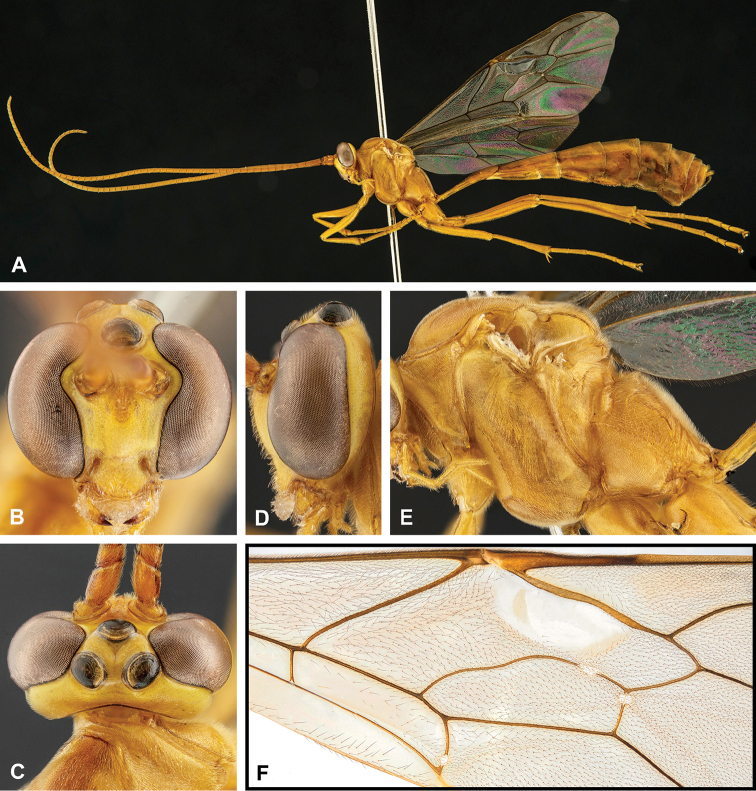
*Enicospilus pseudoconspersae* (Sonan, 1927) ♀ from Japan **A** habitus **B** head, frontal view **C** head, dorsal view **D** head, lateral view **E** mesosoma, lateral view **F** central part of fore wing.

This species can be very easily distinguished from all other *Enicospilus* species by its characteristic sclerites of the fore wing fenestra (i.e., proximal sclerite entirely weakly pigmented and half-moon-shaped, and margin of the proximal sclerite distinctly separated from the margin of the fenestra, as in Fig. 34F).

This species exhibits a wide range of morphological variation in size and colour pattern. The proximal sclerite is usually weakly pigmented but is strongly pigmented in the holotype of *E. tenuinubeculus*.

#### Enicospilus pseudopuncticulatus

Taxon classificationAnimaliaHymenopteraIchneumonidae

Shimizu sp. nov.

38E26CA5-C3F6-52A9-BD55-43CCD8D6D77E

http://zoobank.org/24488D4C-E242-4199-B87E-98B67E27BC4A

Figure 35

##### Etymology.

This species is very close to *E. puncticulatus*, hence the specific name based on their similarity.

##### Type series.

Total of 8 specimens (5♀♀3♂♂): Japan (5♀♀3♂♂).

HT: ♀, NIAES, Tsukuba City, Ibaraki Pref., Kantô-Kôshin, JAPAN, 17–26.VII.1989, M. Sharkey leg. (NIAES).

PT: 1♂, Awashima, Niigata Pref., Hokuriku, JAPAN, 7.VII.1937, K. Baba & J. Sawano leg. (MNHA); 1♂, same data as HT (NIAES); 1♀, NIAES, Tsukuba City, Ibaraki Pref., Kantô-Kôshin, JAPAN, 14–19.IV.1989, M. Sharkey leg. (EMUS); 2♀♀, Nokata, Nisshin, Aichi Pref., Tôkai, JAPAN, 10–16.VII.2011 (1♀), 23–30.VII.2011 (1♀), H. Seo & R. Mizutani leg. (MsT) (ELMU); 1♀1♂, Niryou, Takatsuki City, Ōsaka Pref., Kinki, JAPAN, 30.VI–4.VIII.2013, S. Fujie leg. (MsT) (♀, MNHA, SEN176–DDBJ-LC492933; ♂, OMNH, SEN147–DDBJ-LC492932).

##### Distribution.

Eastern Palaearctic region.

JAPAN: [Hokuriku] Niigata; [Kantô-Kôshin] Ibaraki; [Tôkai] Aichi; [Kinki] Ōsaka.

##### Bionomics.

Unknown.

##### Differential diagnosis.

**Figure 35.**
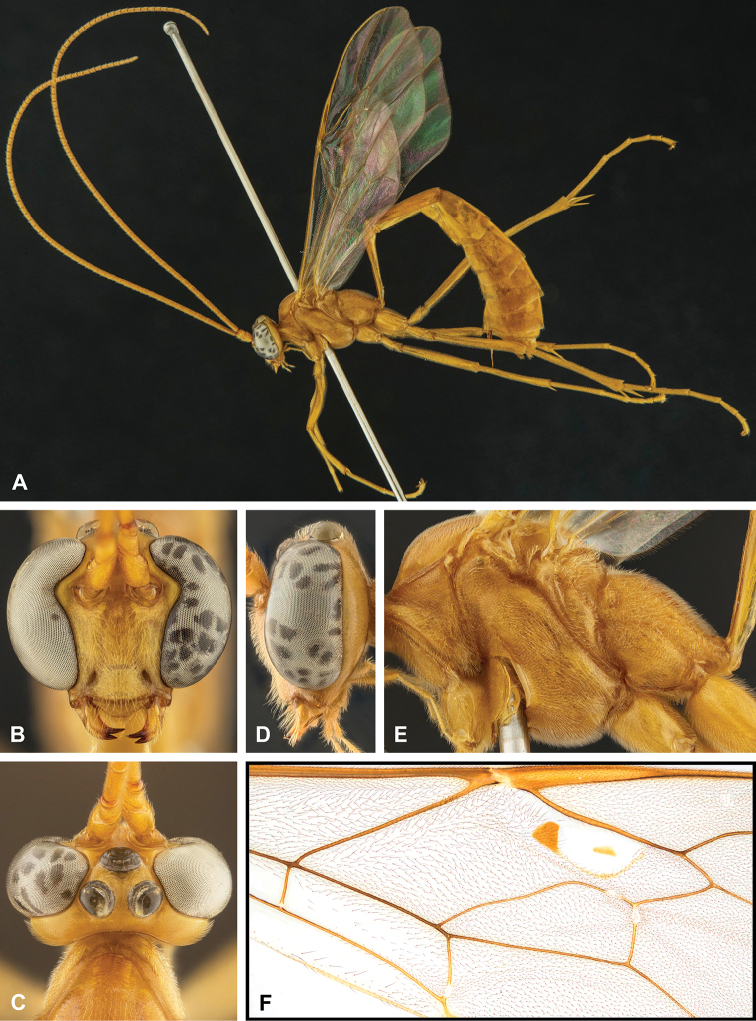
*Enicospilus pseudopuncticulatus* Shimizu, sp. nov. ♀ (**A–E**HT**F**PT) **A** habitus **B** head, frontal view **C** head, dorsal view **D** head, lateral view **E** mesosoma, lateral view **F** central part of fore wing.

Although this species is very close to *E. puncticulatus* based on both morphology and DNA barcoding (Fig. 6), it is rather readily distinguishable by the linear central sclerite and straight fore wing vein 1cu-a, as in Fig. 35F.

##### Description.

Female (n = 5). Body length 20.0–22.0 (HT: ca. 20.5) mm.

Head with GOI = 3.0–3.2 (HT: 3.1) (Fig. 35D). Lower face 0.8× as wide as high, strongly shiny, finely punctate with setae (Fig. 35B). Clypeus 1.7–2.0× (HT: 1.9) as wide as high, moderately punctate with setae as in upper face, moderately convex in profile, ventral margin impressed (Fig. 35B, D). Malar space 0.3× as long as basal mandibular width (Fig. 35B, D). Mandible moderately twisted by 25–40° (HT: ca. 30°), rather long, proximally narrowed, distally parallel-sided, outer surface with diagonal setose groove (Fig. 35B, D). Upper tooth of mandible 1.8–2.1× (HT: 2.1) as long as lower (Fig. 35B). Frons, vertex and gena strongly shiny with fine setae (Fig. 35B–D). Posterior ocellus very close to eye (Fig. 35B–D). Ventral end of occipital carina joining oral carina. Antennae with 66–69 (HT: 67) flagellomeres; first flagellomere 1.6–1.7× (HT: 1.6) as long as second; 20^th^ flagellomere 2.0–2.1× (HT: 2.0) as long as wide.

Mesosoma entirely very strongly shiny with setae (Fig. 35E). Pronotum diagonally striate to moderately punctate (Fig. 35E). Mesoscutum 1.5× as long as maximum width, finely punctate with setae, and evenly rounded in profile (Fig. 35E). Notauli absent (Fig. 35E). Scutellum moderately convex, finely punctate, with lateral longitudinal carinae along entire length of scutellum. Epicnemium densely punctate with setae. Epicnemial carina strong, evenly curved to anterior, dorsal end not reaching anterior margin of mesopleuron (Fig. 35E). Mesopleuron entirely longitudinally striate to punctostriate (Fig. 35E). Submetapleural carina broadened anteriorly (Fig. 35E). Metapleuron entirely moderately punctate with diagonal striae posteriorly (Fig. 35E). Propodeum declivous in profile; anterior transverse carina complete; anterior area longitudinally striate; spiracular area finely punctate with setae; posterior area finely to moderately rugose; propodeal spiracle elliptical and not joining pleural carina by ridge (Fig. 35E).

Wings (Fig. 35F). Fore wing length 13.0–14.0 (HT: ca. 13.0) mm with AI = 0.3–0.6 (HT: 0.5), CI = 0.2–0.3 (HT: 0.3), DI = 0.4, ICI = 0.5, SDI = 1.1–1.2 (HT: 1.1), SI = 0.2, SRI = 0.3; vein 1m-cu&M moderately sinuous; vein 2r&RS slightly sinuous; vein RS evenly curved; fenestra and sclerites of discosubmarginal cell as in Fig. 35F; proximal sclerite rounded triangular, not confluent with distal sclerite, strongly pigmented; central sclerite linear and pigmented, subparallel to 2r&RS, positioned in medio-distal part of fenestra; distal sclerite moderately pigmented; proximal corner of marginal cell evenly setose; posterodistal corner of second discal cell 95–100° (HT: ca. 95°) and of subbasal cell 75–85° (HT: ca. 75°); vein 1cu-a antefurcal to M&RS by 0.1× length of 1cu-a. Hind wing with NI = 1.3–1.4 (HT: 1.4); vein RS slightly evenly curved; vein RA with 7–8 (HT: 7) uniform hamuli.

Legs. Hind leg with coxa in profile 1.9–2.0× (HT: 1.9) as long as deep; basitarsus 2.0–2.1× (HT: 2.0) as long as second tarsomere; fourth tarsomere 4.5–4.6× (HT: 4.6) as long as wide; tarsal claw simply pectinate.

Metasoma with DMI = 1.3–1.4 (HT: 1.3), PI = 2.9–3.3 (HT: 3.2), THI = 2.2–2.6 (HT: 2.3); thyridium suboval, moderately sized; ovipositor sheath not longer than posterior depth of metasoma (Fig. 35A).

Colour (Fig. 35). Entirely red-brown except for apex of mandible black. Wings hyaline; sclerites pigmented and amber; veins red-brown.

Male (n = 3). Very similar to female.

#### Enicospilus pudibundae

Taxon classificationAnimaliaHymenopteraIchneumonidae

(Uchida, 1928)

FC06CA5E-0289-55B6-A71C-3AB114C035B4

Figure 36

Henicospilus pudibundae Uchida, 1928: 219; LCT ♂ from Japan, designated by Townes et al. (1965: 330), SEHU, examined.

##### Specimens examined.

Total of 124 specimens (85♀♀37♂♂ and 2 unsexed): Brunei (3♀♀), India (1♀), Japan (81♀♀37♂♂ and 2 unsexed).

Type series: LCT ♂ of *Henicospilus pudibundae* Uchida, 1928, Sapporo, Hokkaidô, JAPAN, 4.VI.1925, Tamanuki leg. (emerged from *Dasychira pudibunda* L.) (SEHU).

##### Distribution.

Eastern Palaearctic and Oriental regions (Yu et al. 2016).

JAPAN: [Hokkaidô] (Uchida 1928; Shimizu 2020; present study); [Tôhoku] Aomori*, Iwate*, Yamagata*, and Fukushima*; [Hokuriku] Niigata (Shimizu 2020) and Ishikawa (Togashi 2005); [Kantô-Kôshin] Ibaraki*, Tochigi*, Gunma*, Nagano (Chiu 1954; present study), Yamanashi*, Saitama*, and Tôkyô (Uchida 1928; Shimizu 2020; present study); [Tôkai] Shizuoka*, Gifu (Chiu 1954), and Mie*; [Kinki] Kyôto*, Hyôgo (Shimizu 2020), and Wakayama*; [Chûgoku] Hiroshima (Konishi and Nakamura 2002, 2005, 2010; Maeto and Shimizu 2019; Shimizu 2020; present study) and Yamaguchi (Konishi and Nakamura 2002; present study); [Shikoku] Tokushima*, Ehime* and Kôchi*; [Kyûshû] Fukuoka*, Nagasaki*, Miyazaki* and Kagoshima (Fukuda and Kusigemati 1986; present study). *New records.

##### Bionomics.

Reared from Erebidae (Lymantriinae) in Japan: *Calliteara pudibunda* (L.) (Uchida 1928, 1930) and *Orgyia thyellina* Butler (Togashi 2005).

##### Differential diagnosis.

**Figure 36.**
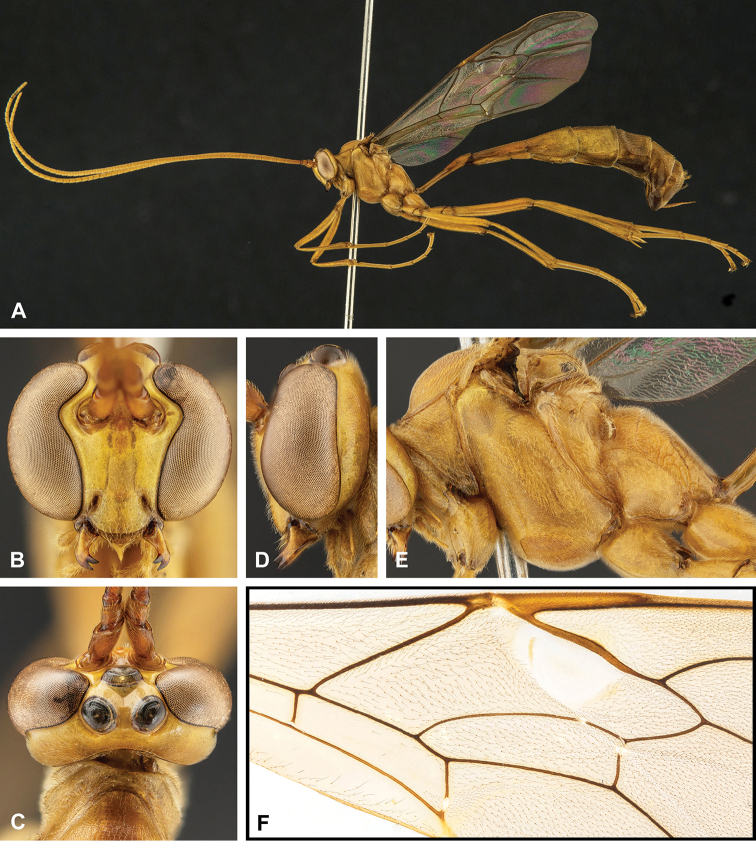
*Enicospilus pudibundae* (Uchida, 1928) ♀ from Japan **A** habitus **B** head, frontal view **C** head, dorsal view **D** head, lateral view **E** mesosoma, lateral view **F** central part of fore wing.

This species resembles *E. biharensis*, *E. maruyamanus* and *E. transversus*, all of which are rather difficult to distinguish from each other. However, *E. pudibundae* can be distinguished by the evenly curved fore wing vein 1m-cu&M (Fig. 36F), lack of proximal pectinae of the hind tarsal claw, and entirely closely punctate meso- and metapleuron (Fig. 36E).

Gauld and Mitchell (1981) separated *E. biharensis*, *E. pudibundae* and *E. transversus* by the value of CI, but with the caveat, “Whether this character will prove to be completely reliable we doubt”. However, we consider these species to be certainly distinct when using a combination of characters (cf. Table 7).

#### Enicospilus puncticulatus

Taxon classificationAnimaliaHymenopteraIchneumonidae

Tang, 1990

72FE5683-E935-5E11-A859-C168CE04FA5F

Figure 37

Enicospilus puncticulatus Tang, 1990: 128; HT ♀ from China, FAFU, not examined.

##### Specimens examined.

Total of 31 specimens (24♀♀7♂♂): Japan (22♀♀6♂♂), Taiwan (2♀♀1♂).

##### Distribution.

Oriental region (Tang 1990); new to the Eastern Palaearctic region.

Newly recorded from Japan.

JAPAN: [Hokkaidô]; [Hokuriku] Niigata; [Kantô-Kôshin] Tochigi, Nagano, Yamanashi, and Saitama; [Kinki] Hyôgo; [Shikoku] Kôchi; [Kyûshû] Fukuoka.

##### Bionomics.

Unknown.

##### Differential diagnosis.

**Figure 37.**
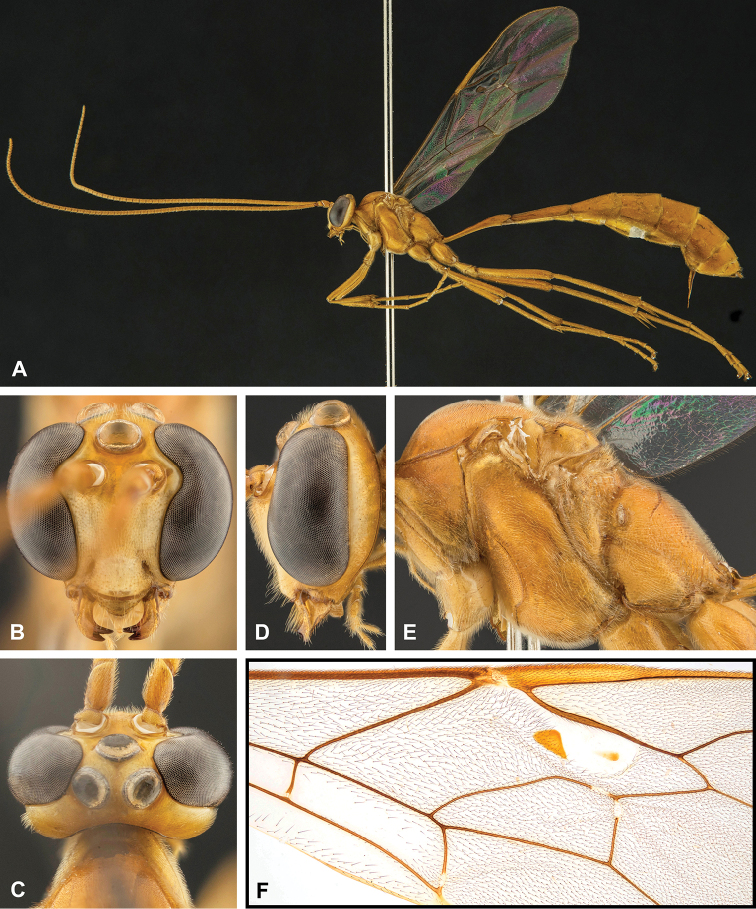
*Enicospilus puncticulatus* Tang, 1990 ♀ from Japan **A** habitus **B** head, frontal view **C** head, dorsal view **D** head, lateral view **E** mesosoma, lateral view **F** central part of fore wing.

This species is most similar to *E. pseudopuncticulatus* sp. nov., but easily distinguished by the rounded central sclerite and more or less curved fore wing vein 1cu-a, as in Fig. 37F. Furthermore, *E. puncticulatus* resembles *E. melanocarpus*, but can be distinguished by the usually separated proximal and distal sclerites (Fig. 37F) (proximal and distal sclerites strongly confluent in *E. melanocarpus*, as in Fig. 28F) and entirely testaceous metasoma (Fig. 37A) (posterior metasomal segments almost always black in *E. melanocarpus*, as in Fig. 28A).

##### Remarks.

Specimens with various shapes of sclerites of the fore wing fenestra but which are very similar in sculpture are identified as this species by the key provided by Tang (1990). It has proved impossible to segregate morphospecies based on discrete differences in sclerites, and our DNA barcodes differ by less than 1%; if *E. puncticulatus* represents a species complex, it seems that the CO1 gene is not useful for delimiting species in this complex.

#### Enicospilus pungens

Taxon classificationAnimaliaHymenopteraIchneumonidae

(Smith, 1874)

5F36CE27-34B8-57D1-9472-E6F860BC2E19

Figure 38

Ophion pungens Smith, 1874: 396; HT ♂ from Japan, NHMUK, examined, photographs provided by Shimizu and Broad (2020: fig. 24).
Enicospilus striatus Cameron, 1899: 103; HT ♀ from India, OUMNH, not examined; junior secondary homonym of Enicospilus striatus (Brullé); **syn. nov.**
Henicospilus lineolatus Roman, 1913: 30; HT ♂ from Philippines, NR, not examined; **syn. nov.**
Enicospilus uniformis Chiu, 1954: 25; HT ♀ from Taiwan, TARI, examined; **syn. nov.**
Enicospilus flatus Chiu, 1954: 28; HT ♀ from Taiwan, TARI, examined; **syn. nov.**
Enicospilus gussakovskii Viktorov, 1957: 185; HT ♀ from Moscow, Ussr, not examined; **syn. nov.**
Enicospilus striolatus Townes, Townes and Gupta, 1961: 290; replacement name for Enicospilus striatus Cameron, 1899; **syn. nov.**
Enicospilus unicornis Rao and Nikam, 1969: 343; LCT ♂ from India, designated by Gauld and Mitchell (1981: 304), NHMUK, examined; **syn. nov.**
Enicospilus unicornis Rao and Nikam, 1970: 103; HT ♀ from India, MUC, not examined; junior primary homonym of Enicospilus unicornis Rao & Nikam, 1969; **syn. nov.**

##### Specimens examined.

Total of 174 specimens (143♀♀25♂♂ and 6 unsexed): Australia (1♀), Brunei (2♀♀), India (37♀♀9♂♂ and 1 unsexed), Japan (54♀♀3♂♂), Nepal (4♀♀), Papua New Guinea (2♀♀), Sri Lanka (1♀), Taiwan (41♀♀13♂♂ and 5 unsexed), unknown (1♀).

Type series: HT ♂ of *Ophion pungens* Smith, 1874, Hyôgo Pref., Kinki, JAPAN (NHMUK, Type 3b.1274); HT ♀ of *Enicospilus uniformis* Chiu, 1954, Taihoku, TAIWAN, 14.IV.1921, S. Aoki leg. (TARI); HT ♀ of *Enicospilus flatus* Chiu, 1954, Taihoku, TAIWAN, 28.V.1931, J. Sonan leg. (TARI); LCT ♂ of *Enicospilus unicornis* Rao & Nikam, 1969, Himayatbagh, Aurangabad, Maharashtra, INDIA, VIII.1968, Nikam leg. (NHMUK, Type 3b.2858).

##### Distribution.

Australasian, Eastern Palaearctic, Oceanic, and Oriental regions (Yu et al. 2016).

Newly recorded from Australia, Bhutan, Brunei, Indonesia, Laos, Malaysia, Nepal, New Caledonia, Papua New Guinea, Philippines, Solomon Islands, Sri Lanka, Tajikistan, and Taiwan.

JAPAN: [Hokkaidô] (Shimizu 2020); [Tôhoku] Yamagata* and Fukushima*; [Hokuriku] Niigata (Ohmori 1960; present study); [Kantô-Kôshin] Tochigi (Katayama et al. 2016; present study), Ngano (Chiu 1954), Yamanashi*, and Tôkyô (Konishi et al. 2014; present study); [Tôkai] Shizuoka* and Mie (Uchida 1928; present study); [Kinki] Kyôto (Chiu 1954; present study) and Hyôgo (Smith 1874; Uchida 1928; present study); [Shikoku] Ehime* and Kôchi*; [Kyûshû] Saga* and Kagoshima (Momoi 1970; Fukuda and Kusigemati 1986; Watanabe and Yamauchi 2014; preseny study); [Ryûkyûs] Kagoshima (Uchida 1956; present study) and Okinawa (Matsumura and Uchida 1926; Uchida 1928; present study). *New records.

##### Bionomics.

No host records from Japan. A variety of hosts have been reported in the literature (e.g., Tang 1990; Chen et al. 2009), but concentrated in Erebidae.

##### Differential diagnosis.

**Figure 38.**
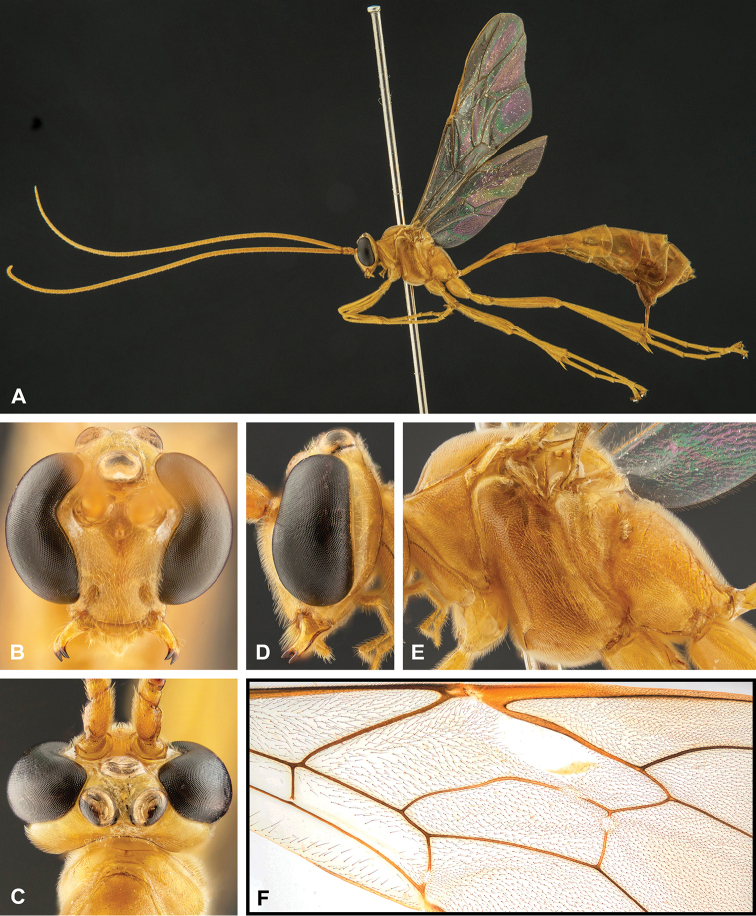
*Enicospilus pungens* (Smith, 1874) ♀ from Japan **A** habitus **B** head, frontal view **C** head, dorsal view **D** head, lateral view **E** mesosoma, lateral view **F** central part of fore wing.

This species is easily distinguished from all other Japanese species of *Enicospilus* by the absence of central and proximal sclerites and presence of a thick and pigmented distal sclerite (Fig. 38F).

##### Remarks.

According to Gauld and Mitchell (1981), the holotype of *Ophion pungens* runs to *E. biharensis* in their key and differs from it by the mandible characters. However, wing morphology clearly differs between *E. pungens* and *E. biharensis*, and *E. lineolatus* syn. nov. is the same species as *E. pungens*. Hence, *E. lineolatus* syn. nov. and the names previously placed in synonymy with *E. lineolatus* syn. nov. are newly synonymised under *E. pungens* here.

The treatment of *Enicospilus unicornis* Rao & Nikam, 1969 as a valid name requires some explanation. Rao and Nikam (1969) published a description of the male of this species, which Gauld and Mitchell (1981) regarded as a valid description of *E. unicornis*, and they designated a lectotype. Rao and Nikam’s (1970) subsequent description of the species under the same name, *unicornis*, including a holotype designation, was regarded by Gupta (1987) as the valid description, and the 1969 description as invalid. We agree with Gauld and Mitchell (1981), that the description of *E. unicornis* from 1969 was a valid description, and the material listed should be regarded as a type series. Therefore, we accept their lectotype designation and *E. unicornis* Rao & Nikam, 1970 as a junior homonym and synonym of *E. unicornis* Rao & Nikam, 1969, contrary to the listing in Yu et al. (2016).

#### Enicospilus ramidulus

Taxon classificationAnimaliaHymenopteraIchneumonidae

(Linnaeus, 1758)

374584E8-BFD3-59BC-8F38-A6E833DB9E28

Figure 39

Ichneumon ramidulus Linnaeus, 1758: 566; HT, sex and locality unknown, not examined.

##### Specimens examined.

Total of 144 specimens (93♀♀50♂♂ and 1 unsexed): England (4♀♀1♂), Germany (1♀), Italy (1♀), Japan (71♀♀47♂♂ and 1 unsexed), Korea (1♀), Mallorca (6♀♀), Russia (1♀), Scotland (1♀), Spain (1♀), Sweden (2♀♀1♂), Switzerland (1♂), unknown (4♀♀).

##### Distribution.

Afrotropical, Oriental, and trans-Palaearctic regions (Yu et al. 2016); this is a predominantly Palaearctic species and may be restricted to there, i.e., all reliable distribution records have been only from the Palaearctic region. *Enicospilus ramidulus* is one of the most frequently encountered *Enicospilus* species throughout the Palaearctic.

JAPAN: [Hokkaidô] (Uchida 1928; Hori et al. 2009; present study); [Tôhoku] Aomori* and Fukushima*; [Hokuriku] Niigata (Ohmori 1960; present study), Toyama*, Ishikawa*, and Fukui*; [Kantô-Kôshin] Ibaraki*, Tochigi*, Nagano (Uchida 1928; present study), Saitama*, Tôkyô (Konishi et al. 2014; present study), and Kanagawa (Kawashima et al. 2018; present study); [Tôkai] Shizuoka* and Mie*; [Kinki] Kyôto (Chiu 1954; Iwata 1960), Ōsaka*, Hyôgo (Iwata 1958, 1960; present study), Nara (Iwata 1960), and Wakayama*; [Chûgoku] Shimane (Konishi and Nakamura 2000, 2002; present study) and Hiroshima (Konishi and Nakamura 2000, 2002, 2005, 2010; present study); [Shikoku] Kagawa (Iwata 1960) and Ehime (Konishi and Yamamoto 2000); [Kyûshû] Fukuoka* and Kumamoto*. *New records.

##### Bionomics.

Recorded from a wide variety of hosts, but some records are undoubtedly the result of misidentifications of the ichneumonid. Reliable rearings are from species of Noctuidae, particularly the subfamily Hadeninae (Broad and Shaw 2016).

##### Differential diagnosis.

**Figure 39.**
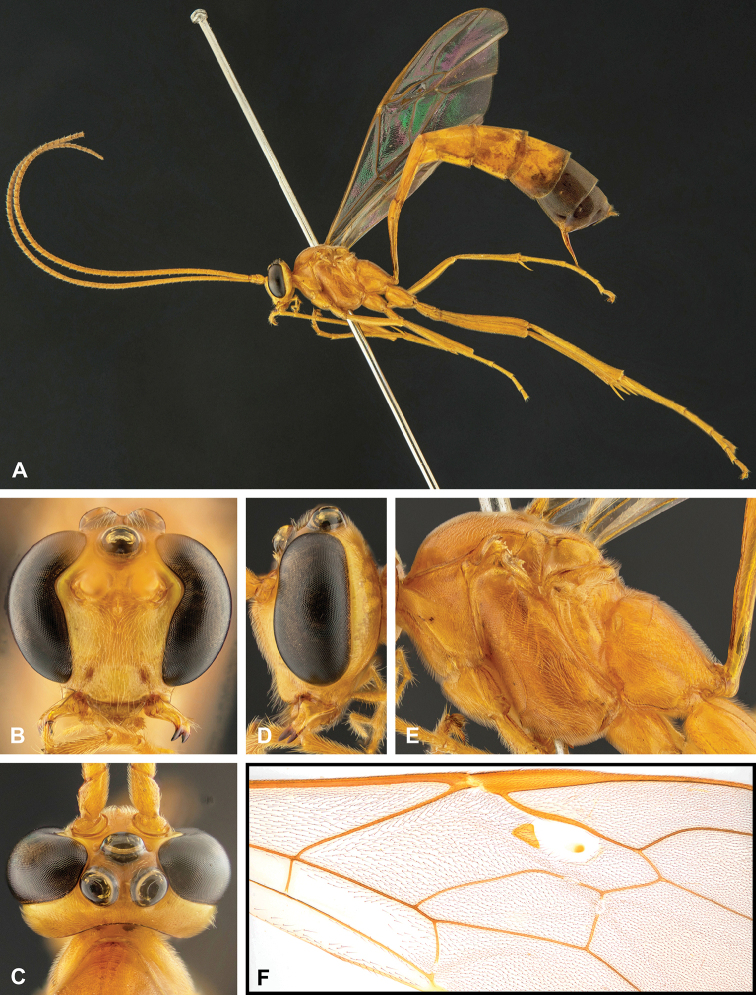
*Enicospilus ramidulus* (Linnaeus, 1758) ♀ from Japan **A** habitus **B** head, frontal view **C** head, dorsal view **D** head, lateral view **E** mesosoma, lateral view **F** central part of fore wing.

This species is sometimes confused with *E. melanocarpus* but is easily distinguishable (cf. Differential diagnosis of *E. melanocarpus*). Some species have similarly shaped fore wing sclerites, but *E. ramidulus* can be distinguished by many characters, for example, the wider face (Fig. 39B), black posterior segments of the metasoma (Fig. 39A), and entirely moderately punctate meso- and metapleuron (Fig. 39E). Some other Japanese species share a similar colour pattern (i.e., body entirely testaceous except for black posterior segments of the metasoma, as in Figs 28A, 42A, 53A) as *E. ramidulus* (Fig. 39A); these species can be separated from each other using the summarised characters in Table 8.

#### Enicospilus riukiuensis

Taxon classificationAnimaliaHymenopteraIchneumonidae

(Matsumura & Uchida, 1926)

D4408FBD-95D6-539E-B53D-EEB31E4E1E80

Figure 40

Henicospilus riukiuensis Matsumura and Uchida, 1926: 71; HT ♂ from Ryûkyûs, SEHU, examined.
Enicospilus nasutus Chiu, 1954: 65; HT ♀ from Taiwan, TARI, examined; synonymised by Gauld and Mitchell (1981: 278).
Enicospilus vontalis Gauld and Mitchell, 1978: 125; HT ♀ from Madagascar, MNHN, not examined; synonymised by Gauld and Mitchell (1981: 278).

##### Specimens examined.

Total of 29 specimens (20♀♀7♂♂ and 2 unsexed): Indonesia (1♀), Japan (16♀♀5♂♂ and 1 unsexed), Madagascar (1 unsexed), Malaysia (2♀♀), Taiwan (1♀2♂♂).

Type series: HT ♂ of *Henicospilus riukiuensis* Matsumura & Uchida, 1926, Okinawa, Ryûkyûs, JAPAN, Sakaguchi leg. (SEHU); HT ♀ of *Enicospilus nasutus* Chiu, 1954, Kanshirei, Tainan, TAIWAN, 9.XI.1926, S. Toyota leg. (TARI); AT ♂ of *E. nasutus*, Kuraru [= Kueitzuchiao in Hengchun, Kenting National Park], TAIWAN, 31.VII.1931, T. Shiraki leg. (TARI); PT of *Enicospilus vontalis* Gauld & Mitchell, 1978, unsexed, Bekily, Madagascar, VI.1936, A. Seyrig leg. (NHMUK).

##### Distribution.

Afrotropical, Australasian, Eastern Palaearctic, Oceanic and Oriental regions (Yu et al. 2016). This is a widely distributed species in tropical to temperate regions of the Old World. Gauld and Mitchell (1981) thought that the presence of *E. riukiuensis* in the Afrotropical region (Madagascar) resulted from human trans-Indian Ocean trade, although there is no reliable evidence to support or refute this hypothesis.

Newly recorded from Indonesia.

JAPAN: [Kantô-Kôshin] Tochigi (Katayama et al. 2016) and Kanagawa*; [Tôkai] Gifu* and Mie*; [Kinki] Kyôto (Chiu 1954; present study), Hyôgo*, and Wakayama*; [Chûgoku] Shimane (Konishi and Nakamura 2002, 2010; present study) and Hiroshima (Konishi and Nakamura 2005; present study); [Shikoku] Kôchi*; [Kyûshû] Fukuoka (Chiu 1954; present study), Nagasaki* and Kagoshima*; [Ryûkyûs] Okinawa (Matsumura and Uchida 1926; Uchida 1928; present study). *New records.

##### Bionomics.

Unknown.

##### Differential diagnosis.

**Figure 40.**
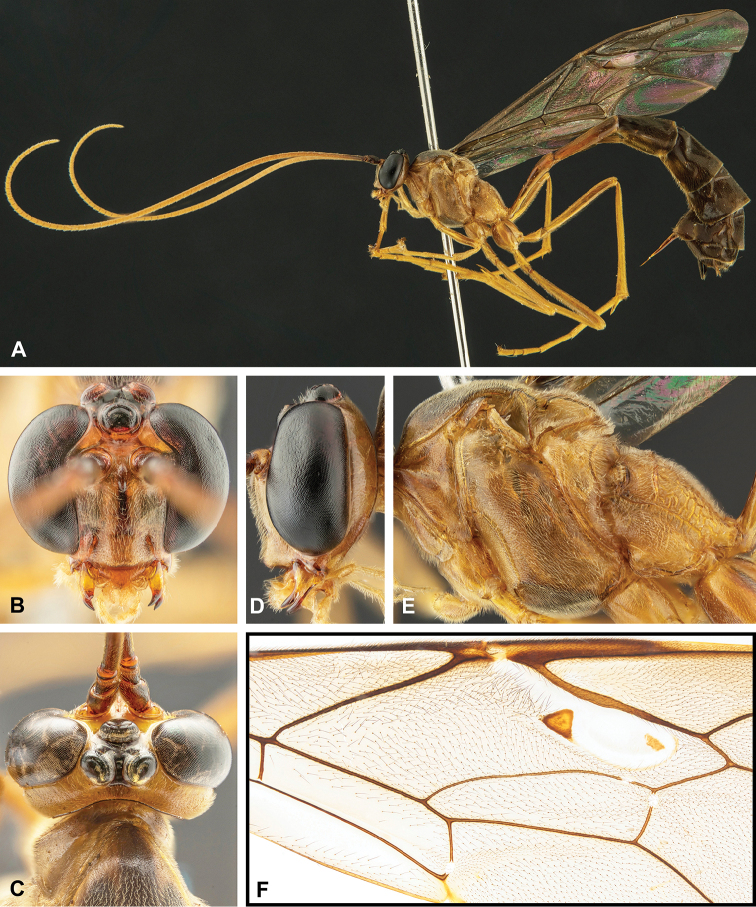
*Enicospilus riukiuensis* (Matsumura & Uchida, 1926) ♀ from Japan **A** habitus **B** head, frontal view **C** head, dorsal view **D** head, lateral view **E** mesosoma, lateral view **F** central part of fore wing.

This species is one of the most easily identified *Enicospilus* species based on the following character states: clypeus nasute (Fig. 40B, D); mandible evenly tapered, rather short and stout, with upper tooth as long as lower (Fig. 40B, D); proximal and central sclerites strongly sclerotised (Fig. 40F).

##### Remarks.

The central sclerite of the Madagascan specimens is smaller than others. The colour of the interocellar area is usually a useful character for identification of *Enicospilus*, however, the interocellar area varies considerably in colour in *E. riukiuensis*.

The nasute clypeus of *E. riukiuensis* is unique among the described Ophioninae, although a similar clypeus is also known in other Ichneumonidae, such as *Zagryphus* Cushman, 1919 of Tryphoninae, but its function is unknown. In addition, some undescribed Oriental *Enicospilus* species have a similar clypeus and mandible.

#### Enicospilus sakaguchii

Taxon classificationAnimaliaHymenopteraIchneumonidae

(Matsumura & Uchida, 1926)

7A8262EB-8F2D-5E41-8146-2C3519812FED

Figure 41

Henicospilus sakaguchii Matsumura and Uchida, 1926: 73; HT ♂ from Ryûkyûs, SEHU, examined.
Enicospilus iracundus Chiu, 1954: 17; HT ♂ from Ryûkyûs, TARI, examined; **syn. nov.**

##### Specimens examined.

Total of 141 specimens (94♀♀29♂♂ and 18 unsexed): Japan (45♀♀13♂♂), Taiwan (49♀♀16♂♂ and 18 unsexed).

Type series: HT ♂ of *Henicospilus sakaguchii* Matsumura & Uchida, 1926, Okinawa, Ryûkyûs, JAPAN, S. Sakaguchi leg. (SEHU); HT ♂ of *Enicospilus iracundus* Chiu, 1954, Okinawa, Ryûkyûs, JAPAN, 1922, J. Sonan (TARI).

##### Distribution.

Eastern Palaearctic and Oriental regions (Yu et al. 2016); this is a predominantly Oriental species.

Newly recorded from Indonesia.

JAPAN: [Tôhoku] Aomori*; [Kantô-Kôshin] Tochigi* and Kanagawa*; [Tôkai] Shizuoka*; [Kyûshû] Kagoshima (Nagatomi et al. 1972); [Ryûkyûs] Kagoshima (Kusigemati 1972; Nagatomi et al. 1972; present study) and Okinawa (Matsumura and Uchida 1926; Uchida 1928; Chiu 1954; present study). *New records.

##### Bionomics.

Reared from two species of Noctuidae in Japan: *Sesamia turpis* (Butler) (Kusigemati 1972; Nagatomi et al. 1972) and *S. inferens* (Walker) (e.g., Uchida 1928, 1930; Sonan 1944; Nagatomi et al. 1972; Momoi and Watanabe 1975). This species is frequently collected in sugar cane fields where it is a parasitoid of *Sesamia* Guenée species (Moutia 1934; Nagatomi et al. 1972).

##### Differential diagnosis.

**Figure 41.**
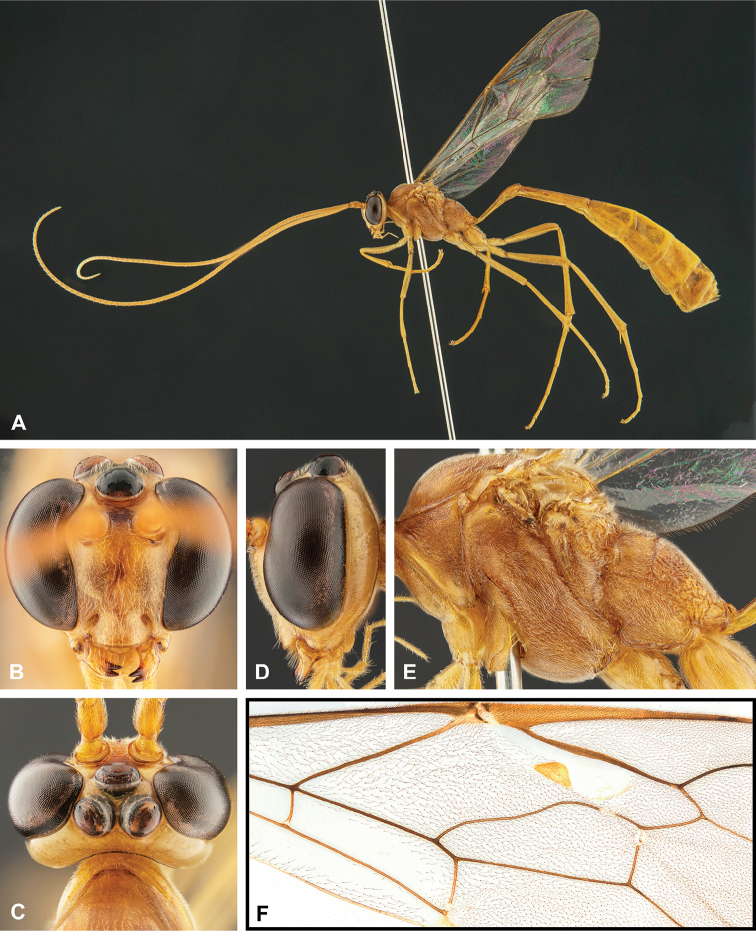
*Enicospilus sakaguchii* (Matsumura & Uchida, 1926) ♀ from Japan **A** habitus **B** head, frontal view **C** head, dorsal view **D** head, lateral view **E** mesosoma, lateral view **F** central part of fore wing.

This species is very easily distinguished from all other species of *Enicospilus* by the shape of the clypeus (flat and projecting, with a distinct gap between clypeus and mandibles in profile, as in Fig. 41B, D), mandible (short and stout, as in Fig. 41B, D), and fore wing sclerites (Fig. 41F).

##### Remarks.

We could find no morphological differences between *E. sakaguchii* and *E. iracundus* syn. nov., except the faint presence or absence of the central sclerite. Hence, *E. iracundus* syn. nov. is newly synonymised with *E. sakaguchii*.

#### Enicospilus sauteri

Taxon classificationAnimaliaHymenopteraIchneumonidae

(Enderlein, 1921)

33758E35-C652-5835-8E5D-EA7062044710

Figure 42

Henicospilus sauteri Enderlein, 1921: 84; HT ♀ from Taiwan, IZPAN, not examined.
Henicospilus analis Matsumura and Uchida, 1926: 72; LCT ♀ from Ryûkyûs, designated by Gauld and Mitchell (1981: 374), SEHU, missing so not examined; synonymised by Gauld and Mitchell (1981: 374).
Enicospilus molopos Chiu, 1954: 57; HT ♀ from Taiwan, TARI, examined; synonymised by Gauld and Mitchell (1981: 374).

##### Specimens examined.

Total of 9 specimens (6♀♀3♂♂): Laos (4♀♀1♂), Taiwan (2♀♀2♂♂). No Japanese specimens available.

Type series: HT ♀ of *Enicospilus molopos* Chiu, 1954, Rengechi, TAIWAN, 9.X.1935, S. Isshiki leg. (TARI); AT ♂ of *Enicospilus molopos*, Kappanzan, TAIWAN, 18.V.1930, J. Sonan leg. (TARI).

##### Distribution.

Eastern Palaearctic and Oriental regions (Yu et al. 2016); this is a predominantly Oriental species.

JAPAN: [Ryûkyûs] Okinawa (Matsumura and Uchida 1926; Uchida 1928; Sonan 1940; Momoi 1970).

##### Bionomics.

Unknown.

##### Differential diagnosis.

**Figure 42.**
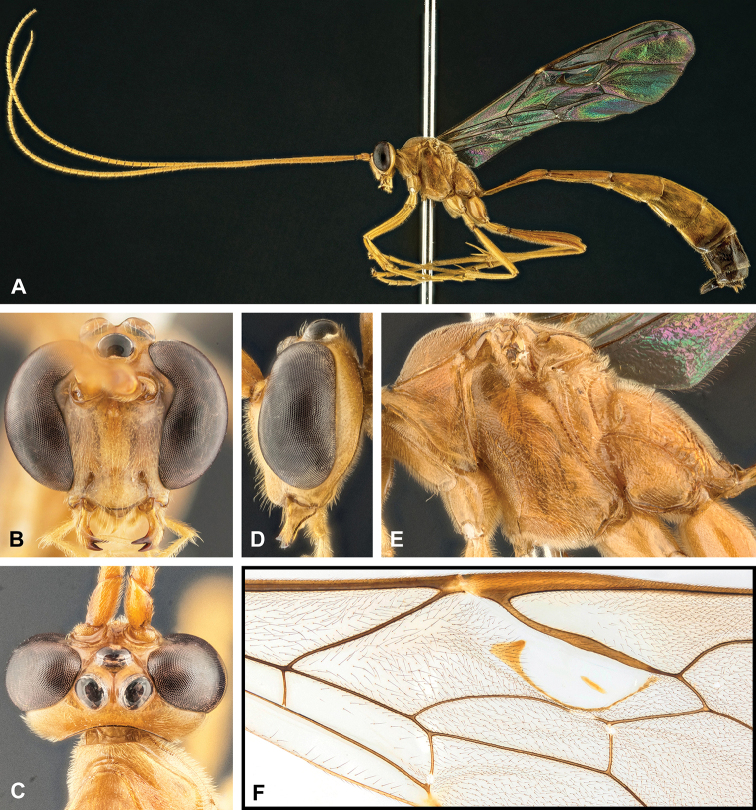
*Enicospilus sauteri* (Enderlein, 1921) ♂ from Taiwan **A** habitus **B** head, frontal view **C** head, dorsal view **D** head, lateral view **E** mesosoma, lateral view **F** central part of fore wing.

This species resembles *E. melanocarpus* but can be distinguished by the presence of a glabrous area in the proximal part of the fore wing marginal cell (Fig. 42F) (marginal cell uniformly setose in *E. melanocarpus*, as in Fig. 28F) and linear central sclerite (Fig. 42F) (central sclerite usually circular to oval in *E. melanocarpus*, as in Fig. 28F) (also see Table 8).

##### Remarks.

Gauld and Mitchell (1981) designated the lectotype of *Henicospilus analis* based on the Ryûkyûs specimen. However, the lectotype label of *H. analis* was pinned to a Taiwanese specimen of *E. sauteri*. Although the type series (1♀2♂♂) were all collected in Ryûkyûs (Matsumura and Uchida 1926), the author could not find any Japanese specimens of this species in the ichneumonid collection at SEHU. Therefore, the lectotype of *H. analis* is currently missing. However, given the similarity in label data between the missing Japanese specimens of *H. analis* and the type of *Allocamptus orientalis*, which had also been mislabelled, we strongly suspect that I. D. Gauld confused the data of *H. analis* and *A. orientalis*, and that it is unlikely that *Enicospilus sauteri* has been collected in Japan.

#### Enicospilus sharkeyi

Taxon classificationAnimaliaHymenopteraIchneumonidae

Shimizu sp. nov.

ABDD718A-06AF-5688-BFB3-FEF8D94D9303

http://zoobank.org/A136126B-EA7A-4F7F-978B-F00D4A7BED5E

Figure 43

##### Etymology.

The specific name is dedicated to Dr Michael Sharkey (University of Kentucky Lexington, Kentucky, USA) who collected many of the type series.

##### Type series.

Total of 25 specimens (15♀♀10♂♂): Japan (15♀♀10♂♂).

HT: ♀, Mt. Tsurugi-yama (33°51'50.8"N, 134°04'42.4"E, 1,250 m alt.), Higashiiyasugeoi, Miyoshi City, Tokushima Pref., Shikoku, JAPAN, 22.VIII.2015, Y. Kitayama leg. (LT) (MNHA, SEN145–DDBJ-LC486393).

PT: 1♀, Hitsujigaoka (43°00'N 141°24'E), Sapporo City, Hokkaidô, JAPAN, 18–25.VIII.2008, K. Konishi leg. (MsT) (EUM, SEN33–DDBJ-LC486392); 2♀♀, Hitsujigaoka (43°00'N 141°24'E), Sapporo City, Hokkaidô, JAPAN, 14–21.VII.2008, K. Konishi leg. (MsT) (EUM); 2♀♀, Kamegai (330 m alt.), Toyama City, Toyama Pref., Hokuriku, JAPAN, 28.VII–4.VIII.2009 (1♀), 25.VIII–1.IX.2009 (1♀), M. Watanabe leg. (MsT) (KPMNH); 2♂♂, Kiyotaki, Nikkô City, Tochigi Pref., Kantô-Kôshin, JAPAN, 8.VII.1977 (LT) (NIAES); 1♂, Shirane-onsen, Katashina Vil., Gunma Pref., Kantô-Kôshin, JAPAN, 12.VII.1991, M. Yoshida leg. (LT) (NIAES);1♀, Mt. Fujiwaradake, Mie Pref., Tôkai, JAPAN, 3.IX.1996, A. Kawazoe leg. (KPMNH); 1♀, Takaosan, Kyôto Pref., Kinki, JAPAN, 1.X.1958 (MNHA-SMCM); 1♀, Kogagawa, Wakayama Pref., Kinki, JAPAN, 20.IX.1957, S. Momoi leg. (MNHA-SMCM); 1♂, Teragawa, Hongawa, Kôchi Pref., Shikoku, JAPAN, 25.VII.1991, I. Yamashita leg. (NIAES); 3♀♀4♂♂, Mt. Hiko (700 m alt.), Fukuoka Pref., Kyûshû, JAPAN, 29.V–9.VI.1989 (1♂), 9.VI.1989 (1♂), 29.VI–10.VII.1989 (2♂♂), 10–20.VII.1989 (1♀), 11–18.IX.1989 (1♀), 18–25.IX.1989 (1♀), K. Takeno & M. Sharkey leg. (EMUS); 2♀♀1♂, Yufuin, Ōita Pref., Kyûshû, JAPAN, 4.VII.1981, S. Yoshimatsu leg. (NIAES); 1♀, Hacchobaru, Mts. Kujû, Ōita Pref., Kyûshû, JAPAN, 14.VII.1984, S. Yoshimatsu leg. (LT) (NIAES); 1♂, Yunoo, Kirishima, Kagoshima Pref., Kyûshû, JAPAN, 3.VII.1958, H. Maebara leg. (MNHA-SMCM).

##### Distribution.

Eastern Palaearctic region.

JAPAN: [Hokkaidô]; [Hokuriku] Toyama; [Kantô-Kôshin] Tochigi and Gunma; [Tôkai] Mie; [Kinki] Kyôto and Wakayama; [Shikoku] Tokushima and Kôchi; [Kyûshû] Fukuoka, Ōita, and Kagoshima.

##### Bionomics.

Unknown.

##### Differential diagnosis.

**Figure 43.**
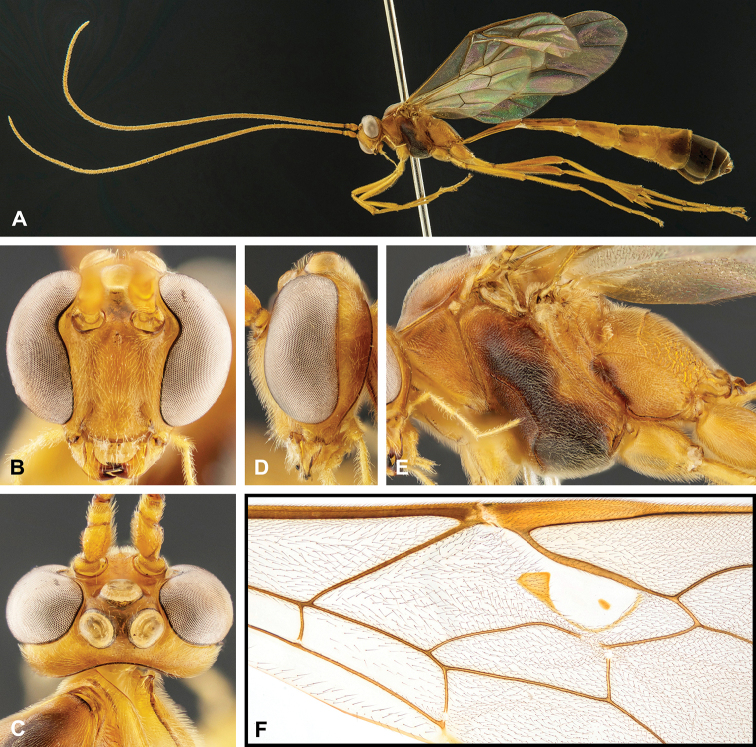
*Enicospilus sharkeyi* Shimizu, sp. nov. ♀ (**A–E**HT**F**PT) from Japan **A** habitus **B** head, frontal view **C** head, dorsal view **D** head, lateral view **E** mesosoma, lateral view **F** central part of fore wing.

This species has a very similar colour pattern to *E. combustus* and some specimens have been misidentified as *E. combustus*. The two species can be distinguished by many characters, such as (in *E. sharkeyi* sp. nov.) shorter and stouter mandible (Fig. 43B, D), confluent proximal and distal sclerites (Fig. 43F), smaller central sclerites (Fig. 43F), etc. *Enicospilus sharkeyi* sp. nov. also resembles *E. ramidulus* and *E. melanocarpus* in morphology, but can readily be separated by the darker mesosoma (Fig. 43A, E), narrower face (Fig. 43B), etc.

##### Description.

Female (n = 15). Body length 19.0–22.5 (HT: ca. 21.0) mm.

Head with GOI = 2.3–2.6 (HT: 2.5) (Fig. 43D). Lower face 0.7–0.8× (HT: 0.8) as wide as high, strongly shiny, punctate with rather long setae (Fig. 43B, D). Clypeus 1.4–1.7× (HT: 1.6) as wide as high, punctate with setae, entirely moderately convex in profile, ventral margin impressed (Fig. 43B, D). Malar space 0.3–0.4× (HT: 0.3) as long as basal mandibular width (Fig. 43B, D). Mandible moderately twisted by 20–30° (HT: ca. 25°), moderately long, evenly narrowed, outer surface with diagonal setose groove (Fig. 43B, D). Upper tooth of mandible 1.5–1.7× (HT: 1.5) as long as lower (Fig. 43B). Frons, vertex, and gena strongly shiny with fine setae (Fig. 43B–D). Posterior ocellus close to eye (Fig. 43B–D). Ventral end of occipital carina joining oral carina. Antennae with 56–60 (HT: 58) flagellomeres; first flagellomere 1.6–1.8× (HT: 1.8) as long as second; 20^th^ flagellomere 2.3–2.8× (HT: 2.3) as long as wide.

Mesosoma entirely shiny with setae (Fig. 43E). Pronotum diagonally wrinkled to punctate (Fig. 43E). Mesoscutum 1.4–1.5× (HT: 1.5) as long as maximum width, moderately shiny and finely punctate to smooth with setae, and evenly rounded in profile (Fig. 43E). Notauli absent (Fig. 43E). Scutellum moderately convex, smooth, with lateral longitudinal carinae along anterior 0.7–1.0 (HT: 1.0) of scutellum. Epicnemium densely punctate with setae. Epicnemial carina slightly curved and inclined to anterior, dorsal end close to anterior margin of mesopleuron but not reaching (Fig. 43E). Mesopleuron entirely longitudinally wrinkled to punctostriate (Fig. 43E). Submetapleural carina weakly broadened anteriorly (Fig. 43E). Metapleuron densely punctate (Fig. 43E). Propodeum evenly rounded in profile; anterior transverse carina complete; anterior area longitudinally striate; spiracular area finely punctate with fine setae; posterior area moderately wrinkled; propodeal spiracle elliptical and not joining pleural carina by ridge (Fig. 43E).

Wings (Fig. 43F). Fore wing length 12.0–13.5 (HT: ca. 13.0) mm with AI = 0.3–0.4 (HT: 0.4), CI = 0.3–0.4 (HT: 0.4), DI = 0.4, ICI = 0.3–0.4 (HT: 0.4), SDI = 1.2–1.3 (HT: 1.3), SI = 0.1, SRI = 0.3; vein 1m-cu&M slightly sinuous; vein 2r&RS almost straight; vein RS evenly curved; fenestra and sclerites of discosubmarginal cell as in Fig. 43F; proximal sclerite triangular, strongly pigmented, confluent with strongly pigmented distal sclerite; central sclerite suboval, small and less than 1.0× as wide as maximum thickness of 2r&RS, strongly pigmented, and positioned in medio-distal part of fenestra; proximal corner of marginal cell evenly setose; posterodistal corner of second discal cell 70–85° (HT: ca. 85°) and of subbasal cell 85–90° (HT: ca. 85°); vein 1cu-a subinterstitial or antefurcal to M&RS by less than 0.2× length of 1cu-a (HT: antefurcal by 0.2×). Hind wing with NI = 1.1–1.4 (HT: 1.4); vein RS basally slightly bowed and straight; vein RA with 6–8 (HT: 8) uniform hamuli.

Legs. Hind leg with coxa in profile 1.7–1.8× (HT: 1.8) as long as deep; basitarsus 2.0–2.1× (HT: 2.0) as long as second tarsomere; fourth tarsomere 4.3–5.0× (HT: 5.0) as long as wide; tarsal claw simply pectinate.

Metasoma with DMI = 1.3–1.4 (HT: 1.4), PI = 3.0–3.2 (HT: 3.1), THI = 3.0–3.3 (HT: 3.2); thyridium oval and rather small; ovipositor sheath not longer than posterior depth of metasoma (Fig. 43A).

Colour (Fig. 43). Entirely testaceous except for apex of mandible, mesoscutum, mesopleuron, and T6–8 blackish. Wings weakly infuscate; sclerites pigmented and amber; veins red-brown.

Male (n = 10). Very similar to female.

##### Remarks.

This species is morphologically very stable except that the mesosoma varies from entirely dark to reddish.

#### Enicospilus shikokuensis

Taxon classificationAnimaliaHymenopteraIchneumonidae

(Uchida, 1928)

980AD80D-8005-51CB-A63B-2CCD5EAB0E56

Figure 44

Henicospilus combustus var. shikokuensis Uchida, 1928: 224; LCT ♀ from Japan, designated by Townes et al. (1965: 334), SEHU, examined.
Enicospilus seniculus Chiu, 1954: 71; HT ♀ from Korea, TARI, examined; synonymised by Gauld and Mitchell (1981: 375).
Enicospilus sigmatoides Chiu, 1954: 75; HT ♂ from Korea, TARI, examined; **syn. nov.**

##### Specimens examined.

Total of 93 specimens (36♀♀55♂♂ and 2 unsexed): Japan (35♀♀54♂♂ and 2 unsexed), Korea (1♀1♂).

Type series: LCT ♀ of Henicospilus combustus var. shikokuensis Uchida, 1928, Ehime, Shikoku, JAPAN, 9.V.1924, Tsushima leg. (SEHU); HT ♀ of *Enicospilus seniculus* Chiu, 1954, Suigen, KOREA, IV–VI.1927, K. Sato leg. (TARI); HT ♂ of *Enicospilus sigmatoides* Chiu, 1954, Suigen, KOREA, 24.IV.1930, K. Sato leg. (TARI).

##### Distribution.

Eastern Palaearctic and Oriental regions (Yu et al. 2016); this is a predominantly Eastern Palaearctic species.

JAPAN: [Hokkaidô]*; [Hokuriku] Niigata (Ohmori 1960; present study); [Kantô-Kôshin] Tochigi (Katayama et al. 2010), Saitama*, Tôkyô (Chiu 1954; Konishi and Maeto 2000; Konishi et al. 2014; present study), and Kanagawa (Chiu 1954; Watanabe et al. 2016; present study); [Tôkai] Shizuoka* and Mie (Uchida 1928); [Kinki] Kyôto (Uchida 1928) and Hyôgo*; [Chûgoku] Shimane*, Okayama* and Hiroshima (Konishi and Nakamura 2002, 2010; Maeto 2003; present study); [Shikoku] Tokushima*, Ehime (Uchida 1928; Konishi and Yamamoto 2000; present study) and Kôchi*; [Kyûshû] Nagasaki*, Kumamoto*, Miyazaki* and Kagoshima*; [Ryûkyûs] Kagoshima (Watanabe 2018; present study) and Okinawa*. *New records.

##### Bionomics.

No host records from Japan. *Enicospilus shikokuensis* is one of the most frequently collected ichneumonids in spring in Japan, and it seems to be univoltine.

##### Differential diagnosis.

**Figure 44.**
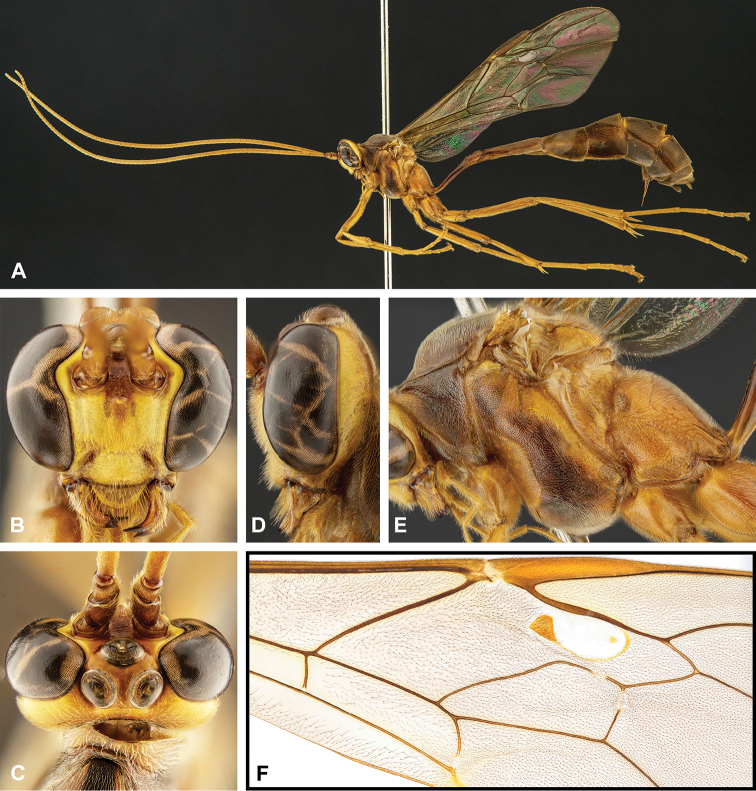
*Enicospilus shikokuensis* (Uchida, 1928) ♀ from Japan **A** habitus **B** head, frontal view **C** head, dorsal view **D** head, lateral view **E** mesosoma, lateral view **F** central part of fore wing.

As mentioned in the diagnosis of *E. multidens* stat. rev., this species is sometimes confused with *E. multidens* stat rev., but can be distinguished by the characters listed in the diagnosis section of *E. multidens* stat. rev. Gauld and Mitchell (1981) compared *E. shikokuensis* to *E. ramidulus*, but *E. shikokuensis* is easily distinguishable by the much wider lower face (Fig. 44B), longer and slenderer mandible (Fig. 44B, D), larger size, etc.

##### Remarks.

This species exhibits a wide range of colour variation from entirely testaceous to dark brown or black. Paler individuals have the proximal and distal sclerites separated and the central sclerite weak, so it is likely that the degree of melanisation has an effect on the sclerite development as well as the colour. The holotype of *Enicospilus sigmatoides* Chiu, syn. nov. is a paler individual with separated proximal and distal sclerites. DNA barcodes of individuals spanning the morphological continuum varied by less than 1%.

#### Enicospilus shinkanus

Taxon classificationAnimaliaHymenopteraIchneumonidae

(Uchida, 1928)

5847E5BA-E34C-5C50-B484-2C4FDC71A377

Figure 45

Henicospilus shinkanus Uchida, 1928: 217; HT ♀ from Taiwan, SEHU, examined.
Henicospilus yamanakai Uchida, 1930: 83; HT ♀ from Japan, SEHU, examined; **syn. nov.**
Henicospilus pankumensis Cheesman, 1936: 184; HT ♀ from Vanuatu, NHMUK, examined; synonymised by Gauld and Mitchell (1981: 361).
Enicospilus relictus Chiu, 1954: 20; HT ♀ from Taiwan, TARI, examined; synonymised by Gauld and Mitchell (1981: 361).
Enicospilus (Unicorniata) bindus Nikam, 1972: 194; HT ♀ from India, MUC, not examined; synonymised by Gauld and Mitchell (1981: 361).

##### Specimens examined.

Total of 87 specimens (64♀♀21♂♂ and 2 unsexed): Chagos archipelago (2♀♀10♂♂ and 2 unsexed), India (40♀♀5♂♂), Japan (18♀♀3♂♂), Solomon Islands (1♀1♂), Taiwan (2♀♀2♂♂), Vanuatu (1♀).

Type series: HT ♀ of *Henicospilus shinkanus* Uchida, 1928, Sugar Ex. St., TAIWAN (SEHU); HT ♀ of *Henicospilus yamanakai* Uchida, 1930, Izuôshima Is., Tôkyô, Kantô-Kôshin, JAPAN, 10.IX.1926, M. Yamanaka leg. (SEHU); HT ♀ of *Enicospilus relictus* Chiu, 1954, Kotosho, TAIWAN, III–IV.1932, S. Hirayama leg. (TARI); HT ♀ of *Henicospilus pankumensis* Cheesman, 1936, Santo, New Hebrides [= VANUATU], VIII–IX.1929, L.E. Cheesman leg. (NHMUK, Type 3b.1239).

##### Distribution.

Australasian, Eastern Palaearctic, Oceanic, and Oriental regions (Yu et al. 2016).

JAPAN: [Hokkaidô]*; [Hokuriku] Niigata*; [Kantô-Kôshin] Tôkyô*; [Tôkai] Shizuoka*; [Chûgoku] Hiroshima*; [Kyûshû] Kagoshima*; [Ryûkyûs] Kagoshima* and Okinawa (Chiu 1954; Momoi 1970; present study). *New records.

##### Bionomics.

No host records from Japan. Reported as a parasitoid of *Dendrolimus punctatus* (Walker) (Lasiocampidae) in China (Tang 1990).

##### Differential diagnosis.

**Figure 45.**
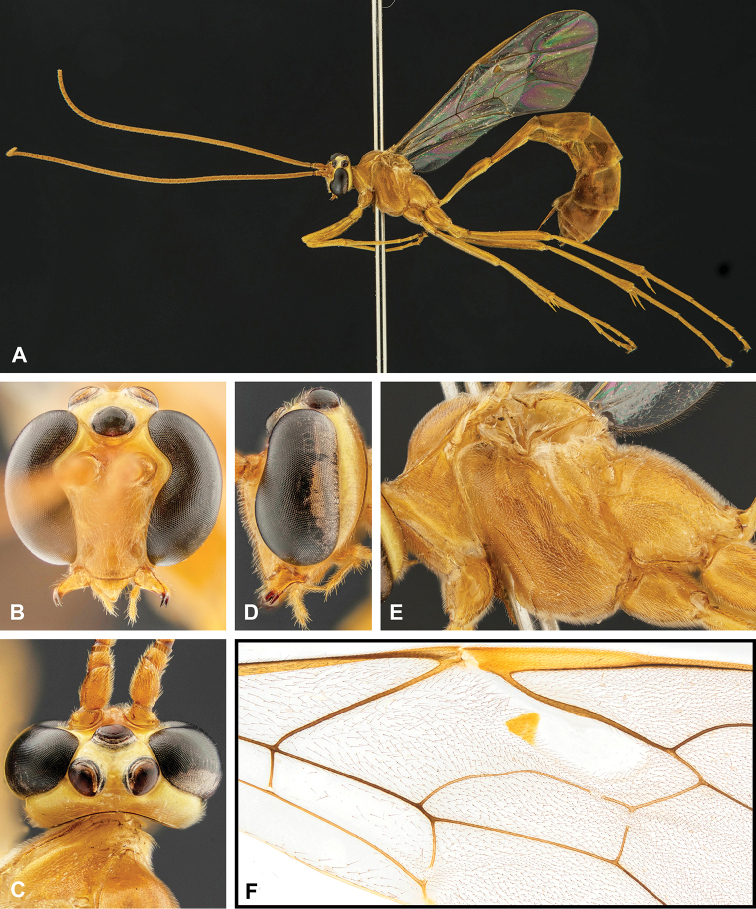
*Enicospilus shinkanus* (Uchida, 1928) ♀ from Japan **A** habitus **B** head, frontal view **C** head, dorsal view **D** head, lateral view **E** mesosoma, lateral view **F** central part of fore wing.

According to Gauld and Mitchell (1981), this species is very similar to *E. rufus* (Brullé, 1846), but distinguished by its longer fore wing fenestra (Fig. 45F). It is also sometimes confused with *E. sakaguchii* due to their similar clypeus shape (i.e., flat, projecting apically above mandibles in profile, as in Figs 41D and 45D) and absence of the fore wing central sclerite (Figs 41F, 45F), but easily distinguished by many mandibular characters, such as the mandible rather long in *E. shinkanus* (Fig. 45B, D) but very short and stout in *E. sakaguchii* (Fig. 41B, D); and outer mandibular surface smooth in *E. shinkanus* (Fig. 45D) but with a diagonal setose groove in *E. sakaguchii* (Fig. 41B, D).

##### Remarks.

The holotype of *Henicospilus yamanakai* Uchida, 1930 was examined and identified as *E. shinkanus*. Hence, *H. yamanakai* syn. nov. is newly synonymised with *E. shinkanus*.

#### Enicospilus signativentris

Taxon classificationAnimaliaHymenopteraIchneumonidae

(Tosquinet, 1903)

514CC11C-D440-5A04-92D2-B907F7C538C3

Figure 46

Ophion (Enicospilus) signativentris Tosquinet, 1903: 37; LCT ♀ from Java, designated by Townes et al. (1961: 289), IRSNB, not examined.
Henicospilus incompletus Szépligeti, 1906: 143; HT ♀ from Sulawesi, TM, not examined; synonymised by Gauld and Mitchell (1981: 411).
Henicospilus nigrosignatus Enderlein, 1921: 22; HT ♀ from Java, IZPAN, not examined; synonymised by Townes et al. (1961: 289).
Henicospilus tristrigatus Enderlein, 1921: 23; HT ♀ from Taiwan, IZPAN, not examined; synonymised by Gauld and Mitchell (1981: 411).
Henicospilus formosanus Enderlein, 1921: 25; HT ♀ from Taiwan, IZPAN, not examined; synonymised by Gauld and Mitchell (1981: 411).
Henicospilus emacescens Enderlein, 1921: 25; HT ♂ from Taiwan, IZPAN, not examined; synonymised by Gauld and Mitchell (1981: 411).
Henicospilus taiwanus Uchida, 1928: 226; SYT ♀ from Taiwan, SEHU, examined; synonymised by Gauld and Mitchell (1981: 411).
Enicospilus frater Cushman, 1937: 311; HT ♂ from Taiwan, DEI, not examined; synonymised by Gauld and Mitchell (1981: 411).
Enicospilus pectiniclavae Rao and Nikam, 1969: 14; HT ♀ from India, MUC, not examined; synonymised by Gauld and Mitchell (1981: 411).

##### Specimens examined.

Total of 208 specimens (154♀♀46♂♂ and 8 unsexed): India (39♀♀17♂♂), Indonesia (7♀♀5♂♂ and 3 unsexed), Japan (95♀♀23♂♂), Sri Lanka (1♀), Taiwan (12♀♀1♂ and 5 unsexed).

Type series: SYT ♀ of *Henicospilus taiwanus* Uchida, 1928, Kyuhabon, TAIWAN, 6.VIII.1915, K. Kikuchi leg. (SEHU).

##### Distribution.

Australasian, Eastern Palaearctic, Oceanic, and Oriental regions (Yu et al. 2016); this is a predominantly Oriental species.

JAPAN: [Kantô-Kôshin] Tôkyô (Konishi and Maeto 2000; Konishi et al. 2014; present study) and Kanagawa (Watanabe et al. 2016; present study); [Tôkai] Shizuoka (Watanabe and Makanai 2011; present study), Gifu (Chiu 1954), and Mie*; [Kinki] Ōsaka*, Hyôgo (Iwata 1960; present study), Nara (Iwata 1958, 1960), and Wakayama*; [Chûgoku] Shimane* and Hiroshima (Konishi and Nakamura 2000, 2010; Maeto and Shimizu 2019; present study); [Shikoku] Ehime (Konishi and Yamamoto 2000; present study) and Kôchi*; [Kyûshû] Fukuoka*, Ōita*, Kumamoto* and Kagoshima (Yasumatsu 1934; Chiu 1954; Fukuda and Kusigemati 1986; present study); [Ryûkyûs] Kagoshima (Uchida 1956; present study) and Okinawa (Chiu 1954; Townes 1958; present study); [Ogasawara] Tôkyô (Townes 1958; Takahashi and Shimizu 2006; present study). *New records. This is one of the most common *Enicospilus* species in Japan and peripheral areas.

##### Bionomics.

Japanese host records are from several species of plusiine Noctuidae and Erebidae: *Anadevidia peponis* (Fabricius, 1775) (Kusigemati and Tanaka 1992), *Autographa nigrisigna* (Walker, 1857) (Kusigemati 1976), *Trichoplusia intermixta* (Warren, 1913) (Kusigemati 1981) (all Noctuidae), and *Ericeia inangulata* (Guenée, 1852) (Erebidae) (Sonan 1944).

##### Differential diagnosis.

**Figure 46.**
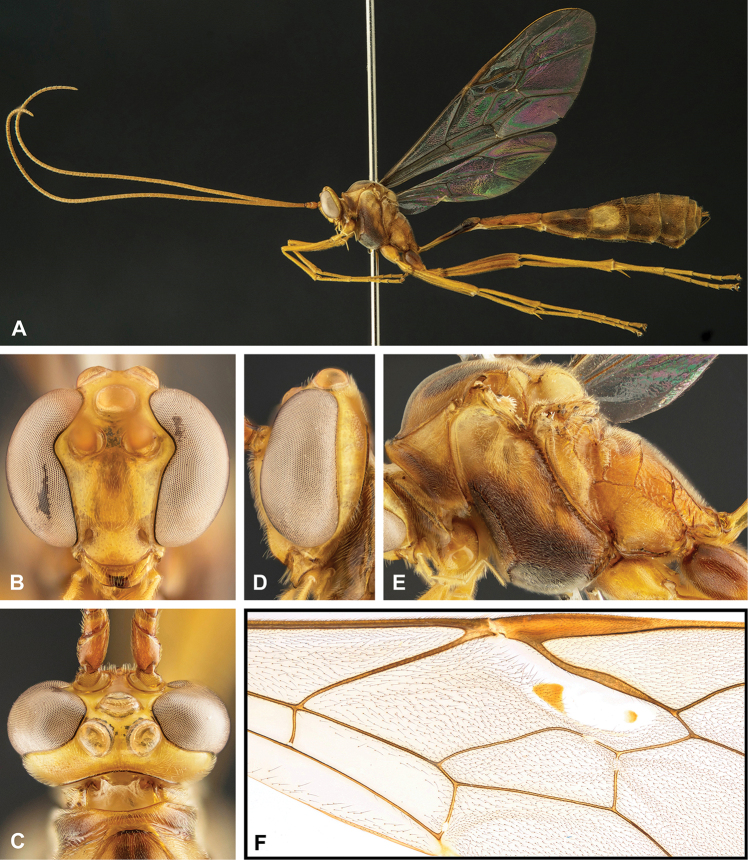
*Enicospilus signativentris* (Tosquinet, 1903) ♀ from Japan **A** habitus **B** head, frontal view **C** head, dorsal view **D** head, lateral view **E** mesosoma, lateral view **F** central part of fore wing.

This species is morphologically close to *E. abdominalis* but can easily be distinguished from it, and also from all other Japanese species, by the strong posterior transverse carina of the propodeum (Fig. 46E) and characteristic colour pattern (T4 is usually conspicuously brighter than adjacent segments) (Fig. 46A).

##### Remarks.

*Enicospilus signativentris* is more or less morphologically stable, although it exhibits a very wide range of colour variation (i.e., from entirely orange to entirely dark brown). DNA barcoding analysis supports the conclusion that variable body colour represents intraspecific variation. There was no difference of p-distance between the entirely testaceous (SEN97 from Ōsaka) and the entirely dark brown individuals (SEN98 from Wakayama).

#### Enicospilus stenophleps

Taxon classificationAnimaliaHymenopteraIchneumonidae

Cushman, 1937

8D574209-4044-54F6-94EA-E7853C2E738D

Figure 47

Enicospilus stenophleps Cushman, 1937: 309; HT ♀ from Taiwan, DEI, not examined.

##### Specimens examined.

Total of 7 specimens (5♀♀1♂ and 1 unsexed): Japan (2♀♀1♂), Sri Lanka (2♀♀ and 1 unsexed), Taiwan (1♀).

##### Distribution.

Oriental region (Yu et al. 2016).

Newly recorded from Japan.

JAPAN: [Ryûkyûs] Okinawa.

##### Bionomics.

Unknown.

##### Differential diagnosis.

**Figure 47.**
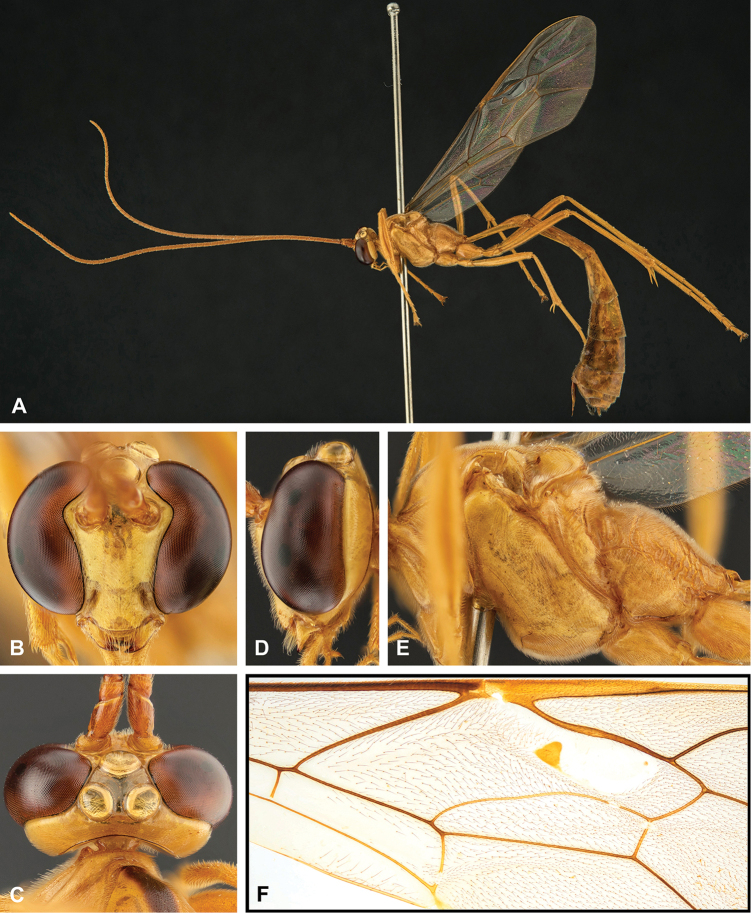
*Enicospilus stenophleps* Cushman, 1937 ♀ from Japan **A** habitus **B** head, frontal view **C** head, dorsal view **D** head, lateral view **E** mesosoma, lateral view **F** central part of fore wing.

*Enicospilus stenophleps* can be readily distinguished from all other species of *Enicospilus* by the characteristic very small circular central sclerite and wide fenestra, as in Fig. 47F, although *E. stenophleps* is closely related to *E. vestigator* and *E. nigribasalis*.

#### Enicospilus takakuwai

Taxon classificationAnimaliaHymenopteraIchneumonidae

Shimizu sp. nov.

D38784CF-1CA4-51CB-BC20-5286DEDB654E

http://zoobank.org/761D3C15-1EDF-4D58-AE38-FA778F7E9087

Figure 48

##### Etymology.

The specific name is derived from the name of the collector of the holotype specimen, a famous Japanese insect taxonomist, Masatoshi Takakuwa.

##### Type series.

A holotype male only.

HT: ♂, Tairoike, Miyake-jima, Izu Iss., Tôkyô, Kantô-Kôshin, JAPAN, 10–11.VI.2010, M. Takakuwa leg. (KPMNH).

##### Distribution.

Eastern Palaearctic region.

JAPAN: [Kantô-Kôshin] Tôkyô (Miyake-jima Island).

##### Bionomics.

Unknown.

##### Differential diagnosis.

**Figure 48.**
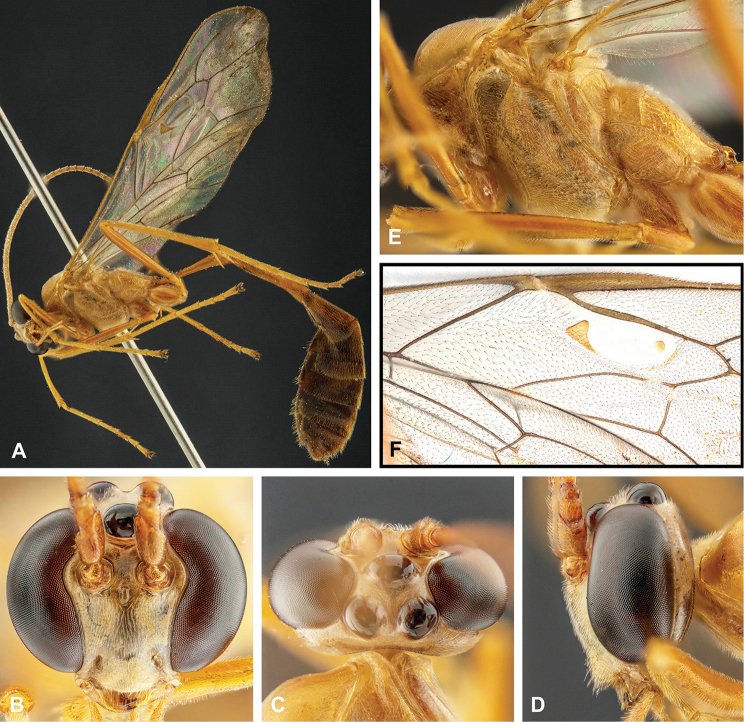
*Enicospilus takakuwai* Shimizu, sp. nov. ♂ (HT) from Japan **A** habitus **B** head, frontal view **C** head, dorsal view **D** head, lateral view **E** mesosoma, lateral view **F** central part of fore wing.

This species is more or less similar to *E. laqueatus*, but easily distinguished by the position and shape of the central sclerite: central sclerite positioned in the anterodistal part of the fenestra, smaller and moderately sclerotised in *E. takakuwai* sp. nov., as in Fig. 48F, but positioned in the centrodistal part of the fenestra, larger and strongly sclerotised in *E. laqueatus*, as in Fig. 24F.

##### Description.

Male (HT). Body length ca. 16.5 mm.

Head with GOI = 3.2 (Fig. 48D). Lower face 0.8× as wide as high, strongly shiny, finely punctate with setae (Fig. 48B, D). Clypeus 1.5× as wide as high, finely punctate with setae, convex in profile, and ventral margin acute (Fig. 48B, D). Malar space 0.4× as long as basal mandibular width (Fig. 48B, D). Mandible moderately twisted by 15°, moderately long, evenly narrowed, outer surface with diagonal setose groove (Fig. 48B, D). Upper tooth of mandible 1.4× as long as lower (Fig. 48B). Frons, vertex and gena strongly shiny with fine setae (Fig. 48B–D). Posterior ocellus touching eye (Fig. 48B–D). Ventral end of occipital carina joining oral carina. Antennae with 59 flagellomeres; first flagellomere 1.8× as long as second; 20^th^ flagellomere 2.1× as long as wide.

Mesosoma entirely rather weakly shiny with fine setae (Fig. 48E). Pronotum fairly extensively diagonally striate (Fig. 48E). Mesoscutum 1.5× as long as maximum width, finely punctate, and evenly rounded in profile (Fig. 48E). Notauli absent (Fig. 48E). Scutellum with lateral longitudinal carinae along entire length of scutellum. Epicnemium punctate with setae. Epicnemial carina strong, almost straight, inclined to anterior, dorsal end not reaching anterior margin of mesopleuron (Fig. 48E). Meso- and metapleuron evenly moderately longitudinally striate (Fig. 48E). Submetapleural carina broadened anteriorly (Fig. 48E). Propodeum declivous in profile; anterior transverse carina complete; anterior area longitudinally striate; spiracular area smooth; posterior area entirely moderately reticulate; propodeal spiracle elliptical and joining pleural carina by ridge (Fig. 48E).

Wings (Fig. 48F). Fore wing length ca. 11.0 mm with AI = 0.4, CI = 0.4, DI = 0.3, ICI = 0.5, SDI = 1.3, SI = 0.1, SRI = 0.3; vein 1m-cu&M evenly weakly curved; vein 2r&RS almost straight; vein RS evenly curved; fenestra and sclerites of discosubmarginal cell as in Fig. 48F; proximal sclerite triangular, not confluent with distal sclerite, strongly pigmented; central sclerite D-shaped, moderately sclerotised, and positioned in anterodistal part of fenestra; distal sclerite weakly pigmented; proximal corner of marginal cell rather sparsely setose, but without glabrous area; posterodistal corner of second discal cell ca. 100° and of subbasal cell ca. 90°; vein 1cu-a antefurcal to M&RS by 0.2× length of 1cu-a. Hind wing with NI = 2.3; vein RS straight; vein RA with 7 uniform hamuli.

Legs. Hind leg with coxa in profile 1.7× as long as deep; basitarsus 1.9× as long as second tarsomere; fourth tarsomere 3.3× as long as wide; tarsal claw simply pectinate.

Metasoma with DMI = 1.4, PI = 3.3, THI = 4.5; thyridium oval.

Colour (Fig. 48). Head and mesosoma entirely yellow-brown except for apex of mandible black. Metasoma dark brown. Wings hyaline. Fore wing sclerites pigmented and amber. Wing veins red-brown to amber.

Female. Unknown.

#### Enicospilus tripartitus

Taxon classificationAnimaliaHymenopteraIchneumonidae

Chiu, 1954

47F1FED2-5261-5761-8359-E4F965015860

Figure 49

Enicospilus tripartitus Chiu, 1954: 36; HT ♀ from Taiwan, TARI, examined.

##### Specimens examined.

Total of 67 specimens (39♀♀26♂♂ and 2 unsexed): China (1♀), India (1♂), Japan (12♀♀14♂♂ and 1 unsexed), Nepal (24♀♀8♂♂ and 1 unsexed), Taiwan (2♀♀), unknown (3♂♂).

Type series: HT ♀ of *Enicospilus tripartitus* Chiu, 1954, Taihoku, TAIWAN, 27.VIII.1937, J. Sonan leg. (TARI); PT ♂ of *E. tripartitus*, no data (NHMUK).

##### Distribution.

Eastern Palaearctic and Oriental regions (Yu et al. 2016).

JAPAN: [Tôhoku] Miyagi*; [Hokuriku] Niigata* and Ishikawa*; [Kantô-Kôshin] Kanagawa*; [Tôkai] Shizuoka*, Aichi*, and Mie*; [Chûgoku] Shimane* and Hiroshima (Maeto and Shimizu 2019; present study); [Shikoku] Tokushima* and Kôchi*; [Kyûshû] Ōita (Chiu 1954); [Ryûkyûs] Kagoshima* and Okinawa (Chiu 1954; Shimizu 2020; present study). *New records.

##### Bionomics.

Unknown.

##### Differential diagnosis.

**Figure 49.**
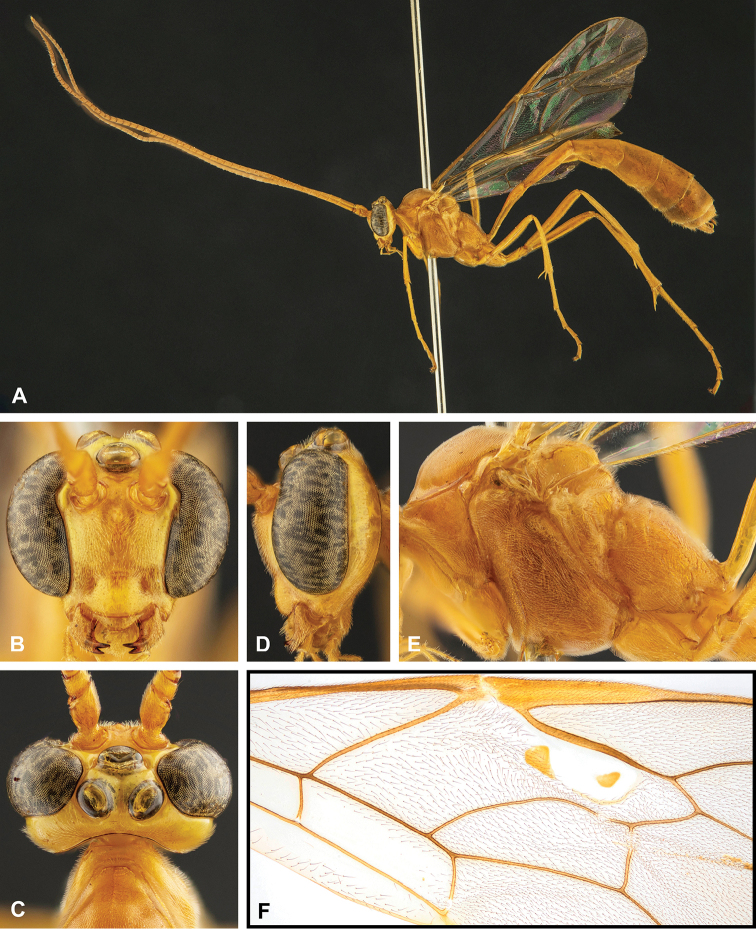
*Enicospilus tripartitus* Chiu, 1954 ♂ from Japan **A** habitus **B** head, frontal view **C** head, dorsal view **D** head, lateral view **E** mesosoma, lateral view **F** central part of fore wing.

This species resembles *E. laqueatus*, *E. pseudantennatus*, and *E. vestigator* in the shapes of the sclerites, but can easily be distinguished by the dense and stout setae and punctures of the outer mandibular surface (Fig. 49B, D), deep basal concavity of the outer mandibular surface (Fig. 49B, D), etc., as summarised in Table 6.

#### Enicospilus unctus

Taxon classificationAnimaliaHymenopteraIchneumonidae

Shimizu sp. nov.

23B905CC-6473-5742-B81A-9D0E55F13058

http://zoobank.org/71C94F28-C439-4F37-86F8-DDEE53B9BD67

Figure 50

##### Etymology.

The specific name is derived from the Latin *unctus*, meaning polished, referring to the posterior part of the propodeum.

##### Type series.

The holotype female only.

HT: ♀, Matsuyama, Ehime, Shikoku, JAPAN, 26.X.1954, S. Ueda leg. (EUM).

##### Distribution.

Eastern Palaearctic region.

JAPAN: [Shikoku] Ehime.

##### Bionomics.

Unknown.

##### Differential diagnosis.

Some characters (e.g., wide face, long and slender mandible, shape of sclerites) suggest that *E. unctus* sp. nov. is related to *E. shikokuensis*. However, *E. unctus* sp. nov. can easily be distinguished from all other Japanese *Enicospilus* by the uniformly punctate and shiny posterior area of the propodeum.

##### Description.

Female (HT). Body length ca. 21.0 mm.

**Figure 50.**
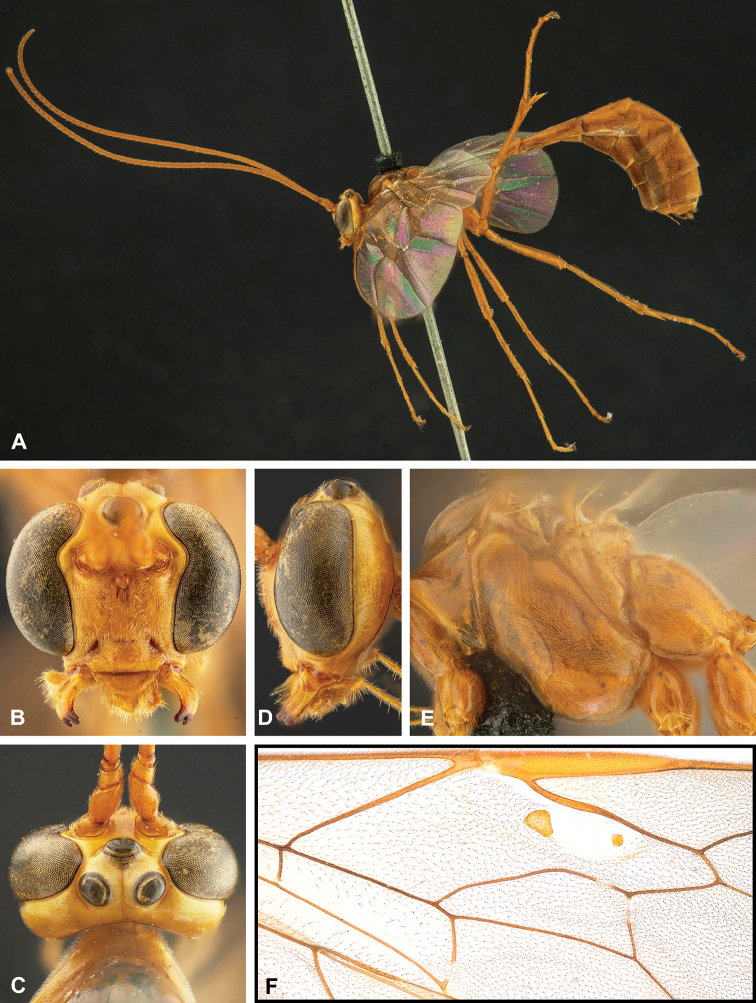
*Enicospilus unctus* Shimizu, sp. nov. ♀ (HT) from Japan **A** habitus **B** head, frontal view **C** head, dorsal view **D** head, lateral view **E** mesosoma, lateral view **F** central part of fore wing.

Head with GOI = 2.0 (Fig. 50D). Lower face 0.9× as wide as high, strongly shiny, moderately punctate with setae (Fig. 50B, D). Clypeus 2.1× as wide as high, moderately punctate with setae, convex in profile, and ventral margin impressed (Fig. 50B, D). Malar space 0.4× as long as basal mandibular width (Fig. 50B, D). Mandible moderately twisted by 20°, very long, proximally narrowed, distally parallel sided, outer surface with diagonal setose groove (Fig. 50B, D). Upper tooth of mandible 2.1× as long as lower. Frons, vertex and gena strongly shiny with fine setae (Fig. 50B–D). Posterior ocellus distinctly separated from eye (Fig. 50B–D). Ventral end of occipital carina joining oral carina. Antennae with 54 flagellomeres; first flagellomere 1.5× as long as second; 20^th^ flagellomere 1.9× as long as wide.

Mesosoma entirely strongly shiny with fine setae (Fig. 50E). Pronotum diagonally finely punctostrigose (Fig. 50E). Mesoscutum 1.3× as long as maximum width, finely punctate to smooth and evenly rounded in profile (Fig. 50E). Notauli absent (Fig. 50E). Scutellum moderately convex, finely punctate to smooth, with lateral longitudinal carinae along entire length of scutellum. Epicnemium moderately punctate with setae. Epicnemial carina weak, evenly curved to anterior, dorsal end not reaching anterior margin of mesopleuron (Fig. 50E). Meso- and metapleuron evenly moderately punctate (Fig. 50E). Submetapleural carina broadened anteriorly (Fig. 50E). Propodeum evenly rounded in profile; anterior transverse carina complete centrally and not joining pleural carina laterally; anterior area longitudinally striate; spiracular area smooth; posterior area entirely moderately punctate; propodeal spiracle elliptical and not joining pleural carina by ridge (Fig. 50E).

Wings (Fig. 50F). Fore wing length ca. 14.5 mm with AI = 0.6, CI = 0.3, DI = 0.4, ICI = 0.4, SDI = 1.2, SI = 0.1, SRI = 0.4; vein 1m-cu&M slightly sinuous; vein 2r&RS slightly sinuous; vein RS evenly curved; fenestra and sclerites of discosubmarginal cell as in Fig. 50F; proximal sclerite rounded triangular, not confluent with distal sclerite, strongly pigmented; central sclerite suboval, pigmented, positioned in anterodistal part of fenestra; distal sclerite vestigial; proximal corner of marginal cell evenly setose; posterodistal corner of second discal cell ca. 80° and of subbasal cell ca. 80°; vein 1cu-a antefurcal to M&RS by less than 0.1× length of 1cu-a. Hind wing with NI = 1.0; vein RS almost straight; vein RA with 6 rather stout uniform hamuli.

Legs. Hind leg with coxa in profile 1.9× as long as deep; basitarsus 2.1× as long as second tarsomere; fourth tarsomere 3.7× as long as wide; tarsal claw simply pectinate.

Metasoma with DMI = 1.2, PI = 3.1, THI = 1.7; thyridium oval; ovipositor sheath not longer than posterior depth of metasoma (Fig. 50A).

Colour (Fig. 50). Entirely red-brown except for head yellow-brown and apex of mandible black. Wings hyaline. Fore wing sclerites pigmented and amber. Wing veins red-brown to amber.

Male. Unknown.

#### Enicospilus vestigator

Taxon classificationAnimaliaHymenopteraIchneumonidae

(Smith, 1858)

982A80BB-F3F8-52A1-82BB-DB6E3A99F0B9

Figure 51

Ophion vestigator Smith, 1858: 122; HT ♂ from Malaysia, OUMNH, not examined.
Eniscospilus (sic) unilineatus Cameron, 1905a: 123; HT ♂ from Sri Lanka, NHMUK, examined; synonymised by Gauld and Mitchell (1981: 471).
Henicospilus xantusi Szépligeti, 1906: 138; HT ♀ from Borneo, TM, not examined; synonymised by Gauld and Mitchell (1981: 471).
Enicospilus receptor Chiu, 1954: 40; HT ♀ from Taiwan, TARI, examined; synonymised by Townes et al. (1961: 293).
Enicospilus glabrifascies Chiu, 1954: 40; HT ♂ from Taiwan, TARI, examined; synonymised by Gauld and Mitchell (1981: 471).
Enicospilus (Polycorniata) carinatus Rao and Nikam, 1971: 103; HT ♀ from India, MUC, not examined; synonymised by Gauld and Mitchell (1981: 472).

##### Specimens examined.

Total of 23 specimens (19♀♀4♂♂): Brunei (2♀♀), India (11♀♀1♂), Japan (5♀♀1♂), Sri Lanka (1♂), Taiwan (1♀1♂).

Type series: HT ♂ of *Eniscospilus* (sic) *unilineatus* Cameron, 1905, Peradeniya, Ceylon, SRI LANKA, P. Cameron leg. (NHMUK, Type 3b.1237); HT ♀ of *Enicospilus receptor* Chiu, 1954, Koshun, TAIWAN, 27.XI.1923, R. Takahashi leg. (TARI); HT ♂ of *Enicospilus glabrifascies* Chiu, 1954, Kuraru, TAIWAN, 1.VIII.1931, T. Shiraki leg. (TARI).

##### Distribution.

Australasian, Oceanic, and Oriental regions (Yu et al. 2016); new to the Eastern Palaearctic region.

Newly recorded from Japan.

JAPAN: [Kyûshû] Nagasaki; [Ryûkyûs] Okinawa.

##### Bionomics.

Unknown.

##### Differential diagnosis.

**Figure 51.**
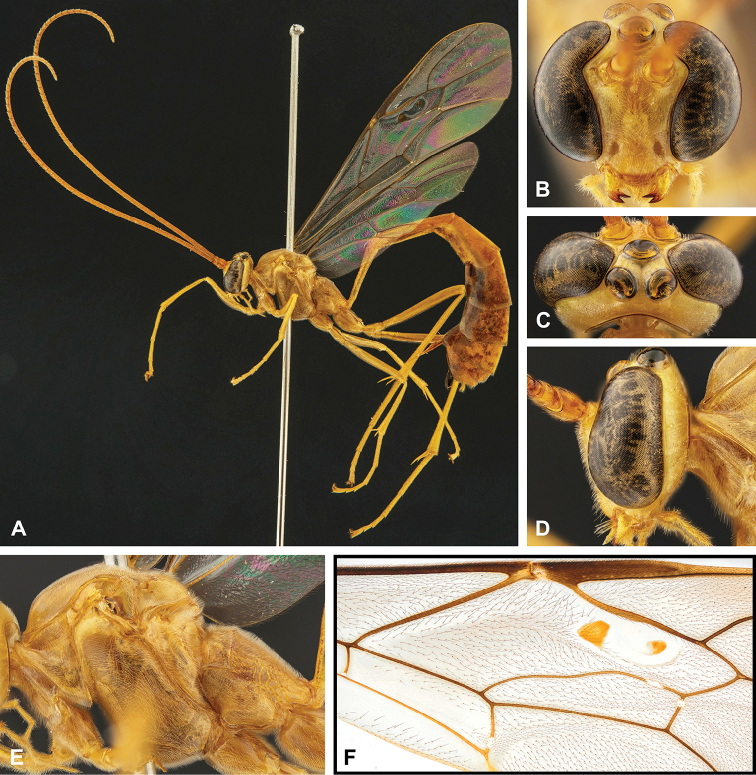
*Enicospilus vestigator* (Smith, 1858) ♀ from Japan **A** habitus **B** head, frontal view **C** head, dorsal view **D** head, lateral view **E** mesosoma, lateral view **F** central part of fore wing.

This species resembles *E. laqueatus*, *E. pseudantennatus* and *E. tripartitus*, which all have similar sclerites, but can be distinguished from *E. laqueatus* by the smooth outer mandibular surface (Fig. 51B) (outer mandibular surface with a diagonal setose groove in *E. laqueatus*, as in Fig. 24B); from *E. pseudantennatus* by the strongly twisted mandible (i.e., twisted by 60–80°, as in Fig. 51B, D, but 10–20° in *E. pseudantennatus*) and elongate discosubmarginal cell (Fig. 51F) (rather short in *E. pseudantennatus*); and from *E. tripartitus* by the strongly twisted and smooth mandible (Fig. 51B, D) (mandible rather weakly twisted and outer surface with dense and stout setae and punctures in *E. tripartitus*, as in Fig. 49B, D) and elongate discosubmarginal cell (Fig. 51F) (rather short in *E. tripartitus*, as in Fig. 49F), as summarised in Table 6.

##### Remarks.

Although Japanese specimens do not vary significantly, some wing characters of the holotype of *E. unilineatus* are different from other specimens, as Gauld and Mitchell (1981: 472) mentioned.

#### Enicospilus xanthocephalus

Taxon classificationAnimaliaHymenopteraIchneumonidae

Cameron, 1905

EB941790-C1A4-5501-A093-0CD6EABACDA7

Figure 52

Eniscospilus (sic) xanthocephalus Cameron, 1905a: 122; HT ♀ from Sri Lanka, NHMUK, examined.
Enicospilus bullatus Chiu, 1954: 53; HT ♀ from Taiwan, TARI, examined; synonymised by Gauld and Mitchell (1981: 210).
Enicospilus obliquus Chiu, 1954: 54; HT ♂ from Taiwan, TARI, examined; junior secondary homonym of Enicospilus obliquus (Morley, 1912); synonymised by Gauld and Mitchell (1981: 210).
Enicospilus clinatus Townes, Townes and Gupta, 1961: 272; replacement name for Enicospilus obliquus Chiu, 1954.
Enlcospilus (sic) (Bicorn’ata) (sic) *paraclinatus* Nikam, 1975: 198; HT ♂ from India, MUC, not examined; synonymised by Nikam (1980).
Enicospilus pexus Gauld, 1977: 57, 86; HT ♀ from Australia, ANIC, not examined; synonymised by Gauld and Mitchell (1981: 211).

##### Specimens examined.

Total of 123 specimens (103♀♀19♂♂ and 1 unsexed): Brunei (3♀♀), India (83♀♀5♂♂), Japan (7♀♀9♂♂), Philippines (1♀), Sri Lanka (3♀♀), Taiwan (6♀♀5♂♂ and 1 unsexed).

Type series: HT ♀ of *Eniscospilus* (sic) *xanthocephalus* Cameron, 1905, Peradeniya, Ceylon, SRI LANKA, VI.1902, P. Cameron leg. (NHMUK, Type 3b.1236); HT ♀ of *Enicospilus bullatus* Chiu, 1954, Kanshirei, TAIWAN, 19.XI.1928, J. Sonan leg. (TARI); HT ♂ of *Enicospilus obliquus* Chiu, 1954, Kuraru, TAIWAN, 12–15.III.1931, T. Shiraki leg. (TARI).

##### Distribution.

Australasian and Oriental regions (Yu et al. 2016); this is a predominantly Oriental species.

JAPAN: [Ryûkyûs] Kagoshima (Shimizu and Maeto 2016; present study) and Okinawa (Shimizu and Maeto 2016; present study).

##### Bionomics.

No Japanese rearings. A range of hosts have been recorded in the literature, with some looking more reliable than others.

##### Differential diagnosis.

**Figure 52.**
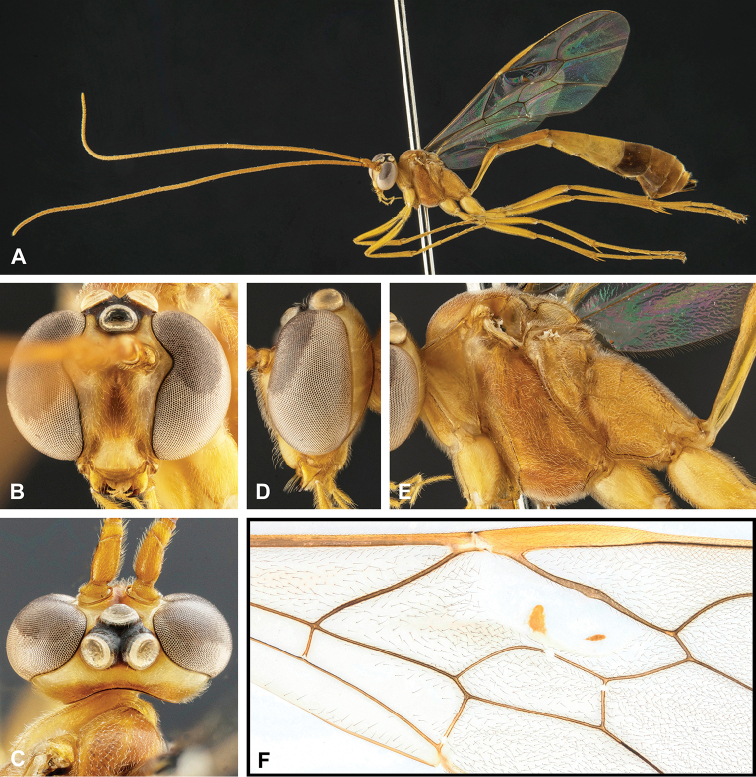
*Enicospilus xanthocephalus* Cameron, 1905 ♀ from Japan **A** habitus **B** head, frontal view **C** head, dorsal view **D** head, lateral view **E** mesosoma, lateral view **F** central part of fore wing.

This species is sometimes confused with *E. flavocephalus* because their body size, general colour, body shape, etc., are very similar, as in Figs 17 and 52. However, *E. xanthocephalus* is easily distinguished by the black interocellar area (Fig. 52B, C), shape of fore wing veins and sclerites (Fig. 52F), etc. (cf. Differential diagnosis of *E. flavocephalus* for details). The significantly large value of AI (more than 2.0) is also characteristic of *E. xanthocephalus* and helps identification.

#### Enicospilus yezoensis

Taxon classificationAnimaliaHymenopteraIchneumonidae

(Uchida, 1928)

B14DC437-0455-5F33-9AB8-5FD54E0E05E5

Figure 53

Henicospilus yezoensis Uchida, 1928: 227; LCT ♀ from Japan, SEHU, examined.
Enicospilus ranunculus Chiu, 1954: 36; HT ♀ from South Korea, TARI, examined; **syn. nov.**

##### Specimens examined.

Total of 31 specimens (18♀♀12♂♂ and 1 unsexed): Japan (16♀♀12♂♂ and 1 unsexed), South Korea (1♀), unknown (1♀).

Type series: LCT ♀ of *Henicospilus yezoensis* Uchida, 1928, Maruyama, Hokkaidô, JAPAN, 27.VII.1929, T. Uchida leg. (SEHU); HT ♀ of *Enicospilus ranunculus* Chiu, 1954, SOUTH KOREA (TARI).

##### Distribution.

Eastern Palaearctic region (Yu et al. 2016).

Newly recorded from South Korea.

JAPAN: [Hokkaidô] (Uchida 1928; present study); [Tôhoku] Aomori (Ichita 1994; present study), Yamagata*, and Fukushima*; [Kantô-Kôshin] Tochigi*, Nagano*, Tôkyô*, and Kanagawa*; [Tôkai] Mie*; [Kinki] Hyôgo* and Nara*; [Shikoku] Kôchi*; [Kyûshû] Fukuoka*, Saga* and Kagoshima (Fukuda and Kusigemati 1986; present study). *New records.

##### Bionomics.

Unknown.

##### Differential diagnosis.

**Figure 53.**
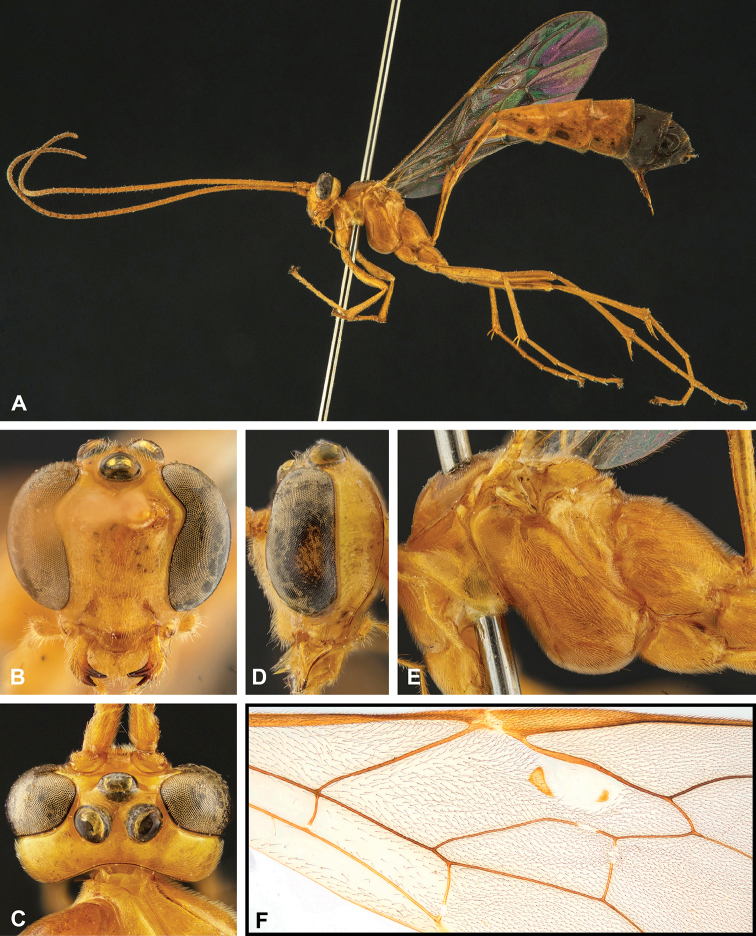
*Enicospilus yezoensis* (Uchida, 1928) ♀ from Japan **A** habitus **B** head, frontal view **C** head, dorsal view **D** head, lateral view **E** mesosoma, lateral view **F** central part of fore wing.

This species is similar to *E. melanocarpus* and *E. ramidulus*. However, *E. yezoensis* is easily distinguished from all other species of *Enicospilus* by the following combination of character states: proximal and distal sclerites separated (Fig. 53F); central sclerite comma-shaped (Fig. 53F); face wide and subquadrate (Fig. 53B); gena wide and not constricted behind eye in dorsal view (Fig. 53C, D); and diagonal groove of outer mandibular surface with dense and long setae (Fig. 53B, D) (also see Table 8).

##### Remarks.

This species is rather morphologically stable. The holotype of *E. ranunculus* syn. nov. is clearly conspecific with *E. yezoensis* and is newly synonymised here.

#### Enicospilus yonezawanus

Taxon classificationAnimaliaHymenopteraIchneumonidae

(Uchida, 1928)

F9FBFAD0-FF4C-5C66-95F2-B6DCAC40E5E6

Figure 54

Henicospilus yonezawanus Uchida, 1928: 218; LCT ♀ from Japan, designated by Townes et al. (1965: 337), SEHU, examined.
Enicospilus microstriatellus Uchida, 1956: 95; HT ♂ from Ryûkyûs, SEHU, examined; synonymised by Gauld and Mitchell (1981: 337).

##### Specimens examined.

Total of 303 specimens (196♀♀103♂♂ and 4 unsexed): India (12♀♀ and 2 unsexed), Indonesia (1♀), Japan (166♀♀101♂♂), Laos (11♀♀1♂), Malaysia (5♀♀ and 2 unsexed), Papua New Guinea (1♀), Taiwan (1♂).

Type series: LCT ♀ of *Henicospilus yonezawanus* Uchida, 1928, Yonezawa, Yamagata Pref., Tôhoku, JAPAN, 23.VII.1919, S. Matsumura leg. (SEHU); HT ♂ of *Enicospilus microstriatellus* Uchida, 1956, Sinmura, Amami-ôshima, Kagoshima Pref., Ryûkyûs, JAPAN, 7.IV.1954, T. Kumata leg. (SEHU).

##### Distribution.

Australasian, Eastern Palaearctic, and Oriental regions (Yu et al. 2016).

JAPAN: [Tôhoku] Akita*, Yamagata (Uchida 1928; Shimizu 2020; present study), and Fukushima*; [Hokuriku] Niigata*; [Kantô-Kôshin] Tochigi (Uchida 1928) and Tôkyô (Konishi and Maeto 2000; present study); [Tôkai] Shizuoka*; [Kinki] Shiga*, Kyôto*, Hyôgo*, and Wakayama*; [Chûgoku] Shimane* and Hiroshima (Konishi and Nakamura 2002, 2005, 2010; Maeto and Shimizu 2019; present study); [Kyûshû] Fukuoka*, Nagasaki*, and Kagoshima (Shimizu 2020); [Ryûkyûs] Kagoshima (Uchida 1956; Momoi 1970; Shimizu 2020; present study) and Okinawa (Momoi 1970; Shimizu 2020; present study). *New records.

##### Bionomics.

Unknown.

##### Differential diagnosis.

**Figure 54.**
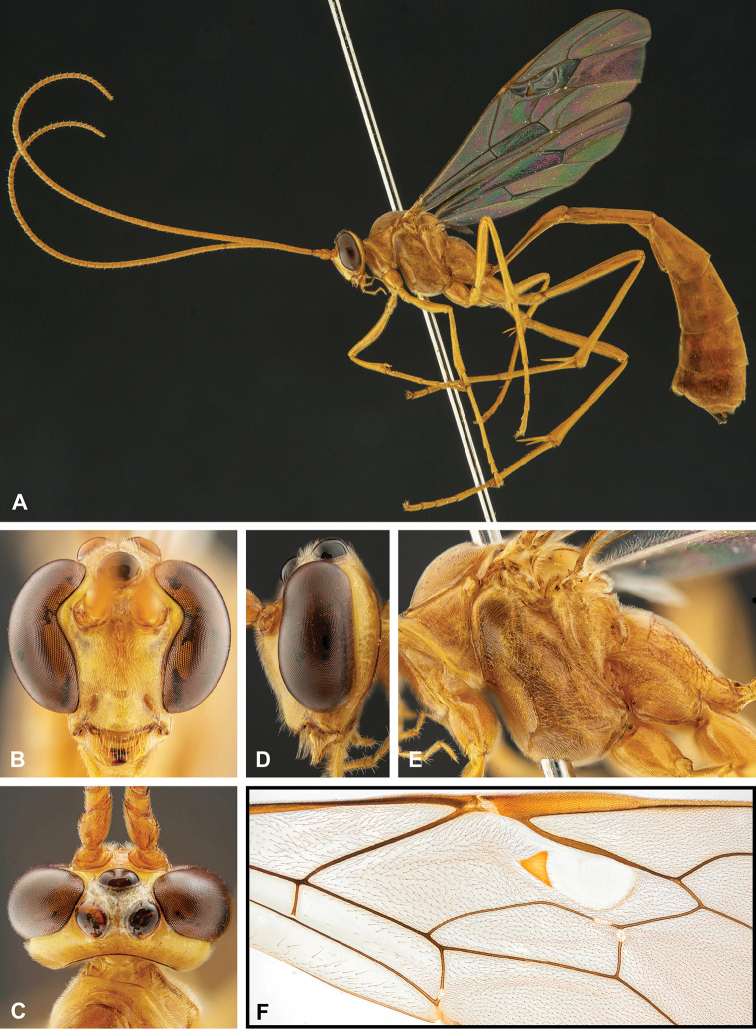
*Enicospilus yonezawanus* (Uchida, 1928) ♀ from Japan **A** habitus **B** head, frontal view **C** head, dorsal view **D** head, lateral view **E** mesosoma, lateral view **F** central part of fore wing.

*Enicospilus yonezawanus* is one of the most common *Enicospilus* species in Japan and easily distinguished from all other *Enicospilus* species by the following combination of character states: ventral margin of clypeus impressed (Fig. 54B, D); fore wing fenestra with triangular proximal sclerite and without central sclerite (Fig. 54F); and meso- and metapleuron closely punctostriate (Fig. 54E). *Enicospilus yonezawanus* is also sometimes confused with *E. jilinensis* but can easily be separated (cf. Differential diagnosis of *E. jilinensis*).

##### Remarks.

There is some variation in the shape of the proximal sclerite, but in Japanese specimens it is usually very stable.

#### Enicospilus zeugos

Taxon classificationAnimaliaHymenopteraIchneumonidae

Chiu, 1954 stat. rev.

3A2C025C-C7FD-54C5-A6A5-470E4E09EA52

Figure 55

Enicospilus zeugos Chiu, 1954: 64; HT ♀ from Taiwan, TARI, examined; **stat. rev.**
Enicospilus henrytownesi Chao and Tang, 1991: 51; HT ♀ from Taiwan, EMUS, examined; **syn. nov.**

##### Specimens examined.

Total of 5 specimens (3♀♀2♂♂): Japan (1♂), Taiwan (3♀♀1♂).

Type series: HT ♀ of *Enicospilus zeugos* Chiu, 1954, Urai, TAIWAN, VI.1931, J. Sonan leg. (TARI); HT ♀ of *Enicospilus henrytownesi* Chao & Tang, 1991, Wushe (1,150 m), TAIWAN, 26.IV.1983, H. Townes leg. (EMUS).

##### Distribution.

Oriental region (Yu et al. 2016).

Newly recorded from Japan.

JAPAN: [Ryûkyûs] Okinawa.

##### Bionomics.

Unknown.

##### Differential diagnosis.

**Figure 55.**
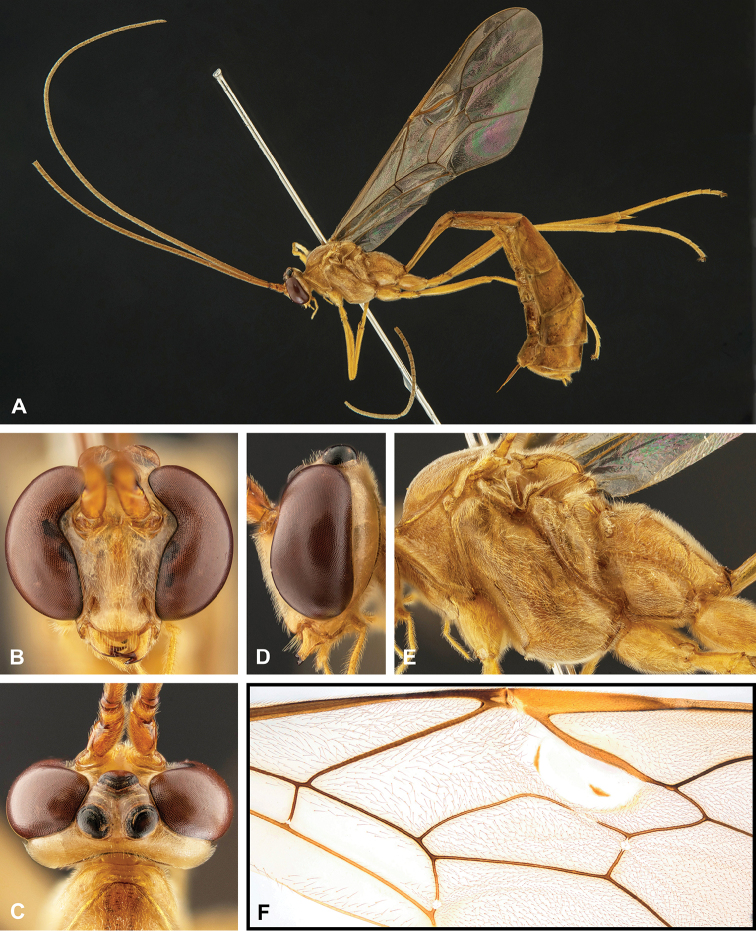
*Enicospilus zeugos* Chiu, 1954, stat. rev. ♀ from Japan **A** habitus **B** head, frontal view **C** head, dorsal view **D** head, lateral view **E** mesosoma, lateral view **F** central part of fore wing.

This species can very easily be distinguished from all other *Enicospilus* by the unique shape of the fore wing sclerites (Fig. 55F).

##### Remarks.

Both *E. zeugos* stat. rev. and *E. henrytownesi* syn. nov. had been synonymised under *E. grammospilus* (Enderlein, 1921) by Gauld and Mitchell (1981: 316) and Shimizu (2018: 93) respectively. However, the shape of fore wing veins and sclerites and the results of DNA barcoding analysis indicate that *E. zeugos* stat. rev. (with *E. henrytownesi* syn. nov. as a junior synonym) is easily separated from *E. grammospilus*, hence we propose a revised status for *E. zeugos* stat. rev. here.

### *Enicospilus* species erroneously recorded from Japan

The following species have been recorded from Japan in error so were not included in the present study.

***Enicospilus biharensis* Townes, Townes & Gupta, 1961.**Gupta (1987) recorded this species from Japan, but this record was probably based on a misidentification. We examined the HT of Henicospilus horsfieldi var glabratus Morley, 1913 [♀, Chapra, INDIA (NHMUK, Type 3b.1266)] (= HT of *E. biharensis*) and many other specimens of this species. *Enicospilus biharensis* is characterised by the small value of CI, densely roughly punctate mesopleuron, and evenly curved fore wing vein 1m-cu&M (cf. Table 7). However, we have not seen any Japanese specimens and Gupta (1987) probably recorded this species based on *E. maruyamanus*.

***Enicospilus flavicaput* (Morley, 1912).** This species had been recorded from Japan by Matsumura and Uchida (1926), and some additional Japanese specimens were also identified as *E. flavicaput*. However, all Japanese specimens identified as this species were based on misidentifications of other *Enicospilus* species, including a new species (*E. matsumurai* sp. nov.).

***Enicospilus merdarius* (Gravenhorst, 1829).** This species had been recorded from Japan by Matsumura and Uchida (1926) and Uchida (1928). However, all Japanese specimens identified as this species have proved to belong to other *Enicospilus* species.

*Enicospilus merdarius* has been recorded from all over the world except the Afrotropical, Antarctic and Australasian regions (Yu et al. 2016). As Gauld and Mitchell (1981) mentioned, however, any individuals of *Enicospilus* with two fore wing sclerites and an entirely orange-brown metasoma have been identified as this species, and the name ‘*E. merdarius*’ had frequently been used as a blanket term. Broad and Shaw (2016) demonstrated that the name *E. merdarius* in fact applies to a very different species of *Enicospilus* (formerly known as *E. tournieri* (Vollenhoven)) and that several species, including *E. adustus* (Haller), *E. cerebrator* Aubert and *E. myricae* Broad & Shaw, had been mixed up under the name ‘*merdarius*’ in Europe.

### Species richness analysis

**Figure 56.**
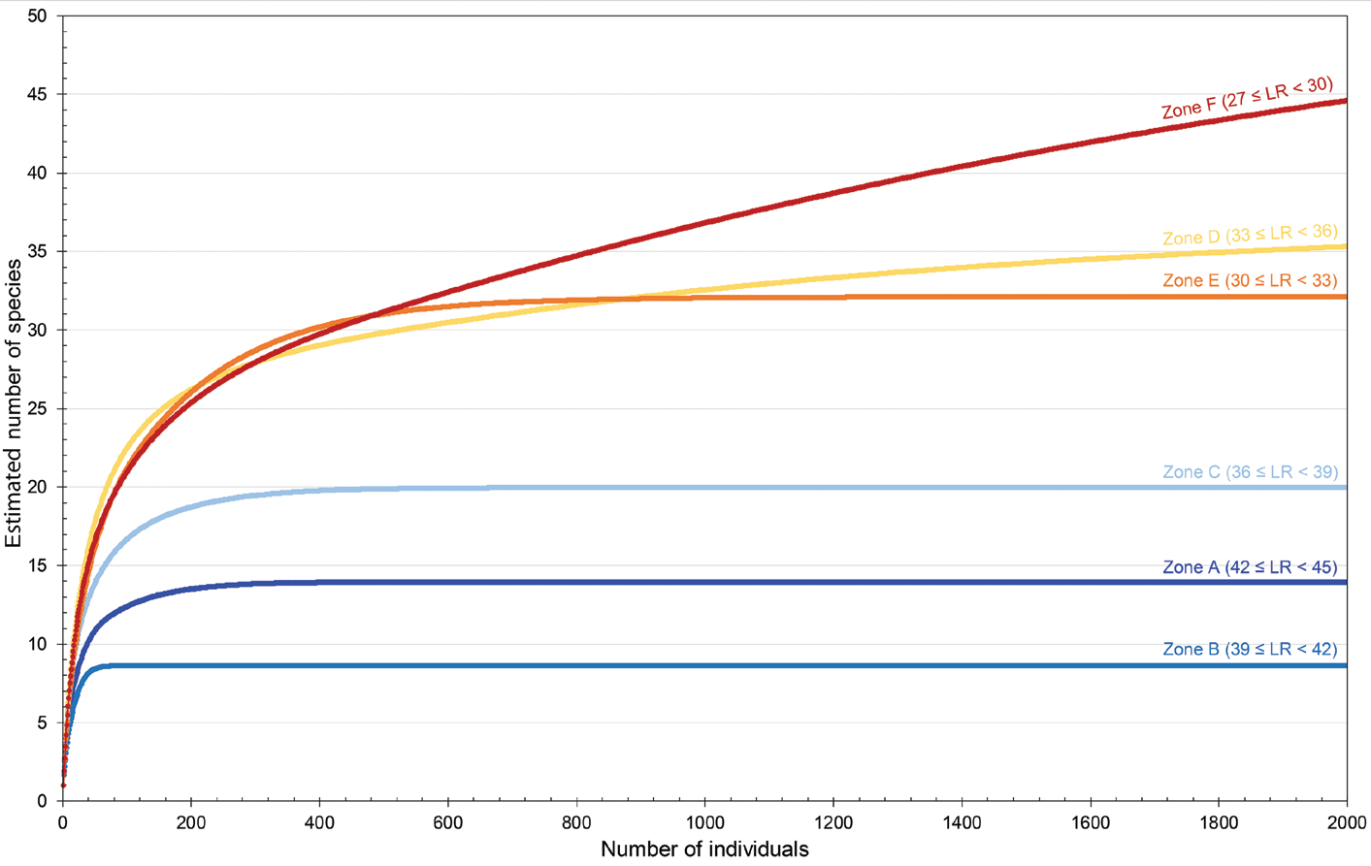
Individual-based extrapolated species accumulation curve, comparing each zone rarefied to 2,000 individuals (LR = latitudinal ranges).

**Figure 57.**
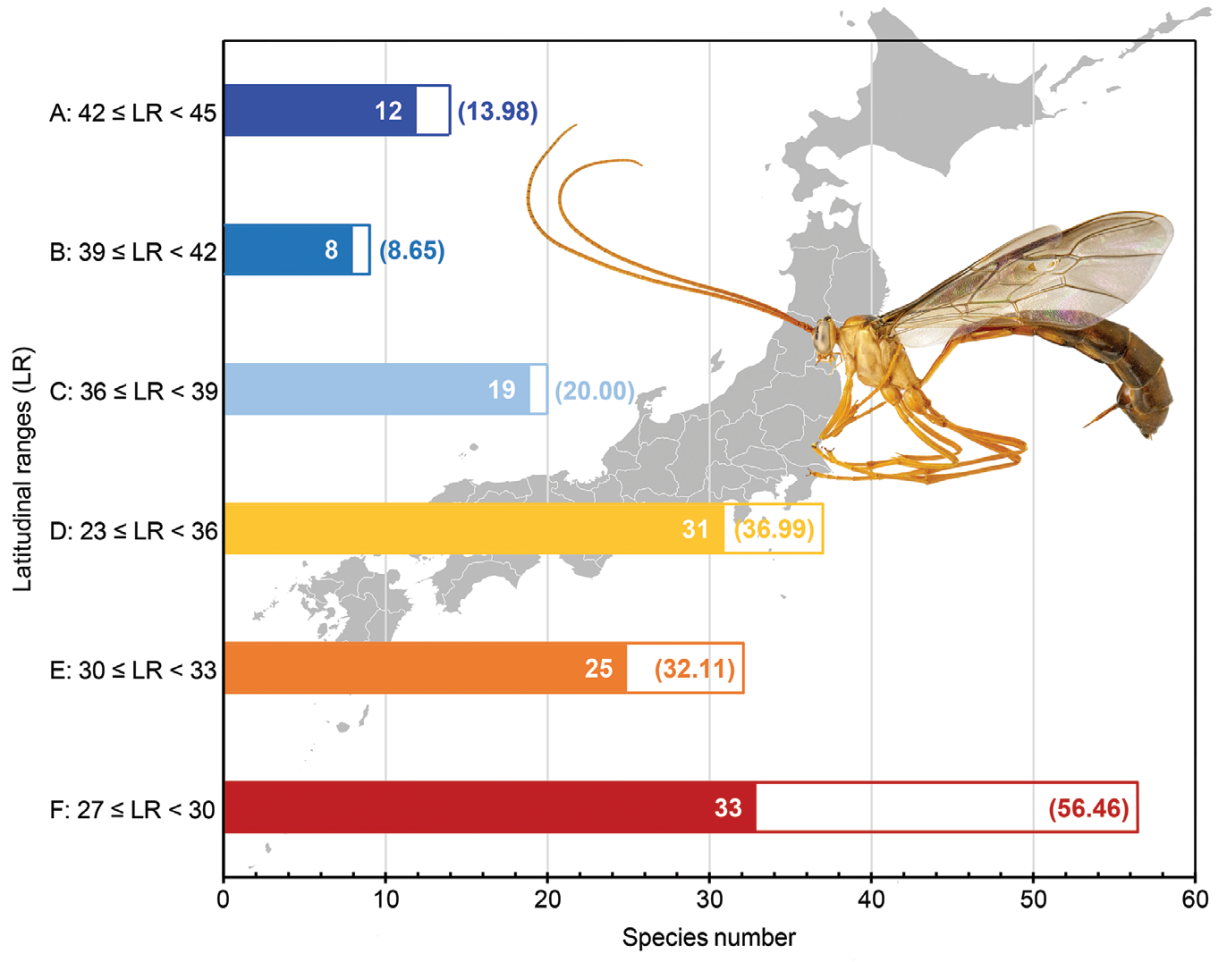
Latitudinal pattern of *Enicospilus* species richness across Japan. Coloured bars indicate observed species number and extended non-coloured bars indicate saturation species richness, estimated by individual-based extrapolation methods based on Chao1 richness estimator in EstimateS v.9.1.0 software application. *Enicospilus* species richness across Japan significantly decreases towards the north (Spearman’s rank correlation coefficient = -0.89, *p*-value = 0.03).

A total of 12, 8, 19, 31, 25, and 33 species were observed from the latitudinal zones A to F respectively, and saturation species richness was estimated at 13.98, 8.65, 20.00, 36.99, 32.11 and 56.46 in each zone (Figs 56, 57; values rounded to the nearest whole number for biological meaningfulness). *Enicospilus* species richness in Japan significantly decreases towards the north (Spearman’s rank correlation coefficient = -0.89, *p*-value = 0.03; Fig. 57).

**Figure 58.**
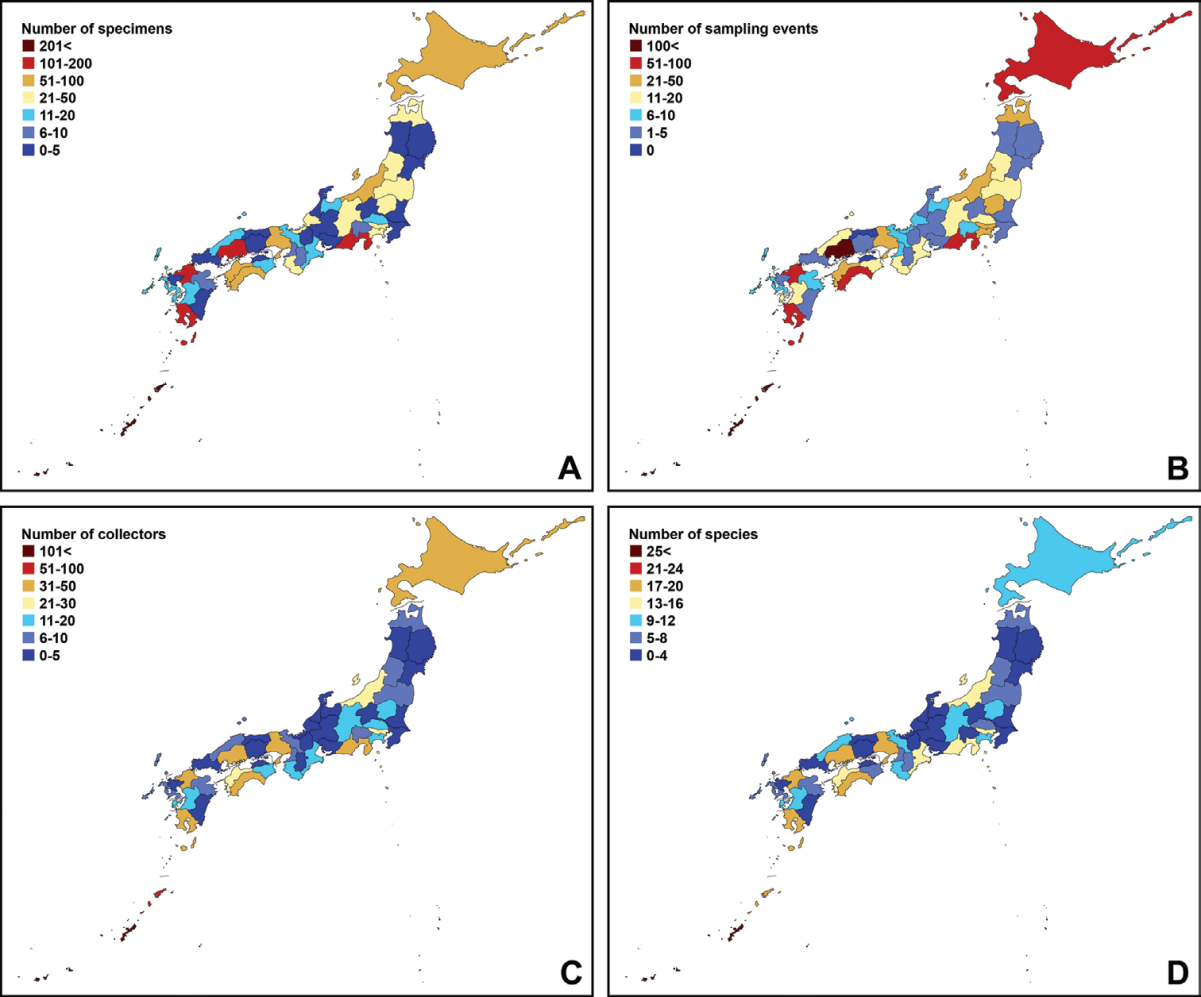
Heat maps of regional patterns **A** number of specimens **B** number of sampling events **C** number of collectors **D** number of species.

Regional patterns of the four categories (i.e., number of specimens, collection events, collectors, and species) are visualised as heat maps in Fig. 58. All of these indicate apparent regional biases. The number of specimens, collection events, and collectors were remarkably high in Ryûkyûs (285, 112, and 69 in Kagoshima, and 242, 181, and 125 in Okinawa respectively, as in Table 9 and Fig. 58), and there were many collection events in Shizuoka (= 94) and Hiroshima (= 101) too. In contrast, three of six prefectures in Tôhoku (Akita, Iwate, and Miyagi), three of four in Hokuriku (Toyama, Ishikawa, and Fukui), four of nine in Kantô-Kôshin (Ibaraki, Gunama, Yamanashi, and Chiba), two of four in Tôkai (Gifu and Aichi), four of six in Kinki (Kyôto, Shiga, Ōsaka, and Nara), three of five in Chûgoku (Tottori, Okayama, and Yamaguchi), one of four in Shikoku (Kagawa), three of seven in Kyûshû (Saga, Ōita, and Miyazaki), and Ogasawara were sparsely represented in all categories (Table 9; Fig. 58). In particular, the number of specimens, collection events, and collectors as well as of species were significantly scarce in the following regions: zero in one prefecture of Chûgoku (Tottori) and one of Shikoku (Kagawa); and one or two in three of Tôhoku (Akita, Iwate, and Miyagi), one of Kantô-Kôshin (Chiba), one of Tôkai (Gifu), one of Kinki (Shiga), and one of Chûgoku (Okayama) (Table 9; Fig. 58).

**Figure 59.**
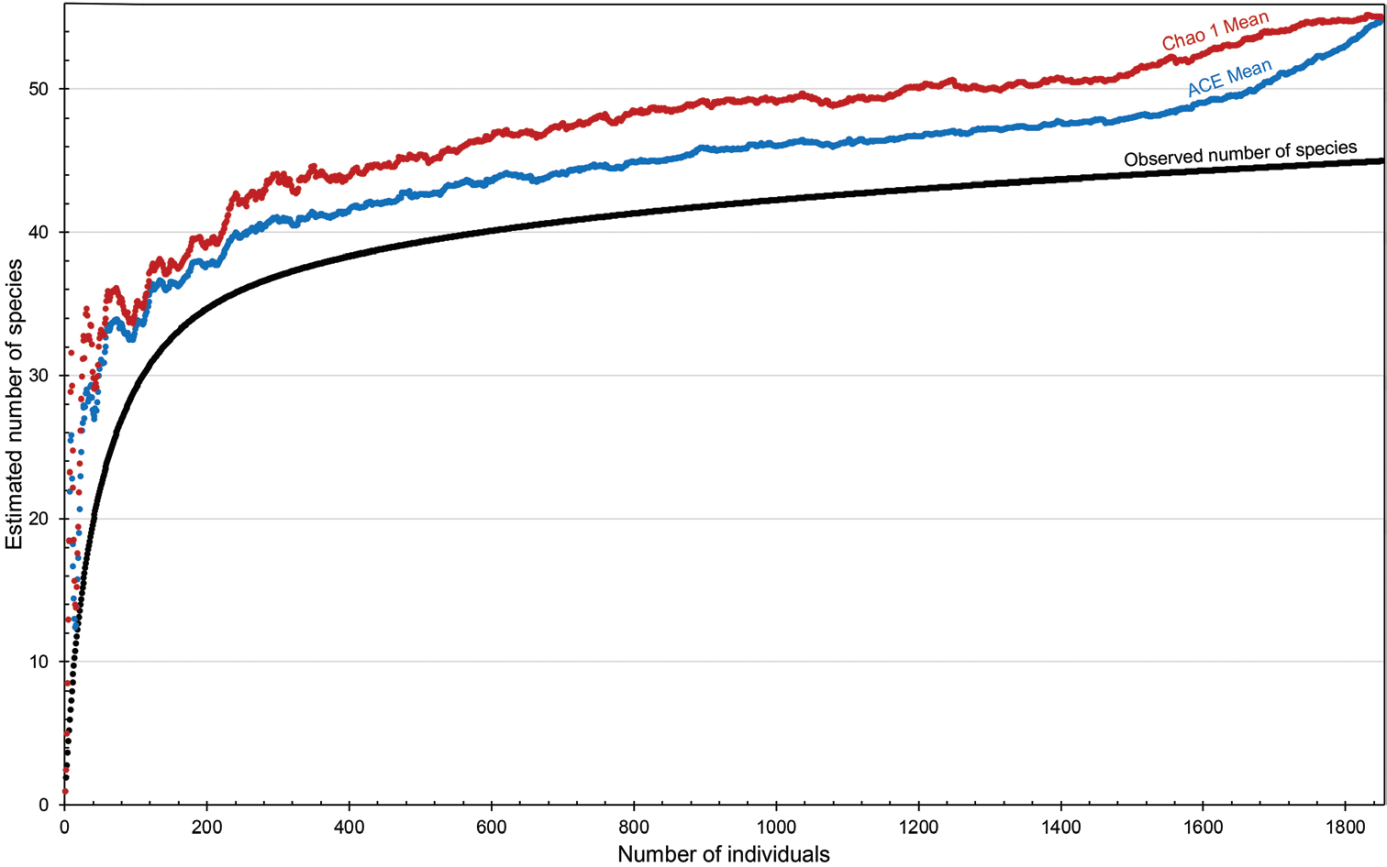
Individual-based species accumulation curve, comparing the observed and estimated numbers of *Enicospilus* species in Japan, based on ACE and Chao 1 estimators.

Individual-based observed and rarefaction numbers of *Enicospilus* species in Japan are shown in Table 10 and as a species accumulation curve in Figure 59, based on ACE and Chao 1 estimators. Estimated species number of *Enicospilus* in Japan differed between the two estimators and was lower with ACE than Chao 1 estimator: 54.79 and 55.02 species at 1,850 individuals in ACE and Chao 1 estimators respectively.

**Table 9.** Regional patterns of the number of specimens, collection events, collectors, and species. Bold indicates especially small numbers (fewer than 5).

Provinces	Prefectures	Specimens	Collection events	Collectors	Species
Hokkaidô	Hokkaidô	81	53	33	12
Tôhoku	Aomori	34	30	8	8
	**Akita**	**1**	**1**	**1**	**1**
	**Iwate**	**1**	**1**	**1**	**1**
	Yamagata	24	17	7	6
	**Miyagi**	**2**	**1**	**1**	**1**
	Fukushima	27	15	10	6
Hokuriku	Niigata	52	36	27	13
	Toyama	11	7	**3**	**2**
	**Ishikawa**	**5**	**5**	**4**	**4**
	Fukui	38	9	**4**	**4**
Kantô-Kôshin	**Ibaraki**	**5**	**4**	**3**	**3**
	Tochigi	37	22	17	12
	**Gunma**	**3**	**3**	**3**	**3**
	Nagano	21	15	13	9
	Yamanashi	6	6	6	**4**
	Saitama	19	19	13	7
	Tôkyô	48	36	28	15
	Kanagawa	34	31	17	12
	**Chiba**	**1**	**1**	**1**	**1**
Tôkai	**Gifu**	**2**	**2**	**1**	**4**
	**Aichi**	**4**	**4**	**3**	**2**
	Shizuoka	159	94	33	15
	Mie	16	16	14	13
Kinki	Kyôto	11	7	7	9
	**Shiga**	**1**	**1**	**1**	**1**
	Ōsaka	8	6	6	6
	Hyôgo	52	42	37	17
	Nara	9	**5**	**5**	6
	Wakayama	26	16	12	9
Chûgoku	**Tottori**	**0**	**0**	**0**	**1**
	Shimane	12	12	9	9
	**Okayama**	**2**	**2**	**2**	**2**
	Hiroshima	108	101	34	17
	**Yamaguchi**	**4**	**4**	**4**	**3**
Shikoku	**Kagawa**	**0**	**0**	**0**	**1**
	Tokushima	19	13	11	8
	Ehime	76	43	23	16
	Kôchi	62	52	32	20
Kyûshû	Fukuoka	105	69	44	18
	**Saga**	**5**	**4**	**4**	**4**
	Nagasaki	12	10	8	8
	Ōita	10	7	6	6
	Kumamoto	20	15	15	11
	**Miyazaki**	**5**	**5**	**4**	**3**
	Kagoshima	112	62	37	18
Ryûkyûs	Kagoshima	285	112	69	20
	Okinawa	242	181	125	31
Ogasawara	Tôkyô	38	**4**	**3**	**3**

**Table 10.** Rarefaction of *Enicospilus* species in Japan estimated using ACE and Chao 1 estimators.

Individuals	Observed number of species	Estimated number of species	
		ACE Mean	Chao 1 Mean
50	22.12	30.84	32.94
100	29.14	33.50	34.77
150	32.64	36.58	38.07
200	34.68	37.75	39.18
250	36.02	39.78	42.21
300	36.99	40.83	43.55
350	37.74	41.34	44.66
400	38.35	41.60	43.88
450	38.88	42.15	44.75
500	39.34	42.69	45.43
550	39.75	43.40	45.70
600	40.12	43.61	46.69
650	40.46	43.83	46.93
700	40.78	44.21	47.63
750	41.07	44.65	48.16
800	41.34	44.93	48.40
850	41.60	45.11	48.52
900	41.84	45.88	49.08
950	42.07	45.83	49.10
1000	42.29	46.02	49.07
1050	42.49	46.29	49.33
1100	42.69	46.31	49.12
1150	42.88	46.42	49.32
1200	43.06	46.72	50.08
1250	43.23	47.11	50.63
1300	43.40	47.25	50.05
1350	43.56	47.38	50.37
1400	43.72	47.70	50.79
1450	43.87	47.89	50.67
1500	44.02	48.04	51.12
1550	44.16	48.38	52.16
1600	44.30	49.07	52.50
1650	44.44	49.61	53.15
1700	44.58	50.77	53.98
1750	44.72	51.97	54.66
1800	44.85	53.02	54.83
1850	44.99	54.79	55.02

## Discussion

We revised the Japanese species of *Enicospilus* using a combined morphological and DNA barcoding approach to delimit and describe species. Some studies have suggested that genetic introgression has rather frequently occurred in the Ichneumonidae by *Wolbachia* endosymbionts, leading to misleading DNA barcode signals (e.g., Klopfstein et al. 2016). The inconsistency of each approach for a few species (*E. xanthocephalus*, *E. stenophleps*, *E. puncticulatus*, and *E. flavicaput* (non-Japanese specimens)) suggests that these species are potentially affected by genetic introgression or include cryptic species. Although *E. puncticulatus* exhibits a very wide range of variation in morphology and probably does contain cryptic species, an especially small divergence of p-distances among the specimens (less than 1.0%) also suggests that the CO1 evolutionary rate is too slow to delimit species and cannot catch up with very rapid speciation; hence, using another molecular approach, such as the internal transcribed spacer 2 (ITS2) and SSR markers, a future study could help improve species delimitation in *Enicospilus*. Although the fore wing sclerites have been traditionally considered to be one of the most useful diagnostic characters, our results indicate that the character states of the fore wing sclerites are sometimes strongly affected by the degree of melanisation and it has been leading to species misidentification. Hence, we should try not to rely only on the character states of fore wing sclerites as much as possible, and should use a combination of more than two morphological characters for accurate species identification. Moreover, sequence data on databases (e.g., GenBank, BOLD, and DDBJ) are sometimes based on misidentified specimens. For example, a sequence of *E. ramidulus* (accession number: AB917966) has been deposited in GenBank based on a misidentification of a *Netelia* species. Therefore, we always have to use not only either DNA barcoding data or morphological characters, but also some other characters to accurately delimit species, as many previous authors have suggested (e.g., Schwarzfeld and Sperling 2014, 2015; Klopfstein et al. 2016; Johansson and Cederberg 2019). Padial et al. (2010) have also suggested that more than three different sources may be needed for reliable species delimitation.

Species richness of *Enicospilus* significantly increases from north to south in Japan (Fig. 57); this latitudinal diversity pattern has not been demonstrated for many groups of ichneumonids (e.g., Townes 1969; Owen and Owen 1974; Gauld and Mitchell 1981; Gauld 1987; Quicke 2015), but is usual in ophionines (e.g., Gauld and Mitchell 1978, 1981; Gauld 1985) and is probably true for some other subfamilies, although the data are not yet available to test this (Veijalainen et al. 2012). Observed species richness did not increase by uniform percentage from north to south, for example fewer species were found in southern zones B and E than in northern zones A and D, presumably because of the smaller amount of suitable habitat and smaller geographic area in some zones. However, the Japanese archipelago is fortunately located in a rather narrow longitudinal range and we have used six latitudinal zones to reduce the effect of regional sampling biases in the analysis, therefore our results provide enough information to describe a latitudinal trend in species richness of *Enicospilus* species.

An overwhelmingly larger number of species in zone F (= Ryûkyûs) is worthy of special mention, especially as the diversity of habitats in Ryûkyûs is apparently narrower than the other zones; these suggest that Ryûkyûs is one of the biodiversity hotspots of *Enicospilus* species. In contrast, although Ogasawara is in the southern subtropical region, only 3 species (*E. laqueatus*, *E. melanocarpus*, and *E. signativentris*: 6% of Japanese *Enicospilus* species) were found there. This is very low species richness compared to 31 species (66% of Japanese species) in Okinawa; this is probably because Ogasawara is a group of small remote oceanic islands and the wasps have arrived there recently, indicating their surprisingly strong dispersal abilities. Moreover, *E. signativentris* is frequently reared from cocoons of agricultural pest moths on leaves of Brassicaceae plants (e.g., Cabbage), suggesting that human activities have facilitated their dispersal.

Nine of 47 (19%) Japanese *Enicospilus* species are endemic to Japan, and the rest (81%) are shared with other countries. Most of the non-endemic species are probably derived from the southern tropics, but some trans-Palaearctic species (*E. combustus* and *E. ramidulus*) probably dispersed from the continental temperate region via the Korean Peninsula or Sakhalin. Moreover, no endemic *Enicospilus* species are recognised in Ogasawara.

In the present study, a total of 47 species are recognised in Japan. However, some species, such as *E. puncticulatus*, probably consist of some potential cryptic species. Many *Enicospilus* species described in the present study and by previous authors have been described based on very few specimens (sometimes only the holotype). The estimated species number in Japan based on the ACE and Chao 1 estimators is ca. 55 species. Therefore, the taxonomy of Japanese *Enicospilus* is not complete.

Sampling of *Enicospilus* is biased to known biodiversity hotspots, as with many other organisms. Ryûkyûs are one of the most famous Japanese collecting sites as well as a biodiversity hotspot, receiving much attention from many collectors and scientists. Because many endemic species are found in Ryûkyûs, this is a good location to study phylogeography and biogeography. Hence, we have had access to more collection events, and many specimens of *Enicospilus*, compared to other regions (Table 9; Fig. 58). Although *Enicospilus* of the northern part of Japan were generally not well sampled, those of Hokkaidô have been rather well collected, because Hokkaidô is also a famous collection site with rich fauna and flora. However, there are not many *Enicospilus* specimens from Nagano Prefecture, although there is one of the most famous Japanese insect collection sites. This is probably because the aim of most insect collectors is to collect diurnal insects, especially longicorn beetles or butterflies, in high elevational Alps.

Regional sampling biases also seem to be related to the distribution of universities with traditional entomological laboratories or of active and large entomological societies. *Enicospilus* as well as all other insects of Hokkaidô, Ehime/Kôchi, and Fukuoka prefectures are well sampled, because of Hokkaidô University (SEHU), Ehime University (EUM), and Kyûshû University (KUEC) respectively. *Enicospilus* of Niigata and Hyôgo prefectures are also well sampled, probably because of the Essa entomological society in Niigata and Teneral in Hyôgo.

In regions which are difficult to access and/or far from large cities (and which are not famous collection sites, nor near entomological laboratories and societies), *Enicospilus* are not well sampled, as is the case for all insects. There are regions of high potential biodiversity which are under-sampled, such as low elevational and coastal laurel forests on the Pacific side and Western Japan, grassland on karst in Chûgoku and Shikoku mountains, and alpine areas of the Chûbu region.

These regional sampling biases affect not only analyses of species richness of *Enicospilus* in the present study, but also our general understanding of biodiversity and how to conserve it. Biodiversity is threatened with decline or loss in many environments. For instance, grasslands and marshes are declining by a progression of plant succession, sometimes due to changes in human land use, and in Japanese forests, over-browsing by a population explosion of deer frequently results in a bare forest floor; thus, many insects found there, including parasitoid wasps, are declining and threatened with extinction (e.g., Takatsuki 2009; Sakata and Yamasaki 2015; Nakahama et al. 2018, 2020). To conserve such insects, we have to gather information on their diversity and distribution, as well as their ecology. Further comprehensive sampling efforts are strongly needed.

More comprehensive sampling can be achieved through a combination of professional study and citizen science, amateur collecting. Either alone will be insufficient. In Japan, there are many people who enjoy entomology, especially field collecting (like hunting) and making private collections. This hobby, and feeling insects to be very special, is often called “Mushi-ya” (Takada 2014). Practitioners of Mushi-ya are very skilled in collecting insects and support faunistic and taxonomic studies as well as all other fields of natural history in Japan. Large numbers of specimens examined in the present study were actually provided by Mushi-ya. Hence, we have to continue to strengthen cooperation with citizen scientists, especially Mushi-ya.

## Conclusions

The study of a large specimen base has been vital for resolving the taxonomic confusion that has surrounded Japanese *Enicospilus*. We can now identify them at a species level and therefore conduct more applied research, although some undescribed or unrecorded species are probably still present in Japan. Species richness of *Enicospilus* in Japan significantly increases from north to south, as in many other ophionines, although a southern group of small remote oceanic islands, Ogasawara, have only 6% of Japanese *Enicospilus* species. However, there are regional sampling biases, and the knowledge of the taxonomy and species richness patterns of Japanese *Enicospilus* is incomplete. Therefore, we need further sampling to fill the regional gaps and complete the taxonomy.

## Supplementary Material

XML Treatment for Enicospilus

XML Treatment for Enicospilus abdominalis

XML Treatment for Enicospilus aciculatus

XML Treatment for Enicospilus acutus

XML Treatment for Enicospilus capensis

XML Treatment for Enicospilus combustus

XML Treatment for Enicospilus concentralis

XML Treatment for Enicospilus dasychirae

XML Treatment for Enicospilus erythrocerus

XML Treatment for Enicospilus flavocephalus

XML Treatment for Enicospilus formosensis

XML Treatment for Enicospilus insinuator

XML Treatment for Enicospilus javanus

XML Treatment for Enicospilus jilinensis

XML Treatment for Enicospilus kikuchii

XML Treatment for Enicospilus kunigamiensis

XML Treatment for Enicospilus laqueatus

XML Treatment for Enicospilus limnophilus

XML Treatment for Enicospilus maruyamanus

XML Treatment for Enicospilus matsumurai

XML Treatment for Enicospilus melanocarpus

XML Treatment for Enicospilus multidens

XML Treatment for Enicospilus nigribasalis

XML Treatment for Enicospilus nigristigma

XML Treatment for Enicospilus nigronotatus

XML Treatment for Enicospilus nigropectus

XML Treatment for Enicospilus pseudoconspersae

XML Treatment for Enicospilus pseudopuncticulatus

XML Treatment for Enicospilus pudibundae

XML Treatment for Enicospilus puncticulatus

XML Treatment for Enicospilus pungens

XML Treatment for Enicospilus ramidulus

XML Treatment for Enicospilus riukiuensis

XML Treatment for Enicospilus sakaguchii

XML Treatment for Enicospilus sauteri

XML Treatment for Enicospilus sharkeyi

XML Treatment for Enicospilus shikokuensis

XML Treatment for Enicospilus shinkanus

XML Treatment for Enicospilus signativentris

XML Treatment for Enicospilus stenophleps

XML Treatment for Enicospilus takakuwai

XML Treatment for Enicospilus tripartitus

XML Treatment for Enicospilus unctus

XML Treatment for Enicospilus vestigator

XML Treatment for Enicospilus xanthocephalus

XML Treatment for Enicospilus yezoensis

XML Treatment for Enicospilus yonezawanus

XML Treatment for Enicospilus zeugos

## Supplementary materials

Supplementary material 1 Table S1. Specimens examined Data type specimen data Explanation note Abbreviations for repositories are as follows: CNC-Canadian National Collection of Insects, Ottawa, Canada; ELMU-Entomological Laboratory, Meijô University, Nagoya, Japan; EMUS-Utah State University Insect Collection (= American Entomological Institute: AEI), Department of Biology, Utah State University, Logan, Utah, USA; EUM-Ehime University Museum, Matsuyama, Japan; HMNH-Hiwa Museum for Natural History, Shôbara, Japan; KPMNH-Kanagawa Prefectural Museum of Natural History, Odawara, Japan; KUEC-Entomological Laboratory, Kyûshû University, Fukuoka, Japan; MNHA-Museum of Nature and Human Activities, Sanda, Japan; NIAES-Institute for Agro-Environmental Sciences, NARO (= National Institute for Agro-Environmental Sciences), Tsukuba, Japan; NHMUK-Natural History Museum, London, United Kingdom (formerly BMNH = British Museum of Natural History); NSMT-National Museum of Nature and Science, Tsukuba, Japan; OMNH-Ōsaka Museum of Natural History, Ōsaka, Japan; SEHU-The Laboratory of Systematic Entomology (= Entomological Institute: EIHU), Hokkaidô University, Sapporo, Japan; TARI-Taiwan Agricultural Research Institute Council of Agriculture, Executive Yuan, Taichung, Taiwan; TPM-Tochigi Prefectural Museum, Utsunomiya, Japan. Abbreviaions for non-repositories are as follows: AT-allotype; F-female; HT-holotype; LCT-lectotype; LT-light traps; M-male; MsT-Malaise traps; PT-paratype; SYT-syntype. Abbreviaions for collection are as follows: JMC, J. Minamikawa collection at NIAES; KUSIG, K. Kusigemati collection at SEHU; SMCM, S. Momoi collection at MNHA; TIC, T. Ishii collection at NIAES; SAC, S. Asahina collection at NSMT. https://binary.pensoft.net/file/473826This dataset is made available under the Open Database License (http://opendatacommons.org/licenses/odbl/1.0/). The Open Database License (ODbL) is a license agreement intended to allow users to freely share, modify, and use this Dataset while maintaining this same freedom for others, provided that the original source and author(s) are credited.So Shimizu

Supplementary material 2 Table S2. Specimen data used in the DNA barcoding analyses Data type molecular data Explanation note Abbreviations for identifiers: AB, Andrew Bennett; DJ, Daniel Janzen; DQ, Donald L.J. Quicke; PR, Pascal Rousse; and SS, So Shimizu. Abbreviations for countries: BEN, Benin; BLZ, Belize; CAN, Canada; CRI, Costa Rica; ENG, England; ESP, Spain; GUF, French Guiana; JPN, Japan; LAO, Laos; MDG, Madagascar; MYS, Malaysia; PYF, French Polynesia; THA, Thailand; TWN, Taiwan; and ZAF, South Africa. https://binary.pensoft.net/file/473827This dataset is made available under the Open Database License (http://opendatacommons.org/licenses/odbl/1.0/). The Open Database License (ODbL) is a license agreement intended to allow users to freely share, modify, and use this Dataset while maintaining this same freedom for others, provided that the original source and author(s) are credited.So Shimizu

Supplementary material 3 Checklist and nomenclatural summary of the Japanese species of *Enicospilus* Data type species list https://binary.pensoft.net/file/473828This dataset is made available under the Open Database License (http://opendatacommons.org/licenses/odbl/1.0/). The Open Database License (ODbL) is a license agreement intended to allow users to freely share, modify, and use this Dataset while maintaining this same freedom for others, provided that the original source and author(s) are credited.So Shimizu
