# Supplementary material for: Integrative taxonomy and analysis of species richness patterns of nocturnal Darwin wasps of the genus Enicospilus Stephens (Hymenoptera, Ichneumonidae, Ophioninae) in Japan
Source: Zookeys. 2020 Nov 10;990:1–144. doi: 10.3897/zookeys.990.55542 (PMC7674391; doi:10.3897/zookeys.990.55542)
Supplement: Supplementary material 2 — Table S2. Specimen data used in the DNA barcoding analyses [file zookeys-990-001-s002.docx]

**Supplementary material 2**

Author: So Shimizu

Data type: molecular data

**Table S2**. Specimen data used in the DNA barcoding analyses. Abbreviations for identifiers: AB, Andrew Bennett; DJ, Daniel Janzen; DQ, Donald L.J. Quicke; PR, Pascal Rousse; and SS, So Shimizu. Abbreviations for countries: BEN, Benin; BLZ, Belize; CAN, Canada; CRI, Costa Rica; ENG, England; ESP, Spain; GUF, French Guiana; JPN, Japan; LAO, Laos; MDG, Madagascar; MYS, Malaysia; PYF, French Polynesia; THA, Thailand; TWN, Taiwan; and ZAF, South Africa.

| **Species** | **Identifier** | **Locality** | **Sample code** | **Accession number** |
| --- | --- | --- | --- | --- |
| **Ingroups (*Enicospilus*)** |  |  |  |  |
| *E*. *abdominalis* | SS | TWN: Nantou: Ren’ai | SEN44 | LC484177 |
| *E*. *abdominalis* | SS | TWN: Nantou: Ren’ai | SEN45 | LC484178 |
| *E*. *abessyniensis* | DQ | BEN | - | JF963317 |
| *E*. *aciculatus* | SS | JPN: Ryûkyûs: Okinawa | SEN204 | LC484189 |
| ***E*. *acutus* sp. nov.** | SS | JPN: Honshû: Hyôgo | SEN16 | LC484181 |
| ***E*. *acutus* sp. nov.** | SS | JPN: Honshû: Hyôgo | SEN17 | LC484182 |
| ***E*. *acutus* sp. nov.** | SS | JPN: Shikoku: Ehime | SEN78 | LC484183 |
| ***E*. *acutus* sp. nov.** | SS | TWN: Pingtung: Chunri | SEN130 | LC484184 |
| *E*. *aequalis* | PR | PYF: Tahiti Is. | - | KX053087 |
| *E*. *aequalis* | PR | PYF: Tahiti Is. | - | KX053086 |
| *E*. *aequalis* | PR | PYF: Tahiti Is. | - | KX053085 |
| *E*. *aequalis* | PR | PYF: Tahiti Is. | - | KX053084 |
| *E*. *aequalis* | PR | PYF: Tahiti Is. | - | KX053083 |
| *E*. *bozai* | DJ | CRI: Guanacaste | - | JF793019 |
| *E*. *bozai* | - | CRI | - | FN662467 |
| *E*. *cameronii* | - | CRI: Guanacaste | - | JQ576060 |
| *E*. *cameronii* | - | CRI: Guanacaste | - | HQ548493 |
| *E*. *cameronii* | DJ | CRI: Guanacaste | - | JF793022 |
| *E*. *chiriquensis* | DJ | CRI: Guanacaste | - | JF793023 |
| *E*. *colini* | DJ | CRI: Guanacaste | - | JF793024 |
| *E*. *combustus* | SS | JPN: Hokkaidô: Sapporo | SEN32 | LC484315 |
| *E*. *combustus* | SS | JPN: Hokkaidô: Sapporo | SEN34 | LC484312 |
| *E*. *concentralis* | SS | JPN: Shikoku: Tokushima | SEN100 | LC484318 |
| *E*. *concentralis* | SS | JPN: Shikoku: Kôchi | SEN101 | LC484321 |
| *E*. *dasychirae* | SS | JPN: Ryûkyûs: Okinawa | SEN99 | LC484322 |
| *E*. *dolosus* | SS | TWN: Nantou: Ren’ai | SEN52 | LC484325 |
| *E*. *dolosus* | SS | TWN: Nantou: Ren’ai | SEN53 | LC484326 |
| *E*. *dolosus* | SS | TWN: Nantou: Ren’ai | SEN54 | LC484327 |
| *E*. *dolosus* | SS | TWN: Nantou: Ren’ai | SEN94 | LC484328 |
| *E*. *dolosus* | SS | TWN: Nantou: Ren’ai | SEN121 | LC484331 |
| *E*. *echeverri* | DJ | CRI: Guanacaste | - | JF793025 |
| *E*. *erythrocerus* | SS | JPN: Ryûkyûs: Okinawa | SEN194 | LC486493 |
| *E*. *exaggeratus* | SS | TWN: Nantou: Ren’ai | SEN51 | LC492946 |
| *E*. *exaggeratus* | SS | LAO: Phabang | SEN63 | LC492947 |
| *E*. *fernaldi* | DJ | CRI: Guanacaste | - | JF793026 |
| *E*. *flavicaput* | SS | THA: Chiang Mai | SEN37 | LC493050 |
| *E*. *flavicaput* | SS | LAO: Phabang | SEN57 | LC493051 |
| *E*. *flavicaput* | SS | LAO: Xieng Khouang | SEN58 | LC493052 |
| *E*. *flavicaput* | SS | TWN: Nantou: Ren’ai | SEN131 | LC493053 |
| *E*. *flavocephalus* | SS | LAO: Phabang | SEN61 | LC493069 |
| *E*. *flavocephalus* | SS | JPN: Kyûshû: Kagoshima | SEN116 | LC493070 |
| *E*. *flavocephalus* | SS | JPN: Ryûkyûs: Okinawa | SEN117 | LC493071 |
| *E*. *fogdenorum* | DJ | CRI: Alajuela | - | JF793028 |
| *E*. *formosensis* | SS | JPN: Shikoku: Ehime | SEN36 | LC493072 |
| *E*. *formosensis* | SS | LAO: Khouang | SEN60 | LC493060 |
| *E*. *formosensis* | SS | JPN: Shikoku: Ehime | SEN77 | LC493061 |
| *E*. *galilea* | DJ | CRI: Alajuela | - | JF793029 |
| *E*. *glabratus* | DJ | CRI: Guanacaste | - | JF793034 |
| *E*. *grammospilus* | SS | LAO: Xaisomboun | SEN64 | LC486489 |
| *E*. *hacha* | - | CRI: Guanacaste | - | JQ575190 |
| *E*. *hacha* | - | CRI: Guanacaste | - | JQ575111 |
| *E. hacha* | DJ | CRI: Guanacaste | - | JF793035 |
| *E. insinuator* | SS | JPN: Ryûkyûs: Okinawa | SEN205 | LC486490 |
| *E. javanus* | SS | JPN: Honshû: Kyôto | SEN103 | LC492916 |
| *E. javanus* | SS | JPN: Ryûkyû: Kagoshima | SEN104 | LC492917 |
| *E. javanus* | SS | TWN: Nantou: Ren’ai | SEN163 | LC492918 |
| *E. kanshirensis* | SS | LAO: Phabang | SEN59 | LC493062 |
| *E. kelloggae* | DJ | CRI: Guanacaste | - | JF793039 |
| *E. kikuchii* | SS | JPN: Shikoku: Kôchi | SEN108 | LC492906 |
| *E. kikuchii* | SS | JPN: Honshû: Fukui | SEN109 | LC492907 |
| *E. kleini* | DJ | CRI: Guanacaste | - | JF793040 |
| *E. lacsa* | DJ | CRI: Alajuela | - | JF793041 |
| *E. laqueatus* | SS | JPN: Ogasawara | SEN76 | LC492912 |
| *E. laqueatus* | SS | JPN: Ogasawara | SEN188 | LC492913 |
| *E. lebophagus* | DJ | CRI: Guanacaste | - | JF793042 |
| *E. leoni* | DJ | CRI: Guanacaste | - | JF793043 |
| *E. liesneri* | DJ | CRI: Guanacaste | - | JF793044 |
| ***E. limnophilus* sp. nov.** | SS | JPN: Honshû: Fukui | SEN174 | LC486396 |
| ***E. limnophilus* sp. nov.** | SS | JPN: Honshû: Fukui | SEN175 | LC486397 |
| *E. luisi* | DJ | CRI: Guanacaste | - | JF793045 |
| *E. maai* | SS | TWN: Nantou: Ren’ai | SEN48 | LC486702 |
| *E. maai* | SS | TWN: Nantou: Ren’ai | SEN49 | LC486703 |
| *E. maculipennis* | - | CRI: Guanacaste | - | JQ576040 |
| *E. maculipennis* | DJ | CRI: Guanacaste | - | JF793046 |
| *E. madrigalae* | DJ | CRI: Guanacaste | - | JF793047 |
| *E. major* | DJ | CRI: Alajuela | - | JF793048 |
| *E. maruyamanus* | SS | JPN: Honshû: Niigata | SEN22 | LC493073 |
| *E. maruyamanus* | SS | JPN: Hokkaidô: Chitose | SEN89 | LC493074 |
| *E. maruyamanus* | SS | JPN: Shikoku: Ehime | SEN90 | LC493075 |
| *E. maruyamanus* | SS | JPN: Ryûkyûs: Okinawa | SEN92 | LC493076 |
| *E. melanocarpus* | SS | JPN: Ryûkyûs: Okinawa | SEN15 | LC503974 |
| *E. melanocarpus* | PR | PYF: Huahine Is. | - | KX053088 |
| *E. melanocarpus* | PR | PYF: Huahine Is. | - | KX053089 |
| ***E. multidens* stat. rev.** | SS | JPN: Hokkaidô: Assabu | SEN40 | LC492904 |
| ***E. multidens* stat. rev.** | SS | JPN: Shikoku: Ehime | SEN73 | LC492905 |
| *E. nigribasalis* | SS | JPN: Honshû: Hyôgo | SEN21 | LC503975 |
| *E. nigribasalis* | SS | TWN: Nantou: Ren’ai | SEN93 | LC486699 |
| *E. nigribasalis* | SS | JPN: Shikoku: Kôchi | SEN118 | LC503977 |
| *E. nigristigma* | SS | JPN: Ryûkyûs: Okinawa | SEN110 | LC493179 |
| *E. nigristigma* | SS | JPN: Ryûkyûs: Okinawa | SEN111 | LC493180 |
| *E. nigronotatus* | SS | TWN: Nantou: Ren’ai | SEN50 | LC503976 |
| *E. nigropectus* | SS | JPN: Ryûkyûs: Okinawa | SEN115 | LC493191 |
| *E. pallidistigma* | SS | TWN: Nantou: Ren’ai | SEN133 | LC486700 |
| *E. pescadori* | DJ | CRI: Guanacaste | - | JF793051 |
| *E. pinguivena* | SS | TWN | SEN199 | LC486701 |
| *E. pseudoconspersae* | SS | JPN: Honshû: Hyôgo | SEN19 | LC492924 |
| *E. pseudoconspersae* | SS | JPN: Shikoku: Tokushima | SEN102 | LC492925 |
| ***E*. *pseudopuncticulatus* sp. nov.** | SS | JPN: Honshû: Ōsaka | SEN147 | LC492932 |
| ***E*. *pseudopuncticulatus* sp. nov.** | SS | JPN: Honshû: Ōsaka | SEN176 | LC492933 |
| *E. pudibundae* | SS | JPN: Honshû: Hyôgo | SEN20 | LC493192 |
| *E. pudibundae* | SS | JPN: Honshû: Wakayama | SEN86 | LC493193 |
| *E. pudibundae* | SS | JPN: Honshû: Niigata | SEN87 | LC493194 |
| *E. pudibundae* | SS | JPN: Shikoku: Tokushima | SEN88 | LC493195 |
| *E. pudibundae* | SS | JPN: Shikoku: Kôchi | SEN155 | LC493196 |
| *E. puncticulatus* | SS | JPN: Honshû: Niigata | SEN23 | LC492934 |
| *E. puncticulatus* | SS | JPN: Honshû: Hyôgo | SEN140 | LC492935 |
| *E. puncticulatus* | SS | JPN: Honshû: Niigata | SEN143 | LC492936 |
| *E. puncticulatus* | SS | JPN: Hokkaidô | SEN149 | LC492937 |
| *E. puncticulatus* | SS | JPN: Honshû: Nagano | SEN150 | LC492938 |
| *E. puncticulatus* | SS | JPN: Honshû: Nagano | SEN151 | LC492939 |
| *E. puncticulatus* | SS | JPN: Shikoku: Kôchi | SEN160 | LC492940 |
| *E. puncticulatus* | SS | TWN: Nantou: Ren’ai | SEN120 | LC486478 |
| *E. puncticulatus* | SS | TWN: Nantou: Ren’ai | SEN122 | LC486479 |
| *E. puncticulatus* | SS | TWN: Nantou: Ren’ai | SEN125 | LC486480 |
| *E. pungens* | SS | JPN: Honshû: Kyôto | SEN80 | LC493043 |
| *E. pungens* | SS | JPN: Honshû: Hyôgo | SEN81 | LC493044 |
| *E. pungens* | SS | JPN: Shikoku: Ehime | SEN83 | LC493045 |
| *E. pungens* | SS | JPN: Shikoku: Kôchi | SEN84 | LC493046 |
| *E. pungens* | SS | JPN: Honshû: Niigata | SEN85 | LC493047 |
| *E. ramidulus* | SS | JPN: Honshû: Ōsaka | SEN184 | LC503979 |
| *E. ramidulus* | DQ | ENG | - | JF963318 |
| *E. randalli* | DJ | CRI: Guanacaste | - | JF793052 |
| *E. riukiuensis* | SS | TWN: Pingtung | SEN68 | LC503978 |
| *E. riukiuensis* | SS | JPN: Shikoku: Kôchi | SEN70 | LC493181 |
| *E. riukiuensis* | SS | JPN: Honshû: Wakayama | SEN112 | LC493182 |
| *E. sakaguchii* | SS | JPN: Ryûkyûs: Okinawa | SEN171 | LC493209 |
| *E. sanchezi* | DQ | BLZ | - | JF963319 |
| *E. sauteri* | SS | TWN: Nantou: Ren’ai | SEN139 | LC493210 |
| ***E. sharkeyi* sp. nov.** | SS | JPN: Hokkaidô: Sapporo | SEN33 | LC486392 |
| ***E. sharkeyi* sp. nov.** | SS | JPN: Shikoku: Tokushima | SEN145 | LC486393 |
| *E. shikokuensis* | SS | JPN: Honshû: Hyôgo | SEN41 | LC492900 |
| *E. shikokuensis* | SS | JPN: Honshû: Niigata | SEN42 | LC492901 |
| *E. shinkanus* | SS | JPN: Ryûkyûs: Okinawa | SEN106 | LC492926 |
| *E. shinkanus* | SS | JPN: Honshû: Niigata | SEN107 | LC492927 |
| *E. signativentris* | SS | TWN: Nantou: Ren’ai | SEN95 | LC486706 |
| *E. signativentris* | SS | JPN: Honshû: Ōsaka | SEN97 | LC493204 |
| *E. signativentris* | SS | JPN: Honshû: Wakayama | SEN98 | LC493205 |
| *E. signativentris* | SS | TWN: Pingtung: Mudan | SEN123 | LC486707 |
| *E. signativentris* | SS | JPN: Shikoku: Kôchi | SEN162 | LC492941 |
| *E. simoni* | DJ | CRI: Guanacaste | - | JF793057 |
| *E. stenophleps* | SS | JPN: Ryûkyûs: Okinawa | SEN24 | LC508267 |
| *E. stenophleps* | SS | JPN: Ryûkyûs: Okinawa | SEN203 | LC486492 |
| *E. stenophleps* | SS | TWN: Nantou: Ren’ai | SEN119 | LC486705 |
| *E. stenophleps* | SS | TWN: Nantou: Ren’ai | SEN134 | LC486704 |
| *E. transversus* | SS | LAO: Phabang | SEN62 | LC492960 |
| *E. tripartitus* | SS | JPN: Shikoku: Tokushima | SEN74 | LC493206 |
| *E. tripartitus* | SS | JPN: Shikoku: Tokushima | SEN75 | LC493207 |
| *E. tripartitus* | SS | JPN: Shikoku: Tokushima | SEN183 | LC508266 |
| *E. tripartitus* | SS | JPN: Shikoku: Tokushima | SEN192 | LC493208 |
| *E. ugaldei* | DJ | CRI: Guanacaste | - | JF793059 |
| *E. umbratus* | DQ | MDG | - | JF963320 |
| *E. vestigator* | SS | JPN: Ryûkyûs: Okinawa | SEN202 | LC486494 |
| *E. xanthocephalus* | SS | JPN: Ryûkyûs: Okinawa | SEN113 | LC493183 |
| *E. xanthocephalus* | SS | JPN: Ryûkyûs: Okinawa | SEN114 | LC493184 |
| *E*. *yezoensis* | SS | JPN: Honshû: Yamagata | SEN28 | LC493058 |
| *E*. *yezoensis* | SS | JPN: Honshû: Yamagata | SEN29 | LC493059 |
| *E*. *yezoensis* | SS | JPN: Honshû: Hyôgo | SEN141 | LC492948 |
| *E*. *yezoensis* | SS | JPN: Honshû: Aomori | SEN156 | LC492949 |
| *E*. *yonezawanus* | SS | LAO: Phabang | SEN67 | LC492950 |
| *E*. *yonezawanus* | SS | JPN: Ryûkyûs: Okinawa | SEN173 | LC492951 |
| ***E*. *zeugos* stat. rev.** | SS | JPN: Ryûkyûs: Okinawa | SEN208 | LC486491 |
| *Enicospilus* sp. MY1 | SS | MYS: Kelantan | SEN79 | LC493197 |
| *Enicospilus* sp. T4 | SS | TWN: Nantou: Ren’ai | SEN138 | LC503981 |
| *Enicospilus* sp. T5 | SS | TWN: Nantou: Ren’ai | SEN126 | LC503980 |
| *Enicospilus* sp. T7 | SS | TWN: Nantou | SEN129 | LC492921 |
| *Enicospilus* sp. | - | CAN | - | JN288781 |
| *Enicospilus* sp. | AB | CAN: NL | - | MG482162 |
| *Enicospilus* sp. G | SS | GUF | SEN27 | LC503982 |
| **Outgroups (all from Ophioninae)** |  |  |  |  |
| *Dicamptus* sp. | SS | JPN: Honshû: Tsushima | Dica1 | LC503984 |
| *Ophion* sp. | SS | - | SHOP212 | LC503983 |
| *Leptophion maculipennis* | SS | TWN | LM1 | LC503985 |
| *Afrophion hynnis* | DQ | ZAF | JM177 | JF962473 |
| *Hellwigiella dichromoptera* | DQ | ESP | JM330 | JF957051 |
| *Rhynchophion flammipennis* | DJ | CRI: Guanacaste | DHJPAR0000295 | JF793291 |
| *Thyreodon laticinctus* | DJ | CRI: Alajuela | DHJPAR0000517 | JF793300 |
