# Supplementary material for: Integrative taxonomy and analysis of species richness patterns of nocturnal Darwin wasps of the genus Enicospilus Stephens (Hymenoptera, Ichneumonidae, Ophioninae) in Japan
Source: Zookeys. 2020 Nov 10;990:1–144. doi: 10.3897/zookeys.990.55542 (PMC7674391; doi:10.3897/zookeys.990.55542)
Supplement: Supplementary material 3 — Checklist and nomenclatural summary of the Japanese species of Enicospilus [file zookeys-990-001-s003.docx]

**Supplementary material 3**

Author: So Shimizu

Data type: species list

**Checklist and nomenclatural summary of the Japanese species of *Enicospilus*.**

1. ***Enicospilus abdominalis* (Szépligeti, 1906)**

*Henicospilus abdominalis* Szépligeti, 1906: 138.

*Ophion semiopacus* Matsumura, 1912: 114.

1. ***Enicospilus aciculatus* (Taschenberg, 1875)**

*Ophion aciculatus* Taschenberg, 1875: 434.

*Enicospilus malaitensis* Brues, 1918: 117.

*Henicospilus okinawensis* Matsumura & Uchida, 1926: 71.

*Henicospilus neddenveli* Cheesman, 1936: 185.

*Enicospilus crucis* Chiu, 1954: 70.

1. ***Enicospilus acutus* Shimizu sp. nov.**
2. ***Enicospilus capensis* (Thunberg, 1822)**

*Ichneumon capensis* Thunberg, 1824: 262.

*Ophion lativertex* Taschenberg, 1875: 435.

*Ophion antankarus* Saussure, 1892: 15.

*Henicospilus montinus* Enderlein, 1921: 21.

*Henicospilus praedator* Enderlein, 1921: 28.

*Henicospilus incarinatus* Enderlein, 1921: 30.

*Henicospilus euxoae* Wilkinson, 1928: 261.

*Enicospilus obnoxius* Seyrig, 1935: 75.

*Henicospilus yanagiharai* Sonan, 1940: 371.

*Enicospilus selvaraji* Rao & Kurian, 1950: 174, 178, 180, 188; nomen nudum.

*Enicospilus selvaraji* Rao & Kurian, 1951: 68.

*Enicospilus fossatus* Chiu, 1954: 63.

*Enicospilus indica* Rao & Grover, 1960: 280.

1. ***Enicospilus combustus* (Gravenhorst, 1829)**

*Ophion combustus* Gravenhorst, 1829: 701.

1. ***Enicospilus concentralis* Cushman, 1937**

*Enicospilus concentralis* Cushman, 1937: 305.

1. ***Enicospilus dasychirae* Cameron, 1905**

*Eniscospilus* (*sic*) *dasychirae* Cameron, 1905a: 123.

*Eniscospilus* (*sic*) *horsfieldi* Cameron, 1905a: 124.

*Henicospilus borneensis* Szépligeti, 1906: 138.

*Enicospilus nigrimarginalis* Cushman, 1937: 311.

1. ***Enicospilus erythrocerus* (Cameron, 1905)**

*Pleuroneurophion erythrocerus* Cameron, 1905a: 121.

*Allocamptus orientalis* Uchida, 1928: 230; junior secondary homonym of *Enicospilus orientalis* (Morley, 1913).

*Enicospilus hirayamai* Uchida, 1955: 120; replacement name for *Enicospilus orientalis* (Uchida, 1928).

1. ***Enicospilus flavocephalus* (Kirby, 1900)**

*Ophion flavocephalus* Kirby, 1900: 82.

*Henicospilus lunulatus* Szépligeti, 1906: 143.

*Henicospilus albicaput* Morley, 1912: 50.

*Henicospilus similis* Matsumura & Uchida, 1926: 221.

1. ***Enicospilus formosensis* (Uchida, 1928)**

*Henicospilus formosensis* Uchida, 1928: 223.

*Enicospilus saepis* Chiu, 1954: 77.

*Enicospilus vacuus* Gauld & Mitchell, 1981: 453; **syn. nov.**

1. ***Enicospilus insinuator* (Smith, 1860)**

*Ophion insinuator* Smith, 1860: 141.

*Enicospilus zyzzus* Chiu, 1954: 23.

1. ***Enicospilus javanus* (Szépligeti, 1910)**

*Henicospilus javanus* Szépligeti, 1910: 93.

*Enicospilus fulacorensis* Brues, 1918: 117.

*Enicospilus gephyrus* Chiu, 1954: 32.

*Enicospilus* (*Bicorniata*) *diurnus* Nikam, 1975: 193, 194.

1. ***Enicospilus jilinensis* Tang, 1990 (new to Japan)**

*Enicospilus jilinensis* Tang, 1990: 72.

1. ***Enicospilus kikuchii* Shimizu, 2017**

*Enicospilus kikuchii* Shimizu, 2017: 187.

1. ***Enicospilus kunigamiensis* Shimizu sp. nov.**
2. ***Enicospilus laqueatus* (Enderlein, 1921) (new to Japan)**

*Henicospilus laqueatus* Enderlein, 1921: 26.

*Enicospilus leetoni* Chiu, 1954: 38.

1. ***Enicospilus limnophilus* Shimizu sp. nov.**
2. ***Enicospilus maruyamanus* (Uchida, 1928)**

*Henicospilus maruyamanus* Uchida, 1928: 220.

1. ***Enicospilus matsumurai* Shimizu sp. nov.**
2. ***Enicospilus melanocarpus* Cameron, 1905**

*Enicospilus reticulatus* Cameron, 1902: 52; junior primary homonym of *Enicospilus reticulatus* Cameron, 1899.

*Eniscospilus* (*sic*) *melanocarpus* Cameron, 1905a: 122.

*Henicospilus nigrinervis* Szépligeti, 1906: 142; junior secondary homonym of *Enicospilus nigrinervis* Cameron, 1901.

*Ophion* (*Henicospilus*) *nocturnus* Kohl, 1908: 315.

*Henicospilus batavianus* Szépligeti, 1910: 92.

*Henicospilus turneri* Morley, 1912: 51.

*Henicospilus atricornis* var*. zeylanicus* Morley, 1913: 392.

*Henicospilus uncivena* Enderlein, 1921: 23.

*Henicospilus crassivena* Enderlein, 1921: 24.

*Enicospilus nigrivenalis* Cushman, 1937: 307.

*Enicospilus quintuplex* Chiu, 1954: 61.

*Enicospilus* (*Polycorniata*) *brunnis* Rao & Nikam, 1971: 105.

1. ***Enicospilus multidens* Chiu, 1954 stat. rev.**

*Enicospilus multidens* Chiu, 1954: 75; **stat. rev.**

1. ***Enicospilus* *nigribasalis* (Uchida, 1928)**

*Henicospilus nigribasalis* Uchida, 1928: 222.

1. ***Enicospilus nigristigma* Cushman, 1937**

*Enicospilus nigristigma* Cushman, 1937: 309.

1. ***Enicospilus nigronotatus* Cameron, 1903**

*Enicospilus nigronotatus* Cameron, 1903: 133.

*Henicospilus triguttatus* Uchida, 1928: 221.

1. ***Enicospilus nigropectus* Cameron, 1905**

*Enicospilus nigropectus* Cameron, 1905b: 123.

*Henicospilus hariolus* Morley, 1912: 44.

*Amesospilus nigrostemmaticus* Enderlein, 1921: 19.

*Henicospilus fuscomaculatus* Uchida, 1928: 216.

*Henicospilus fuscomaculatus yakushimensis* Yasumatsu, 1934: 67.

1. ***Enicospilus pseudoconspersae* (Sonan, 1927)**

*Henicospilus pseudoconspersae* Sonan, 1927: 48.

*Henicospilus mushanus* Uchida, 1928: 216.

*Enicospilus tenuinubeculus* Chiu, 1954: 34.

1. ***Enicospilus pseudopuncticulatus* Shimizu sp. nov.**
2. ***Enicospilus pudibundae* (Uchida, 1928)**

*Henicospilus pudibundae* Uchida, 1928: 219.

1. ***Enicospilus puncticulatus* Tang, 1990 (new to Japan)**

*Enicospilus puncticulatus* Tang, 1990: 128.

1. ***Enicospilus pungens* (Smith, 1874)**

*Ophion pungens* Smith, 1874: 396.

*Enicospilus striatus* Cameron, 1899: 103; **syn. nov.**

*Henicospilus lineolatus* Roman, 1913: 30; **syn. nov.**

*Enicospilus uniformis* Chiu, 1954: 25; **syn. nov.**

*Enicospilus flatus* Chiu, 1954: 28; **syn. nov.**

*Enicospilus gussakovskii* Viktorov, 1957: 185; **syn. nov.**

*Enicospilus striolatus* Townes, Townes & Gupta, 1961: 290; replacement name for *Enicospilus striatus* Cameron, 1899; **syn. nov.**

*Enicospilus unicornis* Rao & Nikam, 1969: 343; **syn. nov.**

*Enicospilus unicornis* Rao & Nikam, 1970: 103; **syn. nov.**

1. ***Enicospilus ramidulus* (Linnaeus, 1758)**

*Ichneumon ramidulus* Linnaeus, 1758: 566.

1. ***Enicospilus riukiuensis* (Matsumura & Uchida, 1926)**

*Henicospilus riukiuensis* Matsumura & Uchida, 1926: 71.

*Enicospilus nasutus* Chiu, 1954: 65.

*Enicospilus vontalis* Gauld & Mitchell, 1978: 125.

1. ***Enicospilus sakaguchii* (Matsumura & Uchida, 1926)**

*Henicospilus sakaguchii* Matsumura & Uchida, 1926: 73.

*Enicospilus iracundus* Chiu, 1954: 17; **syn. nov.**

1. ***Enicospilus sauteri* (Enderlein, 1921)**

*Henicospilus sauteri* Enderlein, 1921: 84.

*Henicospilus analis* Matsumura & Uchida, 1926: 72.

*Enicospilus molopos* Chiu, 1954: 57.

1. ***Enicospilus sharkeyi* Shimizu sp. nov.**
2. ***Enicospilus shikokuensis* (Uchida, 1928)**

*Henicospilus combustus* var. *shikokuensis* Uchida, 1928: 224.

*Enicospilus seniculus* Chiu, 1954: 71.

*Enicospilus sigmatoides* Chiu, 1954: 75; **syn. nov.**

1. ***Enicospilus shinkanus* (Uchida, 1928)**

*Henicospilus shinkanus* Uchida, 1928: 217.

*Henicospilus yamanakai* Uchida, 1930: 83; **syn. nov.**

*Henicospilus pankumensis* Cheesman, 1936: 184.

*Enicospilus relictus* Chiu, 1954: 20.

*Enicospilus* (*Unicorniata*) *bindus* Nikam, 1972: 194.

1. ***Enicospilus signativentris* (Tosquinet, 1903)**

*Ophion* (*Enicospilus*) *signativentris* Tosquinet, 1903: 37.

*Henicospilus incompletus* Szépligeti, 1906: 143.

*Henicospilus nigrosignatus* Enderlein, 1921: 22.

*Henicospilus tristrigatus* Enderlein, 1921: 23.

*Henicospilus formosanus* Enderlein, 1921: 25.

*Henicospilus emacescens* Enderlein, 1921: 25.

*Henicospilus taiwanus* Uchida, 1928: 226.

*Enicospilus frater* Cushman, 1937: 311.

*Enicospilus pectiniclavae* Rao & Nikam, 1969: 14*.*

1. ***Enicospilus stenophleps* Cushman, 1937 (new to Japan)**

*Enicospilus stenophleps* Cushman, 1937: 309.

1. ***Enicospilus takakuwai* Shimizu sp. nov.**
2. ***Enicospilus tripartitus* Chiu, 1954**

*Enicospilus tripartitus* Chiu, 1954: 36.

1. ***Enicospilus unctus* Shimizu sp. nov.**
2. ***Enicospilus vestigator* (Smith, 1858) (new to Japan)**

*Ophion vestigator* Smith, 1858: 122.

*Eniscospilus* (*sic*) *unilineatus* Cameron, 1905a: 123.

*Henicospilus xantusi* Szépligeti, 1906: 138.

*Enicospilus receptor* Chiu, 1954: 40.

*Enicospilus glabrifascies* Chiu, 1954: 40.

*Enicospilus* (*Polycorniata*) *carinatus* Rao & Nikam, 1971: 103.

1. ***Enicospilus xanthocephalus* Cameron, 1905**

*Eniscospilus* (sic) *xanthocephalus* Cameron, 1905a: 122.

*Enicospilus bullatus* Chiu, 1954: 53.

*Enicospilus obliquus* Chiu, 1954: 54; junior secondary homonym of *Enicospilus obliquus* (Morley, 1912).

*Enicospilus* clinatus Townes, Townes & Gupta, 1961: 272; replacement name for *Enicospilus obliquus* Chiu, 1954.

*Enlcospilus* (sic) (*Bicorn’ata*) (sic) *paraclinatus* Nikam, 1975: 198.

*Enicospilus pexus* Gauld, 1977: 57, 86.

1. ***Enicospilus yezoensis* (Uchida, 1928)**

*Henicospilus yezoensis* Uchida, 1928: 227.

*Enicospilus ranunculus* Chiu, 1954: 36; **syn. nov.**

1. ***Enicospilus yonezawanus* (Uchida, 1928)**

*Henicospilus yonezawanus* Uchida, 1928: 218.

*Enicospilus microstriatellus* Uchida, 1956: 95.

1. ***Enicospilus zeugos* Chiu, 1954 stat. rev. (new to Japan)**

*Enicospilus zeugos* Chiu, 1954: 64; **stat. rev.**

*Enicospilus henrytownesi* Chao & Tang, 1991: 51; **syn. nov.**
